# Contrast Agent Dynamics Determine Radiomics Profiles in Oncologic Imaging

**DOI:** 10.3390/cancers16081519

**Published:** 2024-04-16

**Authors:** Martin L. Watzenboeck, Lucian Beer, Daria Kifjak, Sebastian Röhrich, Benedikt H. Heidinger, Florian Prayer, Ruxandra-Iulia Milos, Paul Apfaltrer, Georg Langs, Pascal A. T. Baltzer, Helmut Prosch

**Affiliations:** 1 Department of Biomedical Imaging and Image-Guided Therapy, Medical University of Vienna, 1090 Vienna, Austriageorg.langs@meduniwien.ac.at (G.L.); pascal.baltzer@meduniwien.ac.at (P.A.T.B.); helmut.prosch@meduniwien.ac.at (H.P.); 2 Computational Imaging Research Lab, Christian Doppler Laboratory for Machine Learning Driven Precision Medicine, Department of Biomedical Imaging and Image-Guided Therapy, Medical University of Vienna, 1090 Vienna, Austria; 3 Zentralröntgeninstitut für Diagnostik, Interventionelle Radiologie und Nuklearmedizin, Landesklinikum Wiener Neustadt, 2700 Wiener Neustadt, Austria

**Keywords:** radiomics, machine-learning, contrast agent, lung cancer, prostate cancer

## Abstract

**Simple Summary:**

Radiomics is the extraction of high-dimensional quantitative features from medical images. These features have shown potential to provide diagnostic and prognostic information in various oncological settings, but their reproducibility and stability can be affected by multiple physiological and technical factors. In this study, we assessed the effect of contrast agent timing on radiomics features using dynamic contrast-enhanced CT or MRI in prostate and lung cancers, whereby multiple images are acquired with a high temporal resolution after contrast agent application. We observed that a substantial number of radiomics features are contrast perfusion dependent and prone to bias. Therefore, when using contrast-enhanced scans for radiomics studies, patient-specific perfusion timing (e.g., test bolus protocol) should be applied.

**Abstract:**

Background: The reproducibility of radiomics features extracted from CT and MRI examinations depends on several physiological and technical factors. The aim was to evaluate the impact of contrast agent timing on the stability of radiomics features using dynamic contrast-enhanced perfusion CT (dceCT) or MRI (dceMRI) in prostate and lung cancers. Methods: Radiomics features were extracted from dceCT or dceMRI images in patients with biopsy-proven peripheral prostate cancer (pzPC) or biopsy-proven non-small cell lung cancer (NSCLC), respectively. Features that showed significant differences between contrast phases were identified using linear mixed models. An L2-penalized logistic regression classifier was used to predict class labels for pzPC and unaffected prostate regions-of-interest (ROIs). Results: Nine pzPC and 28 NSCLC patients, who were imaged with dceCT and/or dceMRI, were included in this study. After normalizing for individual enhancement patterns by defining seven individual phases based on a reference vessel, 19, 467 and 128 out of 1204 CT features showed significant temporal dynamics in healthy prostate parenchyma, prostate tumors and lung tumors, respectively. CT radiomics-based classification accuracy of healthy and tumor ROIs was highly dependent on contrast agent phase. For dceMRI, 899 and 1027 out of 1118 features were significantly dependent on time after contrast agent injection for prostate and lung tumors. Conclusions: CT and MRI radiomics features in both prostate and lung tumors are significantly affected by interindividual differences in contrast agent dynamics.

## 1. Introduction

Radiomics is the automated high-throughput extraction of a large number of quantitative features from radiologic/medical imaging data [1]. While radiomics features have successfully been applied in oncologic imaging to gain insights into tumor biology [2,3] and predict clinical responses and outcomes [4,5,6,7], the repeatability and reproducibility of such features is influenced by several patient-related and technical factors. For example, inspiration depth, examination protocol, slice thickness and the reconstruction kernel have all been shown to alter the extracted radiomics features [8]. Common practice is to exclude unstable features based on test–retest studies [9,10]. 

The administration of intravenous (i.v.) contrast agent may be another potential source of variation. In oncologic imaging, i.v. contrast agents are used in clinical practice to enhance the tissue contrast between the tumor and surrounding structures. However, the protocols for the timing between the start of i.v. contrast injection and scan acquisition are set individually at the respective institutions or between scanners within the same institution according to local preferences. In addition, even when standardized contrast injection protocols are used, the pharmacokinetics of the injected contrast material can vary substantially from patient to patient due to patient-specific factors, such as the cardiac output and blood volume [11,12]. While these factors might impair the stability of radiomics features extracted from contrast-enhanced computed tomography (CT) or magnetic resonance imaging (MRI) and can limit the use these data in quantitative research settings [13], at present, no comprehensive analyses of the magnitude of these effects exist. The lack of these studies may be the reason that, despite the potential effects of i.v. contrast agents on radiomics features, recently published guidelines on data acquisition and analysis do not address these effects and their consequences for the design of radiomics studies [13,14].

Therefore, the aim of this study was to evaluate the impact of contrast agents on the stability of radiomics features at different timepoints. For the purpose of this study, two tumor entities were examined: peripheral zone prostate cancer (pzPC) and non-small cell lung cancer (NSCLC); each of which are exemplified by distinct contrast agent uptake characteristics. pzPC is characterized by rapid intense contrast agent uptake compared to the surrounding non-tumoral peripheral zone tissue [15], while NSCLC shows a more heterogeneous and less intense uptake [16]. Dynamic contrast-enhanced computed tomography (dceCT) or MRI (dceMRI) images, which provided a high temporal resolution of radiomics profiles after contrast agent application (see graphical abstract), were included in this study. Seven distinct contrast agent phases were defined, and radiomics features which are dependent on contrast agent phase for NSCLC or pzPC tumors were identified. Furthermore, the dependency of contrast agent phases on machine-learning-based differentiation between healthy prostate parenchyma and pzPC lesions were evaluated. Finally, the influence of contrast agent phases on radiomics features could also be observed in dceMRI, where even stronger effects were observed.

## 2. Materials and Methods

### 2.1. Ethics

This retrospective study was approved by the Institutional Review Board of the Medical University of Vienna (Numbers 1764/2016 and 1789/2015) and performed in accordance with the Declaration of Helsinki. Written informed consent was obtained from all patients. 

The cohort of pzPC patients has been described previously [10]. Briefly, patients without contraindications to MRI or CT of the prostate from December 2015 to May 2018, who presented with an MRI-proven suspicious lesion of the prostate (≥PI-RADS 3), and who were scheduled for MRI-guided transrectal biopsy, were eligible for this study. Exclusion criteria were a prior local therapy to the prostate (e.g., brachytherapy) or to other organs in the vicinity. 

MRI protocols and target-MRI in-bore biopsy details were performed as described, and 14 patients with biopsy-proven peripheral zone prostate cancer, who received a multiparametric MRI within a 3-month time window of the dynamic contrast-enhanced CT and/or MRI were further included. For dceCT images, seven of these patients were excluded, since prostate tumors were too small to be reliably segmented on CT. For dceMRI images, five patients were excluded, since tumors could not be reliably segmented on dceMRI. Patient characteristics for the cohort of included prostate cancer patients are shown in Table 1.

The dceCT cohort of NSCLC patients consisted of nine patients with histologically verified adenocarcinoma or squamous cell carcinoma who were scheduled for adjuvant therapy. 

Patient characteristics for the cohort of NSCLC patients imaged with dceCT can be found in Table 2. 

An additional cohort of NSCLC patients, consisting of 21 patients, was imaged with dceMRI of the lung. If available, not only was the initial scan included, but so were follow-up investigations (between one and five investigations per patient), resulting in a total of 36 investigations being included in the final cohort (Table 3).

### 2.2. Inclusion and Exclusion Criteria 

For NSCLC patients the inclusion criteria were as follows: an identifiable thoracic target lesion larger than 1 cm and the ability to undergo a CT examination. Exclusion criteria were radiotherapy within three months of inclusion or low image quality (*n* = 2). 

For pzPC patients the inclusion criteria were as follows: an identifiable thoracic target lesion larger than 0.5 cm and the ability to undergo a CT examination. Exclusion criteria were previous therapy or low image quality.

### 2.3. Dynamic Contrast-Enhanced CT Imaging Protocol 

A SOMATOM Definition Flash CT scanner (Siemens Healthineers, Erlangen, Germany) was utilized for all patients. An a priori performed non-contrast pelvis CT (for pzPC patients) or thoracic CT (for NSCLC patients) was used for planning, in which the prostate or index lesion (for NSCLC patients) was delineated. After i.v. injection of 40 mL of non-ionic iodinated contrast agent at an injection rate of 5 mL/second (Iomeron 400, Bracco Imaging, S.p.A., Rome, Italy), followed by a saline chaser of 40 mL, 26–33 scans of the prostate or 20–30 scans of the tumor in the thorax were acquired. The first 20 examinations were performed at 1.5 s intervals, and the following scans at 3 s intervals, to reduce the radiation dose after the initial arterial influx. A tube voltage of 80 or 90 kVp was used, depending on patient body weight. Slice width was 5 mm for prostate scans and 1.5 mm for lung scans, rotation time 0.25 s, slice collimation 48 × 1.2 mm; and scan length was 114 mm for prostate scans, and 110 or 174 mm for lung scans.

### 2.4. Dynamic Contrast-Enhanced MRI Imaging Protocol for pzPC Patients 

Patients with pzCT were scanned on a Magnetom Trio (A Tim; Siemens Healthineers, Erlangen, Germany) scanner. Dynamic contrast-enhanced (DCE) MRI was acquired using a three-dimensional, T1-weighted gradient echo sequence (TWIST) with a temporal resolution of 4.22 s (FOV 260 mm; matrix 160). Before dynamic scanning, T1-mapping sequences using the variable flip-angle method were applied. Gadoterate meglumine (Gd-DOTA, Dotarem^®^, Guerbet, France) was injected after three baseline scans intravenously as a bolus (0.2 mL/kg body weight) using a power injector at a flow rate of 4 mL/s, followed by a flush of 20 mL of saline solution.

### 2.5. Dynamic Contrast-Enhanced MRI Imaging Protocol for NSCLC Patients 

A SiemensBiograph mMR 3T system (Tim Trio, Siemens Healthineers, Erlangen, Germany) was used to scan NSCLC patients. Dynamic contrast-enhanced (DCE) MRI was acquired using a three-dimensional, T1-weighted gradient echo sequence (TWIST) with a temporal resolution of 11.025 s (FOV 280 mm; matrix 192). Before dynamic scanning, T1-mapping sequences using the variable flip-angle method were applied. Gadoterate meglumine (Gd-DOTA, Dotarem^®^, Guerbet, France) was injected after three baseline scans intravenously as a bolus (0.2 mL/kg body weight) using a power injector at a flow rate of 5 mL/s, followed by a flush of 40 mL of saline solution.

### 2.6. Region-of-Interest Segmentation

Region-of-interest (ROI) segmentation was performed using a Microsoft Advanced Imaging Labeler (Microsoft, 2022, Redmond, Washington, DC, USA) by a radiology resident (M.W., with three years’ experience in oncologic imaging) under the supervision of a board certified radiologist (L.B., with nine years’ experience in oncologic imaging). For pzPC patients, tumors were segmented on consecutive slices in venous phase scans. To investigate whether the dynamics of radiomics features after contrast agent injection differed between tumors and healthy tissue, for pzPC patients imaged with dceCT, an additional ROI was placed in the contralateral, tumor-free prostatic hemisphere. For both dceCT and dceMRI of pzPC patients, a cylindric ROI was placed in the common femoral artery lumen. For NSCLC patients, the best visible (index) lesion was segmented in a venous phase scan, which was the primary tumor in four patients, and a lymph node metastasis in three patients imaged with dceCT, and the primary tumor in all patients was imaged with dceMRI. Analogous to pzPC patients, an additional ROI was placed in the descending aortic lumen. For each patient, masks were propagated across different dynamic contrast CT or MRI series. Propagated masks were inspected visually to ensure that tumors, the healthy prostate parenchyma and the arterial lumen were accurately delineated in all series. Segmentation masks were exported as DICOM-RT files. Both images and segmentation masks were subsequently converted to an NRRD file format using the open source software plastimatch (version 1.9.0, The General Hospital Corporation, Inc., Boston, MA, USA).

### 2.7. Radiomics Feature Extraction

Pyradiomics [17] version 3.0.1 running under Python 3.7.1 was used to extract radiomics features from segmented ROIs. Example settings for CT images, included in the pyradiomics library (specified in the file exampleCT.yaml), were used for feature extractions, and shape features were a priori excluded from the study, since regions were propagated across different dynamic contrast series, and therefore shape features were not expected to change across series. The binWidth parameter (specifying the number of bins for image discretization) was set to 25, the voxelArrayShift parameter to 1000 (to prevent negative values from being squared), sitkBSpline was used for image resampling, resampledPixelSpacing was defined as [1, 1, 1], and padDistance as 10. A total of 13 different filtering transformations were applied to the original images: Laplacian of Gaussian (LoG) edge enhancement filters, with sigma values (defining the coarseness of the emphasized texture) set to 1, 2, 3, 4 and 5 mm; and wavelet filtering for all possible combinations of applying High or Low pass filters along the x, y and z axes, which resulted in eight (23) different combinations. Including the original unfiltered CT scans, this resulted in 14 different sets of images, from which 18 first-order, 22 gray-level co-occurrence matrix (GLCM), 14 gray-level dependence matrix (GLDM), 16 gray-level run length matrix (GLRLM) and 16 gray-level size zone matrix (GLSZM) features (a total of 14 × 86 = 1204 features) were extracted.

For MR images, example settings specified in the file exampleMR_3 mm.yaml were used for feature extractions. The binWidth parameter was set to 5, the voxelArrayShift parameter to 300, sitkBSpline was used for image resampling, resampledPixelSpacing was defined as [2, 2, 2], normalize was set to true, and normalizeScale to 100. A total of 12 filtering transformations (LoG edge enhancement filters, with sigma values set to 2, 3, 4, and 5 mm, and eight different combinations of wavelet filtering with High or Low pass filters) were applied to the original images (13 different sets of images, including the original MRI scans). From the resulting images, 18 first-order, 22 GLCM, 14 GLDM, 16 GLRLM and 16 gray-level size zone matrix (GLSZM) features (a total of 13 × 86 = 1118 features) were extracted.

### 2.8. Statistical Analysis

All statistical analyses were performed using R version 4.0.5 (R Foundation for Statistical Computing, Vienna, Austria) or Python 3.7.1. Custom R functions were used to define contrast agent phases according to arterial lumen median voxel intensity (see Results section). To assess the temporal dynamics after contrast agent injection, linear mixed models (R package lmerTest version 3.1.3) were applied, using the following formulas:

For pzPC and NSCLC dceCT and pzPC dceMRI data:

1. Feature ~ Timepoint+(1|Patient)

For NSCLC dceMRI data, where multiple visits per patient were included:

2. Feature ~ Timepoint+(1|Patient)+(1|Visit)

Therefore, timepoints (after contrast injection) were treated as a fixed covariate and patient identity and visit as random covariates, as applicable. *p*-values for the timepoint covariate were calculated using the Satterthwaite approximation and corrected for multiple testing with the false discovery rate (FDR) method of Benjamini and Hochberg [18]. Features with a *p*-value and FDR of <0.05 were considered to show significant temporal dynamics after contrast agent application. These approaches and thresholds were chosen to maintain a high statistical power, but keep the proportion of false-positives low. For the features wavelet.HHH_firstorder_TotalEnergy, wavelet.HHL_firstorder_TotalEnergy, wavelet.HHL_firstorder_Energy and wavelet.HHH_firstorder_Energy extracted from lung tumor sections, linear mixed models failed to converge. Thus, these features had to be excluded from the analysis. Heatmaps of features with significant temporal dynamics were generated using the R package ComplexHeatmap version 2.6.2, after calculating the mean value for each feature over all patients and applying a z-score transformation over all seven timepoints. Clustering of features with similar temporal dynamics was performed using the complete linkage method on Euclidean distance matrices. For visualization as heatmaps, median voxel intensities of the aortal lumen or radiomics features were standardized across timepoints the using a z-score transformation. This method involves subtracting the mean and dividing by the standard deviation, resulting in transformed data with a mean of zero and a standard deviation of one.

Classification of healthy prostate and tumor regions-of-interest (ROIs) based on CT radiomics features was performed using the Python package scikit-learn version 1.0.2. Logistic regression classifiers with default settings (which uses L2 regularization with regularization strength C = 1) was applied. Receiver operator characteristic (ROC) curves were generated using leave-one-out cross validation, whereby for each of the timepoints, each (healthy or tumor) ROI was iteratively left out; classifiers were trained on the remaining ROIs, and class probability was predicted for the left-out ROI.

## 3. Results

### 3.1. Contrast Agent Dynamics Vary between Patients and Require Standardization for Systematic Analysis

**Figure 1:**
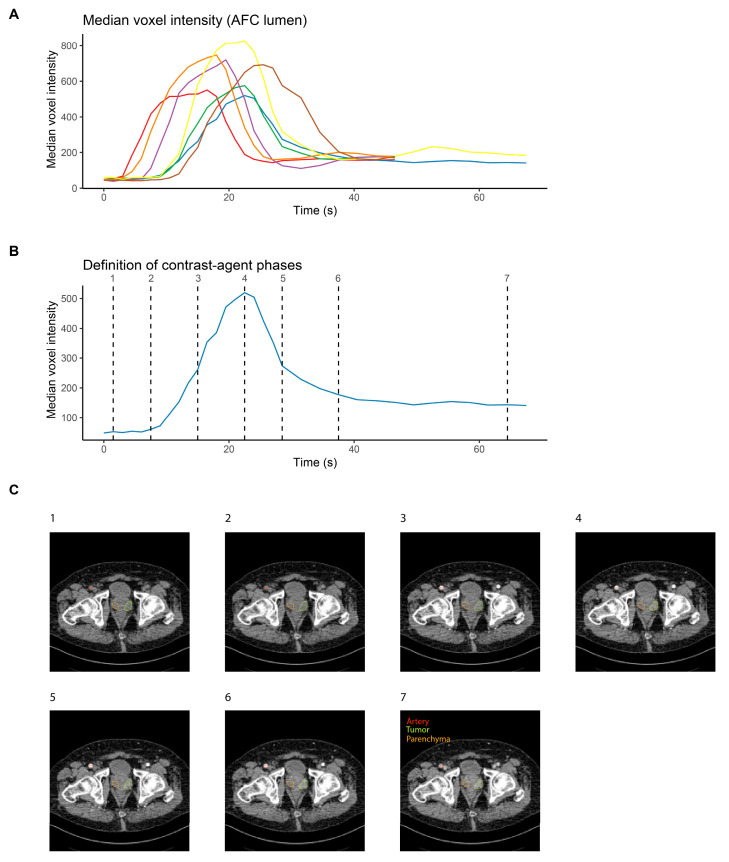
Synchronization of CT image series across prostate cancer patients. (**A**) Median voxel intensity as a function of time for each of the seven pzPC patients who were imaged with dceCT after intravenous injection of contrast medium. Lines are colored according to patients. (**B**) Definition of seven contrast agent phases on a single patient, to enable synchronization of image series across patients. (**C**) Representative slices for the seven defined contrast agent phases of a single prostate cancer patient.

Arterial lumen voxel median intensity values showed a high variability between patients throughout the acquisition phase (Figure 1A), with peaks occurring between 15 and 26 s and peak values between 520 and 827 HU. Taking fixed timepoints for radiomics evaluation (and thus simulating a standard CT protocol with a limited number of scans) led to a high variability of radiomics features in both tumors and healthy tissue (Figures S1 and S2). 

To enable the identification of radiomics features that depended on the contrast agent phase across patients, arterial median intensity values were used to define seven distinct contrast agent phases. Timepoint (TP) 1 was defined as the second acquired scan, TP2 as the scan closest to an increase of at least 15% in median voxel intensity in the arterial lumen t compared to the mean of the first two scans. TP4 was the scan with peak voxel intensity, TP3 was the scan closest to the center between TP2 and TP4. Similarly, TP6 was defined as the last scan with a median voxel intensity at least 15% higher than the mean of the last two acquired scans, TP5 was the scan closest to the center between TP4 and TP6, and TP7 was the second-to-last acquired scan. Contrast agent phase definition according to the arterial lumen median intensity values for a representative patient are shown in Figure 1B, and representative slices for a single patient and for all seven contrast agent phases are shown in Figure 1C.

### 3.2. Contrast Agent Injection Induces Changes in CT Radiomics Features in Peripheral Zone Prostate Cancer and Healthy Prostate Parenchyma

**Figure 2:**
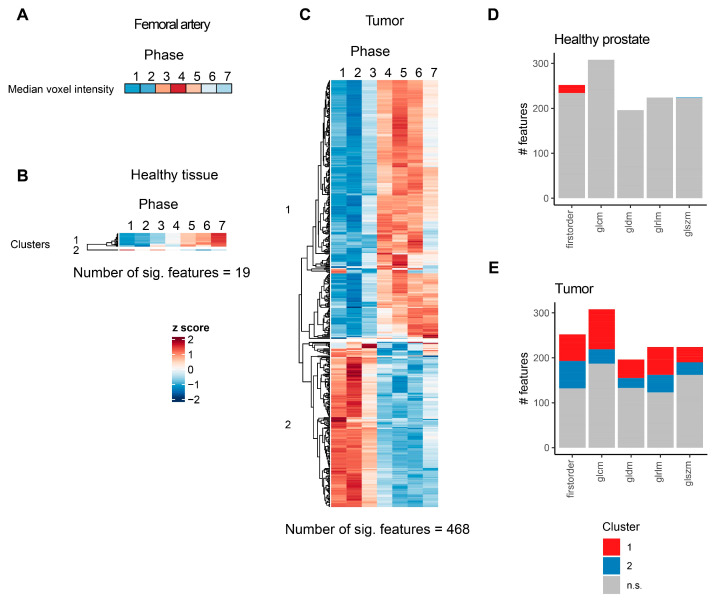
Significantly affected CT radiomics features in healthy prostate tissue and pzPC. (**A**) Heatmap of arterial lumen median voxel intensity. (**B**,**C**) Heatmaps of radiomics features with significant temporal dynamics after intravenous contrast agent injection for healthy prostate parenchyma (**B**) and pzPC (**C**) sections. For each feature, mean values for all seven patients were z-score transformed over the seven timepoints. Features are grouped according to hierarchical clustering, which is represented by dendrograms. (**D**,**E**) Number of radiomics features with and without significant temporal dynamics, grouped by feature class and colored according to clusters as defined in (**B**,**C**). glcm: gray level co-occurrence matrix, gldm: gray level dependence matrix, glrlm: gray level run length matrix, glszm: gray level size zone matrix.

Having defined contrast agent phases across patients, the next aim was to identify radiomics features that depended on contrast agent phase; i.e., showed significant temporal changes after contrast agent application. Using linear mixed models to determine feature values according to timepoints after injection, 19 (1.6%) and 467 (38.8%) of the 1204 features that showed significant (FDR < 0.05) temporal dynamics in healthy prostate parenchyma and prostate tumors, respectively, were identified (Figure 2B,C). The z-score-transformed arterial lumen median voxel intensities for each timepoint are shown as a reference (Figure 2A). Using hierarchical clustering, two diverging clusters of radiomics features could be identified for both healthy prostate parenchyma and prostate tumors. Linear mixed model *p* values and cluster membership of all features for both healthy prostate parenchyma and prostate tumors can be found in Supplementary Tables S1 and S2, respectively. 

For healthy prostate parenchyma, 18 of the 19 significant features belonged to the first cluster, which displayed low values initially and a monotonous increase after contrast agent injection. All these 18 features belonged to the first order class of radiomics features (Figure 2D, Supplementary Table S1). The only feature belonging to the second cluster, which showed high values initially and subsequently declined after contrast agent injection, was the feature wavelet.LLL_glszm_GrayLevelNonUniformityNormalized. On the other hand, in prostate tumors, more than one-third of all features showed significant temporal dynamics. Of note, while the strongest effect sizes and lowest *p* values were observed for first-order features, contrast media led to significant alterations in features that belonged to all radiomics feature classes (Figure 2E, Supplementary Table S2).

### 3.3. Machine-Learning-Based Classification of Healthy and Tumor ROIs Depends on Contrast Agent Phase

**Figure 3:**
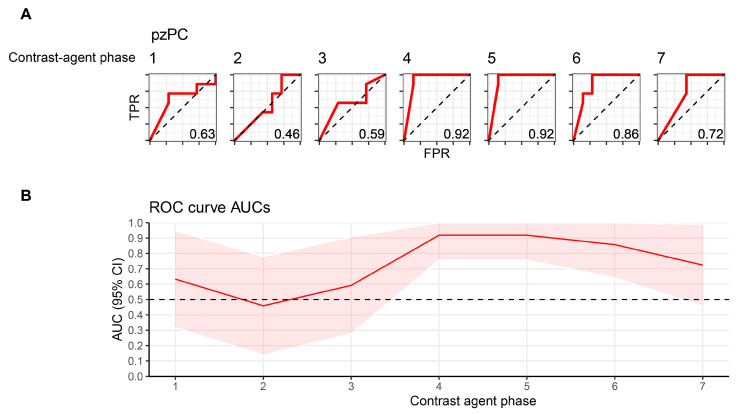
Machine-learning-based classification of healthy and pzPC ROIs depends on contrast agent phase. (**A**) ROC curves for logistic regression classifiers with an L2 penalty differentiating between healthy and tumor ROIs. Numbering above the curves denotes the timepoint after contrast agent injection. (**B**) AUC and 95% confidence intervals for each timepoint, which is represented on the *x*-axis. ROI, region-of-interest; pzPC, peripheral zone prostate cancer; TPR, true-positive rate; FPR, false-positive rate.

The next aim was to evaluate whether the ability of machine-learning classifiers to discriminate between healthy prostate tissue and prostate tumors is dependent on contrast agent phase. To this end, penalized logistic regression classifiers were trained on radiomics features to differentiate between healthy and tumor ROIs, and evaluated predictive accuracy using leave-one-out cross validation. Classification accuracy showed a high dependence on contrast agent phase (Figure 3A,B). As expected, classifiers could not differentiate well between healthy prostate tissue and tumors early after contrast agent application, with AUROC values ranging from 0.46 to 0.59 for the first three timepoints. In contrast, the highest predictive accuracies (AUROC 0.92) were found on TP4 (during peak arterial contrast enhancement) and TP5. Classification accuracy subsequently declined, but remained higher than during the initial phase after contrast agent injection (AUROC 0.86 and 0.72 for TP6 and TP7, respectively).

### 3.4. CT Radiomics Features Show Distinct Contrast Agent Dynamics in Lung Cancer

**Figure 4:**
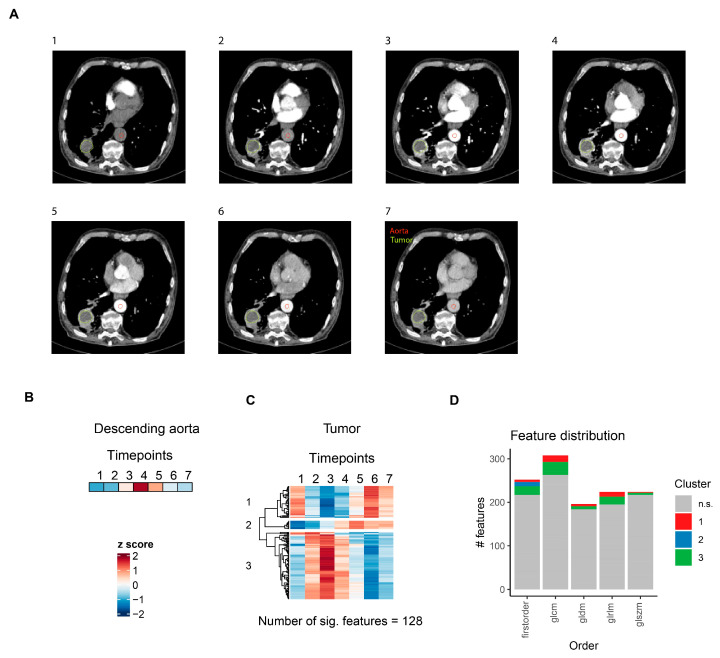
CT radiomics feature dynamics in lung cancer. (**A**) Representative slices for the seven defined contrast agent phases of a single lung cancer patient. (**B**) Heatmap of aortal lumen median voxel intensity. (**C**) Heatmap of radiomics features with significant temporal dynamics after intravenous contrast agent injection in solid lung tumors. For each feature, mean values for all seven patients are z-score transformed over the seven timepoints. Features are grouped by hierarchical clustering. (**D**) Distribution of radiomics features across different feature classes. Features are colored according to clusters defined in (**C**). glcm: gray level co-occurrence matrix, gldm: gray level dependence matrix, glrlm: gray level run length matrix, glszm: gray level size zone matrix.

To determine how contrast agent application affected radiomics features in NSCLC, a similar methodology was applied, but the seven contrast agent phases were defined based on the descending aortal lumen median voxel intensity. Representative slices for a single patient are shown in Figure 4A. A total of 128 (10.6%) features with significant temporal dynamics after contrast agent application were identified, which, unlike in prostate tumors, could be grouped into three different clusters (Figure 4C, Supplementary Table S3). As in Figure 3, z-score transformed aortal lumen median voxel intensities for each timepoint are shown as a reference (Figure 4B). The first cluster consisted of features belonging to all radiomics feature classes, all of which were extracted from images after wavelet filter transformations (Supplementary Table S3). The second cluster, which gradually increased after contrast agent administration—and thus most likely reflected contrast agent influx into the tumor—was the smallest cluster in terms of the number of significant features. However, it included the features with the strongest effect sizes and lowest *p* values (Supplementary Table S3) and consisted exclusively of first-order features (Figure 4D). The third cluster, to which the highest number of features with significant temporal dynamics belonged, included radiomics features of all classes (Figure 4D), most of which were also extracted from images after wavelet filter transformations (Supplementary Table S3).

### 3.5. Contrast Agent Application Induces Drastic Changes in MRI Radiomics Features in pzPC and NSCLC Tumors

**Figure 5:**
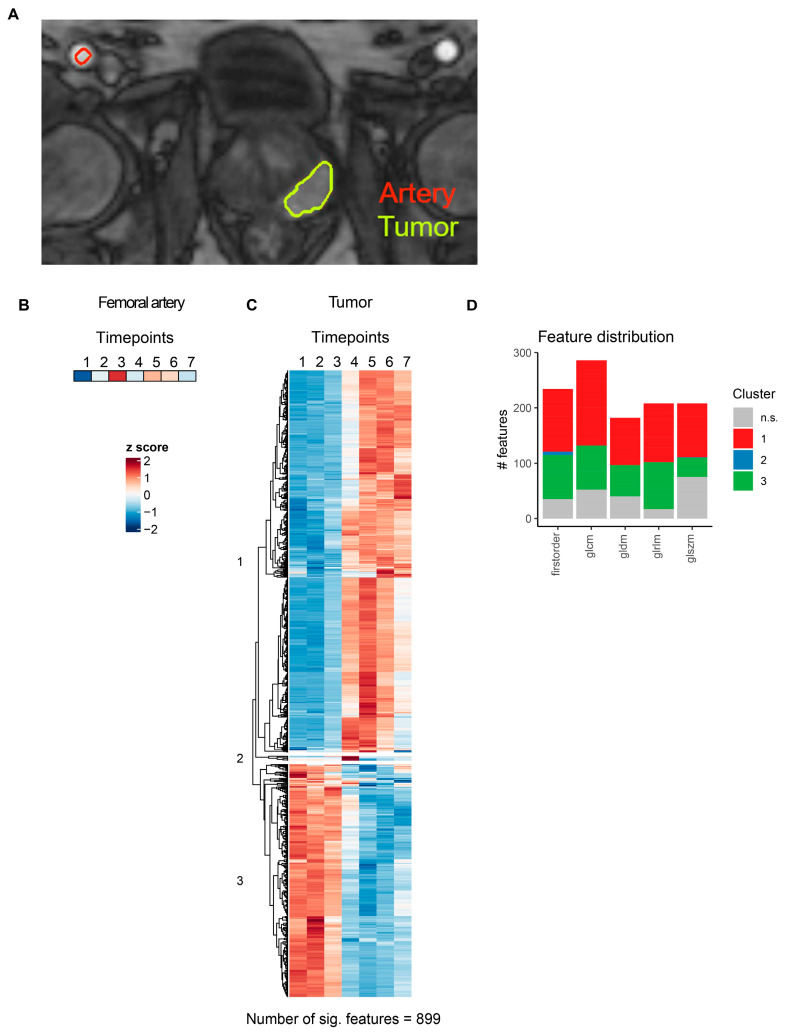
MRI radiomics feature dynamics in prostate cancer. (**A**) Representative slice illustrating segmentation of femoral artery and pzPC lesions in a single pzPC patient. (**B**) Heatmap of the femoral artery lumen median voxel intensity. (**C**) Heatmap of radiomics features with significant temporal dynamics after intravenous contrast agent injection in pzPC lesions. Features are grouped by hierarchical clustering. (**D**) Distribution of radiomics features across different feature classes. Features are colored according to clusters defined in (**C**). glcm: gray level co-occurrence matrix, gldm: gray level dependence matrix, glrlm: gray level run length matrix, glszm: gray level size zone matrix.

**Figure 6:**
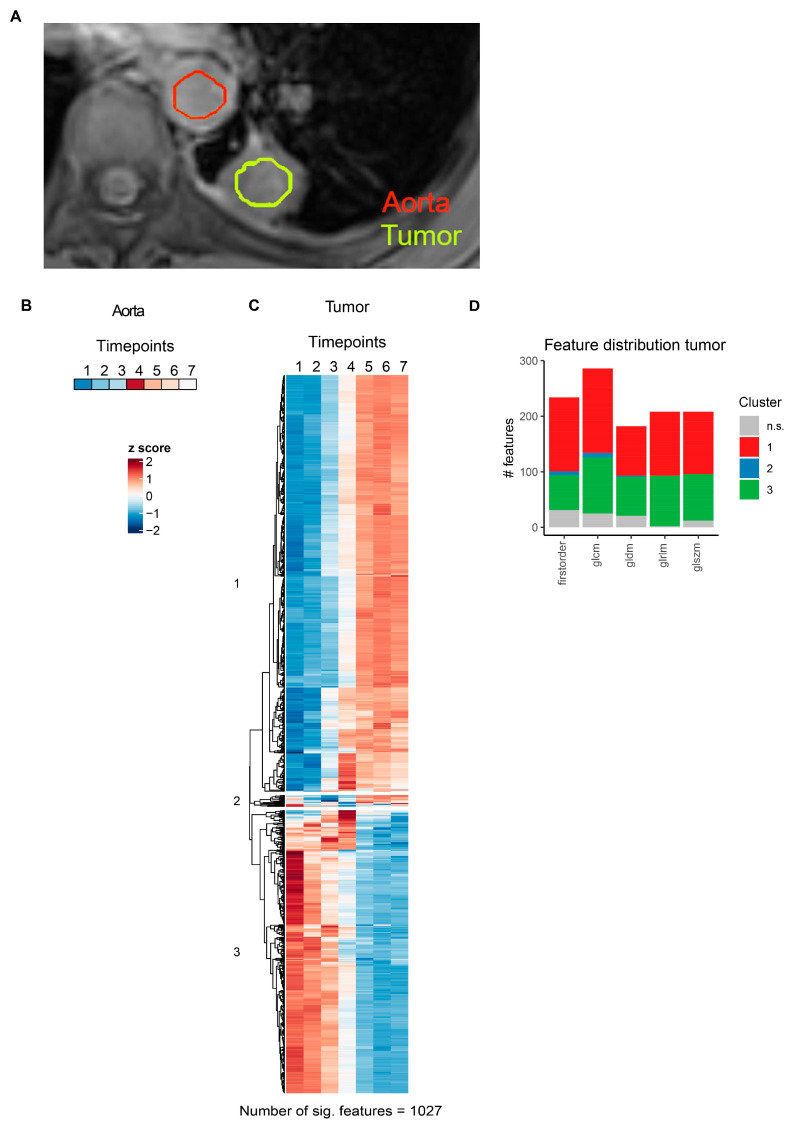
MRI radiomics feature dynamics in lung cancer. (**A**) Representative slice illustrating segmentation of thoracic aorta and NSCLC lesions in a single NSCLC patient. (**B**) Heatmap of aortal lumen median voxel intensity. (**C**) Heatmap of radiomics features with significant temporal dynamics after intravenous contrast agent injection in solid lung tumors. Features are grouped by hierarchical clustering. (**D**) Distribution of radiomics features across different feature classes. Features are colored according to clusters defined in (**C**). glcm: gray level co-occurrence matrix, gldm: gray level dependence matrix, glrlm: gray level run length matrix, glszm: gray level size zone matrix.

Next, the effects of contrast agent administration on the MRI radiomics profiles of pzPC or NSCLC tumors were examined. Tumors were segmented on T1-weighted sequences (Figure 5A and Figure 6A), and contrast agent phases were manually identified based on signal intensities in the femoral artery (for prostate MR images) or the aortal (for lung MR images) lumen. Z-score-transformed arterial or aortal lumen median voxel intensities for each timepoint are shown in Figure 5B and Figure 6B.

For pzPC tumors, 899 of 1118 (80.4%) MRI radiomics features showed significant temporal dynamics after multiple testing correction (Figure 5C, Supplementary Table S4). Hierarchical clustering grouped these features into three distinct clusters (Figure 5C,D). Similarly, for NSCLC tumors, 1027 of 1118 (91.9%) MRI radiomics features were significantly associated with time after contrast agent injection (Figure 6C, Supplementary Table S5). As in pzPC tumors, these features could be grouped into three clusters via hierarchical clustering (Figure 6C,D).

## 4. Discussion

The data presented in this study show that radiomics profiles of pZC and NSCLC are highly dependent on the contrast agent phase. Data from patients with pzPC and NSCLC with dynamic contrast-enhanced computed tomography (dceCT) were analyzed. These two cancer types were selected due to their contrary contrast agent uptake characteristics: PzC is characterized by rapid, intense contrast agent uptake compared to the surrounding non-tumoral peripheral zone tissue. This enables the visualization of pzPC using dynamic imaging techniques such as dynamic contrast-enhanced magnetic resonance imaging (dceMRI) [19] or dceCT [15]. In contrast, NSCLC does show a more heterogonous and weaker contrast agent uptake compared to pzPC, and is characterized by a longer contrast agent plateau phase with a slower in- and out-flow of the contrast agent [16]. Based on this difference in vascularity, it was hypothesized that contrast agent timing would have a more pronounced effect on radiomics features in patients with pzPC compared to NSCLC. 

The use of dynamic scanning protocols, in which multiple scans are acquired at short (1.5–3 s) pre-defined intervals after contrast agent injection, enabled us to examine the potential effects of contrast agents on radiomics features at an unprecedented temporal resolution. These data provide insights into the impact of contrast agent timing on the stability of radiomics features extracted from pzPC and NSCLC tumors.

Several other studies have analyzed the robustness of MRI- and CT-based radiomics features. These include phantom studies [9,20], test–retest studies [21,22], and reports on reproducibility and repeatability in oncological settings [23,24]. However, alterations in radiomics features in dceCT imaging have not been investigated systematically. In contrast to noise observed in test–retest variability, radiomics feature changes during dceCT time series may reflect the characteristics of the tumor. Hence, in this paper, important considerations for the analysis of radiomics features for contrast agent-enhanced CT and MRI data sets are described. 

First, this study shows that CT and MRI radiomics profiles of tumor ROIs in pzPC and NSCLC are dependent on contrast perfusion timing. There were 468 (38.8%) CT radiomics features that showed contrast agent timing-dependent alterations in pzPC, highlighting the effect of contrast agent timing on radiomics feature profiles. In contrast to the highly perfused pzPC, CT radiomics changes in NSCLC were less pronounced with 128 (10.6%) of features showing time-dependent changes. On MRI, radiomics features were considerably contrast agent-dependent in both pzPC and NSCLC patients. These changes are important for the further analysis of the features, as well as for the correlation of radiomics features with clinical outcomes and need to be dealt with in any quantitative imaging study involving the use of contrast agents. While heterogeneity-inducing scanning parameters (such as kVp, slice thickness, reconstruction kernel) and post-processing parameters (such as the reconstruction bins) can be standardized, harmonization of contrast agent timing is more challenging. This is particularly important for large-scale, multi-center studies with heterogeneous contrast agent timing protocols. The use of bolus tracking systems that start the scan once a certain amount of contrast attenuation reaches a tracking vessel improves the comparability between patients compared to fixed delay protocols. However, these bolus tracking protocols face problems in patients who suffer from low cardiac output [12], or who are unable to perform the examination with the planned i.v. injection flow rate, or in elderly patients [11]. Awareness of this potential problem when dealing with contrast agent enhanced datasets is needed. Simple quantitative measurements in reference vessels visible on all study participants (i.e., aorta, femoral vessel) could provide an overview of the data heterogeneity in terms of contrast distribution, and could be used to adequately implement time-scanning protocols, enabling standardization of contrast agent phases across patients and exclusion of obvious outliers. Alternatively, post hoc temporal alignment of contrast agent phases based on voxel intensity in time series with high temporal resolution may establish sufficient temporal correspondence among radiomics profiles, if contrast perfusion data sets are available. 

A possible solution to address the variability introduced by contrast agent dynamics is to utilize non-contrast scans whenever feasible and diagnostically meaningful. This approach would mitigate the bias introduced by contrast agent dynamics and could enhance the reproducibility of radiomics results.

Furthermore, this study underscores the importance of addressing contrast agent protocols. For multi-center studies, standardized contrast agent protocols are essential. These standardization efforts may involve recommendations for uniform timing, dosage and the specific types of contrast agents utilized. Nevertheless, even after controlling and harmonizing all these parameters, intra-patient differences in cardiac output will still significantly impact contrast dynamics

The data on machine-learning-based classification of healthy and tumorous prostate ROIs proves the relevance of contrast agent-induced changes in radiomics features for further analysis. Given that, even for a relatively simple classification task (i.e., differentiation between healthy and tumor ROIs), accuracy was greatly dependent on contrast agent phase, vast implications for more sophisticated approaches (e.g., prediction of survival or therapeutic response based on radiomics data) can be expected. 

Further advantages of this study include the high temporal resolution at which dceCT or dceMRI scans were performed, which gives unprecedented insights into the dynamics of radiomics features after contrast agent application, the robust statistical analysis, which was performed using linear mixed models. 

The main limitation of this study was the small size of the cohorts and that only two cancer types were investigated. However, this was sufficient to perform basic analysis due to the large number of effects observed for multiple radiomics features, but associations of even more radiomics features with contrast agent phases with a larger cohort size could be expected. A further limitation is the retrospective, single-center study design. Nevertheless, the presented data clearly show the influence of agent dynamics on radiomics features, and a prospective study design would likely yield similar results. Finally, the NSCLC included both adenocarcinomas and squamous cell carcinomas, and this heterogeneity may have influenced the results to a certain degree. This can, however, also be interpreted as a strength, since it suggests that the presented findings are generalizable across different NSCLC subtypes.

The presented data open up multiple avenues for further research. Future studies may explore the influence of contrast agent timing on radiomics features in other types of cancers, such as breast or ovarian cancer, in which radiomics have shown potential [25,26,27]. Additionally, radiomics feature dynamics after contrast agent application may harbor biological information, which might be useful to infer quantifiable data on tumor heterogeneity and predict outcome. Radiomics features are quantitative measurements extracted from CT or MRI scans, capturing various aspects of tumor morphology, texture and intensity. In lung or prostate cancer, certain features may reveal unique patterns in contrast agent dynamics, reflecting characteristics such as vascularity and perfusion within the tumor microenvironment. Identifying these features can provide insights into tumor biology, aiding in diagnosis, prognosis and treatment planning by differentiating between subtypes, assessing treatment response and predicting outcomes. Overall, these features might offer a non-invasive method to understand tumor behavior and personalize patient management.

## 5. Conclusions

This study shows that CT and MRI radiomics profiles of pZC and NSCLC are highly dependent on the contrast agent dynamics. This highlights the need for careful design of radiomics studies when contrast media are involved, as a substantial number of radiomics features are dependent on individual contrast perfusion dynamics and are therefore prone to bias. When using contrast-enhanced scans for radiomics studies, care should be taken to ensure a patient-specific perfusion timing for radiomics analysis, which could be achieved by a test bolus application.

## Figures and Tables

**Table 1:** Prostate cancer patient cohort.

ID	Age (Years)	Tumor Location	PI-RADS	Gleason Score	Number of CT Scans	Number of MRI Scans
1	78	Left peripheral zone	5	3 + 4	26	70
2	75	Left peripheral zone	4	3 + 3	33	70
3	64	Left peripheral zone	4	3 + 4	26	21
4	57	Right peripheral zone	4	3 + 3	26	No MRI included
5	76	Left peripheral zone	4	3 + 4	33	70
6	71	Left peripheral zone	4	4 + 3	26	No MRI included
7	57	Left peripheral zone	4	3 + 3	26	No MRI included
8	76	Right peripheral zone	4	3 + 4	No CT included	70
9	63	Left peripheral zone	4	3 + 3	No CT included	70
10	84	Left peripheral zone	4	3 + 3	No CT included	70
11	58	Right peripheral zone	4	3 + 3	No CT included	70
12	63	Right peripheral zone	4	4 + 3	No CT included	70

**Table 2:** CT lung cancer patient cohort.

ID	Age (Years)	Sex	Histology	Target Lesion	Stage	Number of CT Scans
1	70	Male	Squamous cell carcinoma	Primary tumor	IV	20
2	77	Male	Squamous cell carcinoma	Primary tumor	IV	30
3	74	Female	Adenocarcinoma	Primary tumor	IV	20
4	61	Male	Squamous cell carcinoma	Lymph node metastasis	IV	30
5	60	Male	Adenocarcinoma	Lymph node metastasis	IV	20
6	67	Male	Squamous cell carcinoma	Primary tumor	III	30
7	81	Male	Squamous cell carcinoma	Lymph node metastasis	IV	30

**Table 3:** MRI lung cancer patient cohort.

ID	Age (Years)	Sex	Histology	Target Lesion	Stage	Number of Visits	Number of Scans
1	55	Male	Squamous cell carcinoma	Primary tumor	IV	5	60–78 per visit
2	76	Male	Squamous cell carcinoma	Primary tumor	IV	2	76 per visit
3	43	Female	Adenocarcinoma	Primary tumor	IV	2	76 per visit
4	76	Male	Adenocarcinoma	Primary tumor	IV	2	76 per visit
5	66	Female	Squamous cell carcinoma	Primary tumor	IV	1	76
6	64	Male	Squamous cell carcinoma	Primary tumor	IV	2	74–76 per visit
7	70	Male	Adenocarcinoma	Primary tumor	IV	1	76
8	55	Female	Squamous cell carcinoma	Primary tumor	IV	2	76–78 per visit
9	67	Male	Squamous cell carcinoma	Primary tumor	IV	1	16
10	54	Male	Squamous cell carcinoma	Primary tumor	IV	1	76
11	74	Male	Adenocarcinoma	Primary tumor	IV	3	70–76 per visit
12	56	Male	Adenocarcinoma	Primary tumor	IV	2	76 per visit
13	69	Female	Adenocarcinoma	Primary tumor	IV	2	76 per visit
14	52	Female	Squamous cell carcinoma	Primary tumor	IV	3	76 per visit
15	81	Female	Adenocarcinoma	Primary tumor	IV	1	76
16	54	Male	Adenocarcinoma	Primary tumor	IV	1	76
17	64	Male	Squamous cell carcinoma	Primary tumor	IV	1	76
18	68	Female	Squamous cell carcinoma	Primary tumor	IV	1	76
19	50	Female	Adenocarcinoma	Primary tumor	IV	1	70
20	74	Male	Adenocarcinoma	Primary tumor	IV	1	76
21	60	Female	Squamous cell carcinoma	Primary tumor	IV	1	76

## Data Availability

Radiomics data and analysis scripts are available upon request.

## Supplementary Materials

The following supporting information can be downloaded at: https://www.mdpi.com/article/10.3390/cancers16081519/s1, Figure S1: Perfusion dynamics of selected CT radiomics features in prostate tumors; Figure S2: Perfusion dynamics of selected CT radiomics features in in healthy prostate tissue; Table S1: Linear mixed model *p* values and cluster membership of all CT radiomics features for prostate parenchyma; Table S2: Linear mixed model *p* values and cluster membership of all CT radiomics features for prostate tumors; Table S3: Linear mixed model *p* values and cluster membership of all CT radiomics features for lung tumors; Table S4: Linear mixed model *p* values and cluster membership of all MRI radiomics features for pzPC tumors; Table S5: Linear mixed model *p* values and cluster membership of all MRI radiomics features for NSCLC tumors.

## Abbreviations

CT	Computed tomography
dceCT	Dynamic contrast-enhanced CT
dceMRI	Dynamic contrast-enhanced MRI
FDR	False discovery rate
GLCM	Gray level co-occurrence matrix
GLDM	Gray level dependence matrix
GLRLM	Gray level run length matrix
GLSZM	Gray level size zone matrix
i.v.	Intravenous
kVp	Kilovolt pascal
LoG	Laplacian of Gaussian
mL	Mililiter
MRI	Magnetic resonance imaging
NSCLC	Non-small cell lung cancer
PI-RADS	Prostate Imaging-Reporting and Data System
pzPC	Peripheral zone prostate cancer
ROC	Receiver-operator-characteristics
ROI	Region-of-interest
