# Supplementary material for: Contrast Agent Dynamics Determine Radiomics Profiles in Oncologic Imaging
Source: Cancers (Basel). 2024 Apr 16;16(8):1519. doi: 10.3390/cancers16081519 (PMC11049400; doi:10.3390/cancers16081519)
Supplement: Supplementary file 1 [file cancers-16-01519-s001.zip › Table S1.pdf]

Table S1: Linear mixed model p values and cluster membership of all CT radiomics features for prostate parenchyma

Feature	F value	p value	FDR	Cluster
wavelet.LLL_firstorder_Mean	25,88065	1,13E-11	3,4E-09	1
wavelet.LLL_firstorder_RootMeanSquared	25,87682	1,13E-11	3,4E-09	1
original_firstorder_Mean	25,89841	1,11E-11	3,4E-09	1
original_firstorder_RootMeanSquared	25,87986	1,13E-11	3,4E-09	1
original_firstorder_Energy	9,792666	8,05E-11	1,38E-08	1
original_firstorder_Median	23,04962	5,75E-11	1,38E-08	1
original_firstorder_TotalEnergy	9,792666	8,05E-11	1,38E-08	1
wavelet.LLL_firstorder_Energy	9,662352	1,16E-10	1,55E-08	1
wavelet.LLL_firstorder_TotalEnergy	9,662352	1,16E-10	1,55E-08	1
wavelet.LLL_firstorder_Median	20,16553	3,58E-10	4,31E-08	1
wavelet.LLL_firstorder_90Percentile	17,41994	2,46E-09	2,7E-07	1
original_firstorder_90Percentile	17,11097	3,1E-09	3,11E-07	1
wavelet.LLL_firstorder_10Percentile	14,73152	2,03E-08	1,75E-06	1
original_firstorder_10Percentile	14,773	1,96E-08	1,75E-06	1
wavelet.LLL_firstorder_Maximum	12,93112	9,69E-08	7,78E-06	1
original_firstorder_Maximum	9,275476	3,71E-06	0,000279	1
wavelet.HLH_firstorder_InterquartileRange	7,483071	2,98E-05	0,002111	1
wavelet.LLL_glszm_GrayLevelNonUniformityNormalized	5,673845	0,000314	0,021011	2
wavelet.HLH_firstorder_RobustMeanAbsoluteDeviation	5,209451	0,000602	0,038163	1
log.sigma.4.0.mm.3D_gldm_LargeDependenceEmphasis	4,936225	0,000892	0,051121	n.s.
log.sigma.5.0.mm.3D_glrlm_RunLengthNonUniformityNormalized	4,939578	0,000887	0,051121	n.s.
log.sigma.4.0.mm.3D_glcm_SumSquares	4,689768	0,001278	0,069947	n.s.
log.sigma.4.0.mm.3D_glrlm_RunPercentage	4,586181	0,00149	0,077418	n.s.
log.sigma.4.0.mm.3D_glcm_Id	4,458872	0,0018	0,077418	n.s.
log.sigma.4.0.mm.3D_glcm_DifferenceAverage	4,458872	0,0018	0,077418	n.s.
log.sigma.4.0.mm.3D_glcm_InverseVariance	4,458872	0,0018	0,077418	n.s.
log.sigma.4.0.mm.3D_glcm_Contrast	4,458872	0,0018	0,077418	n.s.
log.sigma.4.0.mm.3D_glcm_Idm	4,458872	0,0018	0,077418	n.s.
log.sigma.4.0.mm.3D_glcm_ClusterTendency	4,373946	0,002045	0,084897	n.s.
log.sigma.4.0.mm.3D_gldm_DependenceEntropy	4,308149	0,002258	0,087694	n.s.
original_glszm_GrayLevelVariance	4,325923	0,002198	0,087694	n.s.
log.sigma.1.0.mm.3D_gldm_SmallDependenceHighGrayLevelEmphasis	4,251836	0,002459	0,092505	n.s.
log.sigma.4.0.mm.3D_glcm_DifferenceVariance	4,205982	0,002636	0,096164	n.s.
wavelet.HLH_firstorder_MeanAbsoluteDeviation	4,165317	0,002804	0,099292	n.s.
log.sigma.4.0.mm.3D_glcm_JointEntropy	4,130661	0,002956	0,101689	n.s.
log.sigma.4.0.mm.3D_glcm_JointEnergy	4,060907	0,003289	0,10519	n.s.
log.sigma.4.0.mm.3D_glcm_SumEntropy	4,071058	0,003238	0,10519	n.s.
log.sigma.4.0.mm.3D_gldm_GrayLevelVariance	4,054789	0,00332	0,10519	n.s.
wavelet.HLL_glrlm_GrayLevelNonUniformity	3,98586	0,003691	0,113404	n.s.
log.sigma.5.0.mm.3D_glrlm_RunPercentage	3,972527	0,003768	0,113404	n.s.
log.sigma.4.0.mm.3D_glcm_MaximumProbability	3,87829	0,004358	0,113557	n.s.

log.sigma.5.0.mm.3D_glcm_Id	3,867365	0,004433	0,113557	n.s.
log.sigma.5.0.mm.3D_glcm_DifferenceAverage	3,867365	0,004433	0,113557	n.s.
log.sigma.5.0.mm.3D_glcm_InverseVariance	3,867365	0,004433	0,113557	n.s.
log.sigma.5.0.mm.3D_glcm_Contrast	3,867365	0,004433	0,113557	n.s.
log.sigma.5.0.mm.3D_glcm_Idm	3,867365	0,004433	0,113557	n.s.
log.sigma.5.0.mm.3D_gldm_LargeDependenceEmphasis	3,949861	0,003902	0,113557	n.s.
log.sigma.1.0.mm.3D_glcm_JointAverage	3,81782	0,004787	0,113702	n.s.
log.sigma.1.0.mm.3D_glcm_Autocorrelation	3,818267	0,004784	0,113702	n.s.
log.sigma.1.0.mm.3D_gldm_HighGrayLevelEmphasis	3,813968	0,004816	0,113702	n.s.
log.sigma.4.0.mm.3D_glcm_DifferenceEntropy	3,820403	0,004768	0,113702	n.s.
wavelet.LLL_firstorder_Range	3,786235	0,005029	0,116438	n.s.
log.sigma.1.0.mm.3D_glrlm_HighGrayLevelRunEmphasis	3,696231	0,005788	0,131495	n.s.
log.sigma.4.0.mm.3D_glrlm_LongRunEmphasis	3,672535	0,006008	0,133945	n.s.
log.sigma.1.0.mm.3D_glrlm_LongRunHighGrayLevelEmphasis	3,647424	0,006249	0,134356	n.s.
log.sigma.5.0.mm.3D_glcm_DifferenceVariance	3,656897	0,006157	0,134356	n.s.
log.sigma.1.0.mm.3D_firstorder_Minimum	3,619864	0,006526	0,137845	n.s.
log.sigma.5.0.mm.3D_glrlm_ShortRunEmphasis	3,596939	0,006766	0,140449	n.s.
wavelet.HLL_gldm_GrayLevelNonUniformity	3,552466	0,007258	0,148104	n.s.
log.sigma.4.0.mm.3D_glcm_Idn	3,503642	0,00784	0,15733	n.s.
log.sigma.1.0.mm.3D_glrlm_ShortRunHighGrayLevelEmphasis	3,48899	0,008025	0,157359	n.s.
log.sigma.1.0.mm.3D_glszm_SmallAreaHighGrayLevelEmphasis	3,474763	0,008208	0,157359	n.s.
log.sigma.4.0.mm.3D_firstorder_Uniformity	3,47275	0,008234	0,157359	n.s.
log.sigma.1.0.mm.3D_gldm_LargeDependenceHighGrayLevelEmphasis	3,44166	0,008651	0,16274	n.s.
wavelet.HLL_glcm_SumEntropy	3,306664	0,010729	0,187304	n.s.
log.sigma.4.0.mm.3D_firstorder_Entropy	3,333523	0,010278	0,187304	n.s.
log.sigma.5.0.mm.3D_glcm_DifferenceEntropy	3,31108	0,010653	0,187304	n.s.
original_firstorder_Range	3,306364	0,010734	0,187304	n.s.
original_glszm_GrayLevelNonUniformityNormalized	3,315033	0,010586	0,187304	n.s.
log.sigma.5.0.mm.3D_glrlm_RunLengthNonUniformity	3,250263	0,011744	0,202001	n.s.
wavelet.LLH_gldm_DependenceVariance	3,217032	0,012388	0,210076	n.s.
wavelet.HLL_glcm_JointEntropy	3,194107	0,012854	0,214941	n.s.
log.sigma.4.0.mm.3D_glrlm_RunLengthNonUniformity	3,17184	0,013323	0,216903	n.s.
log.sigma.5.0.mm.3D_firstorder_Range	3,171452	0,013331	0,216903	n.s.
wavelet.HLL_glcm_ClusterTendency	3,14785	0,013848	0,222312	n.s.
wavelet.HLH_firstorder_90Percentile	3,128591	0,014286	0,226313	n.s.
wavelet.LLL_glrlm_RunLengthNonUniformity	3,055497	0,016078	0,251405	n.s.
wavelet.HLL_firstorder_10Percentile	2,760809	0,025992	0,284181	n.s.
wavelet.HLL_firstorder_Uniformity	2,893837	0,020911	0,284181	n.s.
wavelet.HLL_firstorder_Entropy	2,857047	0,022205	0,284181	n.s.
wavelet.HLL_glrlm_GrayLevelVariance	2,756606	0,026171	0,284181	n.s.
wavelet.HLL_glrlm_GrayLevelNonUniformityNormalized	2,755764	0,026207	0,284181	n.s.
wavelet.HLL_glcm_JointEnergy	2,885926	0,021182	0,284181	n.s.
wavelet.HLL_glcm_DifferenceEntropy	2,865818	0,021889	0,284181	n.s.
wavelet.HLL_glcm_MaximumProbability	2,757697	0,026124	0,284181	n.s.
wavelet.HLL_glcm_SumSquares	2,90321	0,020594	0,284181	n.s.
wavelet.HLL_gldm_GrayLevelVariance	2,850029	0,022461	0,284181	n.s.

wavelet.HLH_firstorder_Variance	2,84701	0,022572	0,284181	n.s.
wavelet.HLH_glrlm_LongRunEmphasis	2,77881	0,025236	0,284181	n.s.
wavelet.HLH_glszm_SmallAreaHighGrayLevelEmphasis	2,850567	0,022441	0,284181	n.s.
wavelet.HHH_firstorder_10Percentile	2,803658	0,02423	0,284181	n.s.
wavelet.HHH_firstorder_90Percentile	2,739684	0,026908	0,284181	n.s.
wavelet.LLL_glszm_GrayLevelVariance	2,748519	0,026521	0,284181	n.s.
wavelet.LLL_glszm_SmallAreaHighGrayLevelEmphasis	2,910301	0,020357	0,284181	n.s.
wavelet.LLL_gldm_SmallDependenceEmphasis	2,951959	0,019022	0,284181	n.s.
log.sigma.1.0.mm.3D_firstorder_Median	2,95246	0,019006	0,284181	n.s.
log.sigma.1.0.mm.3D_firstorder_Range	2,792134	0,024692	0,284181	n.s.
log.sigma.1.0.mm.3D_glrlm_LongRunLowGrayLevelEmphasis	2,94359	0,019282	0,284181	n.s.
log.sigma.1.0.mm.3D_glszm_SizeZoneNonUniformity	2,744188	0,026709	0,284181	n.s.
log.sigma.1.0.mm.3D_glszm_LargeAreaLowGrayLevelEmphasis	2,84768	0,022547	0,284181	n.s.
log.sigma.1.0.mm.3D_gldm_SmallDependenceEmphasis	2,744906	0,026678	0,284181	n.s.
log.sigma.2.0.mm.3D_firstorder_Maximum	2,765667	0,025785	0,284181	n.s.
log.sigma.4.0.mm.3D_glrlm_RunVariance	2,836853	0,02295	0,284181	n.s.
log.sigma.4.0.mm.3D_glrlm_ShortRunHighGrayLevelEmphasis	2,74866	0,026514	0,284181	n.s.
log.sigma.4.0.mm.3D_glcm_Imc2	2,764713	0,025826	0,284181	n.s.
log.sigma.4.0.mm.3D_gldm_DependenceNonUniformityNormalized	2,902776	0,020608	0,284181	n.s.
log.sigma.5.0.mm.3D_glrlm_ShortRunHighGrayLevelEmphasis	2,867676	0,021823	0,284181	n.s.
log.sigma.5.0.mm.3D_glrlm_GrayLevelVariance	2,796085	0,024532	0,284181	n.s.
log.sigma.5.0.mm.3D_glcm_Idn	2,843063	0,022718	0,284181	n.s.
log.sigma.5.0.mm.3D_glcm_JointEntropy	2,908441	0,020419	0,284181	n.s.
log.sigma.5.0.mm.3D_glcm_SumEntropy	2,81319	0,023855	0,284181	n.s.
log.sigma.5.0.mm.3D_glcm_SumSquares	2,767398	0,025712	0,284181	n.s.
log.sigma.5.0.mm.3D_glszm_GrayLevelVariance	2,948715	0,019122	0,284181	n.s.
log.sigma.5.0.mm.3D_gldm_GrayLevelVariance	2,7604	0,026009	0,284181	n.s.
wavelet.LLL_glszm_HighGrayLevelZoneEmphasis	2,725526	0,02754	0,286431	n.s.
log.sigma.1.0.mm.3D_gldm_LargeDependenceLowGrayLevelEmphasis	2,724276	0,027596	0,286431	n.s.
wavelet.LLL_glrlm_LongRunEmphasis	2,712268	0,028146	0,28718	n.s.
log.sigma.2.0.mm.3D_glszm_SizeZoneNonUniformity	2,71229	0,028145	0,28718	n.s.
wavelet.HLL_firstorder_MeanAbsoluteDeviation	2,705428	0,028463	0,287983	n.s.
wavelet.HLL_glrlm_RunEntropy	2,694232	0,028992	0,290882	n.s.
wavelet.HLL_firstorder_Variance	2,665985	0,030369	0,294872	n.s.
wavelet.HLL_glszm_GrayLevelNonUniformityNormalized	2,679694	0,029692	0,294872	n.s.
log.sigma.1.0.mm.3D_glszm_HighGrayLevelZoneEmphasis	2,675832	0,029881	0,294872	n.s.
log.sigma.5.0.mm.3D_glszm_HighGrayLevelZoneEmphasis	2,668599	0,030239	0,294872	n.s.
wavelet.LLL_glrlm_RunVariance	2,648932	0,031232	0,300831	n.s.
wavelet.HLL_glcm_DifferenceVariance	2,634356	0,03199	0,305686	n.s.
log.sigma.5.0.mm.3D_firstorder_Entropy	2,614958	0,033028	0,313117	n.s.
wavelet.HLH_glrlm_RunVariance	2,591737	0,034316	0,32278	n.s.
wavelet.HLL_glrlm_RunVariance	2,553898	0,036523	0,334939	n.s.
wavelet.HLL_glcm_DifferenceAverage	2,544609	0,037087	0,334939	n.s.
log.sigma.4.0.mm.3D_glrlm_RunLengthNonUniformityNormalized	2,559612	0,036181	0,334939	n.s.
log.sigma.5.0.mm.3D_glrlm_GrayLevelNonUniformityNormalized	2,558774	0,036231	0,334939	n.s.
log.sigma.5.0.mm.3D_glcm_ClusterTendency	2,549493	0,03679	0,334939	n.s.

log.sigma.5.0.mm.3D_gldm_SmallDependenceLowGrayLevelEmphasis	2,541509	0,037277	0,334939	n.s.
log.sigma.5.0.mm.3D_gldm_DependenceEntropy	2,536378	0,037594	0,335283	n.s.
wavelet.LLH_gldm_DependenceNonUniformity	2,52196	0,038499	0,340828	n.s.
wavelet.LLL_gldm_SmallDependenceHighGrayLevelEmphasis	2,515159	0,038933	0,342159	n.s.
wavelet.HLL_firstorder_RobustMeanAbsoluteDeviation	2,505927	0,039531	0,344894	n.s.
log.sigma.5.0.mm.3D_glcm_JointEnergy	2,498144	0,040042	0,34684	n.s.
wavelet.LLL_glszm_SizeZoneNonUniformity	2,486301	0,040833	0,351086	n.s.
log.sigma.1.0.mm.3D_glszm_GrayLevelNonUniformityNormalized	2,48212	0,041116	0,351086	n.s.
wavelet.HLH_firstorder_10Percentile	2,473718	0,04169	0,353484	n.s.
wavelet.HLL_glcm_Contrast	2,468983	0,042017	0,353768	n.s.
wavelet.HHH_firstorder_RobustMeanAbsoluteDeviation	2,449872	0,043365	0,357946	n.s.
wavelet.LLL_glrlm_ShortRunHighGrayLevelEmphasis	2,452691	0,043163	0,357946	n.s.
log.sigma.3.0.mm.3D_firstorder_Maximum	2,449306	0,043405	0,357946	n.s.
wavelet.HLL_firstorder_90Percentile	2,388066	0,048029	0,359175	n.s.
wavelet.HLL_glrlm_RunLengthNonUniformity	2,433813	0,044531	0,359175	n.s.
wavelet.HLL_glcm_Id	2,390652	0,047824	0,359175	n.s.
wavelet.HLL_glcm_Idm	2,401655	0,046962	0,359175	n.s.
wavelet.HHH_firstorder_InterquartileRange	2,440907	0,044012	0,359175	n.s.
wavelet.HHH_firstorder_MeanAbsoluteDeviation	2,397753	0,047266	0,359175	n.s.
wavelet.LLL_glrlm_ShortRunEmphasis	2,412536	0,046125	0,359175	n.s.
wavelet.LLL_glrlm_HighGrayLevelRunEmphasis	2,394456	0,047524	0,359175	n.s.
wavelet.LLL_gldm_DependenceNonUniformity	2,399557	0,047125	0,359175	n.s.
log.sigma.1.0.mm.3D_glszm_SmallAreaEmphasis	2,411242	0,046224	0,359175	n.s.
log.sigma.1.0.mm.3D_gldm_GrayLevelNonUniformity	2,416003	0,045861	0,359175	n.s.
log.sigma.1.0.mm.3D_gldm_LowGrayLevelEmphasis	2,416635	0,045813	0,359175	n.s.
log.sigma.3.0.mm.3D_firstorder_Range	2,396819	0,047339	0,359175	n.s.
log.sigma.4.0.mm.3D_glcm_Idmn	2,397846	0,047259	0,359175	n.s.
log.sigma.5.0.mm.3D_glrlm_RunEntropy	2,433068	0,044586	0,359175	n.s.
wavelet.LHH_firstorder_Skewness	2,375611	0,049029	0,361978	n.s.
wavelet.LHH_firstorder_Median	2,329856	0,049498	0,361978	n.s.
wavelet.LLL_gldm_HighGrayLevelEmphasis	2,368526	0,049607	0,361978	n.s.
log.sigma.1.0.mm.3D_glrlm_LowGrayLevelRunEmphasis	2,372359	0,049293	0,361978	n.s.
log.sigma.3.0.mm.3D_glrlm_LongRunLowGrayLevelEmphasis	2,352229	0,050962	0,369629	n.s.
wavelet.HHL_glszm_LowGrayLevelZoneEmphasis	2,338892	0,052099	0,375612	n.s.
log.sigma.4.0.mm.3D_glrlm_GrayLevelVariance	2,331732	0,05272	0,377826	n.s.
wavelet.HLL_glszm_ZoneVariance	2,326973	0,053137	0,378514	n.s.
log.sigma.3.0.mm.3D_glcm_Imc2	2,32348	0,053445	0,378514	n.s.
wavelet.HLH_glszm_SmallAreaEmphasis	2,315225	0,05418	0,381476	n.s.
wavelet.LLL_glcm_Autocorrelation	2,309143	0,054728	0,383095	n.s.
wavelet.LHL_glrlm_RunLengthNonUniformity	2,286827	0,056787	0,388474	n.s.
wavelet.HLL_glszm_ZoneEntropy	2,291011	0,056395	0,388474	n.s.
log.sigma.1.0.mm.3D_glrlm_ShortRunLowGrayLevelEmphasis	2,294758	0,056046	0,388474	n.s.
log.sigma.4.0.mm.3D_glrlm_ShortRunEmphasis	2,289208	0,056563	0,388474	n.s.
wavelet.LHL_gldm_DependenceNonUniformity	2,270012	0,058389	0,392743	n.s.
wavelet.HLL_glrlm_LongRunEmphasis	2,275358	0,057875	0,392743	n.s.
wavelet.LLL_glrlm_RunPercentage	2,270138	0,058377	0,392743	n.s.

wavelet.HLL_glcm_ClusterProminence	2,219843	0,063445	0,393213	n.s.
wavelet.HLL_glszm_LargeAreaEmphasis	2,236068	0,061764	0,393213	n.s.
wavelet.HLH_firstorder_Kurtosis	2,238606	0,061505	0,393213	n.s.
wavelet.HLH_glszm_LowGrayLevelZoneEmphasis	2,265632	0,058814	0,393213	n.s.
wavelet.HHH_firstorder_Kurtosis	2,247702	0,060586	0,393213	n.s.
wavelet.LLL_glrlm_RunLengthNonUniformityNormalized	2,221262	0,063296	0,393213	n.s.
log.sigma.1.0.mm.3D_firstorder_90Percentile	2,219749	0,063455	0,393213	n.s.
log.sigma.1.0.mm.3D_glrlm_GrayLevelNonUniformity	2,217565	0,063685	0,393213	n.s.
log.sigma.2.0.mm.3D_glrlm_LongRunEmphasis	2,221081	0,063315	0,393213	n.s.
log.sigma.3.0.mm.3D_glszm_SmallAreaLowGrayLevelEmphasis	2,242618	0,061098	0,393213	n.s.
log.sigma.3.0.mm.3D_gldm_LargeDependenceLowGrayLevelEmphasis	2,231864	0,062195	0,393213	n.s.
log.sigma.4.0.mm.3D_gldm_GrayLevelNonUniformity	2,245492	0,060808	0,393213	n.s.
log.sigma.5.0.mm.3D_firstorder_Uniformity	2,227863	0,062609	0,393213	n.s.
original_firstorder_Minimum	2,220017	0,063427	0,393213	n.s.
original_glrlm_RunEntropy	2,261111	0,059256	0,393213	n.s.
original_gldm_GrayLevelNonUniformity	2,232815	0,062097	0,393213	n.s.
log.sigma.4.0.mm.3D_glrlm_LongRunLowGrayLevelEmphasis	2,209092	0,064584	0,396732	n.s.
log.sigma.4.0.mm.3D_glrlm_GrayLevelNonUniformityNormalized	2,201352	0,065417	0,399808	n.s.
log.sigma.5.0.mm.3D_glcm_MaximumProbability	2,194971	0,066112	0,399992	n.s.
log.sigma.5.0.mm.3D_glcm_Correlation	2,195333	0,066072	0,399992	n.s.
log.sigma.4.0.mm.3D_firstorder_Range	2,182678	0,06747	0,405606	n.s.
log.sigma.5.0.mm.3D_glcm_Imc2	2,180508	0,067713	0,405606	n.s.
original_glszm_GrayLevelNonUniformity	2,164161	0,06957	0,414666	n.s.
wavelet.HLH_glszm_HighGrayLevelZoneEmphasis	2,156074	0,070507	0,418182	n.s.
wavelet.HLH_firstorder_Mean	2,11543	0,071537	0,422211	n.s.
wavelet.HLL_glrlm_RunPercentage	2,133671	0,07317	0,427654	n.s.
wavelet.LLL_glcm_JointEntropy	2,135333	0,072969	0,427654	n.s.
log.sigma.5.0.mm.3D_glrlm_LongRunLowGrayLevelEmphasis	2,124913	0,074238	0,431799	n.s.
wavelet.LLL_glrlm_LongRunHighGrayLevelEmphasis	2,118047	0,075086	0,434632	n.s.
wavelet.HLL_gldm_LargeDependenceEmphasis	2,101832	0,077127	0,44431	n.s.
wavelet.LLL_glcm_JointAverage	2,096107	0,077861	0,446401	n.s.
log.sigma.2.0.mm.3D_glrlm_RunLengthNonUniformity	2,089963	0,078656	0,446705	n.s.
log.sigma.3.0.mm.3D_glcm_Correlation	2,090967	0,078525	0,446705	n.s.
wavelet.HLH_firstorder_Skewness	2,077569	0,080284	0,453813	n.s.
wavelet.HLH_firstorder_RootMeanSquared	2,073686	0,080801	0,454601	n.s.
original_glrlm_LongRunEmphasis	2,065131	0,081952	0,458931	n.s.
wavelet.HHL_gldm_SmallDependenceHighGrayLevelEmphasis	2,046366	0,084533	0,469407	n.s.
wavelet.LLH_glrlm_RunVariance	2,042327	0,085099	0,469407	n.s.
wavelet.HHH_glcm_Imc1	2,029589	0,086909	0,469407	n.s.
wavelet.HHH_glszm_GrayLevelNonUniformity	2,027049	0,087274	0,469407	n.s.
wavelet.HHH_glszm_SizeZoneNonUniformityNormalized	2,031486	0,086637	0,469407	n.s.
wavelet.LLL_glszm_ZoneEntropy	2,029057	0,086985	0,469407	n.s.
log.sigma.1.0.mm.3D_gldm_DependenceNonUniformity	2,043312	0,084961	0,469407	n.s.
log.sigma.3.0.mm.3D_glszm_LargeAreaLowGrayLevelEmphasis	2,026652	0,087332	0,469407	n.s.
log.sigma.5.0.mm.3D_gldm_LargeDependenceLowGrayLevelEmphasis	2,038079	0,085699	0,469407	n.s.
wavelet.LHL_firstorder_Range	2,013955	0,089182	0,475112	n.s.

log.sigma.4.0.mm.3D_glrlm_HighGrayLevelRunEmphasis	2,016436	0,088817	0,475112	n.s.
log.sigma.5.0.mm.3D_glcm_Idmn	2,010354	0,089714	0,475839	n.s.
wavelet.HHL_firstorder_Maximum	1,985234	0,093512	0,488273	n.s.
wavelet.HHL_glcm_Correlation	1,991744	0,092512	0,488273	n.s.
wavelet.LHL_gldm_SmallDependenceEmphasis	1,983698	0,093749	0,488273	n.s.
wavelet.LLL_firstorder_Entropy	1,972682	0,095468	0,488273	n.s.
wavelet.LLL_glcm_DifferenceEntropy	1,97533	0,095052	0,488273	n.s.
wavelet.LLL_gldm_LargeDependenceEmphasis	1,9635	0,096925	0,488273	n.s.
log.sigma.3.0.mm.3D_glrlm_RunEntropy	1,963511	0,096923	0,488273	n.s.
log.sigma.3.0.mm.3D_glcm_JointAverage	1,97947	0,094405	0,488273	n.s.
log.sigma.3.0.mm.3D_gldm_LowGrayLevelEmphasis	1,965003	0,096685	0,488273	n.s.
log.sigma.3.0.mm.3D_gldm_LargeDependenceHighGrayLevelEmphasis	1,977075	0,094779	0,488273	n.s.
log.sigma.5.0.mm.3D_firstorder_Skewness	1,96782	0,096237	0,488273	n.s.
original_glrlm_RunVariance	1,98572	0,093437	0,488273	n.s.
original_firstorder_Entropy	1,958274	0,097763	0,490446	n.s.
wavelet.HLL_firstorder_InterquartileRange	1,954774	0,098329	0,491237	n.s.
log.sigma.2.0.mm.3D_glrlm_ShortRunEmphasis	1,950798	0,098976	0,492425	n.s.
wavelet.HLH_glszm_ZoneVariance	1,941887	0,10044	0,492854	n.s.
wavelet.LLL_glcm_SumEntropy	1,946567	0,099668	0,492854	n.s.
log.sigma.4.0.mm.3D_firstorder_MeanAbsoluteDeviation	1,937859	0,101109	0,492854	n.s.
log.sigma.4.0.mm.3D_glcm_ClusterProminence	1,93908	0,100905	0,492854	n.s.
original_glrlm_RunLengthNonUniformity	1,944524	0,100004	0,492854	n.s.
log.sigma.1.0.mm.3D_glrlm_RunEntropy	1,934414	0,101684	0,49366	n.s.
wavelet.LHL_gldm_SmallDependenceHighGrayLevelEmphasis	1,923509	0,103527	0,496411	n.s.
wavelet.LLL_glcm_Idm	1,928443	0,102689	0,496411	n.s.
log.sigma.1.0.mm.3D_glszm_GrayLevelVariance	1,921325	0,1039	0,496411	n.s.
log.sigma.5.0.mm.3D_glszm_GrayLevelNonUniformityNormalized	1,922818	0,103645	0,496411	n.s.
wavelet.HLL_glrlm_RunLengthNonUniformityNormalized	1,906733	0,106426	0,502497	n.s.
wavelet.LLL_glcm_Id	1,907456	0,106299	0,502497	n.s.
log.sigma.3.0.mm.3D_firstorder_Variance	1,906801	0,106414	0,502497	n.s.
wavelet.LHL_firstorder_Maximum	1,879182	0,111359	0,517671	n.s.
wavelet.LHL_glszm_SmallAreaHighGrayLevelEmphasis	1,881502	0,110936	0,517671	n.s.
wavelet.HHH_firstorder_Variance	1,881855	0,110871	0,517671	n.s.
log.sigma.4.0.mm.3D_firstorder_InterquartileRange	1,881448	0,110945	0,517671	n.s.
wavelet.HLL_glrlm_ShortRunEmphasis	1,87077	0,11291	0,522859	n.s.
wavelet.HHH_glcm_Imc2	1,867538	0,113511	0,523629	n.s.
wavelet.LLH_glrlm_LongRunEmphasis	1,855126	0,115849	0,524368	n.s.
log.sigma.3.0.mm.3D_firstorder_MeanAbsoluteDeviation	1,858345	0,115238	0,524368	n.s.
log.sigma.4.0.mm.3D_firstorder_RobustMeanAbsoluteDeviation	1,856136	0,115657	0,524368	n.s.
log.sigma.4.0.mm.3D_gldm_LargeDependenceLowGrayLevelEmphasis	1,856054	0,115672	0,524368	n.s.
original_firstorder_Skewness	1,858711	0,115169	0,524368	n.s.
wavelet.LLL_gldm_GrayLevelNonUniformity	1,851932	0,116458	0,525151	n.s.
wavelet.LHL_glszm_GrayLevelVariance	1,849318	0,116959	0,525442	n.s.
wavelet.HHL_glszm_GrayLevelNonUniformity	1,807551	0,12525	0,528278	n.s.
wavelet.LLH_glrlm_RunLengthNonUniformity	1,823969	0,121925	0,528278	n.s.
wavelet.HLL_glrlm_HighGrayLevelRunEmphasis	1,804263	0,125927	0,528278	n.s.

wavelet.HLL_glrlm_ShortRunHighGrayLevelEmphasis	1,832981	0,120137	0,528278	n.s.
wavelet.HLL_glcm_Autocorrelation	1,837663	0,119217	0,528278	n.s.
wavelet.HLL_gldm_DependenceEntropy	1,815	0,123731	0,528278	n.s.
wavelet.LLL_glrlm_RunEntropy	1,82421	0,121877	0,528278	n.s.
log.sigma.1.0.mm.3D_glrlm_GrayLevelVariance	1,805634	0,125644	0,528278	n.s.
log.sigma.1.0.mm.3D_glcm_Imc2	1,808322	0,125092	0,528278	n.s.
log.sigma.1.0.mm.3D_glszm_LowGrayLevelZoneEmphasis	1,813838	0,123967	0,528278	n.s.
log.sigma.3.0.mm.3D_glrlm_LongRunHighGrayLevelEmphasis	1,840934	0,118579	0,528278	n.s.
log.sigma.3.0.mm.3D_glcm_Autocorrelation	1,831082	0,120511	0,528278	n.s.
log.sigma.3.0.mm.3D_gldm_HighGrayLevelEmphasis	1,813425	0,124051	0,528278	n.s.
log.sigma.4.0.mm.3D_firstorder_Variance	1,806269	0,125514	0,528278	n.s.
log.sigma.4.0.mm.3D_glrlm_LowGrayLevelRunEmphasis	1,815912	0,123546	0,528278	n.s.
log.sigma.5.0.mm.3D_glcm_Imc1	1,832645	0,120203	0,528278	n.s.
log.sigma.5.0.mm.3D_gldm_HighGrayLevelEmphasis	1,819334	0,122855	0,528278	n.s.
original_glcm_SumEntropy	1,817777	0,123169	0,528278	n.s.
original_glcm_MaximumProbability	1,83915	0,118927	0,528278	n.s.
wavelet.LLH_glszm_ZoneEntropy	1,793824	0,128098	0,529066	n.s.
wavelet.HLL_glcm_JointAverage	1,788404	0,129239	0,529066	n.s.
wavelet.HLL_gldm_HighGrayLevelEmphasis	1,798507	0,127119	0,529066	n.s.
log.sigma.1.0.mm.3D_firstorder_Variance	1,784037	0,130166	0,529066	n.s.
log.sigma.2.0.mm.3D_glcm_DifferenceEntropy	1,800156	0,126777	0,529066	n.s.
log.sigma.3.0.mm.3D_glrlm_LowGrayLevelRunEmphasis	1,782512	0,130491	0,529066	n.s.
log.sigma.4.0.mm.3D_gldm_HighGrayLevelEmphasis	1,782427	0,130509	0,529066	n.s.
log.sigma.5.0.mm.3D_firstorder_Minimum	1,793034	0,128263	0,529066	n.s.
original_glrlm_GrayLevelNonUniformity	1,787057	0,129524	0,529066	n.s.
original_gldm_DependenceNonUniformity	1,786592	0,129623	0,529066	n.s.
wavelet.HLL_glcm_Idmn	1,773125	0,132509	0,533582	n.s.
log.sigma.4.0.mm.3D_glrlm_RunEntropy	1,773875	0,132347	0,533582	n.s.
wavelet.HLL_gldm_SmallDependenceEmphasis	1,77016	0,133153	0,534388	n.s.
wavelet.LLL_glrlm_GrayLevelNonUniformity	1,764014	0,134497	0,536373	n.s.
log.sigma.5.0.mm.3D_glrlm_HighGrayLevelRunEmphasis	1,762074	0,134924	0,536373	n.s.
log.sigma.5.0.mm.3D_glcm_Autocorrelation	1,761802	0,134984	0,536373	n.s.
log.sigma.3.0.mm.3D_glszm_LargeAreaHighGrayLevelEmphasis	1,622538	0,136254	0,539638	n.s.
original_glcm_JointEntropy	1,749294	0,13777	0,543852	n.s.
log.sigma.5.0.mm.3D_firstorder_Median	1,74724	0,138232	0,543895	n.s.
wavelet.HHL_glszm_LargeAreaLowGrayLevelEmphasis	1,744764	0,138792	0,544318	n.s.
wavelet.LLH_gldm_LargeDependenceEmphasis	1,723358	0,143722	0,546127	n.s.
wavelet.LLH_gldm_DependenceNonUniformityNormalized	1,727018	0,142868	0,546127	n.s.
wavelet.LHL_glcm_ClusterShade	1,707771	0,142971	0,546127	n.s.
wavelet.LLL_glcm_JointEnergy	1,73528	0,140956	0,546127	n.s.
log.sigma.1.0.mm.3D_glcm_ClusterShade	1,73455	0,141124	0,546127	n.s.
log.sigma.2.0.mm.3D_glrlm_RunVariance	1,729153	0,142371	0,546127	n.s.
log.sigma.3.0.mm.3D_firstorder_InterquartileRange	1,733014	0,141478	0,546127	n.s.
log.sigma.3.0.mm.3D_glrlm_HighGrayLevelRunEmphasis	1,728095	0,142617	0,546127	n.s.
log.sigma.3.0.mm.3D_glcm_ClusterTendency	1,721139	0,144243	0,546127	n.s.
log.sigma.5.0.mm.3D_firstorder_Variance	1,722236	0,143985	0,546127	n.s.

log.sigma.5.0.mm.3D_gldm_DependenceVariance	1,72658	0,14297	0,546127	n.s.
log.sigma.3.0.mm.3D_firstorder_RobustMeanAbsoluteDeviation	1,713845	0,145967	0,550922	n.s.
log.sigma.3.0.mm.3D_glcm_Imc1	1,706597	0,147699	0,555718	n.s.
wavelet.HLH_glrlm_RunPercentage	1,70314	0,148532	0,557112	n.s.
log.sigma.1.0.mm.3D_firstorder_Entropy	1,697153	0,149986	0,560005	n.s.
original_glrlm_GrayLevelVariance	1,696138	0,150234	0,560005	n.s.
log.sigma.3.0.mm.3D_gldm_GrayLevelNonUniformity	1,690746	0,151557	0,561996	n.s.
log.sigma.5.0.mm.3D_glrlm_LongRunEmphasis	1,690158	0,151702	0,561996	n.s.
log.sigma.5.0.mm.3D_glcm_JointAverage	1,687933	0,152251	0,562302	n.s.
wavelet.HHL_glszm_ZonePercentage	1,667178	0,15747	0,562568	n.s.
wavelet.HLL_glcm_Idn	1,685474	0,152861	0,562568	n.s.
wavelet.HLH_gldm_GrayLevelNonUniformity	1,667616	0,157358	0,562568	n.s.
wavelet.LLL_glszm_ZonePercentage	1,680316	0,154147	0,562568	n.s.
log.sigma.1.0.mm.3D_glcm_SumEntropy	1,676413	0,155127	0,562568	n.s.
log.sigma.2.0.mm.3D_glcm_DifferenceVariance	1,665804	0,157821	0,562568	n.s.
log.sigma.3.0.mm.3D_gldm_DependenceEntropy	1,67119	0,156448	0,562568	n.s.
log.sigma.4.0.mm.3D_glcm_JointAverage	1,665379	0,15793	0,562568	n.s.
log.sigma.4.0.mm.3D_glcm_Autocorrelation	1,673089	0,155967	0,562568	n.s.
log.sigma.4.0.mm.3D_gldm_LowGrayLevelEmphasis	1,668521	0,157127	0,562568	n.s.
log.sigma.5.0.mm.3D_firstorder_10Percentile	1,680853	0,154013	0,562568	n.s.
original_firstorder_InterquartileRange	1,671085	0,156475	0,562568	n.s.
log.sigma.4.0.mm.3D_firstorder_Kurtosis	1,659918	0,159335	0,565899	n.s.
wavelet.LLH_glcm_ClusterShade	1,631415	0,162412	0,568131	n.s.
wavelet.HLL_gldm_DependenceVariance	1,63075	0,167042	0,568131	n.s.
wavelet.LHH_glszm_LargeAreaLowGrayLevelEmphasis	1,648104	0,162415	0,568131	n.s.
wavelet.HLH_glcm_ClusterProminence	1,632853	0,166475	0,568131	n.s.
wavelet.HHH_glszm_ZoneEntropy	1,63779	0,16515	0,568131	n.s.
wavelet.LLL_glszm_LargeAreaEmphasis	1,635053	0,165883	0,568131	n.s.
log.sigma.1.0.mm.3D_firstorder_MeanAbsoluteDeviation	1,652649	0,161224	0,568131	n.s.
log.sigma.1.0.mm.3D_glszm_SizeZoneNonUniformityNormalized	1,635986	0,165633	0,568131	n.s.
log.sigma.2.0.mm.3D_glszm_GrayLevelVariance	1,653917	0,160893	0,568131	n.s.
log.sigma.2.0.mm.3D_gldm_LargeDependenceEmphasis	1,648311	0,162361	0,568131	n.s.
log.sigma.3.0.mm.3D_firstorder_Entropy	1,631886	0,166735	0,568131	n.s.
log.sigma.3.0.mm.3D_gldm_GrayLevelVariance	1,640078	0,16454	0,568131	n.s.
log.sigma.5.0.mm.3D_firstorder_MeanAbsoluteDeviation	1,633975	0,166173	0,568131	n.s.
log.sigma.5.0.mm.3D_gldm_SmallDependenceHighGrayLevelEmphasis	1,642956	0,163775	0,568131	n.s.
original_firstorder_Variance	1,636438	0,165512	0,568131	n.s.
log.sigma.2.0.mm.3D_glcm_InverseVariance	1,628896	0,167543	0,568232	n.s.
log.sigma.1.0.mm.3D_glcm_Correlation	1,623014	0,169144	0,57205	n.s.
wavelet.HHL_glszm_HighGrayLevelZoneEmphasis	1,608504	0,173155	0,572552	n.s.
wavelet.LHL_firstorder_Skewness	1,588503	0,174397	0,572552	n.s.
wavelet.HLL_gldm_DependenceNonUniformityNormalized	1,612146	0,17214	0,572552	n.s.
wavelet.HLH_glcm_Id	1,602003	0,17498	0,572552	n.s.
wavelet.HLH_glszm_SizeZoneNonUniformityNormalized	1,602689	0,174787	0,572552	n.s.
wavelet.HHH_gldm_LargeDependenceLowGrayLevelEmphasis	1,605003	0,174136	0,572552	n.s.
wavelet.LLL_glrlm_GrayLevelVariance	1,601935	0,174999	0,572552	n.s.

log.sigma.1.0.mm.3D_glrlm_RunLengthNonUniformity	1,614877	0,171382	0,572552	n.s.
log.sigma.2.0.mm.3D_glszm_GrayLevelNonUniformityNormalized	1,602068	0,174962	0,572552	n.s.
log.sigma.2.0.mm.3D_gldm_GrayLevelNonUniformity	1,612904	0,171929	0,572552	n.s.
original_firstorder_MeanAbsoluteDeviation	1,611547	0,172306	0,572552	n.s.
original_glcm_JointEnergy	1,605831	0,173903	0,572552	n.s.
wavelet.LHL_glszm_GrayLevelNonUniformityNormalized	1,598925	0,175851	0,572614	n.s.
wavelet.HLL_glszm_LargeAreaHighGrayLevelEmphasis	1,598508	0,175969	0,572614	n.s.
wavelet.HLL_glrlm_LongRunHighGrayLevelEmphasis	1,594176	0,177202	0,573091	n.s.
log.sigma.1.0.mm.3D_firstorder_Skewness	1,59512	0,176933	0,573091	n.s.
log.sigma.3.0.mm.3D_glszm_LargeAreaEmphasis	1,592981	0,177544	0,573091	n.s.
log.sigma.1.0.mm.3D_glrlm_LongRunEmphasis	1,590136	0,17836	0,573229	n.s.
log.sigma.3.0.mm.3D_glcm_SumSquares	1,589512	0,178539	0,573229	n.s.
log.sigma.2.0.mm.3D_glrlm_RunPercentage	1,573928	0,183074	0,586226	n.s.
wavelet.LHL_glrlm_RunVariance	1,546352	0,191363	0,588587	n.s.
wavelet.LHL_glrlm_LongRunEmphasis	1,536447	0,194424	0,588587	n.s.
wavelet.LHL_gldm_GrayLevelNonUniformity	1,542786	0,19246	0,588587	n.s.
wavelet.HLL_firstorder_Minimum	1,556335	0,188323	0,588587	n.s.
wavelet.HLL_glcm_Correlation	1,537074	0,194229	0,588587	n.s.
wavelet.LHH_firstorder_Maximum	1,553599	0,189152	0,588587	n.s.
wavelet.HLH_firstorder_Energy	1,441025	0,194478	0,588587	n.s.
wavelet.HLH_firstorder_TotalEnergy	1,441025	0,194478	0,588587	n.s.
wavelet.HLH_glrlm_GrayLevelNonUniformity	1,543478	0,192247	0,588587	n.s.
wavelet.HLH_glrlm_RunLengthNonUniformityNormalized	1,54742	0,191036	0,588587	n.s.
wavelet.HLH_glcm_Idm	1,545476	0,191632	0,588587	n.s.
wavelet.HLH_glszm_LargeAreaHighGrayLevelEmphasis	1,535993	0,194566	0,588587	n.s.
wavelet.LLL_glszm_ZoneVariance	1,548268	0,190776	0,588587	n.s.
wavelet.LLL_gldm_GrayLevelVariance	1,563726	0,186101	0,588587	n.s.
wavelet.LLL_gldm_LargeDependenceHighGrayLevelEmphasis	1,557872	0,187859	0,588587	n.s.
log.sigma.1.0.mm.3D_glrlm_GrayLevelNonUniformityNormalized	1,543653	0,192193	0,588587	n.s.
log.sigma.2.0.mm.3D_firstorder_Range	1,552002	0,189637	0,588587	n.s.
log.sigma.3.0.mm.3D_firstorder_Uniformity	1,541206	0,192948	0,588587	n.s.
log.sigma.4.0.mm.3D_glcm_Imc1	1,550901	0,189972	0,588587	n.s.
log.sigma.4.0.mm.3D_glszm_GrayLevelVariance	1,550066	0,190227	0,588587	n.s.
original_glcm_DifferenceEntropy	1,542519	0,192542	0,588587	n.s.
original_gldm_GrayLevelVariance	1,563938	0,186038	0,588587	n.s.
wavelet.LHL_glszm_LowGrayLevelZoneEmphasis	1,53232	0,195714	0,590574	n.s.
wavelet.LLH_firstorder_Energy	1,42438	0,200748	0,593639	n.s.
wavelet.LLH_firstorder_TotalEnergy	1,42438	0,200748	0,593639	n.s.
wavelet.LLH_glszm_SmallAreaHighGrayLevelEmphasis	1,514375	0,201411	0,593639	n.s.
wavelet.LHL_glcm_Imc2	1,520345	0,199499	0,593639	n.s.
wavelet.LLL_firstorder_Variance	1,516397	0,200761	0,593639	n.s.
wavelet.LLL_firstorder_MeanAbsoluteDeviation	1,521466	0,199142	0,593639	n.s.
log.sigma.2.0.mm.3D_glcm_Id	1,512079	0,202151	0,593639	n.s.
log.sigma.2.0.mm.3D_glszm_LargeAreaEmphasis	1,513532	0,201682	0,593639	n.s.
log.sigma.3.0.mm.3D_glcm_SumEntropy	1,515072	0,201187	0,593639	n.s.
log.sigma.4.0.mm.3D_glrlm_ShortRunLowGrayLevelEmphasis	1,521481	0,199137	0,593639	n.s.

log.sigma.5.0.mm.3D_gldm_DependenceNonUniformityNormalized	1,512073	0,202153	0,593639	n.s.
wavelet.LLL_glszm_LargeAreaLowGrayLevelEmphasis	1,505284	0,204355	0,5962	n.s.
log.sigma.1.0.mm.3D_gldm_GrayLevelVariance	1,504808	0,204511	0,5962	n.s.
log.sigma.5.0.mm.3D_gldm_LowGrayLevelEmphasis	1,505663	0,204232	0,5962	n.s.
wavelet.HHL_firstorder_Entropy	1,498816	0,206474	0,598238	n.s.
wavelet.HHH_glszm_SmallAreaLowGrayLevelEmphasis	1,498129	0,2067	0,598238	n.s.
original_glrlm_GrayLevelNonUniformityNormalized	1,498642	0,206531	0,598238	n.s.
wavelet.LHL_glszm_ZonePercentage	1,496205	0,207335	0,598548	n.s.
wavelet.LHL_glszm_LargeAreaEmphasis	1,494795	0,207801	0,598548	n.s.
log.sigma.2.0.mm.3D_glszm_SmallAreaHighGrayLevelEmphasis	1,493024	0,208389	0,598807	n.s.
log.sigma.2.0.mm.3D_glcm_Idm	1,485554	0,210882	0,604528	n.s.
wavelet.HHL_glrlm_GrayLevelVariance	1,482399	0,211943	0,606128	n.s.
wavelet.HHL_firstorder_Uniformity	1,47823	0,213353	0,607274	n.s.
wavelet.HLL_glszm_LargeAreaLowGrayLevelEmphasis	1,479639	0,212876	0,607274	n.s.
original_glrlm_ShortRunEmphasis	1,470254	0,216074	0,613569	n.s.
wavelet.HHL_firstorder_90Percentile	1,464951	0,217901	0,61366	n.s.
wavelet.LHL_glrlm_RunPercentage	1,462775	0,218655	0,61366	n.s.
wavelet.LHL_glszm_ZoneVariance	1,462868	0,218622	0,61366	n.s.
wavelet.HLH_glcm_DifferenceAverage	1,464881	0,217925	0,61366	n.s.
wavelet.LLL_glcm_DifferenceAverage	1,468213	0,216776	0,61366	n.s.
log.sigma.3.0.mm.3D_glcm_MaximumProbability	1,459964	0,219632	0,614969	n.s.
wavelet.HHH_gldm_LargeDependenceHighGrayLevelEmphasis	1,4569	0,220701	0,615103	n.s.
log.sigma.5.0.mm.3D_glcm_ClusterProminence	1,457691	0,220425	0,615103	n.s.
wavelet.HHL_firstorder_Range	1,419896	0,233997	0,616663	n.s.
wavelet.HHL_glrlm_RunLengthNonUniformityNormalized	1,399909	0,241476	0,616663	n.s.
wavelet.HHL_glrlm_GrayLevelNonUniformityNormalized	1,447444	0,224032	0,616663	n.s.
wavelet.HHL_gldm_GrayLevelVariance	1,445594	0,224689	0,616663	n.s.
wavelet.HHL_gldm_SmallDependenceEmphasis	1,402938	0,240329	0,616663	n.s.
wavelet.LHL_firstorder_Minimum	1,421258	0,233495	0,616663	n.s.
wavelet.LHL_glrlm_GrayLevelNonUniformity	1,404691	0,239668	0,616663	n.s.
wavelet.LHL_gldm_LargeDependenceEmphasis	1,441785	0,226047	0,616663	n.s.
wavelet.HLL_glszm_GrayLevelVariance	1,409741	0,237771	0,616663	n.s.
wavelet.HLL_gldm_LargeDependenceHighGrayLevelEmphasis	1,409922	0,237703	0,616663	n.s.
wavelet.HLL_gldm_DependenceNonUniformity	1,413859	0,236234	0,616663	n.s.
wavelet.HLH_glrlm_ShortRunEmphasis	1,398783	0,241904	0,616663	n.s.
wavelet.HLH_glcm_JointEnergy	1,446536	0,224354	0,616663	n.s.
wavelet.HLH_glcm_InverseVariance	1,421718	0,233326	0,616663	n.s.
wavelet.HLH_glcm_Imc1	1,396069	0,242937	0,616663	n.s.
wavelet.HLH_glcm_ClusterTendency	1,441456	0,226165	0,616663	n.s.
wavelet.HLH_glszm_GrayLevelNonUniformity	1,415133	0,23576	0,616663	n.s.
wavelet.HLH_glszm_ZoneEntropy	1,416601	0,235216	0,616663	n.s.
wavelet.HLH_gldm_SmallDependenceHighGrayLevelEmphasis	1,39919	0,241749	0,616663	n.s.
wavelet.HLH_gldm_LargeDependenceEmphasis	1,429023	0,230652	0,616663	n.s.
wavelet.HHH_glrlm_LongRunLowGrayLevelEmphasis	1,4142	0,236107	0,616663	n.s.
wavelet.HHH_glcm_Idn	1,446351	0,22442	0,616663	n.s.
wavelet.HHH_glcm_Idmn	1,445025	0,224892	0,616663	n.s.

wavelet.HHH_gldm_SmallDependenceHighGrayLevelEmphasis	1,427651	0,231152	0,616663	n.s.
wavelet.HHH_gldm_DependenceNonUniformityNormalized	1,40023	0,241355	0,616663	n.s.
wavelet.LLL_firstorder_Uniformity	1,395159	0,243285	0,616663	n.s.
wavelet.LLL_firstorder_Minimum	1,428653	0,230787	0,616663	n.s.
wavelet.LLL_glrlm_GrayLevelNonUniformityNormalized	1,403979	0,239936	0,616663	n.s.
wavelet.LLL_glcm_DifferenceVariance	1,4383	0,227297	0,616663	n.s.
wavelet.LLL_glcm_InverseVariance	1,403741	0,240026	0,616663	n.s.
log.sigma.1.0.mm.3D_firstorder_RootMeanSquared	1,406192	0,239102	0,616663	n.s.
log.sigma.1.0.mm.3D_glcm_JointEntropy	1,43786	0,227455	0,616663	n.s.
log.sigma.2.0.mm.3D_glrlm_RunLengthNonUniformityNormalized	1,420513	0,23377	0,616663	n.s.
log.sigma.2.0.mm.3D_glcm_JointEntropy	1,402199	0,240608	0,616663	n.s.
log.sigma.2.0.mm.3D_glcm_SumEntropy	1,415192	0,235739	0,616663	n.s.
log.sigma.2.0.mm.3D_glcm_DifferenceAverage	1,447482	0,224019	0,616663	n.s.
log.sigma.3.0.mm.3D_glrlm_GrayLevelNonUniformityNormalized	1,401691	0,240801	0,616663	n.s.
log.sigma.3.0.mm.3D_glcm_JointEnergy	1,430832	0,229995	0,616663	n.s.
log.sigma.3.0.mm.3D_glcm_JointEntropy	1,430911	0,229966	0,616663	n.s.
log.sigma.3.0.mm.3D_glszm_ZoneVariance	1,397194	0,242509	0,616663	n.s.
log.sigma.4.0.mm.3D_firstorder_10Percentile	1,43092	0,229962	0,616663	n.s.
original_glrlm_RunPercentage	1,434678	0,228601	0,616663	n.s.
original_gldm_LargeDependenceEmphasis	1,450822	0,222837	0,616663	n.s.
wavelet.LLL_glcm_MaximumProbability	1,393419	0,24395	0,617051	n.s.
wavelet.LHL_glszm_LargeAreaLowGrayLevelEmphasis	1,372154	0,252216	0,618059	n.s.
wavelet.HLL_firstorder_RootMeanSquared	1,374814	0,251169	0,618059	n.s.
wavelet.HLL_glszm_ZonePercentage	1,38747	0,246238	0,618059	n.s.
wavelet.HLH_glcm_SumSquares	1,376245	0,250607	0,618059	n.s.
wavelet.HHH_glrlm_LongRunHighGrayLevelEmphasis	1,37649	0,250511	0,618059	n.s.
wavelet.HHH_glcm_JointAverage	1,376997	0,250312	0,618059	n.s.
wavelet.HHH_glszm_GrayLevelVariance	1,381404	0,248591	0,618059	n.s.
wavelet.HHH_gldm_LowGrayLevelEmphasis	1,383948	0,247601	0,618059	n.s.
log.sigma.1.0.mm.3D_glcm_ClusterProminence	1,370347	0,25293	0,618059	n.s.
log.sigma.1.0.mm.3D_glszm_LargeAreaEmphasis	1,370108	0,253025	0,618059	n.s.
log.sigma.2.0.mm.3D_firstorder_Skewness	1,379627	0,249283	0,618059	n.s.
log.sigma.2.0.mm.3D_glszm_LargeAreaLowGrayLevelEmphasis	1,37516	0,251033	0,618059	n.s.
log.sigma.3.0.mm.3D_firstorder_Skewness	1,373158	0,251821	0,618059	n.s.
log.sigma.3.0.mm.3D_glrlm_ShortRunHighGrayLevelEmphasis	1,375649	0,250841	0,618059	n.s.
log.sigma.3.0.mm.3D_glszm_SmallAreaEmphasis	1,379391	0,249376	0,618059	n.s.
log.sigma.5.0.mm.3D_glszm_SizeZoneNonUniformityNormalized	1,386276	0,2467	0,618059	n.s.
original_firstorder_Uniformity	1,36998	0,253076	0,618059	n.s.
log.sigma.1.0.mm.3D_firstorder_InterquartileRange	1,367037	0,254242	0,618399	n.s.
log.sigma.5.0.mm.3D_glrlm_LowGrayLevelRunEmphasis	1,367436	0,254084	0,618399	n.s.
wavelet.LLL_glszm_LargeAreaHighGrayLevelEmphasis	1,361876	0,256301	0,620897	n.s.
log.sigma.2.0.mm.3D_gldm_SmallDependenceEmphasis	1,362195	0,256173	0,620897	n.s.
wavelet.HHH_glcm_Autocorrelation	1,359	0,257454	0,621191	n.s.
log.sigma.3.0.mm.3D_glrlm_GrayLevelVariance	1,359394	0,257295	0,621191	n.s.
wavelet.HHH_glszm_ZoneVariance	1,354412	0,259303	0,623235	n.s.
log.sigma.5.0.mm.3D_gldm_SmallDependenceEmphasis	1,35433	0,259336	0,623235	n.s.

original_glszm_LargeAreaLowGrayLevelEmphasis	1,352348	0,260139	0,623918	n.s.
wavelet.LHL_glrlm_RunLengthNonUniformityNormalized	1,342508	0,264156	0,626197	n.s.
wavelet.LHL_glszm_HighGrayLevelZoneEmphasis	1,341649	0,264509	0,626197	n.s.
wavelet.HLH_gldm_SmallDependenceLowGrayLevelEmphasis	1,342384	0,264207	0,626197	n.s.
wavelet.HHH_gldm_HighGrayLevelEmphasis	1,338712	0,26572	0,626197	n.s.
wavelet.LLL_glcm_SumSquares	1,340239	0,26509	0,626197	n.s.
wavelet.LLL_glcm_ClusterTendency	1,338592	0,26577	0,626197	n.s.
log.sigma.2.0.mm.3D_glrlm_GrayLevelNonUniformityNormalized	1,341764	0,264462	0,626197	n.s.
log.sigma.4.0.mm.3D_glszm_SmallAreaHighGrayLevelEmphasis	1,344477	0,263347	0,626197	n.s.
log.sigma.4.0.mm.3D_gldm_DependenceNonUniformity	1,338849	0,265663	0,626197	n.s.
wavelet.LHL_firstorder_Kurtosis	1,326579	0,266836	0,626259	n.s.
original_firstorder_RobustMeanAbsoluteDeviation	1,33674	0,266536	0,626259	n.s.
wavelet.HLL_glszm_SmallAreaEmphasis	1,333738	0,267782	0,627256	n.s.
log.sigma.3.0.mm.3D_firstorder_Minimum	1,331492	0,268718	0,628225	n.s.
wavelet.HLH_firstorder_Uniformity	1,330062	0,269315	0,628402	n.s.
wavelet.HLH_gldm_GrayLevelVariance	1,326572	0,270778	0,630592	n.s.
wavelet.LHL_glszm_SmallAreaEmphasis	1,325219	0,271346	0,630697	n.s.
wavelet.HHL_glrlm_ShortRunEmphasis	1,257786	0,301045	0,631921	n.s.
wavelet.HHL_glrlm_RunPercentage	1,304279	0,280284	0,631921	n.s.
wavelet.HHL_glrlm_ShortRunHighGrayLevelEmphasis	1,268598	0,296103	0,631921	n.s.
wavelet.HHL_glrlm_LowGrayLevelRunEmphasis	1,287042	0,287832	0,631921	n.s.
wavelet.HHL_glrlm_ShortRunLowGrayLevelEmphasis	1,285059	0,288712	0,631921	n.s.
wavelet.HHL_glcm_JointEntropy	1,252407	0,30353	0,631921	n.s.
wavelet.HHL_glcm_SumEntropy	1,250798	0,304276	0,631921	n.s.
wavelet.HHL_glcm_ClusterProminence	1,293456	0,285003	0,631921	n.s.
wavelet.HHL_glcm_ClusterTendency	1,264824	0,29782	0,631921	n.s.
wavelet.HHL_glszm_ZoneEntropy	1,307285	0,278985	0,631921	n.s.
wavelet.HHL_gldm_DependenceEntropy	1,249174	0,305031	0,631921	n.s.
wavelet.LLH_glcm_Idn	1,287355	0,287694	0,631921	n.s.
wavelet.LLH_glcm_InverseVariance	1,247121	0,305989	0,631921	n.s.
wavelet.LHL_glrlm_ShortRunEmphasis	1,289285	0,28684	0,631921	n.s.
wavelet.LHL_glcm_ClusterProminence	1,283061	0,2896	0,631921	n.s.
wavelet.LHL_glcm_MaximumProbability	1,292711	0,285331	0,631921	n.s.
wavelet.LHL_glszm_SmallAreaLowGrayLevelEmphasis	1,252484	0,303494	0,631921	n.s.
wavelet.LHL_gldm_DependenceVariance	1,251795	0,303813	0,631921	n.s.
wavelet.LHL_gldm_LargeDependenceHighGrayLevelEmphasis	1,256244	0,301755	0,631921	n.s.
wavelet.HLL_glrlm_LowGrayLevelRunEmphasis	1,250905	0,304226	0,631921	n.s.
wavelet.HLL_glrlm_LongRunLowGrayLevelEmphasis	1,277759	0,291969	0,631921	n.s.
wavelet.HLL_glszm_SmallAreaLowGrayLevelEmphasis	1,266979	0,296838	0,631921	n.s.
wavelet.HLL_glszm_HighGrayLevelZoneEmphasis	1,320927	0,273158	0,631921	n.s.
wavelet.HLL_gldm_LargeDependenceLowGrayLevelEmphasis	1,256549	0,301615	0,631921	n.s.
wavelet.HLH_firstorder_Minimum	1,266403	0,2971	0,631921	n.s.
wavelet.HLH_glrlm_RunLengthNonUniformity	1,291245	0,285976	0,631921	n.s.
wavelet.HLH_glrlm_GrayLevelVariance	1,249279	0,304982	0,631921	n.s.
wavelet.HLH_glrlm_GrayLevelNonUniformityNormalized	1,272701	0,294246	0,631921	n.s.
wavelet.HLH_glcm_Imc2	1,267774	0,296477	0,631921	n.s.

wavelet.HLH_glcm_SumEntropy	1,312825	0,276606	0,631921	n.s.
wavelet.HLH_glcm_Contrast	1,292808	0,285288	0,631921	n.s.
wavelet.HLH_gldm_DependenceNonUniformity	1,255764	0,301977	0,631921	n.s.
wavelet.HHH_glrlm_HighGrayLevelRunEmphasis	1,298584	0,282759	0,631921	n.s.
wavelet.HHH_glrlm_ShortRunHighGrayLevelEmphasis	1,26104	0,29955	0,631921	n.s.
wavelet.HHH_glrlm_LowGrayLevelRunEmphasis	1,291956	0,285663	0,631921	n.s.
wavelet.HHH_glcm_JointEnergy	1,256041	0,301849	0,631921	n.s.
wavelet.HHH_glszm_LargeAreaLowGrayLevelEmphasis	1,218406	0,29309	0,631921	n.s.
wavelet.HHH_glszm_GrayLevelNonUniformityNormalized	1,309021	0,278238	0,631921	n.s.
wavelet.HHH_glszm_LargeAreaHighGrayLevelEmphasis	1,238239	0,282951	0,631921	n.s.
wavelet.LLL_firstorder_Skewness	1,320427	0,27337	0,631921	n.s.
wavelet.LLL_firstorder_RobustMeanAbsoluteDeviation	1,313131	0,276475	0,631921	n.s.
wavelet.LLL_firstorder_InterquartileRange	1,263125	0,298596	0,631921	n.s.
wavelet.LLL_glszm_LowGrayLevelZoneEmphasis	1,277474	0,292097	0,631921	n.s.
wavelet.LLL_gldm_DependenceVariance	1,259582	0,300219	0,631921	n.s.
log.sigma.1.0.mm.3D_firstorder_Uniformity	1,288626	0,287131	0,631921	n.s.
log.sigma.1.0.mm.3D_firstorder_Mean	1,306971	0,279121	0,631921	n.s.
log.sigma.1.0.mm.3D_firstorder_RobustMeanAbsoluteDeviation	1,247242	0,305932	0,631921	n.s.
log.sigma.1.0.mm.3D_glcm_ClusterTendency	1,312475	0,276756	0,631921	n.s.
log.sigma.1.0.mm.3D_glszm_ZoneVariance	1,274247	0,293548	0,631921	n.s.
log.sigma.1.0.mm.3D_glszm_LargeAreaHighGrayLevelEmphasis	1,288247	0,287299	0,631921	n.s.
log.sigma.2.0.mm.3D_firstorder_Entropy	1,27685	0,292377	0,631921	n.s.
log.sigma.2.0.mm.3D_glrlm_LongRunLowGrayLevelEmphasis	1,257681	0,301093	0,631921	n.s.
log.sigma.2.0.mm.3D_glcm_Contrast	1,286586	0,288034	0,631921	n.s.
log.sigma.2.0.mm.3D_gldm_DependenceVariance	1,250691	0,304326	0,631921	n.s.
log.sigma.2.0.mm.3D_gldm_DependenceEntropy	1,291488	0,285869	0,631921	n.s.
log.sigma.5.0.mm.3D_firstorder_Mean	1,286309	0,288157	0,631921	n.s.
log.sigma.5.0.mm.3D_firstorder_RootMeanSquared	1,279228	0,291311	0,631921	n.s.
log.sigma.5.0.mm.3D_glrlm_GrayLevelNonUniformity	1,286419	0,288108	0,631921	n.s.
original_glrlm_RunLengthNonUniformityNormalized	1,254083	0,302753	0,631921	n.s.
original_glcm_DifferenceVariance	1,312484	0,276752	0,631921	n.s.
original_glcm_Imc2	1,256152	0,301798	0,631921	n.s.
original_glcm_ClusterTendency	1,261301	0,299431	0,631921	n.s.
original_glcm_ClusterShade	1,262526	0,298869	0,631921	n.s.
original_glszm_ZoneVariance	1,313483	0,276324	0,631921	n.s.
original_glszm_LargeAreaEmphasis	1,291295	0,285954	0,631921	n.s.
wavelet.HLL_gldm_LowGrayLevelEmphasis	1,244983	0,306988	0,632036	n.s.
log.sigma.2.0.mm.3D_glrlm_GrayLevelVariance	1,244757	0,307094	0,632036	n.s.
wavelet.HHH_firstorder_Median	1,235678	0,307775	0,632358	n.s.
wavelet.HHL_glszm_SizeZoneNonUniformity	1,240332	0,309171	0,632815	n.s.
wavelet.HHH_glszm_LargeAreaEmphasis	1,186281	0,310101	0,632815	n.s.
log.sigma.1.0.mm.3D_glcm_Idmn	1,238699	0,309941	0,632815	n.s.
log.sigma.5.0.mm.3D_glszm_ZonePercentage	1,239022	0,309789	0,632815	n.s.
wavelet.HHL_firstorder_Variance	1,229673	0,314224	0,633409	n.s.
wavelet.HHL_glcm_Id	1,227787	0,315126	0,633409	n.s.
wavelet.HLL_glrlm_ShortRunLowGrayLevelEmphasis	1,235453	0,311476	0,633409	n.s.

wavelet.HLH_glcm_JointEntropy	1,227927	0,315059	0,633409	n.s.
log.sigma.2.0.mm.3D_gldm_LargeDependenceLowGrayLevelEmphasis	1,231735	0,313242	0,633409	n.s.
log.sigma.4.0.mm.3D_gldm_LargeDependenceHighGrayLevelEmphasis	1,229602	0,314259	0,633409	n.s.
original_glcm_Id	1,231953	0,313138	0,633409	n.s.
original_glcm_SumSquares	1,234426	0,311963	0,633409	n.s.
original_glszm_SmallAreaHighGrayLevelEmphasis	1,229491	0,314311	0,633409	n.s.
wavelet.HHL_glrlm_LongRunLowGrayLevelEmphasis	1,224431	0,316735	0,633502	n.s.
wavelet.HHL_gldm_LowGrayLevelEmphasis	1,224457	0,316723	0,633502	n.s.
log.sigma.2.0.mm.3D_glszm_GrayLevelNonUniformity	1,224398	0,316751	0,633502	n.s.
log.sigma.1.0.mm.3D_glcm_MaximumProbability	1,221823	0,317991	0,634927	n.s.
original_gldm_DependenceVariance	1,219362	0,319179	0,636245	n.s.
wavelet.HHL_glrlm_GrayLevelNonUniformity	1,177748	0,339841	0,639625	n.s.
wavelet.HHL_glrlm_HighGrayLevelRunEmphasis	1,199113	0,329099	0,639625	n.s.
wavelet.HHL_glrlm_RunEntropy	1,207775	0,324824	0,639625	n.s.
wavelet.HHL_glcm_SumSquares	1,18771	0,334797	0,639625	n.s.
wavelet.HHL_glcm_Idm	1,191134	0,333077	0,639625	n.s.
wavelet.HHL_glszm_LargeAreaEmphasis	1,181403	0,337983	0,639625	n.s.
wavelet.HHL_gldm_LargeDependenceEmphasis	1,188824	0,334237	0,639625	n.s.
wavelet.LHL_glrlm_GrayLevelVariance	1,192687	0,3323	0,639625	n.s.
wavelet.LHL_gldm_DependenceNonUniformityNormalized	1,20695	0,32523	0,639625	n.s.
wavelet.HLL_firstorder_Mean	1,195751	0,33077	0,639625	n.s.
wavelet.HLL_firstorder_Range	1,172529	0,342509	0,639625	n.s.
wavelet.HLL_glcm_InverseVariance	1,209119	0,324165	0,639625	n.s.
wavelet.HLL_glszm_LowGrayLevelZoneEmphasis	1,211631	0,322936	0,639625	n.s.
wavelet.HLL_gldm_SmallDependenceHighGrayLevelEmphasis	1,212572	0,322477	0,639625	n.s.
wavelet.HLL_gldm_SmallDependenceLowGrayLevelEmphasis	1,172285	0,342633	0,639625	n.s.
wavelet.LHH_glcm_Imc1	1,172363	0,342593	0,639625	n.s.
wavelet.LHH_glszm_SmallAreaLowGrayLevelEmphasis	1,172686	0,342428	0,639625	n.s.
wavelet.HLH_glrlm_LongRunHighGrayLevelEmphasis	1,17224	0,342657	0,639625	n.s.
wavelet.HLH_glcm_JointAverage	1,186617	0,335347	0,639625	n.s.
wavelet.HLH_glcm_Correlation	1,195021	0,331134	0,639625	n.s.
wavelet.HLH_gldm_SmallDependenceEmphasis	1,18825	0,334525	0,639625	n.s.
wavelet.HHH_glrlm_ShortRunLowGrayLevelEmphasis	1,191461	0,332913	0,639625	n.s.
wavelet.LLL_glcm_Contrast	1,178166	0,339628	0,639625	n.s.
wavelet.LLL_glszm_GrayLevelNonUniformity	1,193896	0,331696	0,639625	n.s.
log.sigma.1.0.mm.3D_glrlm_RunVariance	1,210505	0,323487	0,639625	n.s.
log.sigma.1.0.mm.3D_glcm_DifferenceEntropy	1,186047	0,335634	0,639625	n.s.
log.sigma.1.0.mm.3D_glcm_SumSquares	1,197589	0,329856	0,639625	n.s.
log.sigma.1.0.mm.3D_glszm_ZonePercentage	1,193211	0,332038	0,639625	n.s.
log.sigma.2.0.mm.3D_glszm_SmallAreaEmphasis	1,194608	0,33134	0,639625	n.s.
log.sigma.2.0.mm.3D_glszm_ZoneVariance	1,206152	0,325622	0,639625	n.s.
log.sigma.2.0.mm.3D_gldm_DependenceNonUniformityNormalized	1,178921	0,339244	0,639625	n.s.
log.sigma.3.0.mm.3D_glrlm_RunLengthNonUniformity	1,176314	0,340572	0,639625	n.s.
log.sigma.3.0.mm.3D_glrlm_ShortRunLowGrayLevelEmphasis	1,179627	0,338885	0,639625	n.s.
log.sigma.3.0.mm.3D_glszm_GrayLevelVariance	1,173843	0,341835	0,639625	n.s.
log.sigma.4.0.mm.3D_glrlm_LongRunHighGrayLevelEmphasis	1,192759	0,332264	0,639625	n.s.

log.sigma.5.0.mm.3D_firstorder_RobustMeanAbsoluteDeviation	1,193067	0,33211	0,639625	n.s.
log.sigma.5.0.mm.3D_glszm_LargeAreaHighGrayLevelEmphasis	1,139347	0,336273	0,639625	n.s.
log.sigma.5.0.mm.3D_gldm_LargeDependenceHighGrayLevelEmphasis	1,185949	0,335684	0,639625	n.s.
original_glcm_Idm	1,202031	0,327654	0,639625	n.s.
original_glszm_SmallAreaLowGrayLevelEmphasis	1,198301	0,329502	0,639625	n.s.
original_gldm_SmallDependenceHighGrayLevelEmphasis	1,180607	0,338387	0,639625	n.s.
log.sigma.5.0.mm.3D_glszm_SmallAreaEmphasis	1,164282	0,3435	0,640207	n.s.
wavelet.HHL_glcm_DifferenceAverage	1,166328	0,3457	0,640867	n.s.
wavelet.LLH_glszm_SmallAreaEmphasis	1,165982	0,345878	0,640867	n.s.
wavelet.LHL_glrlm_ShortRunLowGrayLevelEmphasis	1,164708	0,346537	0,640867	n.s.
wavelet.LHH_glszm_LowGrayLevelZoneEmphasis	1,163723	0,347048	0,640867	n.s.
log.sigma.2.0.mm.3D_firstorder_Energy	1,121034	0,346911	0,640867	n.s.
log.sigma.2.0.mm.3D_firstorder_TotalEnergy	1,121034	0,346911	0,640867	n.s.
log.sigma.4.0.mm.3D_glszm_SmallAreaEmphasis	1,156265	0,347713	0,641112	n.s.
wavelet.HHH_glszm_ZonePercentage	1,159213	0,349391	0,64224	n.s.
log.sigma.2.0.mm.3D_gldm_DependenceNonUniformity	1,159249	0,349372	0,64224	n.s.
wavelet.HHL_glszm_ZoneVariance	1,152354	0,35298	0,646862	n.s.
wavelet.HHH_glrlm_GrayLevelVariance	1,15267	0,352814	0,646862	n.s.
wavelet.LHL_glcm_Id	1,145548	0,356571	0,647528	n.s.
wavelet.LHL_gldm_GrayLevelVariance	1,146694	0,355964	0,647528	n.s.
wavelet.HLL_glszm_GrayLevelNonUniformity	1,145777	0,35645	0,647528	n.s.
wavelet.HLH_gldm_LargeDependenceHighGrayLevelEmphasis	1,147972	0,355289	0,647528	n.s.
log.sigma.2.0.mm.3D_gldm_SmallDependenceHighGrayLevelEmphasis	1,150102	0,354165	0,647528	n.s.
original_glszm_SizeZoneNonUniformity	1,145745	0,356467	0,647528	n.s.
wavelet.LLH_firstorder_RootMeanSquared	1,142252	0,35832	0,648177	n.s.
wavelet.LHL_glrlm_LowGrayLevelRunEmphasis	1,141832	0,358543	0,648177	n.s.
log.sigma.4.0.mm.3D_glszm_GrayLevelNonUniformityNormalized	1,143297	0,357764	0,648177	n.s.
wavelet.HHL_gldm_HighGrayLevelEmphasis	1,138832	0,360143	0,648707	n.s.
wavelet.LLH_firstorder_Mean	1,139204	0,359944	0,648707	n.s.
wavelet.HLH_firstorder_Entropy	1,138251	0,360453	0,648707	n.s.
wavelet.HHL_gldm_LargeDependenceLowGrayLevelEmphasis	1,135324	0,362019	0,649648	n.s.
original_glcm_Correlation	1,135258	0,362055	0,649648	n.s.
wavelet.HHL_glcm_JointAverage	1,128006	0,36596	0,652589	n.s.
wavelet.HHL_glszm_GrayLevelVariance	1,124364	0,367934	0,652589	n.s.
wavelet.LLH_firstorder_Kurtosis	1,122693	0,368842	0,652589	n.s.
wavelet.LHL_glcm_Idn	1,121789	0,369334	0,652589	n.s.
wavelet.LHL_gldm_LowGrayLevelEmphasis	1,120759	0,369896	0,652589	n.s.
wavelet.HLH_glrlm_HighGrayLevelRunEmphasis	1,123747	0,368269	0,652589	n.s.
wavelet.HLH_glszm_ZonePercentage	1,119352	0,370664	0,652589	n.s.
wavelet.HLH_glszm_GrayLevelNonUniformityNormalized	1,121989	0,369225	0,652589	n.s.
wavelet.HHH_firstorder_Maximum	1,126778	0,366624	0,652589	n.s.
log.sigma.1.0.mm.3D_gldm_DependenceEntropy	1,119213	0,37074	0,652589	n.s.
log.sigma.2.0.mm.3D_glcm_JointEnergy	1,128399	0,365747	0,652589	n.s.
original_glcm_ClusterProminence	1,121227	0,369641	0,652589	n.s.
original_glszm_HighGrayLevelZoneEmphasis	1,131184	0,364244	0,652589	n.s.
wavelet.HHL_glcm_Contrast	1,117125	0,371882	0,652608	n.s.

wavelet.LHL_glcm_Idm	1,114782	0,373167	0,652608	n.s.
wavelet.HLH_glcm_DifferenceVariance	1,114428	0,373362	0,652608	n.s.
wavelet.HLH_gldm_HighGrayLevelEmphasis	1,116144	0,37242	0,652608	n.s.
log.sigma.4.0.mm.3D_firstorder_Maximum	1,114247	0,373461	0,652608	n.s.
wavelet.LLH_glrlm_RunPercentage	1,111096	0,375196	0,652797	n.s.
wavelet.HLH_glrlm_ShortRunHighGrayLevelEmphasis	1,111664	0,374882	0,652797	n.s.
wavelet.HLH_glcm_Autocorrelation	1,111174	0,375153	0,652797	n.s.
wavelet.HHH_firstorder_Mean	1,10479	0,375749	0,652816	n.s.
wavelet.HHL_glszm_SizeZoneNonUniformityNormalized	1,107581	0,377139	0,654287	n.s.
wavelet.HHL_glcm_JointEnergy	1,102969	0,379699	0,657462	n.s.
wavelet.HHH_gldm_DependenceEntropy	1,10232	0,380061	0,657462	n.s.
log.sigma.5.0.mm.3D_firstorder_InterquartileRange	1,101073	0,380756	0,657719	n.s.
wavelet.HHL_glrlm_LongRunEmphasis	1,099807	0,381463	0,657996	n.s.
wavelet.HHL_glszm_SmallAreaHighGrayLevelEmphasis	1,095581	0,38383	0,661131	n.s.
wavelet.HHL_glcm_Autocorrelation	1,090792	0,386526	0,66407	n.s.
log.sigma.1.0.mm.3D_glszm_ZoneEntropy	1,090592	0,386639	0,66407	n.s.
wavelet.HHL_firstorder_InterquartileRange	1,086142	0,389157	0,667443	n.s.
wavelet.LHL_glszm_SizeZoneNonUniformityNormalized	1,0826	0,391171	0,669628	n.s.
wavelet.HHH_firstorder_RootMeanSquared	1,076131	0,392099	0,669628	n.s.
original_glcm_DifferenceAverage	1,081835	0,391607	0,669628	n.s.
wavelet.HLL_glszm_SizeZoneNonUniformityNormalized	1,079257	0,393079	0,67035	n.s.
wavelet.HHL_glcm_InverseVariance	1,074931	0,395559	0,670751	n.s.
wavelet.LLH_glszm_GrayLevelVariance	1,072258	0,397097	0,670751	n.s.
wavelet.LHL_glcm_Imc1	1,072054	0,397215	0,670751	n.s.
wavelet.LHL_gldm_SmallDependenceLowGrayLevelEmphasis	1,072156	0,397156	0,670751	n.s.
wavelet.HLL_glcm_Imc2	1,077329	0,394183	0,670751	n.s.
wavelet.LHH_glszm_ZoneVariance	1,071089	0,397771	0,670751	n.s.
wavelet.LLL_glcm_ClusterProminence	1,071654	0,397446	0,670751	n.s.
log.sigma.2.0.mm.3D_firstorder_90Percentile	1,076878	0,394441	0,670751	n.s.
log.sigma.3.0.mm.3D_firstorder_90Percentile	1,069323	0,398791	0,671531	n.s.
wavelet.HHL_glcm_Imc1	1,066656	0,400335	0,67283	n.s.
wavelet.LHH_glcm_ClusterShade	1,059555	0,401798	0,67283	n.s.
log.sigma.1.0.mm.3D_glcm_DifferenceVariance	1,064672	0,401487	0,67283	n.s.
log.sigma.2.0.mm.3D_glszm_ZoneEntropy	1,064244	0,401735	0,67283	n.s.
wavelet.HHL_firstorder_MeanAbsoluteDeviation	1,059656	0,404409	0,675734	n.s.
wavelet.LHH_glszm_LargeAreaEmphasis	1,059237	0,404655	0,675734	n.s.
log.sigma.3.0.mm.3D_glszm_SizeZoneNonUniformityNormalized	1,057088	0,405912	0,676896	n.s.
wavelet.LHH_glrlm_RunVariance	1,05283	0,408413	0,677313	n.s.
wavelet.HLH_glrlm_ShortRunLowGrayLevelEmphasis	1,054564	0,407393	0,677313	n.s.
log.sigma.1.0.mm.3D_glcm_Imc1	1,053361	0,4081	0,677313	n.s.
log.sigma.4.0.mm.3D_firstorder_Skewness	1,054673	0,407329	0,677313	n.s.
wavelet.LLH_glcm_Imc2	1,049346	0,410467	0,678058	n.s.
wavelet.LLH_glszm_SmallAreaLowGrayLevelEmphasis	1,049203	0,410552	0,678058	n.s.
wavelet.LHH_firstorder_Kurtosis	1,046038	0,409838	0,678058	n.s.
original_glszm_LargeAreaHighGrayLevelEmphasis	1,047941	0,411298	0,67836	n.s.
wavelet.HHH_firstorder_Entropy	1,046367	0,41223	0,678967	n.s.

wavelet.LHL_glcm_Contrast	1,043039	0,414207	0,681291	n.s.
wavelet.HHL_glszm_SmallAreaEmphasis	1,039316	0,416425	0,683073	n.s.
log.sigma.2.0.mm.3D_glszm_LargeAreaHighGrayLevelEmphasis	1,010538	0,416145	0,683073	n.s.
log.sigma.2.0.mm.3D_gldm_GrayLevelVariance	1,03788	0,417284	0,68355	n.s.
wavelet.HLH_glrlm_LowGrayLevelRunEmphasis	1,03397	0,419627	0,683754	n.s.
log.sigma.1.0.mm.3D_gldm_DependenceNonUniformityNormalized	1,03424	0,419465	0,683754	n.s.
log.sigma.3.0.mm.3D_glszm_HighGrayLevelZoneEmphasis	1,034218	0,419478	0,683754	n.s.
log.sigma.5.0.mm.3D_glcm_ClusterShade	1,033882	0,41968	0,683754	n.s.
wavelet.HHL_glcm_DifferenceEntropy	1,023704	0,425825	0,690032	n.s.
wavelet.LHL_firstorder_Variance	1,024572	0,425299	0,690032	n.s.
log.sigma.1.0.mm.3D_glcm_JointEnergy	1,026482	0,424142	0,690032	n.s.
log.sigma.3.0.mm.3D_firstorder_Kurtosis	1,024357	0,425429	0,690032	n.s.
wavelet.LHH_gldm_LargeDependenceLowGrayLevelEmphasis	1,017858	0,429385	0,693932	n.s.
wavelet.HHH_gldm_SmallDependenceEmphasis	1,018338	0,429092	0,693932	n.s.
wavelet.LLH_glcm_MaximumProbability	1,015128	0,431054	0,693968	n.s.
wavelet.LHL_glcm_DifferenceVariance	1,014994	0,431136	0,693968	n.s.
wavelet.HHH_gldm_DependenceVariance	1,016168	0,430418	0,693968	n.s.
log.sigma.2.0.mm.3D_firstorder_Uniformity	1,009443	0,434546	0,697524	n.s.
log.sigma.3.0.mm.3D_glrlm_LongRunEmphasis	1,008571	0,435084	0,697524	n.s.
log.sigma.5.0.mm.3D_glszm_LargeAreaLowGrayLevelEmphasis	1,009548	0,434481	0,697524	n.s.
wavelet.HHL_glrlm_RunVariance	1,002104	0,439083	0,699235	n.s.
wavelet.LHH_gldm_SmallDependenceLowGrayLevelEmphasis	1,002041	0,439122	0,699235	n.s.
wavelet.HLH_gldm_LowGrayLevelEmphasis	1,001504	0,439456	0,699235	n.s.
log.sigma.4.0.mm.3D_glszm_ZoneVariance	1,004243	0,437757	0,699235	n.s.
log.sigma.5.0.mm.3D_glszm_SizeZoneNonUniformity	1,00001	0,439635	0,699235	n.s.
original_glcm_Contrast	1,002936	0,438567	0,699235	n.s.
wavelet.LHL_glcm_DifferenceAverage	0,997521	0,441934	0,701964	n.s.
wavelet.HLH_glcm_Idmn	0,995199	0,443383	0,702165	n.s.
wavelet.LLL_glcm_Imc2	0,991722	0,44556	0,702165	n.s.
log.sigma.3.0.mm.3D_firstorder_Energy	0,968016	0,445027	0,702165	n.s.
log.sigma.3.0.mm.3D_firstorder_TotalEnergy	0,968016	0,445027	0,702165	n.s.
log.sigma.3.0.mm.3D_glcm_ClusterProminence	0,996036	0,442861	0,702165	n.s.
log.sigma.3.0.mm.3D_gldm_DependenceNonUniformity	0,993626	0,444366	0,702165	n.s.
wavelet.LHL_glrlm_LongRunLowGrayLevelEmphasis	0,988525	0,447567	0,7027	n.s.
wavelet.LHH_gldm_LargeDependenceHighGrayLevelEmphasis	0,989061	0,44723	0,7027	n.s.
log.sigma.5.0.mm.3D_glrlm_LongRunHighGrayLevelEmphasis	0,988392	0,447651	0,7027	n.s.
log.sigma.3.0.mm.3D_glcm_DifferenceEntropy	0,987178	0,448415	0,702984	n.s.
wavelet.HLH_glcm_Idn	0,984062	0,45038	0,7039	n.s.
wavelet.HLH_glcm_DifferenceEntropy	0,983993	0,450424	0,7039	n.s.
log.sigma.2.0.mm.3D_glcm_SumSquares	0,983472	0,450753	0,7039	n.s.
wavelet.LHH_firstorder_Minimum	0,979772	0,453096	0,705728	n.s.
wavelet.LLL_gldm_DependenceEntropy	0,980094	0,452892	0,705728	n.s.
wavelet.HHL_glcm_DifferenceVariance	0,96837	0,460369	0,708948	n.s.
wavelet.HHL_glcm_ClusterShade	0,961608	0,464719	0,708948	n.s.
wavelet.HHL_glszm_SmallAreaLowGrayLevelEmphasis	0,970061	0,459285	0,708948	n.s.
wavelet.LHL_glcm_Idmn	0,970511	0,458997	0,708948	n.s.

wavelet.LHL_glszm_LargeAreaHighGrayLevelEmphasis	0,969716	0,459506	0,708948	n.s.
wavelet.LHH_firstorder_Range	0,966269	0,461717	0,708948	n.s.
wavelet.LHH_glszm_ZoneEntropy	0,962262	0,464297	0,708948	n.s.
wavelet.HHH_glrlm_RunLengthNonUniformityNormalized	0,961643	0,464697	0,708948	n.s.
wavelet.LLL_glszm_SizeZoneNonUniformityNormalized	0,967983	0,460617	0,708948	n.s.
wavelet.LLL_gldm_LargeDependenceLowGrayLevelEmphasis	0,965316	0,46233	0,708948	n.s.
log.sigma.2.0.mm.3D_firstorder_Variance	0,965219	0,462392	0,708948	n.s.
log.sigma.2.0.mm.3D_glcm_MaximumProbability	0,972527	0,457708	0,708948	n.s.
log.sigma.2.0.mm.3D_gldm_LowGrayLevelEmphasis	0,970664	0,458899	0,708948	n.s.
log.sigma.3.0.mm.3D_glcm_ClusterShade	0,960904	0,465174	0,708948	n.s.
log.sigma.3.0.mm.3D_glszm_ZonePercentage	0,961405	0,46485	0,708948	n.s.
log.sigma.4.0.mm.3D_glszm_SmallAreaLowGrayLevelEmphasis	0,963468	0,463519	0,708948	n.s.
log.sigma.5.0.mm.3D_glszm_GrayLevelNonUniformity	0,969698	0,459518	0,708948	n.s.
wavelet.LHH_glrlm_LongRunLowGrayLevelEmphasis	0,958415	0,466783	0,710502	n.s.
log.sigma.3.0.mm.3D_glszm_SmallAreaHighGrayLevelEmphasis	0,955595	0,468611	0,712384	n.s.
wavelet.HHL_glrlm_LongRunHighGrayLevelEmphasis	0,954654	0,469222	0,712413	n.s.
wavelet.LHH_gldm_GrayLevelNonUniformity	0,951937	0,470989	0,714195	n.s.
wavelet.HHL_gldm_GrayLevelNonUniformity	0,949101	0,472839	0,716098	n.s.
log.sigma.4.0.mm.3D_glszm_SizeZoneNonUniformity	0,946101	0,4748	0,718165	n.s.
wavelet.LLH_firstorder_InterquartileRange	0,944459	0,475876	0,718889	n.s.
wavelet.LHH_gldm_DependenceNonUniformityNormalized	0,942691	0,477036	0,719739	n.s.
log.sigma.5.0.mm.3D_glszm_LargeAreaEmphasis	0,921691	0,47782	0,72002	n.s.
log.sigma.1.0.mm.3D_firstorder_Maximum	0,940122	0,478725	0,720482	n.s.
wavelet.HHH_glrlm_ShortRunEmphasis	0,938097	0,480059	0,720687	n.s.
log.sigma.2.0.mm.3D_glcm_ClusterShade	0,938526	0,479777	0,720687	n.s.
wavelet.LHL_firstorder_InterquartileRange	0,934985	0,482114	0,722838	n.s.
log.sigma.3.0.mm.3D_glcm_DifferenceVariance	0,934111	0,482693	0,722838	n.s.
wavelet.HHH_glcm_ClusterShade	0,924544	0,487268	0,727879	n.s.
log.sigma.3.0.mm.3D_glrlm_ShortRunEmphasis	0,927287	0,487223	0,727879	n.s.
log.sigma.1.0.mm.3D_glcm_Idn	0,924231	0,48926	0,728194	n.s.
log.sigma.2.0.mm.3D_firstorder_MeanAbsoluteDeviation	0,924722	0,488933	0,728194	n.s.
log.sigma.2.0.mm.3D_glrlm_LongRunHighGrayLevelEmphasis	0,924182	0,489293	0,728194	n.s.
wavelet.HHH_glcm_SumSquares	0,923041	0,490055	0,728427	n.s.
wavelet.LLH_firstorder_RobustMeanAbsoluteDeviation	0,92204	0,490724	0,728523	n.s.
log.sigma.5.0.mm.3D_glrlm_RunVariance	0,920791	0,49156	0,728865	n.s.
log.sigma.2.0.mm.3D_glcm_ClusterTendency	0,919412	0,492484	0,729337	n.s.
wavelet.LLH_glcm_Imc1	0,915518	0,495099	0,730513	n.s.
wavelet.HLH_glrlm_LongRunLowGrayLevelEmphasis	0,917119	0,494022	0,730513	n.s.
wavelet.HLH_gldm_LargeDependenceLowGrayLevelEmphasis	0,915531	0,49509	0,730513	n.s.
wavelet.LHL_glrlm_RunEntropy	0,911675	0,497688	0,733435	n.s.
wavelet.LHL_glcm_JointAverage	0,907667	0,500397	0,734729	n.s.
wavelet.LHL_glcm_SumSquares	0,909047	0,499463	0,734729	n.s.
wavelet.LLL_gldm_DependenceNonUniformityNormalized	0,907953	0,500203	0,734729	n.s.
wavelet.LLL_gldm_SmallDependenceLowGrayLevelEmphasis	0,905325	0,501984	0,736162	n.s.
wavelet.LHL_glrlm_ShortRunHighGrayLevelEmphasis	0,904018	0,502872	0,736566	n.s.
log.sigma.3.0.mm.3D_glszm_ZoneEntropy	0,90038	0,505346	0,738832	n.s.

original_glcm_Idmn	0,89994	0,505646	0,738832	n.s.
wavelet.LHH_glrlm_LongRunHighGrayLevelEmphasis	0,894514	0,509351	0,739882	n.s.
wavelet.HHH_glrlm_RunLengthNonUniformity	0,894425	0,509412	0,739882	n.s.
wavelet.HHH_glrlm_RunEntropy	0,894389	0,509437	0,739882	n.s.
wavelet.HHH_glszm_HighGrayLevelZoneEmphasis	0,896787	0,507797	0,739882	n.s.
log.sigma.3.0.mm.3D_gldm_DependenceNonUniformityNormalized	0,894701	0,509223	0,739882	n.s.
wavelet.HHL_glcm_MaximumProbability	0,891657	0,511309	0,74096	n.s.
wavelet.LHL_glrlm_LongRunHighGrayLevelEmphasis	0,890613	0,512026	0,74096	n.s.
log.sigma.2.0.mm.3D_glszm_HighGrayLevelZoneEmphasis	0,891451	0,511451	0,74096	n.s.
wavelet.LHL_firstorder_Entropy	0,881215	0,518503	0,743873	n.s.
wavelet.LHL_glrlm_HighGrayLevelRunEmphasis	0,880523	0,518981	0,743873	n.s.
wavelet.LHH_gldm_LowGrayLevelEmphasis	0,882976	0,517285	0,743873	n.s.
wavelet.HLH_glszm_LargeAreaLowGrayLevelEmphasis	0,881519	0,518293	0,743873	n.s.
log.sigma.1.0.mm.3D_glrlm_ShortRunEmphasis	0,884386	0,516312	0,743873	n.s.
log.sigma.2.0.mm.3D_glrlm_RunEntropy	0,886349	0,514959	0,743873	n.s.
log.sigma.3.0.mm.3D_gldm_SmallDependenceHighGrayLevelEmphasis	0,885378	0,515628	0,743873	n.s.
log.sigma.5.0.mm.3D_glszm_ZoneEntropy	0,882686	0,517486	0,743873	n.s.
wavelet.HHH_gldm_GrayLevelNonUniformity	0,878711	0,520236	0,744786	n.s.
wavelet.LHL_firstorder_MeanAbsoluteDeviation	0,875693	0,52233	0,746667	n.s.
wavelet.LLL_glrlm_LongRunLowGrayLevelEmphasis	0,875031	0,522791	0,746667	n.s.
log.sigma.2.0.mm.3D_gldm_SmallDependenceLowGrayLevelEmphasis	0,868993	0,526997	0,751783	n.s.
wavelet.HLL_glcm_Imc1	0,86745	0,528075	0,752429	n.s.
original_glszm_ZoneEntropy	0,865711	0,529292	0,753271	n.s.
wavelet.LLH_firstorder_Median	0,86103	0,532574	0,754288	n.s.
wavelet.LLH_gldm_GrayLevelNonUniformity	0,863716	0,530689	0,754288	n.s.
wavelet.HHH_glcm_MaximumProbability	0,861551	0,532208	0,754288	n.s.
wavelet.HHH_glszm_SizeZoneNonUniformity	0,86149	0,532251	0,754288	n.s.
log.sigma.3.0.mm.3D_firstorder_10Percentile	0,860226	0,533139	0,754288	n.s.
wavelet.LLH_firstorder_90Percentile	0,855726	0,536307	0,756991	n.s.
wavelet.LHL_gldm_HighGrayLevelEmphasis	0,855893	0,536189	0,756991	n.s.
wavelet.HHH_glcm_DifferenceVariance	0,854665	0,537055	0,75716	n.s.
wavelet.LHH_glrlm_LowGrayLevelRunEmphasis	0,851678	0,539166	0,759246	n.s.
wavelet.LHL_firstorder_RobustMeanAbsoluteDeviation	0,850338	0,540114	0,759694	n.s.
wavelet.HHH_gldm_LargeDependenceEmphasis	0,845652	0,543437	0,763076	n.s.
log.sigma.3.0.mm.3D_glszm_GrayLevelNonUniformity	0,844268	0,54442	0,763076	n.s.
log.sigma.4.0.mm.3D_glszm_LargeAreaHighGrayLevelEmphasis	0,833325	0,543819	0,763076	n.s.
wavelet.HHL_firstorder_RobustMeanAbsoluteDeviation	0,84123	0,546583	0,765216	n.s.
wavelet.LLL_glrlm_LowGrayLevelRunEmphasis	0,836843	0,549713	0,767812	n.s.
log.sigma.3.0.mm.3D_glrlm_GrayLevelNonUniformity	0,837419	0,549301	0,767812	n.s.
wavelet.HHL_glszm_GrayLevelNonUniformityNormalized	0,830668	0,554135	0,769583	n.s.
wavelet.HHH_glszm_SmallAreaEmphasis	0,830687	0,554121	0,769583	n.s.
wavelet.LLL_glszm_SmallAreaEmphasis	0,83061	0,554176	0,769583	n.s.
log.sigma.2.0.mm.3D_gldm_LargeDependenceHighGrayLevelEmphasis	0,833084	0,552402	0,769583	n.s.
log.sigma.5.0.mm.3D_glszm_LowGrayLevelZoneEmphasis	0,83279	0,552613	0,769583	n.s.
wavelet.LHH_glszm_LargeAreaHighGrayLevelEmphasis	0,827497	0,556413	0,771637	n.s.
wavelet.HHH_gldm_GrayLevelVariance	0,822397	0,560086	0,771637	n.s.

wavelet.LLL_glrlm_ShortRunLowGrayLevelEmphasis	0,82232	0,560142	0,771637	n.s.
wavelet.LLL_gldm_LowGrayLevelEmphasis	0,823449	0,559327	0,771637	n.s.
log.sigma.1.0.mm.3D_glrlm_RunLengthNonUniformityNormalized	0,823107	0,559574	0,771637	n.s.
log.sigma.2.0.mm.3D_firstorder_RootMeanSquared	0,823313	0,559425	0,771637	n.s.
log.sigma.5.0.mm.3D_glszm_SmallAreaLowGrayLevelEmphasis	0,822652	0,558831	0,771637	n.s.
log.sigma.3.0.mm.3D_gldm_LargeDependenceEmphasis	0,818925	0,562594	0,774129	n.s.
wavelet.HHL_firstorder_10Percentile	0,8123	0,567395	0,775743	n.s.
wavelet.HHL_glcm_Imc2	0,814179	0,566031	0,775743	n.s.
wavelet.LLH_firstorder_Skewness	0,797448	0,578228	0,775743	n.s.
wavelet.LLH_glrlm_ShortRunEmphasis	0,815221	0,565276	0,775743	n.s.
wavelet.LLH_glcm_Id	0,800199	0,576214	0,775743	n.s.
wavelet.LLH_glcm_Idmn	0,800199	0,576214	0,775743	n.s.
wavelet.LLH_glszm_LowGrayLevelZoneEmphasis	0,807498	0,570887	0,775743	n.s.
wavelet.LHL_glcm_Autocorrelation	0,800958	0,575659	0,775743	n.s.
wavelet.LHL_glszm_SizeZoneNonUniformity	0,806827	0,571375	0,775743	n.s.
wavelet.LHL_glszm_ZoneEntropy	0,799723	0,576562	0,775743	n.s.
wavelet.LHH_glrlm_ShortRunLowGrayLevelEmphasis	0,79696	0,578586	0,775743	n.s.
wavelet.HLH_glszm_SmallAreaLowGrayLevelEmphasis	0,803437	0,573848	0,775743	n.s.
wavelet.HHH_glszm_SmallAreaHighGrayLevelEmphasis	0,809898	0,56914	0,775743	n.s.
wavelet.LLL_glszm_SmallAreaLowGrayLevelEmphasis	0,813688	0,566387	0,775743	n.s.
log.sigma.1.0.mm.3D_glcm_Contrast	0,797608	0,578111	0,775743	n.s.
log.sigma.2.0.mm.3D_firstorder_Mean	0,806519	0,5716	0,775743	n.s.
log.sigma.2.0.mm.3D_glrlm_LowGrayLevelRunEmphasis	0,805792	0,572129	0,775743	n.s.
log.sigma.3.0.mm.3D_glcm_Id	0,799272	0,576892	0,775743	n.s.
log.sigma.3.0.mm.3D_glcm_DifferenceAverage	0,799446	0,576765	0,775743	n.s.
log.sigma.3.0.mm.3D_glcm_InverseVariance	0,798989	0,577099	0,775743	n.s.
log.sigma.3.0.mm.3D_glcm_Contrast	0,799968	0,576383	0,775743	n.s.
log.sigma.3.0.mm.3D_glcm_Idm	0,799342	0,576841	0,775743	n.s.
log.sigma.5.0.mm.3D_gldm_GrayLevelNonUniformity	0,797101	0,578482	0,775743	n.s.
wavelet.LHL_glrlm_GrayLevelNonUniformityNormalized	0,790802	0,583106	0,777475	n.s.
wavelet.LHL_glcm_ClusterTendency	0,791324	0,582723	0,777475	n.s.
wavelet.HLL_firstorder_Median	0,792146	0,582118	0,777475	n.s.
wavelet.HHH_gldm_DependenceNonUniformity	0,793504	0,581121	0,777475	n.s.
log.sigma.3.0.mm.3D_glrlm_RunPercentage	0,791851	0,582335	0,777475	n.s.
wavelet.LLH_glszm_SizeZoneNonUniformityNormalized	0,785252	0,587194	0,779182	n.s.
wavelet.LHH_glcm_ClusterProminence	0,784672	0,587622	0,779182	n.s.
wavelet.HLH_glcm_MaximumProbability	0,786934	0,585954	0,779182	n.s.
log.sigma.2.0.mm.3D_glrlm_GrayLevelNonUniformity	0,786362	0,586376	0,779182	n.s.
log.sigma.2.0.mm.3D_glcm_ClusterProminence	0,786327	0,586401	0,779182	n.s.
wavelet.LHH_glrlm_RunLengthNonUniformity	0,781791	0,58975	0,779725	n.s.
wavelet.LHH_glcm_JointAverage	0,781487	0,589974	0,779725	n.s.
wavelet.LHH_glszm_GrayLevelNonUniformityNormalized	0,78217	0,58947	0,779725	n.s.
wavelet.LLH_glszm_GrayLevelNonUniformity	0,77986	0,591178	0,780458	n.s.
wavelet.LHH_glcm_Imc2	0,776644	0,593559	0,781888	n.s.
log.sigma.1.0.mm.3D_glrlm_RunPercentage	0,776993	0,593301	0,781888	n.s.
wavelet.LLH_glszm_GrayLevelNonUniformityNormalized	0,77093	0,5978	0,784043	n.s.

wavelet.HHH_glcm_ClusterProminence	0,771682	0,597242	0,784043	n.s.
log.sigma.1.0.mm.3D_gldm_DependenceVariance	0,773364	0,595992	0,784043	n.s.
original_glrlm_ShortRunHighGrayLevelEmphasis	0,771917	0,597067	0,784043	n.s.
log.sigma.1.0.mm.3D_firstorder_10Percentile	0,768683	0,599471	0,785379	n.s.
wavelet.LHH_glcm_MaximumProbability	0,766871	0,60082	0,786291	n.s.
log.sigma.4.0.mm.3D_glszm_LargeAreaEmphasis	0,765185	0,602077	0,78708	n.s.
wavelet.HHL_gldm_LargeDependenceHighGrayLevelEmphasis	0,760033	0,605922	0,791247	n.s.
wavelet.LLH_firstorder_Range	0,757369	0,607914	0,792989	n.s.
wavelet.LLH_glcm_JointEnergy	0,749612	0,613728	0,793115	n.s.
wavelet.LLH_glcm_ClusterProminence	0,751537	0,612284	0,793115	n.s.
wavelet.LLH_glszm_LargeAreaHighGrayLevelEmphasis	0,750273	0,613232	0,793115	n.s.
wavelet.LHL_glcm_DifferenceEntropy	0,751709	0,612155	0,793115	n.s.
wavelet.LHH_glcm_InverseVariance	0,748303	0,614711	0,793115	n.s.
wavelet.LHH_glcm_Autocorrelation	0,748593	0,614493	0,793115	n.s.
wavelet.LHH_glszm_SizeZoneNonUniformity	0,752524	0,611543	0,793115	n.s.
wavelet.HLH_firstorder_Range	0,747577	0,615257	0,793115	n.s.
log.sigma.1.0.mm.3D_glcm_DifferenceAverage	0,75546	0,609343	0,793115	n.s.
log.sigma.4.0.mm.3D_gldm_SmallDependenceEmphasis	0,755453	0,609348	0,793115	n.s.
log.sigma.5.0.mm.3D_glrlm_ShortRunLowGrayLevelEmphasis	0,753908	0,610506	0,793115	n.s.
original_gldm_DependenceEntropy	0,745717	0,616655	0,794067	n.s.
wavelet.LHH_glszm_GrayLevelVariance	0,743236	0,618522	0,794772	n.s.
wavelet.LHH_gldm_HighGrayLevelEmphasis	0,743253	0,61851	0,794772	n.s.
wavelet.HHH_firstorder_Energy	0,734714	0,621604	0,796758	n.s.
wavelet.HHH_firstorder_TotalEnergy	0,734714	0,621604	0,796758	n.s.
wavelet.LLL_glcm_Idmn	0,738551	0,622053	0,796758	n.s.
wavelet.LHL_gldm_LargeDependenceLowGrayLevelEmphasis	0,735905	0,624051	0,798467	n.s.
log.sigma.5.0.mm.3D_glszm_ZoneVariance	0,732498	0,626626	0,80091	n.s.
wavelet.LHH_gldm_DependenceVariance	0,731374	0,627476	0,801146	n.s.
wavelet.HLL_glszm_SizeZoneNonUniformity	0,724351	0,632796	0,806539	n.s.
wavelet.LHH_glrlm_LongRunEmphasis	0,723185	0,633681	0,806539	n.s.
log.sigma.2.0.mm.3D_firstorder_RobustMeanAbsoluteDeviation	0,723148	0,633709	0,806539	n.s.
wavelet.LHH_glcm_Idmn	0,722215	0,634417	0,806588	n.s.
log.sigma.3.0.mm.3D_glrlm_RunVariance	0,719815	0,63624	0,808051	n.s.
wavelet.LHH_glrlm_HighGrayLevelRunEmphasis	0,71814	0,637513	0,808815	n.s.
wavelet.LHH_gldm_DependenceEntropy	0,715347	0,639637	0,809803	n.s.
original_glrlm_HighGrayLevelRunEmphasis	0,716228	0,638966	0,809803	n.s.
original_gldm_HighGrayLevelEmphasis	0,711505	0,642563	0,812653	n.s.
log.sigma.2.0.mm.3D_glszm_ZonePercentage	0,702975	0,64907	0,820021	n.s.
wavelet.LLH_glcm_Idm	0,701777	0,649985	0,820138	n.s.
wavelet.LHL_firstorder_Uniformity	0,696749	0,65383	0,820138	n.s.
wavelet.LHH_firstorder_Mean	0,69751	0,653247	0,820138	n.s.
wavelet.LLL_glcm_Idn	0,693058	0,656655	0,820138	n.s.
log.sigma.1.0.mm.3D_glcm_Idm	0,694104	0,655854	0,820138	n.s.
log.sigma.1.0.mm.3D_gldm_LargeDependenceEmphasis	0,695971	0,654425	0,820138	n.s.
log.sigma.1.0.mm.3D_gldm_SmallDependenceLowGrayLevelEmphasis	0,693348	0,656433	0,820138	n.s.
log.sigma.2.0.mm.3D_glrlm_ShortRunHighGrayLevelEmphasis	0,693499	0,656317	0,820138	n.s.

log.sigma.4.0.mm.3D_glszm_LargeAreaLowGrayLevelEmphasis	0,700097	0,651269	0,820138	n.s.
log.sigma.4.0.mm.3D_gldm_SmallDependenceHighGrayLevelEmphasis	0,694563	0,655503	0,820138	n.s.
original_glszm_SmallAreaEmphasis	0,694471	0,655573	0,820138	n.s.
original_glszm_SizeZoneNonUniformityNormalized	0,692117	0,657376	0,820188	n.s.
log.sigma.1.0.mm.3D_glcm_Id	0,690412	0,658682	0,820966	n.s.
wavelet.LLH_firstorder_MeanAbsoluteDeviation	0,687025	0,66128	0,821115	n.s.
wavelet.LLH_glrlm_RunLengthNonUniformityNormalized	0,679592	0,666985	0,821115	n.s.
wavelet.LLH_gldm_SmallDependenceEmphasis	0,679601	0,666978	0,821115	n.s.
wavelet.LLH_gldm_DependenceEntropy	0,6838	0,663754	0,821115	n.s.
wavelet.LHH_firstorder_Energy	0,681713	0,66446	0,821115	n.s.
wavelet.LHH_firstorder_RootMeanSquared	0,686956	0,661332	0,821115	n.s.
wavelet.LHH_firstorder_TotalEnergy	0,681713	0,66446	0,821115	n.s.
wavelet.HLH_glszm_GrayLevelVariance	0,681725	0,665347	0,821115	n.s.
log.sigma.2.0.mm.3D_firstorder_Median	0,680761	0,666088	0,821115	n.s.
log.sigma.4.0.mm.3D_firstorder_Median	0,680003	0,666669	0,821115	n.s.
original_glcm_Autocorrelation	0,684007	0,663595	0,821115	n.s.
original_gldm_SmallDependenceEmphasis	0,681787	0,665299	0,821115	n.s.
log.sigma.1.0.mm.3D_firstorder_Kurtosis	0,677461	0,668497	0,822135	n.s.
wavelet.LHH_glcm_ClusterTendency	0,673242	0,671867	0,825436	n.s.
original_gldm_LargeDependenceHighGrayLevelEmphasis	0,671448	0,673247	0,826288	n.s.
wavelet.HLH_glszm_LargeAreaEmphasis	0,665867	0,677542	0,828225	n.s.
wavelet.HHH_glrlm_GrayLevelNonUniformityNormalized	0,66493	0,678264	0,828225	n.s.
log.sigma.3.0.mm.3D_glrlm_RunLengthNonUniformityNormalized	0,667713	0,676121	0,828225	n.s.
log.sigma.3.0.mm.3D_glszm_GrayLevelNonUniformityNormalized	0,665103	0,678131	0,828225	n.s.
original_gldm_SmallDependenceLowGrayLevelEmphasis	0,666651	0,676939	0,828225	n.s.
wavelet.LLH_firstorder_Variance	0,657622	0,683896	0,829215	n.s.
wavelet.HLL_glszm_SmallAreaHighGrayLevelEmphasis	0,659105	0,682752	0,829215	n.s.
wavelet.LHH_glrlm_ShortRunHighGrayLevelEmphasis	0,660757	0,681479	0,829215	n.s.
wavelet.LHH_glszm_HighGrayLevelZoneEmphasis	0,658918	0,682897	0,829215	n.s.
wavelet.HHH_glcm_DifferenceEntropy	0,659851	0,682177	0,829215	n.s.
wavelet.HHH_glszm_LowGrayLevelZoneEmphasis	0,658555	0,683176	0,829215	n.s.
wavelet.LLL_glcm_ClusterShade	0,658211	0,683441	0,829215	n.s.
wavelet.HHL_glcm_Idmn	0,654889	0,686003	0,830098	n.s.
log.sigma.2.0.mm.3D_firstorder_Minimum	0,655443	0,685576	0,830098	n.s.
wavelet.LLH_glrlm_RunEntropy	0,651255	0,688805	0,830178	n.s.
wavelet.LLH_gldm_SmallDependenceHighGrayLevelEmphasis	0,649739	0,689975	0,830178	n.s.
wavelet.LHL_glcm_InverseVariance	0,652057	0,688187	0,830178	n.s.
wavelet.LHH_glcm_Correlation	0,649439	0,690206	0,830178	n.s.
wavelet.HLH_firstorder_Maximum	0,650388	0,689474	0,830178	n.s.
log.sigma.2.0.mm.3D_glcm_JointAverage	0,652936	0,687509	0,830178	n.s.
wavelet.LHH_glrlm_GrayLevelNonUniformity	0,648344	0,691051	0,830365	n.s.
wavelet.HLL_glcm_ClusterShade	0,645381	0,693338	0,832282	n.s.
wavelet.HHL_glrlm_RunLengthNonUniformity	0,642737	0,695378	0,8339	n.s.
wavelet.HLH_gldm_DependenceEntropy	0,63919	0,698116	0,834095	n.s.
log.sigma.1.0.mm.3D_glszm_GrayLevelNonUniformity	0,641528	0,696311	0,834095	n.s.
log.sigma.4.0.mm.3D_glrlm_GrayLevelNonUniformity	0,638937	0,698312	0,834095	n.s.

original_glcm_Idn	0,640292	0,697265	0,834095	n.s.
original_glcm_Imc1	0,637272	0,699597	0,834802	n.s.
wavelet.LHL_firstorder_Energy	0,630344	0,706141	0,838971	n.s.
wavelet.LHL_firstorder_TotalEnergy	0,630344	0,706141	0,838971	n.s.
wavelet.LHH_firstorder_InterquartileRange	0,628278	0,70654	0,838971	n.s.
wavelet.HHH_firstorder_Skewness	0,627977	0,706917	0,838971	n.s.
log.sigma.2.0.mm.3D_firstorder_10Percentile	0,631168	0,704309	0,838971	n.s.
log.sigma.2.0.mm.3D_gldm_HighGrayLevelEmphasis	0,62733	0,707272	0,838971	n.s.
wavelet.HHL_glcm_Idn	0,625138	0,708964	0,839975	n.s.
wavelet.LLH_gldm_SmallDependenceLowGrayLevelEmphasis	0,623523	0,710211	0,839975	n.s.
wavelet.LHL_firstorder_Median	0,623711	0,710066	0,839975	n.s.
wavelet.HLH_firstorder_Median	0,618994	0,713707	0,843281	n.s.
wavelet.HHL_gldm_DependenceVariance	0,616928	0,715302	0,844337	n.s.
wavelet.HHH_glrlm_RunPercentage	0,613926	0,717618	0,845589	n.s.
log.sigma.2.0.mm.3D_glcm_Idn	0,613732	0,717767	0,845589	n.s.
wavelet.LLL_glcm_Correlation	0,607036	0,722931	0,850769	n.s.
log.sigma.5.0.mm.3D_glszm_SmallAreaHighGrayLevelEmphasis	0,606198	0,723578	0,850769	n.s.
log.sigma.2.0.mm.3D_glrlm_HighGrayLevelRunEmphasis	0,605004	0,724498	0,851021	n.s.
wavelet.LLH_glcm_ClusterTendency	0,604061	0,725225	0,851044	n.s.
wavelet.LHL_firstorder_Mean	0,601038	0,727554	0,852945	n.s.
wavelet.HHH_glrlm_GrayLevelNonUniformity	0,593001	0,733742	0,859363	n.s.
log.sigma.2.0.mm.3D_glcm_Autocorrelation	0,591918	0,734575	0,859502	n.s.
wavelet.LLH_glcm_SumSquares	0,586931	0,738409	0,862313	n.s.
wavelet.LHH_gldm_DependenceNonUniformity	0,587016	0,738343	0,862313	n.s.
original_glrlm_LongRunHighGrayLevelEmphasis	0,584613	0,74019	0,863554	n.s.
log.sigma.2.0.mm.3D_glrlm_ShortRunLowGrayLevelEmphasis	0,581625	0,742484	0,865392	n.s.
wavelet.LLH_firstorder_10Percentile	0,576278	0,746584	0,86933	n.s.
wavelet.LLH_glrlm_GrayLevelNonUniformity	0,574352	0,748059	0,869394	n.s.
wavelet.LLH_glcm_SumEntropy	0,564584	0,75553	0,869394	n.s.
wavelet.LLH_glcm_DifferenceAverage	0,569418	0,751836	0,869394	n.s.
wavelet.LLH_glszm_HighGrayLevelZoneEmphasis	0,572149	0,749746	0,869394	n.s.
wavelet.LHL_glcm_JointEnergy	0,570602	0,75093	0,869394	n.s.
wavelet.LHH_glcm_Idn	0,568358	0,752646	0,869394	n.s.
wavelet.HLH_glcm_ClusterShade	0,566032	0,754844	0,869394	n.s.
wavelet.HHH_glcm_JointEntropy	0,563934	0,756026	0,869394	n.s.
wavelet.LLL_glcm_Imc1	0,566548	0,75403	0,869394	n.s.
log.sigma.1.0.mm.3D_firstorder_Energy	0,568515	0,755758	0,869394	n.s.
log.sigma.1.0.mm.3D_firstorder_TotalEnergy	0,568515	0,755758	0,869394	n.s.
log.sigma.2.0.mm.3D_glszm_SizeZoneNonUniformityNormalized	0,564619	0,755503	0,869394	n.s.
log.sigma.3.0.mm.3D_glszm_SizeZoneNonUniformity	0,567624	0,753207	0,869394	n.s.
wavelet.LLH_firstorder_Maximum	0,556241	0,761891	0,869873	n.s.
wavelet.LHL_firstorder_10Percentile	0,559523	0,759391	0,869873	n.s.
wavelet.LHH_glszm_ZonePercentage	0,556993	0,761318	0,869873	n.s.
log.sigma.2.0.mm.3D_firstorder_InterquartileRange	0,562151	0,757387	0,869873	n.s.
log.sigma.2.0.mm.3D_glcm_Idmn	0,556496	0,761697	0,869873	n.s.
log.sigma.5.0.mm.3D_gldm_DependenceNonUniformity	0,555805	0,762223	0,869873	n.s.

original_glszm_ZonePercentage	0,555849	0,76219	0,869873	n.s.
original_gldm_DependenceNonUniformityNormalized	0,560454	0,758681	0,869873	n.s.
wavelet.LLH_glszm_LargeAreaLowGrayLevelEmphasis	0,553048	0,76432	0,87144	n.s.
wavelet.LHL_glcm_JointEntropy	0,548535	0,767748	0,872187	n.s.
wavelet.LHL_glcm_SumEntropy	0,550236	0,766457	0,872187	n.s.
wavelet.LHH_glszm_SmallAreaEmphasis	0,550144	0,766527	0,872187	n.s.
wavelet.HHH_gldm_SmallDependenceLowGrayLevelEmphasis	0,548371	0,767872	0,872187	n.s.
wavelet.LLH_firstorder_Minimum	0,540505	0,77383	0,874197	n.s.
wavelet.LLH_glrlm_ShortRunLowGrayLevelEmphasis	0,543148	0,77183	0,874197	n.s.
wavelet.LLH_glcm_JointEntropy	0,539729	0,774417	0,874197	n.s.
wavelet.LLH_glcm_Contrast	0,538798	0,77512	0,874197	n.s.
wavelet.LHH_firstorder_RobustMeanAbsoluteDeviation	0,536437	0,776902	0,874197	n.s.
wavelet.HLH_glrlm_RunEntropy	0,536901	0,776552	0,874197	n.s.
log.sigma.1.0.mm.3D_glszm_SmallAreaLowGrayLevelEmphasis	0,536436	0,776903	0,874197	n.s.
log.sigma.4.0.mm.3D_glszm_ZonePercentage	0,542091	0,77263	0,874197	n.s.
log.sigma.5.0.mm.3D_firstorder_Maximum	0,543925	0,771243	0,874197	n.s.
original_gldm_LargeDependenceLowGrayLevelEmphasis	0,541103	0,773378	0,874197	n.s.
wavelet.HHL_firstorder_RootMeanSquared	0,531298	0,780774	0,877321	n.s.
wavelet.LLH_gldm_LargeDependenceHighGrayLevelEmphasis	0,529503	0,782124	0,877321	n.s.
wavelet.LHH_firstorder_Variance	0,529282	0,78229	0,877321	n.s.
wavelet.HHH_firstorder_Range	0,528878	0,782593	0,877321	n.s.
wavelet.LHL_firstorder_RootMeanSquared	0,526174	0,784624	0,878778	n.s.
wavelet.LLH_glrlm_LowGrayLevelRunEmphasis	0,524052	0,786215	0,879742	n.s.
log.sigma.5.0.mm.3D_firstorder_Kurtosis	0,518762	0,790171	0,883349	n.s.
original_glcm_JointAverage	0,516821	0,79162	0,884147	n.s.
wavelet.LLH_gldm_LargeDependenceLowGrayLevelEmphasis	0,507128	0,798827	0,888077	n.s.
wavelet.HLH_gldm_DependenceNonUniformityNormalized	0,507652	0,798439	0,888077	n.s.
wavelet.HHH_firstorder_Uniformity	0,508068	0,79813	0,888077	n.s.
log.sigma.4.0.mm.3D_firstorder_Energy	0,515452	0,797124	0,888077	n.s.
log.sigma.4.0.mm.3D_firstorder_TotalEnergy	0,515452	0,797124	0,888077	n.s.
wavelet.LLH_gldm_LowGrayLevelEmphasis	0,503538	0,801484	0,890209	n.s.
wavelet.LHH_firstorder_MeanAbsoluteDeviation	0,501917	0,802681	0,890717	n.s.
wavelet.LLH_glrlm_LongRunLowGrayLevelEmphasis	0,500088	0,80403	0,890742	n.s.
wavelet.LHH_glcm_SumSquares	0,49988	0,804183	0,890742	n.s.
wavelet.HHL_gldm_DependenceNonUniformity	0,496671	0,806546	0,892277	n.s.
wavelet.HHL_gldm_SmallDependenceLowGrayLevelEmphasis	0,493762	0,808682	0,892277	n.s.
wavelet.HLL_firstorder_Maximum	0,494868	0,80787	0,892277	n.s.
wavelet.LLL_firstorder_Kurtosis	0,494418	0,808837	0,892277	n.s.
original_firstorder_Kurtosis	0,493824	0,809274	0,892277	n.s.
wavelet.HHL_firstorder_Mean	0,491435	0,811029	0,893015	n.s.
wavelet.HHH_glrlm_RunVariance	0,490012	0,811428	0,893015	n.s.
wavelet.LHH_glszm_SmallAreaHighGrayLevelEmphasis	0,486011	0,814348	0,89541	n.s.
log.sigma.3.0.mm.3D_glcm_Idn	0,480877	0,81808	0,898694	n.s.
wavelet.HHL_firstorder_Skewness	0,470673	0,825445	0,900263	n.s.
wavelet.HHL_glszm_LargeAreaHighGrayLevelEmphasis	0,476101	0,821536	0,900263	n.s.
wavelet.LLH_glszm_ZoneVariance	0,472259	0,824305	0,900263	n.s.

wavelet.LHL_firstorder_90Percentile	0,473849	0,82316	0,900263	n.s.
wavelet.HLL_firstorder_Skewness	0,471651	0,824743	0,900263	n.s.
wavelet.HHH_glcm_SumEntropy	0,47061	0,82549	0,900263	n.s.
log.sigma.2.0.mm.3D_glszm_LowGrayLevelZoneEmphasis	0,473007	0,823767	0,900263	n.s.
log.sigma.4.0.mm.3D_glcm_ClusterShade	0,475895	0,821685	0,900263	n.s.
wavelet.HHH_glcm_Contrast	0,46103	0,832337	0,906908	n.s.
log.sigma.4.0.mm.3D_firstorder_Mean	0,459801	0,83321	0,907039	n.s.
wavelet.LHL_glcm_Correlation	0,455268	0,836421	0,90718	n.s.
wavelet.HHH_glcm_InverseVariance	0,454296	0,837107	0,90718	n.s.
log.sigma.2.0.mm.3D_glszm_SmallAreaLowGrayLevelEmphasis	0,456405	0,835617	0,90718	n.s.
log.sigma.3.0.mm.3D_gldm_SmallDependenceEmphasis	0,458221	0,834331	0,90718	n.s.
log.sigma.4.0.mm.3D_firstorder_Minimum	0,454566	0,836916	0,90718	n.s.
log.sigma.4.0.mm.3D_firstorder_RootMeanSquared	0,452098	0,838656	0,908041	n.s.
wavelet.LLH_glcm_DifferenceVariance	0,443182	0,844898	0,913912	n.s.
wavelet.HHH_glcm_Id	0,441403	0,846135	0,913912	n.s.
wavelet.HHH_glcm_DifferenceAverage	0,439992	0,847115	0,913912	n.s.
wavelet.HHH_glcm_Idm	0,440267	0,846924	0,913912	n.s.
wavelet.HHL_gldm_DependenceNonUniformityNormalized	0,437036	0,849161	0,915015	n.s.
wavelet.LHH_gldm_SmallDependenceEmphasis	0,436317	0,849657	0,915015	n.s.
wavelet.LHH_glszm_GrayLevelNonUniformity	0,433578	0,851545	0,916228	n.s.
wavelet.HHL_firstorder_Kurtosis	0,42009	0,860733	0,925215	n.s.
wavelet.LLH_glcm_Correlation	0,417062	0,862771	0,925215	n.s.
wavelet.HLH_glszm_SizeZoneNonUniformity	0,417074	0,862763	0,925215	n.s.
log.sigma.3.0.mm.3D_firstorder_Median	0,416765	0,86297	0,925215	n.s.
wavelet.LLH_glrlm_LongRunHighGrayLevelEmphasis	0,412191	0,86603	0,926074	n.s.
wavelet.LHH_firstorder_10Percentile	0,412998	0,865492	0,926074	n.s.
wavelet.LHH_gldm_GrayLevelVariance	0,412118	0,866079	0,926074	n.s.
wavelet.HHH_glrlm_LongRunEmphasis	0,407121	0,869396	0,928361	n.s.
log.sigma.4.0.mm.3D_gldm_SmallDependenceLowGrayLevelEmphasis	0,40657	0,86976	0,928361	n.s.
wavelet.LHH_gldm_SmallDependenceHighGrayLevelEmphasis	0,398723	0,874909	0,933029	n.s.
wavelet.HLH_gldm_DependenceVariance	0,396408	0,876415	0,933809	n.s.
wavelet.LHH_glcm_SumEntropy	0,393394	0,878367	0,934234	n.s.
original_glcm_InverseVariance	0,394372	0,877734	0,934234	n.s.
original_glszm_LowGrayLevelZoneEmphasis	0,386499	0,882791	0,938111	n.s.
wavelet.LLH_gldm_GrayLevelVariance	0,384567	0,88402	0,938589	n.s.
wavelet.LLH_glszm_LargeAreaEmphasis	0,382208	0,885515	0,939348	n.s.
wavelet.LHH_firstorder_90Percentile	0,373567	0,890933	0,942719	n.s.
wavelet.LHH_glcm_Contrast	0,374602	0,890289	0,942719	n.s.
log.sigma.2.0.mm.3D_glcm_Imc2	0,371885	0,891977	0,942719	n.s.
log.sigma.3.0.mm.3D_glszm_LowGrayLevelZoneEmphasis	0,37154	0,89219	0,942719	n.s.
log.sigma.5.0.mm.3D_firstorder_90Percentile	0,370864	0,892608	0,942719	n.s.
wavelet.LLH_glrlm_GrayLevelVariance	0,366751	0,895139	0,943575	n.s.
wavelet.HLL_firstorder_Energy	0,374288	0,895769	0,943575	n.s.
wavelet.HLL_firstorder_TotalEnergy	0,374288	0,895769	0,943575	n.s.
log.sigma.3.0.mm.3D_gldm_SmallDependenceLowGrayLevelEmphasis	0,358246	0,900302	0,94752	n.s.
original_glrlm_ShortRunLowGrayLevelEmphasis	0,35345	0,903169	0,949708	n.s.

log.sigma.4.0.mm.3D_glcm_Correlation	0,349671	0,905406	0,951229	n.s.
wavelet.LLH_glcm_Autocorrelation	0,345723	0,907721	0,952	n.s.
log.sigma.3.0.mm.3D_firstorder_Mean	0,346944	0,907007	0,952	n.s.
wavelet.LLH_glrlm_HighGrayLevelRunEmphasis	0,337513	0,912463	0,95284	n.s.
wavelet.LLH_glrlm_ShortRunHighGrayLevelEmphasis	0,337486	0,912478	0,95284	n.s.
wavelet.LHH_firstorder_Uniformity	0,337766	0,912318	0,95284	n.s.
log.sigma.3.0.mm.3D_firstorder_RootMeanSquared	0,342296	0,909713	0,95284	n.s.
original_glrlm_LongRunLowGrayLevelEmphasis	0,340865	0,910539	0,95284	n.s.
wavelet.HHL_firstorder_Median	0,332026	0,916239	0,953308	n.s.
wavelet.LLH_gldm_HighGrayLevelEmphasis	0,331671	0,915776	0,953308	n.s.
log.sigma.3.0.mm.3D_gldm_DependenceVariance	0,329693	0,916886	0,953308	n.s.
log.sigma.4.0.mm.3D_glszm_GrayLevelNonUniformity	0,334269	0,914309	0,953308	n.s.
original_glrlm_LowGrayLevelRunEmphasis	0,331225	0,916027	0,953308	n.s.
wavelet.HHL_firstorder_Minimum	0,320084	0,922192	0,953615	n.s.
wavelet.LLH_glszm_SizeZoneNonUniformity	0,326176	0,918845	0,953615	n.s.
wavelet.LLH_glszm_ZonePercentage	0,318088	0,923276	0,953615	n.s.
wavelet.LHH_glrlm_GrayLevelVariance	0,31995	0,922265	0,953615	n.s.
wavelet.HHH_glcm_Correlation	0,319181	0,922683	0,953615	n.s.
log.sigma.4.0.mm.3D_glszm_HighGrayLevelZoneEmphasis	0,3228	0,920707	0,953615	n.s.
log.sigma.4.0.mm.3D_glszm_SizeZoneNonUniformityNormalized	0,322779	0,920718	0,953615	n.s.
original_gldm_LowGrayLevelEmphasis	0,317642	0,923518	0,953615	n.s.
wavelet.LHH_glcm_DifferenceVariance	0,313433	0,925779	0,954092	n.s.
wavelet.LHH_glszm_SizeZoneNonUniformityNormalized	0,312768	0,926134	0,954092	n.s.
wavelet.HHH_firstorder_Minimum	0,31235	0,926356	0,954092	n.s.
wavelet.LHH_glrlm_RunEntropy	0,298781	0,933424	0,959728	n.s.
wavelet.HHH_glcm_ClusterTendency	0,299304	0,933157	0,959728	n.s.
wavelet.LHH_glcm_JointEnergy	0,28969	0,937983	0,963594	n.s.
wavelet.LLH_firstorder_Uniformity	0,285671	0,939953	0,964244	n.s.
log.sigma.3.0.mm.3D_glcm_Idmn	0,285127	0,940218	0,964244	n.s.
wavelet.LLH_glcm_DifferenceEntropy	0,275377	0,944866	0,967952	n.s.
wavelet.LLH_glcm_JointAverage	0,274143	0,945442	0,967952	n.s.
log.sigma.4.0.mm.3D_gldm_DependenceVariance	0,267337	0,948568	0,970328	n.s.
wavelet.LLH_firstorder_Entropy	0,258126	0,952661	0,973688	n.s.
wavelet.LHH_glrlm_GrayLevelNonUniformityNormalized	0,252085	0,955258	0,975447	n.s.
wavelet.LHH_glcm_JointEntropy	0,24958	0,956313	0,975447	n.s.
wavelet.LHH_glcm_DifferenceAverage	0,248383	0,956813	0,975447	n.s.
log.sigma.4.0.mm.3D_glszm_ZoneEntropy	0,245434	0,958034	0,975866	n.s.
wavelet.LLH_glrlm_GrayLevelNonUniformityNormalized	0,23926	0,960534	0,977584	n.s.
wavelet.LHH_firstorder_Entropy	0,230552	0,96393	0,980213	n.s.
log.sigma.2.0.mm.3D_glcm_Correlation	0,223351	0,966623	0,982122	n.s.
wavelet.HLL_firstorder_Kurtosis	0,21208	0,970624	0,982869	n.s.
log.sigma.1.0.mm.3D_glcm_InverseVariance	0,219018	0,968192	0,982869	n.s.
log.sigma.5.0.mm.3D_firstorder_Energy	0,221483	0,97004	0,982869	n.s.
log.sigma.5.0.mm.3D_firstorder_TotalEnergy	0,221483	0,97004	0,982869	n.s.
log.sigma.2.0.mm.3D_glcm_Imc1	0,203968	0,973341	0,984793	n.s.
wavelet.LHH_glrlm_RunPercentage	0,189265	0,977914	0,988493	n.s.

wavelet.LHH_glrlm_RunLengthNonUniformityNormalized	0,183922	0,979462	0,988493	n.s.
wavelet.LHH_glcm_Idm	0,186105	0,978837	0,988493	n.s.
wavelet.LHL_gldm_DependenceEntropy	0,173022	0,982432	0,988937	n.s.
wavelet.LHH_glrlm_ShortRunEmphasis	0,174323	0,982091	0,988937	n.s.
wavelet.LHH_glcm_Id	0,167357	0,983876	0,988937	n.s.
wavelet.LHH_gldm_LargeDependenceEmphasis	0,177686	0,981192	0,988937	n.s.
log.sigma.4.0.mm.3D_firstorder_90Percentile	0,164083	0,98468	0,988937	n.s.
log.sigma.4.0.mm.3D_glszm_LowGrayLevelZoneEmphasis	0,16346	0,98483	0,988937	n.s.
wavelet.LHH_glcm_DifferenceEntropy	0,119228	0,993421	0,996733	n.s.
wavelet.HHL_firstorder_Energy	0,099165	0,996483	0,998141	n.s.
wavelet.HHL_firstorder_TotalEnergy	0,099165	0,996483	0,998141	n.s.
wavelet.LHL_glszm_GrayLevelNonUniformity	0,045397	0,999561	0,999561	n.s.
log.sigma.2.0.mm.3D_firstorder_Kurtosis	0,054878	0,999244	0,999561	n.s.
