# Supplementary material for: Contrast Agent Dynamics Determine Radiomics Profiles in Oncologic Imaging
Source: Cancers (Basel). 2024 Apr 16;16(8):1519. doi: 10.3390/cancers16081519 (PMC11049400; doi:10.3390/cancers16081519)
Supplement: Supplementary file 1 [file cancers-16-01519-s001.zip › Table S2.pdf]

Table S2: Linear mixed model p values and cluster membership of all CT radiomics features for prostate tumors

Feature	F value	p value	FDR	Cluster
wavelet.LLL_firstorder_10Percentile	27,23873	5,41E-12	6,51E-09	1
original_firstorder_10Percentile	24,76451	2,1E-11	1,27E-08	1
wavelet.LLL_firstorder_Mean	17,41063	2,48E-09	6,79E-07	1
wavelet.LLL_firstorder_Median	16,65636	4,38E-09	6,79E-07	1
wavelet.LLL_firstorder_RootMeanSquared	16,61733	4,51E-09	6,79E-07	1
original_firstorder_Mean	17,43015	2,45E-09	6,79E-07	1
original_firstorder_Median	16,94702	3,51E-09	6,79E-07	1
original_firstorder_RootMeanSquared	17,09667	3,14E-09	6,79E-07	1
log.sigma.2.0.mm.3D_firstorder_Energy	8,120235	8,48E-09	1,02E-06	2
log.sigma.2.0.mm.3D_firstorder_TotalEnergy	8,120235	8,48E-09	1,02E-06	2
log.sigma.1.0.mm.3D_firstorder_Energy	7,858671	1,74E-08	1,75E-06	2
log.sigma.1.0.mm.3D_firstorder_TotalEnergy	7,858671	1,74E-08	1,75E-06	2
wavelet.LLH_firstorder_Energy	7,770431	2,23E-08	1,91E-06	2
wavelet.LLH_firstorder_TotalEnergy	7,770431	2,23E-08	1,91E-06	2
log.sigma.3.0.mm.3D_firstorder_Energy	7,000608	1,84E-07	1,38E-05	2
log.sigma.3.0.mm.3D_firstorder_TotalEnergy	7,000608	1,84E-07	1,38E-05	2
log.sigma.5.0.mm.3D_firstorder_90Percentile	11,67295	3,14E-07	2,22E-05	2
wavelet.LLL_firstorder_90Percentile	10,35442	1,17E-06	7,85E-05	1
original_firstorder_90Percentile	10,22599	1,34E-06	8,5E-05	1
log.sigma.4.0.mm.3D_firstorder_90Percentile	9,730438	2,26E-06	0,000136	2
wavelet.LLL_firstorder_Maximum	9,538095	2,79E-06	0,000154	1
log.sigma.4.0.mm.3D_firstorder_Energy	5,976198	2,94E-06	0,000154	2
log.sigma.4.0.mm.3D_firstorder_TotalEnergy	5,976198	2,94E-06	0,000154	2
wavelet.LLL_firstorder_Energy	5,774758	5,04E-06	0,000202	1
wavelet.LLL_firstorder_TotalEnergy	5,774758	5,04E-06	0,000202	1
log.sigma.2.0.mm.3D_glszm_ZoneEntropy	9,10847	4,47E-06	0,000202	1
log.sigma.5.0.mm.3D_firstorder_Mean	9,096205	4,53E-06	0,000202	2
log.sigma.5.0.mm.3D_firstorder_RootMeanSquared	9,109004	4,46E-06	0,000202	2
original_firstorder_Energy	5,779267	4,98E-06	0,000202	1
original_firstorder_TotalEnergy	5,779267	4,98E-06	0,000202	1
log.sigma.5.0.mm.3D_firstorder_Median	8,647163	7,51E-06	0,000284	2
original_firstorder_Maximum	8,641996	7,56E-06	0,000284	1
wavelet.HLH_firstorder_InterquartileRange	8,449874	9,42E-06	0,000344	1
wavelet.LLL_glcm_Imc2	8,165853	1,31E-05	0,000465	1
log.sigma.2.0.mm.3D_glrlm_LongRunEmphasis	7,979484	1,64E-05	0,000563	2
log.sigma.4.0.mm.3D_firstorder_RootMeanSquared	7,570064	2,68E-05	0,000896	2
log.sigma.4.0.mm.3D_firstorder_Mean	7,531825	2,81E-05	0,000914	2
log.sigma.2.0.mm.3D_glrlm_ShortRunEmphasis	7,503525	2,91E-05	0,000921	1
log.sigma.5.0.mm.3D_firstorder_Energy	5,085386	3,14E-05	0,000945	2
log.sigma.5.0.mm.3D_firstorder_TotalEnergy	5,085386	3,14E-05	0,000945	2
log.sigma.1.0.mm.3D_firstorder_Range	7,373465	3,41E-05	0,001002	1

log.sigma.4.0.mm.3D_firstorder_Median	7,340504	3,55E-05	0,001019	2
log.sigma.2.0.mm.3D_glszm_ZonePercentage	7,262323	3,92E-05	0,001097	1
log.sigma.5.0.mm.3D_firstorder_10Percentile	7,065753	5,01E-05	0,001371	2
log.sigma.2.0.mm.3D_glrlm_GrayLevelNonUniformityNormalized	6,973229	5,63E-05	0,001455	2
log.sigma.2.0.mm.3D_gldm_SmallDependenceEmphasis	6,966442	5,68E-05	0,001455	1
log.sigma.3.0.mm.3D_gldm_DependenceEntropy	6,975004	5,62E-05	0,001455	1
log.sigma.2.0.mm.3D_firstorder_Median	6,93905	5,88E-05	0,001475	2
original_glcm_JointAverage	6,91272	6,08E-05	0,001494	1
log.sigma.1.0.mm.3D_glszm_GrayLevelVariance	6,789201	7,12E-05	0,001715	1
log.sigma.2.0.mm.3D_glcm_DifferenceEntropy	6,65725	8,44E-05	0,001992	1
log.sigma.3.0.mm.3D_firstorder_RootMeanSquared	6,589421	9,22E-05	0,002134	2
wavelet.HLH_firstorder_RobustMeanAbsoluteDeviation	6,539591	9,83E-05	0,00214	1
log.sigma.2.0.mm.3D_firstorder_Minimum	6,538	9,85E-05	0,00214	2
log.sigma.3.0.mm.3D_firstorder_Mean	6,530428	9,95E-05	0,00214	2
log.sigma.3.0.mm.3D_firstorder_Median	6,565822	9,5E-05	0,00214	2
wavelet.HLL_glrlm_RunLengthNonUniformity	6,508891	0,000102	0,002162	1
log.sigma.1.0.mm.3D_glrlm_GrayLevelVariance	6,441594	0,000112	0,002273	1
log.sigma.2.0.mm.3D_glcm_InverseVariance	6,446664	0,000111	0,002273	1
original_glrlm_LongRunHighGrayLevelEmphasis	6,431514	0,000113	0,002273	1
log.sigma.2.0.mm.3D_firstorder_RootMeanSquared	6,403775	0,000117	0,002319	2
wavelet.LLH_glcm_DifferenceEntropy	6,369127	0,000123	0,002378	1
log.sigma.2.0.mm.3D_firstorder_Mean	6,359595	0,000125	0,002378	2
log.sigma.2.0.mm.3D_gldm_LargeDependenceEmphasis	6,348041	0,000126	0,002378	2
original_glcm_Imc2	6,334601	0,000129	0,002384	1
log.sigma.2.0.mm.3D_glrlm_RunPercentage	6,308824	0,000133	0,002429	1
log.sigma.3.0.mm.3D_glrlm_LongRunEmphasis	6,232028	0,000147	0,002649	2
log.sigma.1.0.mm.3D_firstorder_Minimum	6,173713	0,000159	0,002804	2
log.sigma.1.0.mm.3D_glrlm_RunEntropy	6,167333	0,000161	0,002804	1
log.sigma.1.0.mm.3D_glrlm_GrayLevelNonUniformity	6,142285	0,000166	0,002858	2
wavelet.HLL_firstorder_Mean	6,126607	0,00017	0,002878	2
wavelet.HLL_gldm_GrayLevelNonUniformity	6,083443	0,00018	0,002926	2
log.sigma.1.0.mm.3D_glszm_SmallAreaHighGrayLevelEmphasis	6,087304	0,000179	0,002926	1
original_glrlm_RunLengthNonUniformity	6,085157	0,000179	0,002926	1
log.sigma.2.0.mm.3D_firstorder_Entropy	6,042486	0,00019	0,00305	1
wavelet.HLH_firstorder_MeanAbsoluteDeviation	6,006695	0,000199	0,003159	1
wavelet.HLH_firstorder_10Percentile	5,968071	0,00021	0,003285	2
log.sigma.2.0.mm.3D_glcm_SumEntropy	5,956501	0,000213	0,003294	1
log.sigma.2.0.mm.3D_glcm_JointEntropy	5,941247	0,000218	0,003321	1
original_gldm_GrayLevelNonUniformity	5,929063	0,000222	0,003334	2
wavelet.HLL_firstorder_10Percentile	5,846072	0,000248	0,003593	2
wavelet.HLL_firstorder_Variance	5,838161	0,000251	0,003593	1
wavelet.LLL_gldm_GrayLevelNonUniformity	5,848506	0,000247	0,003593	2
original_gldm_HighGrayLevelEmphasis	5,841593	0,00025	0,003593	1
wavelet.LLL_glrlm_GrayLevelNonUniformity	5,75529	0,000281	0,003668	2
wavelet.LLL_glrlm_GrayLevelNonUniformityNormalized	5,753509	0,000281	0,003668	2
log.sigma.1.0.mm.3D_firstorder_Mean	5,746619	0,000284	0,003668	2

log.sigma.2.0.mm.3D_glrlm_RunLengthNonUniformityNormalized	5,745153	0,000285	0,003668	1
log.sigma.2.0.mm.3D_glcm_Id	5,812882	0,000259	0,003668	2
log.sigma.5.0.mm.3D_firstorder_Minimum	5,803609	0,000263	0,003668	2
original_glrlm_HighGrayLevelRunEmphasis	5,782883	0,00027	0,003668	1
original_glcm_Autocorrelation	5,741047	0,000286	0,003668	1
original_glszm_GrayLevelVariance	5,766566	0,000276	0,003668	1
original_gldm_LargeDependenceHighGrayLevelEmphasis	5,778669	0,000272	0,003668	1
wavelet.HLL_glcm_DifferenceAverage	5,730672	0,00029	0,003681	1
wavelet.HLL_glrlm_RunLengthNonUniformityNormalized	5,722799	0,000294	0,003683	1
wavelet.HLL_firstorder_RootMeanSquared	5,660948	0,00032	0,003843	2
log.sigma.1.0.mm.3D_firstorder_10Percentile	5,670274	0,000316	0,003843	2
log.sigma.1.0.mm.3D_firstorder_RootMeanSquared	5,65677	0,000322	0,003843	2
log.sigma.1.0.mm.3D_firstorder_Entropy	5,682339	0,00031	0,003843	1
log.sigma.2.0.mm.3D_glcm_Idm	5,654958	0,000322	0,003843	2
log.sigma.2.0.mm.3D_glcm_DifferenceVariance	5,647777	0,000326	0,003844	1
wavelet.HLL_firstorder_MeanAbsoluteDeviation	5,627802	0,000335	0,003876	1
wavelet.HLL_glrlm_ShortRunEmphasis	5,627728	0,000335	0,003876	1
log.sigma.1.0.mm.3D_firstorder_Variance	5,579525	0,000358	0,004101	1
original_glrlm_ShortRunHighGrayLevelEmphasis	5,573307	0,000361	0,004101	1
wavelet.HLL_glrlm_GrayLevelNonUniformity	5,503272	0,000398	0,00429	2
wavelet.LLL_firstorder_Uniformity	5,501418	0,000399	0,00429	2
wavelet.LLL_glrlm_RunEntropy	5,510349	0,000394	0,00429	1
log.sigma.1.0.mm.3D_glszm_HighGrayLevelZoneEmphasis	5,514331	0,000392	0,00429	1
log.sigma.4.0.mm.3D_firstorder_10Percentile	5,520373	0,000389	0,00429	2
original_glrlm_GrayLevelNonUniformity	5,502382	0,000399	0,00429	2
wavelet.LLL_glcm_SumEntropy	5,464567	0,00042	0,004477	1
log.sigma.1.0.mm.3D_gldm_GrayLevelVariance	5,441357	0,000434	0,004505	1
log.sigma.2.0.mm.3D_glcm_DifferenceAverage	5,441299	0,000434	0,004505	1
original_gldm_SmallDependenceEmphasis	5,444827	0,000432	0,004505	1
log.sigma.3.0.mm.3D_glcm_DifferenceEntropy	5,432205	0,00044	0,004524	1
wavelet.LLH_glrlm_GrayLevelNonUniformity	5,410212	0,000453	0,004626	2
wavelet.LLL_firstorder_Entropy	5,385451	0,000469	0,004657	1
log.sigma.1.0.mm.3D_firstorder_Median	5,398538	0,000461	0,004657	2
log.sigma.2.0.mm.3D_firstorder_Uniformity	5,381858	0,000472	0,004657	2
log.sigma.2.0.mm.3D_glrlm_RunLengthNonUniformity	5,388063	0,000468	0,004657	1
wavelet.HLL_glcm_DifferenceEntropy	5,369708	0,00048	0,004698	1
log.sigma.1.0.mm.3D_glrlm_GrayLevelNonUniformityNormalized	5,360571	0,000486	0,004721	2
wavelet.HLL_firstorder_Entropy	5,314835	0,000519	0,004971	1
wavelet.HLL_glrlm_GrayLevelVariance	5,309038	0,000523	0,004971	1
log.sigma.3.0.mm.3D_glcm_MaximumProbability	5,307117	0,000524	0,004971	2
wavelet.LLH_glrlm_RunEntropy	5,285895	0,00054	0,005043	1
wavelet.HLL_glcm_Id	5,2882	0,000539	0,005043	2
wavelet.HLL_firstorder_Minimum	5,279992	0,000545	0,005046	2
wavelet.HLL_glcm_JointEntropy	5,252893	0,000566	0,005204	1
wavelet.LLH_firstorder_Entropy	5,247204	0,000571	0,005206	1
log.sigma.3.0.mm.3D_firstorder_90Percentile	5,229083	0,000586	0,005294	2

original_glrlm_GrayLevelNonUniformityNormalized	5,224772	0,000589	0,005294	2
wavelet.HLL_glcm_Idm	5,207202	0,000604	0,005388	2
wavelet.HLL_gldm_GrayLevelVariance	5,180441	0,000628	0,005557	1
log.sigma.1.0.mm.3D_glcm_Imc2	5,150121	0,000655	0,00576	1
wavelet.LLH_glcm_JointEntropy	5,13237	0,000672	0,005865	1
wavelet.LLL_glcm_JointEntropy	5,127353	0,000677	0,005865	1
log.sigma.2.0.mm.3D_glcm_MaximumProbability	5,099887	0,000704	0,006057	2
wavelet.LLH_glcm_DifferenceAverage	5,092525	0,000712	0,00606	1
wavelet.LLL_glszm_GrayLevelNonUniformityNormalized	5,089563	0,000715	0,00606	2
log.sigma.3.0.mm.3D_glcm_JointEnergy	5,07512	0,00073	0,006104	2
log.sigma.4.0.mm.3D_gldm_DependenceEntropy	5,069992	0,000735	0,006104	1
original_glcm_SumEntropy	5,074017	0,000731	0,006104	1
original_firstorder_Range	5,056038	0,00075	0,006185	1
wavelet.LLL_glrlm_RunLengthNonUniformity	5,047549	0,000759	0,006219	1
log.sigma.2.0.mm.3D_firstorder_Range	5,038427	0,000769	0,006258	1
log.sigma.1.0.mm.3D_gldm_SmallDependenceHighGrayLevelEmphasis	5,007142	0,000805	0,006419	1
log.sigma.3.0.mm.3D_glcm_DifferenceVariance	5,006868	0,000805	0,006419	1
original_firstorder_Entropy	5,00836	0,000803	0,006419	1
wavelet.HLL_glrlm_RunPercentage	4,990195	0,000825	0,006447	1
wavelet.LLL_glcm_JointAverage	4,994478	0,00082	0,006447	1
wavelet.LLL_gldm_DependenceEntropy	4,9956	0,000818	0,006447	1
wavelet.LLH_glcm_Imc2	4,96161	0,000859	0,006503	1
wavelet.HLL_glcm_SumSquares	4,95351	0,00087	0,006503	1
wavelet.LLL_glszm_ZoneEntropy	4,973734	0,000845	0,006503	1
log.sigma.2.0.mm.3D_glrlm_RunVariance	4,959237	0,000862	0,006503	2
log.sigma.2.0.mm.3D_glcm_JointEnergy	4,959135	0,000863	0,006503	2
log.sigma.3.0.mm.3D_glrlm_RunVariance	4,965135	0,000855	0,006503	2
original_glcm_JointEntropy	4,956173	0,000866	0,006503	1
log.sigma.2.0.mm.3D_firstorder_10Percentile	4,919912	0,000913	0,006785	2
wavelet.LLL_firstorder_Range	4,909378	0,000927	0,006848	1
log.sigma.1.0.mm.3D_gldm_GrayLevelNonUniformity	4,896547	0,000944	0,006934	2
original_glrlm_RunEntropy	4,889854	0,000954	0,006951	1
original_glszm_GrayLevelNonUniformityNormalized	4,886549	0,000958	0,006951	2
log.sigma.1.0.mm.3D_glcm_DifferenceEntropy	4,880838	0,000966	0,006967	1
wavelet.HLL_glcm_Contrast	4,87632	0,000973	0,006971	1
wavelet.HLL_glcm_SumEntropy	4,865285	0,000988	0,006987	1
original_firstorder_Uniformity	4,862554	0,000992	0,006987	2
original_glcm_Correlation	4,870179	0,000981	0,006987	1
wavelet.LLL_glcm_Imc1	4,833731	0,001035	0,007245	2
wavelet.HLL_glcm_ClusterTendency	4,809931	0,001072	0,007457	1
log.sigma.1.0.mm.3D_glcm_JointEntropy	4,802842	0,001083	0,007492	1
wavelet.LLH_firstorder_10Percentile	4,787613	0,001107	0,007573	2
log.sigma.2.0.mm.3D_gldm_DependenceEntropy	4,791364	0,001101	0,007573	1
original_gldm_LowGrayLevelEmphasis	4,778047	0,001123	0,007637	2
wavelet.LLL_glrlm_LongRunHighGrayLevelEmphasis	4,771153	0,001134	0,007667	1
wavelet.LLL_glszm_GrayLevelVariance	4,760123	0,001153	0,007667	1

log.sigma.2.0.mm.3D_gldm_GrayLevelNonUniformity	4,766055	0,001143	0,007667	2
log.sigma.3.0.mm.3D_glcm_SumEntropy	4,762036	0,001149	0,007667	1
log.sigma.1.0.mm.3D_glszm_ZonePercentage	4,737639	0,001191	0,00788	1
log.sigma.2.0.mm.3D_glrlm_GrayLevelNonUniformity	4,721097	0,001221	0,00803	2
wavelet.LHH_glszm_SizeZoneNonUniformity	4,701431	0,001256	0,008133	1
log.sigma.1.0.mm.3D_glcm_SumEntropy	4,701881	0,001256	0,008133	1
log.sigma.3.0.mm.3D_firstorder_10Percentile	4,70629	0,001247	0,008133	2
original_glrlm_LongRunLowGrayLevelEmphasis	4,690089	0,001278	0,008225	2
original_glrlm_LowGrayLevelRunEmphasis	4,681685	0,001293	0,008283	2
wavelet.LLL_gldm_HighGrayLevelEmphasis	4,677804	0,001301	0,008287	1
wavelet.LLL_glrlm_HighGrayLevelRunEmphasis	4,660084	0,001335	0,008367	1
log.sigma.2.0.mm.3D_glszm_SizeZoneNonUniformityNormalized	4,657118	0,001341	0,008367	2
log.sigma.3.0.mm.3D_gldm_LargeDependenceEmphasis	4,657964	0,001339	0,008367	2
original_glszm_HighGrayLevelZoneEmphasis	4,65878	0,001338	0,008367	1
log.sigma.2.0.mm.3D_gldm_SmallDependenceHighGrayLevelEmphasis	4,651349	0,001353	0,008395	1
log.sigma.1.0.mm.3D_firstorder_MeanAbsoluteDeviation	4,640933	0,001374	0,008481	1
log.sigma.1.0.mm.3D_glcm_DifferenceVariance	4,633203	0,001389	0,008535	1
log.sigma.2.0.mm.3D_glcm_Contrast	4,628988	0,001398	0,008544	1
wavelet.LLL_glrlm_ShortRunHighGrayLevelEmphasis	4,622874	0,001411	0,008578	1
wavelet.HLL_glrlm_RunEntropy	4,598534	0,001463	0,00876	1
wavelet.LLL_glcm_Correlation	4,600919	0,001457	0,00876	1
original_glcm_DifferenceVariance	4,601431	0,001456	0,00876	1
log.sigma.3.0.mm.3D_glcm_JointEntropy	4,591151	0,001479	0,008813	1
wavelet.LLL_glcm_DifferenceEntropy	4,580823	0,001501	0,008905	1
wavelet.LLH_glcm_Contrast	4,572508	0,00152	0,008958	1
wavelet.LLL_glcm_Autocorrelation	4,566907	0,001533	0,008958	1
original_glrlm_ShortRunLowGrayLevelEmphasis	4,569079	0,001528	0,008958	2
log.sigma.3.0.mm.3D_firstorder_Uniformity	4,562616	0,001543	0,008972	2
log.sigma.3.0.mm.3D_firstorder_Entropy	4,531122	0,001616	0,009329	1
log.sigma.4.0.mm.3D_glcm_MaximumProbability	4,529902	0,001619	0,009329	2
wavelet.HLL_glszm_GrayLevelVariance	4,515436	0,001655	0,009487	1
wavelet.LLH_glrlm_GrayLevelNonUniformityNormalized	4,508477	0,001672	0,009501	2
log.sigma.1.0.mm.3D_glcm_SumSquares	4,508083	0,001673	0,009501	1
wavelet.LLL_gldm_SmallDependenceEmphasis	4,48264	0,001738	0,009822	1
wavelet.LLL_glszm_ZonePercentage	4,474219	0,00176	0,009854	1
log.sigma.1.0.mm.3D_glcm_ClusterTendency	4,475408	0,001756	0,009854	1
wavelet.LLL_glszm_SizeZoneNonUniformity	4,464585	0,001785	0,009914	1
wavelet.LLL_glszm_HighGrayLevelZoneEmphasis	4,463932	0,001787	0,009914	1
log.sigma.1.0.mm.3D_glrlm_RunLengthNonUniformity	4,458238	0,001802	0,009953	1
wavelet.LLL_glcm_JointEnergy	4,452387	0,001818	0,009995	2
wavelet.LLL_glrlm_RunLengthNonUniformityNormalized	4,433302	0,001871	0,010237	1
wavelet.LLL_glrlm_ShortRunEmphasis	4,427721	0,001886	0,010269	1
log.sigma.5.0.mm.3D_glszm_SmallAreaEmphasis	4,422174	0,001902	0,010269	1
original_gldm_LargeDependenceLowGrayLevelEmphasis	4,424337	0,001896	0,010269	2
wavelet.LLL_glcm_DifferenceVariance	4,413902	0,001926	0,010327	1
original_glcm_DifferenceEntropy	4,412458	0,00193	0,010327	1

wavelet.HLL_glszm_ZoneEntropy	4,38274	0,002018	0,010751	1
log.sigma.5.0.mm.3D_glszm_HighGrayLevelZoneEmphasis	4,371464	0,002052	0,010886	1
original_glszm_ZonePercentage	4,35923	0,002091	0,01104	1
log.sigma.3.0.mm.3D_glrlm_RunPercentage	4,35018	0,002119	0,011142	1
wavelet.LLL_glcm_DifferenceAverage	4,343294	0,002141	0,01121	1
log.sigma.1.0.mm.3D_glrlm_RunLengthNonUniformityNormalized	4,334775	0,002169	0,011257	1
original_gldm_DependenceEntropy	4,33604	0,002165	0,011257	1
log.sigma.1.0.mm.3D_glrlm_ShortRunEmphasis	4,317066	0,002228	0,011409	1
log.sigma.1.0.mm.3D_gldm_SmallDependenceEmphasis	4,318333	0,002223	0,011409	1
log.sigma.3.0.mm.3D_glcm_InverseVariance	4,320441	0,002216	0,011409	1
log.sigma.4.0.mm.3D_glrlm_LongRunEmphasis	4,314529	0,002236	0,011409	2
wavelet.HLL_firstorder_Range	4,302473	0,002277	0,011569	1
wavelet.HLL_firstorder_RobustMeanAbsoluteDeviation	4,299662	0,002287	0,01157	1
log.sigma.2.0.mm.3D_glszm_HighGrayLevelZoneEmphasis	4,291659	0,002315	0,011661	1
log.sigma.1.0.mm.3D_glszm_GrayLevelNonUniformityNormalized	4,287585	0,002329	0,011685	2
wavelet.LLL_glcm_Contrast	4,275142	0,002373	0,011857	1
wavelet.LLH_gldm_SmallDependenceEmphasis	4,242213	0,002495	0,012412	1
wavelet.LLL_glszm_SmallAreaHighGrayLevelEmphasis	4,230579	0,002539	0,012581	1
wavelet.LLH_firstorder_Mean	4,215094	0,002599	0,012723	2
wavelet.HLL_firstorder_Energy	3,360276	0,002592	0,012723	2
wavelet.HLL_firstorder_TotalEnergy	3,360276	0,002592	0,012723	2
log.sigma.4.0.mm.3D_glcm_JointEnergy	4,209982	0,00262	0,01277	2
original_glcm_Contrast	4,204737	0,002641	0,01282	1
log.sigma.1.0.mm.3D_glcm_Contrast	4,201783	0,002653	0,012826	1
wavelet.HLL_glrlm_GrayLevelNonUniformityNormalized	4,186891	0,002713	0,013067	2
wavelet.LLH_glcm_SumEntropy	4,182382	0,002732	0,013105	1
wavelet.HLH_firstorder_90Percentile	4,17808	0,00275	0,013139	1
wavelet.LLH_glszm_LargeAreaEmphasis	4,175088	0,002763	0,013146	2
wavelet.LLL_glcm_Id	4,154476	0,002851	0,013512	2
log.sigma.3.0.mm.3D_glcm_Id	4,148685	0,002876	0,013579	2
wavelet.LLH_firstorder_Uniformity	4,137992	0,002923	0,013648	2
wavelet.LLH_glcm_Idm	4,138662	0,00292	0,013648	2
log.sigma.5.0.mm.3D_glszm_SmallAreaHighGrayLevelEmphasis	4,137675	0,002925	0,013648	1
wavelet.LLH_glcm_Id	4,131935	0,00295	0,013653	2
wavelet.HLL_glcm_DifferenceVariance	4,131175	0,002954	0,013653	1
log.sigma.2.0.mm.3D_glszm_SizeZoneNonUniformity	4,129872	0,00296	0,013653	1
wavelet.LLH_glszm_ZoneVariance	4,111728	0,003043	0,013983	2
log.sigma.3.0.mm.3D_glcm_Idm	4,105523	0,003072	0,014037	2
log.sigma.4.0.mm.3D_glcm_DifferenceVariance	4,103984	0,003079	0,014037	1
original_glcm_JointEnergy	4,101768	0,003089	0,014037	2
original_glszm_ZoneEntropy	4,097234	0,003111	0,014081	1
wavelet.LLL_gldm_SmallDependenceHighGrayLevelEmphasis	4,085063	0,003169	0,014292	1
log.sigma.1.0.mm.3D_firstorder_Uniformity	4,063263	0,003277	0,014656	2
log.sigma.4.0.mm.3D_glcm_DifferenceEntropy	4,065532	0,003266	0,014656	1
original_gldm_SmallDependenceHighGrayLevelEmphasis	4,061375	0,003287	0,014656	1
wavelet.LLL_glcm_Idm	4,052837	0,00333	0,01474	2

log.sigma.4.0.mm.3D_glrlm_RunLengthNonUniformity	4,053838	0,003325	0,01474	1
log.sigma.3.0.mm.3D_glcm_DifferenceAverage	4,042674	0,003382	0,014916	1
log.sigma.1.0.mm.3D_glcm_DifferenceAverage	4,030401	0,003447	0,015145	1
wavelet.HLL_glrlm_LongRunEmphasis	4,019282	0,003506	0,015294	2
original_glcm_Imc1	4,02137	0,003495	0,015294	2
wavelet.HLL_glszm_LargeAreaLowGrayLevelEmphasis	4,015351	0,003527	0,015331	2
original_glcm_DifferenceAverage	4,002816	0,003596	0,015573	1
log.sigma.3.0.mm.3D_glrlm_RunLengthNonUniformity	3,985436	0,003693	0,015938	1
wavelet.LHL_glrlm_ShortRunLowGrayLevelEmphasis	3,977259	0,00374	0,01596	2
wavelet.LHL_gldm_LowGrayLevelEmphasis	3,976475	0,003745	0,01596	2
log.sigma.4.0.mm.3D_firstorder_Uniformity	3,976523	0,003744	0,01596	2
original_glrlm_ShortRunEmphasis	3,975298	0,003752	0,01596	1
original_glszm_LargeAreaEmphasis	3,966336	0,003804	0,016125	2
wavelet.LHL_glrlm_LowGrayLevelRunEmphasis	3,957825	0,003854	0,016264	2
wavelet.LLL_glrlm_RunPercentage	3,951884	0,003889	0,016264	1
log.sigma.1.0.mm.3D_glrlm_ShortRunHighGrayLevelEmphasis	3,951715	0,00389	0,016264	1
original_glszm_LargeAreaLowGrayLevelEmphasis	3,953339	0,003881	0,016264	2
wavelet.LLH_gldm_GrayLevelNonUniformity	3,941634	0,003951	0,016351	2
wavelet.HLL_firstorder_Uniformity	3,939322	0,003966	0,016351	2
wavelet.HLL_gldm_LargeDependenceEmphasis	3,940927	0,003956	0,016351	2
log.sigma.4.0.mm.3D_glcm_Contrast	3,940355	0,003959	0,016351	1
wavelet.LLH_firstorder_RootMeanSquared	3,929956	0,004023	0,016533	2
wavelet.HLL_glcm_JointAverage	3,920573	0,004082	0,016718	1
log.sigma.4.0.mm.3D_glcm_SumEntropy	3,904858	0,004183	0,017071	1
wavelet.HHH_glszm_ZoneEntropy	3,899482	0,004218	0,017073	1
original_glrlm_RunLengthNonUniformityNormalized	3,9024	0,004199	0,017073	1
original_glcm_Idmn	3,898261	0,004226	0,017073	1
log.sigma.3.0.mm.3D_glrlm_LongRunLowGrayLevelEmphasis	3,894835	0,004248	0,017106	2
log.sigma.3.0.mm.3D_glrlm_ShortRunEmphasis	3,879626	0,004349	0,017455	1
wavelet.LLL_gldm_LargeDependenceHighGrayLevelEmphasis	3,867493	0,004432	0,017669	1
log.sigma.3.0.mm.3D_firstorder_Minimum	3,868483	0,004425	0,017669	2
wavelet.LLH_glcm_SumSquares	3,859787	0,004485	0,017764	1
log.sigma.4.0.mm.3D_glcm_JointEntropy	3,860145	0,004483	0,017764	1
log.sigma.4.0.mm.3D_glrlm_ShortRunEmphasis	3,853229	0,004531	0,017887	1
wavelet.LLL_gldm_DependenceNonUniformity	3,848181	0,004567	0,017969	1
log.sigma.2.0.mm.3D_glrlm_ShortRunHighGrayLevelEmphasis	3,837527	0,004643	0,018178	1
original_gldm_DependenceNonUniformity	3,836545	0,00465	0,018178	1
wavelet.HLL_firstorder_InterquartileRange	3,826364	0,004724	0,018408	1
original_glszm_ZoneVariance	3,800329	0,00492	0,019107	2
wavelet.LLH_firstorder_MeanAbsoluteDeviation	3,7963	0,004951	0,019166	1
log.sigma.2.0.mm.3D_firstorder_MeanAbsoluteDeviation	3,78908	0,005007	0,01932	1
log.sigma.2.0.mm.3D_firstorder_Skewness	3,779472	0,005082	0,019487	2
log.sigma.4.0.mm.3D_firstorder_Minimum	3,779492	0,005082	0,019487	2
wavelet.HLH_glrlm_GrayLevelNonUniformity	3,758927	0,005248	0,020058	2
log.sigma.3.0.mm.3D_gldm_GrayLevelNonUniformity	3,756222	0,00527	0,020079	2
log.sigma.1.0.mm.3D_glrlm_HighGrayLevelRunEmphasis	3,753765	0,00529	0,020093	1

log.sigma.1.0.mm.3D_glrlm_LongRunEmphasis	3,748079	0,005337	0,020208	2
original_glszm_SmallAreaHighGrayLevelEmphasis	3,745093	0,005362	0,020239	1
wavelet.HLL_glszm_GrayLevelNonUniformityNormalized	3,738165	0,005421	0,020332	2
log.sigma.3.0.mm.3D_glcm_Contrast	3,738154	0,005421	0,020332	1
wavelet.LHL_glrlm_LongRunLowGrayLevelEmphasis	3,728915	0,0055	0,020383	2
wavelet.HLL_gldm_DependenceNonUniformityNormalized	3,730278	0,005488	0,020383	1
wavelet.LLL_glcm_InverseVariance	3,732935	0,005465	0,020383	2
log.sigma.3.0.mm.3D_glcm_Idn	3,726684	0,005519	0,020383	2
original_glrlm_GrayLevelVariance	3,727883	0,005509	0,020383	1
log.sigma.4.0.mm.3D_glrlm_RunPercentage	3,713758	0,005632	0,020736	1
log.sigma.4.0.mm.3D_firstorder_Entropy	3,710255	0,005663	0,020786	1
log.sigma.1.0.mm.3D_glcm_Idm	3,704293	0,005716	0,020917	2
log.sigma.3.0.mm.3D_glrlm_GrayLevelNonUniformityNormalized	3,698026	0,005772	0,02106	2
wavelet.LLH_glrlm_LongRunEmphasis	3,694158	0,005807	0,021124	2
log.sigma.2.0.mm.3D_glszm_LowGrayLevelZoneEmphasis	3,690578	0,00584	0,021178	2
wavelet.HLH_glszm_ZoneEntropy	3,682369	0,005916	0,021388	1
log.sigma.1.0.mm.3D_glcm_Correlation	3,673675	0,005997	0,021617	1
log.sigma.4.0.mm.3D_glcm_DifferenceAverage	3,669981	0,006032	0,021678	1
wavelet.HLL_glszm_ZoneVariance	3,641543	0,006307	0,022575	2
original_glszm_LowGrayLevelZoneEmphasis	3,640364	0,006319	0,022575	2
log.sigma.4.0.mm.3D_gldm_LargeDependenceEmphasis	3,636213	0,00636	0,022656	2
wavelet.LLH_glrlm_RunPercentage	3,632635	0,006396	0,022717	1
original_glcm_Idm	3,622004	0,006504	0,023032	2
log.sigma.4.0.mm.3D_glcm_Idm	3,613606	0,006591	0,023235	2
log.sigma.4.0.mm.3D_gldm_DependenceNonUniformityNormalized	3,612699	0,0066	0,023235	2
original_glcm_Id	3,606152	0,006668	0,023407	2
wavelet.HLL_glszm_LargeAreaEmphasis	3,601249	0,00672	0,02345	2
wavelet.LLL_firstorder_MeanAbsoluteDeviation	3,599464	0,006739	0,02345	1
log.sigma.2.0.mm.3D_firstorder_RobustMeanAbsoluteDeviation	3,601167	0,006721	0,02345	1
wavelet.LLH_glrlm_ShortRunEmphasis	3,594226	0,006795	0,023508	1
log.sigma.1.0.mm.3D_glcm_Id	3,59534	0,006783	0,023508	2
original_glcm_Idn	3,582506	0,006922	0,023878	1
wavelet.HLL_glcm_Imc2	3,579409	0,006955	0,023927	1
log.sigma.4.0.mm.3D_glcm_Id	3,572947	0,007027	0,024063	2
original_firstorder_MeanAbsoluteDeviation	3,572189	0,007035	0,024063	1
wavelet.LLL_glrlm_LongRunEmphasis	3,568254	0,007079	0,024144	2
log.sigma.1.0.mm.3D_gldm_HighGrayLevelEmphasis	3,556695	0,007209	0,02452	1
wavelet.HLH_firstorder_Variance	3,54999	0,007286	0,024711	1
wavelet.LLH_glcm_DifferenceVariance	3,54513	0,007342	0,024762	1
log.sigma.2.0.mm.3D_glrlm_HighGrayLevelRunEmphasis	3,546276	0,007329	0,024762	1
wavelet.HLH_glcm_SumEntropy	3,53983	0,007404	0,024901	1
log.sigma.2.0.mm.3D_firstorder_InterquartileRange	3,534107	0,007471	0,024918	1
log.sigma.2.0.mm.3D_glrlm_GrayLevelVariance	3,53423	0,00747	0,024918	1
log.sigma.5.0.mm.3D_glrlm_RunLengthNonUniformity	3,536952	0,007438	0,024918	1
wavelet.HLH_glszm_ZonePercentage	3,52773	0,007547	0,025101	1
original_firstorder_Variance	3,518806	0,007654	0,025388	1

wavelet.LHH_gldm_SmallDependenceEmphasis	3,516015	0,007688	0,02543	1
wavelet.HLH_glcm_DifferenceEntropy	3,507363	0,007794	0,02571	1
wavelet.LLH_glrlm_RunLengthNonUniformityNormalized	3,504844	0,007825	0,025743	1
log.sigma.3.0.mm.3D_gldm_DependenceNonUniformityNormalized	3,482866	0,008103	0,02651	2
original_glrlm_RunPercentage	3,483155	0,008099	0,02651	1
wavelet.HLH_gldm_GrayLevelNonUniformity	3,474828	0,008207	0,026778	2
log.sigma.2.0.mm.3D_glcm_JointAverage	3,471652	0,008248	0,02684	1
log.sigma.3.0.mm.3D_glrlm_RunLengthNonUniformityNormalized	3,462629	0,008367	0,027154	1
log.sigma.1.0.mm.3D_glrlm_RunPercentage	3,458325	0,008425	0,027267	1
original_glrlm_LongRunEmphasis	3,4546	0,008475	0,027355	2
log.sigma.2.0.mm.3D_gldm_HighGrayLevelEmphasis	3,450793	0,008526	0,027447	1
wavelet.HLL_glcm_JointEnergy	3,445677	0,008596	0,027524	2
wavelet.LLL_gldm_DependenceNonUniformityNormalized	3,445988	0,008591	0,027524	1
wavelet.HLH_glcm_JointEntropy	3,442456	0,00864	0,027592	1
original_gldm_GrayLevelVariance	3,438355	0,008696	0,027699	1
log.sigma.1.0.mm.3D_glcm_ClusterProminence	3,431909	0,008786	0,02791	1
log.sigma.4.0.mm.3D_glcm_SumSquares	3,427373	0,008849	0,028039	1
wavelet.HLH_gldm_SmallDependenceEmphasis	3,416417	0,009005	0,028457	1
wavelet.LLH_glcm_ClusterTendency	3,410792	0,009086	0,028563	1
log.sigma.5.0.mm.3D_glrlm_LongRunEmphasis	3,411628	0,009074	0,028563	2
log.sigma.4.0.mm.3D_glcm_InverseVariance	3,406108	0,009154	0,028702	1
wavelet.HLH_glcm_JointEnergy	3,398898	0,00926	0,028958	2
wavelet.LLL_glszm_LargeAreaEmphasis	3,386323	0,009447	0,029391	2
log.sigma.1.0.mm.3D_firstorder_InterquartileRange	3,387106	0,009435	0,029391	1
log.sigma.2.0.mm.3D_glszm_SmallAreaHighGrayLevelEmphasis	3,37857	0,009565	0,02968	1
wavelet.LLH_firstorder_Median	3,370316	0,009691	0,029996	2
log.sigma.4.0.mm.3D_glszm_LargeAreaEmphasis	2,809951	0,009813	0,030294	2
wavelet.HLL_glrlm_HighGrayLevelRunEmphasis	3,355409	0,009925	0,030561	1
wavelet.HLL_gldm_HighGrayLevelEmphasis	3,352716	0,009967	0,030614	1
wavelet.HLH_glrlm_GrayLevelNonUniformityNormalized	3,348208	0,01004	0,030757	2
wavelet.LLH_glcm_JointEnergy	3,343398	0,010117	0,030899	2
wavelet.HLL_glrlm_ShortRunHighGrayLevelEmphasis	3,342147	0,010137	0,030899	1
log.sigma.1.0.mm.3D_glcm_JointAverage	3,334633	0,01026	0,031193	1
log.sigma.1.0.mm.3D_firstorder_Maximum	3,320239	0,010499	0,031839	1
log.sigma.5.0.mm.3D_glrlm_GrayLevelVariance	3,31831	0,010531	0,031857	1
log.sigma.5.0.mm.3D_gldm_GrayLevelVariance	3,314627	0,010593	0,031965	1
wavelet.HLL_gldm_DependenceNonUniformity	3,308256	0,010702	0,032212	1
wavelet.LLH_glszm_ZonePercentage	3,301839	0,010812	0,032223	1
wavelet.LLH_gldm_GrayLevelVariance	3,30273	0,010797	0,032223	1
wavelet.LLH_gldm_DependenceNonUniformityNormalized	3,30327	0,010788	0,032223	1
wavelet.HLH_firstorder_Entropy	3,306256	0,010736	0,032223	1
wavelet.HHL_glszm_GrayLevelNonUniformityNormalized	3,295146	0,010929	0,032489	2
log.sigma.2.0.mm.3D_glcm_Autocorrelation	3,29131	0,010996	0,032609	1
log.sigma.4.0.mm.3D_glcm_ClusterTendency	3,287016	0,011072	0,032754	1
wavelet.LLL_gldm_LargeDependenceEmphasis	3,281356	0,011173	0,03289	2
log.sigma.5.0.mm.3D_glszm_GrayLevelVariance	3,282399	0,011154	0,03289	1

wavelet.HLL_gldm_LargeDependenceLowGrayLevelEmphasis	3,272027	0,011341	0,033143	2
log.sigma.1.0.mm.3D_glcm_Autocorrelation	3,272489	0,011333	0,033143	1
original_glcm_SumSquares	3,274935	0,011288	0,033143	1
wavelet.LLH_glrlm_RunVariance	3,265505	0,01146	0,03333	2
wavelet.HLL_glcm_Autocorrelation	3,265729	0,011456	0,03333	1
wavelet.HLH_glrlm_RunEntropy	3,262115	0,011523	0,033431	1
log.sigma.1.0.mm.3D_glszm_ZoneEntropy	3,254537	0,011664	0,033758	1
wavelet.LLL_glrlm_GrayLevelVariance	3,250159	0,011746	0,033915	1
wavelet.HLL_glrlm_LongRunHighGrayLevelEmphasis	3,245162	0,011841	0,034106	1
wavelet.HLL_glszm_SmallAreaLowGrayLevelEmphasis	3,241051	0,011919	0,03425	2
log.sigma.1.0.mm.3D_firstorder_RobustMeanAbsoluteDeviation	3,231783	0,012098	0,034681	1
wavelet.HLL_glszm_GrayLevelNonUniformity	3,228435	0,012163	0,034712	2
log.sigma.5.0.mm.3D_firstorder_MeanAbsoluteDeviation	3,226802	0,012195	0,034712	1
log.sigma.5.0.mm.3D_glrlm_RunLengthNonUniformityNormalized	3,227297	0,012186	0,034712	1
wavelet.LLL_glszm_ZoneVariance	3,222705	0,012276	0,034777	2
log.sigma.4.0.mm.3D_gldm_GrayLevelVariance	3,222785	0,012274	0,034777	1
wavelet.HLL_glrlm_RunVariance	3,195627	0,012822	0,036239	2
wavelet.LHL_glcm_JointAverage	3,191899	0,012899	0,036289	1
wavelet.HLL_gldm_DependenceEntropy	3,191868	0,0129	0,036289	1
log.sigma.1.0.mm.3D_glrlm_RunVariance	3,18648	0,013012	0,03652	2
wavelet.LHL_glszm_LowGrayLevelZoneEmphasis	3,180009	0,013149	0,036816	2
log.sigma.5.0.mm.3D_firstorder_Variance	3,177559	0,013201	0,036876	1
wavelet.HLL_firstorder_Skewness	3,175792	0,013238	0,036896	2
log.sigma.4.0.mm.3D_glrlm_RunLengthNonUniformityNormalized	3,16827	0,0134	0,037259	1
wavelet.LLL_firstorder_Variance	3,163033	0,013513	0,037489	1
log.sigma.1.0.mm.3D_gldm_DependenceEntropy	3,161424	0,013548	0,037496	1
log.sigma.5.0.mm.3D_glcm_SumSquares	3,160058	0,013578	0,037496	1
wavelet.LLL_gldm_GrayLevelVariance	3,140055	0,014024	0,038637	1
log.sigma.5.0.mm.3D_firstorder_Range	3,119169	0,014505	0,039871	1
wavelet.LLH_gldm_DependenceEntropy	3,116822	0,01456	0,039931	1
log.sigma.4.0.mm.3D_glszm_GrayLevelVariance	3,111509	0,014685	0,040184	1
wavelet.LLH_glszm_LargeAreaLowGrayLevelEmphasis	3,106542	0,014804	0,040416	2
wavelet.HLL_glszm_HighGrayLevelZoneEmphasis	3,101021	0,014936	0,040686	1
log.sigma.1.0.mm.3D_glszm_LargeAreaLowGrayLevelEmphasis	3,093155	0,015127	0,041114	2
original_glcm_ClusterTendency	3,091552	0,015167	0,041128	1
log.sigma.2.0.mm.3D_gldm_GrayLevelVariance	3,088941	0,015231	0,041209	1
wavelet.LLH_firstorder_Variance	3,087382	0,015269	0,04122	1
log.sigma.5.0.mm.3D_gldm_LargeDependenceEmphasis	3,07721	0,015523	0,041811	2
log.sigma.4.0.mm.3D_glrlm_ShortRunLowGrayLevelEmphasis	3,074677	0,015587	0,041889	2
wavelet.HHH_glcm_ClusterShade	2,999634	0,015699	0,042098	2
log.sigma.3.0.mm.3D_gldm_LargeDependenceLowGrayLevelEmphasis	3,067189	0,015777	0,042211	2
wavelet.LLL_firstorder_RobustMeanAbsoluteDeviation	3,050897	0,016198	0,043244	1
wavelet.LLL_glcm_SumSquares	3,047245	0,016295	0,043404	1
log.sigma.1.0.mm.3D_glszm_LowGrayLevelZoneEmphasis	3,02897	0,016785	0,044611	2
log.sigma.5.0.mm.3D_glrlm_RunPercentage	3,015517	0,017155	0,045495	1
log.sigma.1.0.mm.3D_glszm_LargeAreaEmphasis	3,007518	0,017379	0,045988	2

log.sigma.5.0.mm.3D_glcm_ClusterTendency	2,997016	0,017678	0,046676	1
log.sigma.1.0.mm.3D_glszm_ZoneVariance	2,993899	0,017768	0,046811	2
wavelet.LLH_glrlm_GrayLevelVariance	2,982961	0,018086	0,047453	1
log.sigma.5.0.mm.3D_firstorder_InterquartileRange	2,982819	0,018091	0,047453	1
wavelet.LHL_glrlm_HighGrayLevelRunEmphasis	2,980833	0,018149	0,047503	1
wavelet.LLH_gldm_LargeDependenceEmphasis	2,973551	0,018365	0,047861	2
wavelet.LHL_glrlm_LongRunHighGrayLevelEmphasis	2,97458	0,018334	0,047861	1
wavelet.LHL_gldm_HighGrayLevelEmphasis	2,970469	0,018457	0,047973	1
log.sigma.4.0.mm.3D_glrlm_GrayLevelNonUniformityNormalized	2,969451	0,018488	0,047973	2
log.sigma.3.0.mm.3D_glszm_LowGrayLevelZoneEmphasis	2,959602	0,018787	0,048643	2
original_glrlm_RunVariance	2,955903	0,0189	0,048832	2
log.sigma.5.0.mm.3D_firstorder_RobustMeanAbsoluteDeviation	2,953754	0,018966	0,048898	1
wavelet.HLH_firstorder_Uniformity	2,934921	0,019557	0,050312	n.s.
wavelet.LHH_firstorder_Energy	2,512795	0,019668	0,050383	n.s.
wavelet.LHH_firstorder_TotalEnergy	2,512795	0,019668	0,050383	n.s.
wavelet.LHL_glrlm_ShortRunHighGrayLevelEmphasis	2,928691	0,019756	0,050394	n.s.
log.sigma.2.0.mm.3D_glrlm_LongRunLowGrayLevelEmphasis	2,929598	0,019727	0,050394	n.s.
wavelet.LHL_glcm_Autocorrelation	2,912912	0,02027	0,051535	n.s.
wavelet.LLL_glrlm_RunVariance	2,911436	0,020319	0,051535	n.s.
log.sigma.4.0.mm.3D_glrlm_GrayLevelVariance	2,911062	0,020332	0,051535	n.s.
log.sigma.2.0.mm.3D_glcm_Imc2	2,898248	0,020761	0,052513	n.s.
wavelet.LLL_glcm_ClusterTendency	2,895386	0,020858	0,052648	n.s.
wavelet.LLL_firstorder_InterquartileRange	2,887788	0,021118	0,052942	n.s.
log.sigma.1.0.mm.3D_glrlm_LongRunHighGrayLevelEmphasis	2,888175	0,021105	0,052942	n.s.
log.sigma.1.0.mm.3D_glcm_JointEnergy	2,886273	0,02117	0,052942	n.s.
log.sigma.4.0.mm.3D_glrlm_LongRunLowGrayLevelEmphasis	2,885575	0,021194	0,052942	n.s.
log.sigma.5.0.mm.3D_glcm_Contrast	2,888792	0,021083	0,052942	n.s.
log.sigma.4.0.mm.3D_glcm_Imc2	2,881404	0,021339	0,053193	n.s.
log.sigma.3.0.mm.3D_firstorder_MeanAbsoluteDeviation	2,875729	0,021538	0,053467	n.s.
original_firstorder_RobustMeanAbsoluteDeviation	2,876055	0,021526	0,053467	n.s.
wavelet.LLH_glszm_HighGrayLevelZoneEmphasis	2,873539	0,021615	0,053548	n.s.
original_firstorder_InterquartileRange	2,851222	0,022417	0,055421	n.s.
log.sigma.2.0.mm.3D_glcm_SumSquares	2,849522	0,022479	0,055462	n.s.
wavelet.HLL_gldm_LargeDependenceHighGrayLevelEmphasis	2,847941	0,022538	0,055491	n.s.
wavelet.LLL_glcm_MaximumProbability	2,839718	0,022842	0,056127	n.s.
wavelet.LLH_glcm_InverseVariance	2,836864	0,022949	0,056275	n.s.
original_glcm_ClusterProminence	2,833556	0,023074	0,056465	n.s.
original_glcm_InverseVariance	2,827817	0,023291	0,056882	n.s.
log.sigma.2.0.mm.3D_firstorder_Variance	2,8224	0,023498	0,057272	n.s.
log.sigma.5.0.mm.3D_glcm_DifferenceAverage	2,810765	0,02395	0,058254	n.s.
wavelet.LLH_firstorder_RobustMeanAbsoluteDeviation	2,802502	0,024276	0,058928	n.s.
log.sigma.4.0.mm.3D_gldm_SmallDependenceEmphasis	2,800627	0,024351	0,05899	n.s.
log.sigma.5.0.mm.3D_glcm_Idm	2,795129	0,024571	0,059404	n.s.
log.sigma.4.0.mm.3D_firstorder_Variance	2,784317	0,02501	0,060223	n.s.
log.sigma.5.0.mm.3D_glcm_Id	2,784702	0,024994	0,060223	n.s.
wavelet.LHL_firstorder_Skewness	2,722371	0,025214	0,060535	n.s.

log.sigma.1.0.mm.3D_glcm_Idmn	2,778726	0,02524	0,060535	n.s.
wavelet.LHL_gldm_LargeDependenceHighGrayLevelEmphasis	2,773175	0,02547	0,060966	n.s.
log.sigma.4.0.mm.3D_glszm_ZoneEntropy	2,76418	0,025848	0,061749	n.s.
log.sigma.3.0.mm.3D_firstorder_Range	2,757372	0,026138	0,062318	n.s.
wavelet.LHL_glszm_HighGrayLevelZoneEmphasis	2,754954	0,026242	0,062442	n.s.
log.sigma.3.0.mm.3D_firstorder_RobustMeanAbsoluteDeviation	2,745559	0,02665	0,063286	n.s.
wavelet.HLL_gldm_SmallDependenceEmphasis	2,741318	0,026835	0,063477	n.s.
log.sigma.5.0.mm.3D_glcm_InverseVariance	2,742316	0,026792	0,063477	n.s.
log.sigma.1.0.mm.3D_gldm_LargeDependenceEmphasis	2,73763	0,026998	0,063737	n.s.
wavelet.HLH_glrlm_RunLengthNonUniformity	2,731087	0,02729	0,064299	n.s.
wavelet.HLL_glrlm_LongRunLowGrayLevelEmphasis	2,723983	0,02761	0,064926	n.s.
log.sigma.2.0.mm.3D_glrlm_RunEntropy	2,713116	0,028106	0,065965	n.s.
wavelet.LLL_glszm_LargeAreaLowGrayLevelEmphasis	2,703071	0,028574	0,066802	n.s.
log.sigma.4.0.mm.3D_firstorder_InterquartileRange	2,70329	0,028564	0,066802	n.s.
log.sigma.4.0.mm.3D_firstorder_MeanAbsoluteDeviation	2,696563	0,028881	0,067388	n.s.
log.sigma.2.0.mm.3D_glcm_Idn	2,687701	0,029304	0,068244	n.s.
wavelet.HLH_glcm_MaximumProbability	2,68334	0,029515	0,068602	n.s.
wavelet.HLL_glcm_MaximumProbability	2,67684	0,029832	0,069205	n.s.
log.sigma.5.0.mm.3D_firstorder_Entropy	2,669425	0,030198	0,069919	n.s.
wavelet.LHL_gldm_SmallDependenceLowGrayLevelEmphasis	2,659287	0,030705	0,070552	n.s.
log.sigma.1.0.mm.3D_gldm_LargeDependenceHighGrayLevelEmphasis	2,661256	0,030606	0,070552	n.s.
log.sigma.2.0.mm.3D_glcm_ClusterTendency	2,661998	0,030569	0,070552	n.s.
log.sigma.3.0.mm.3D_glrlm_ShortRunLowGrayLevelEmphasis	2,659433	0,030698	0,070552	n.s.
log.sigma.2.0.mm.3D_glszm_GrayLevelVariance	2,653599	0,030994	0,070944	n.s.
log.sigma.2.0.mm.3D_gldm_DependenceVariance	2,65459	0,030943	0,070944	n.s.
wavelet.HLL_glcm_Imc1	2,651131	0,03112	0,071097	n.s.
log.sigma.4.0.mm.3D_firstorder_RobustMeanAbsoluteDeviation	2,644855	0,031443	0,071699	n.s.
wavelet.HLL_firstorder_Median	2,63784	0,031808	0,07213	n.s.
log.sigma.3.0.mm.3D_glszm_SizeZoneNonUniformityNormalized	2,637768	0,031811	0,07213	n.s.
log.sigma.5.0.mm.3D_glcm_JointEntropy	2,63791	0,031804	0,07213	n.s.
wavelet.HLH_glcm_Idm	2,63177	0,032127	0,072708	n.s.
log.sigma.5.0.mm.3D_glrlm_ShortRunEmphasis	2,625386	0,032466	0,073338	n.s.
wavelet.LHL_glszm_SmallAreaHighGrayLevelEmphasis	2,620726	0,032716	0,073626	n.s.
original_gldm_LargeDependenceEmphasis	2,621596	0,032669	0,073626	n.s.
wavelet.LLH_glrlm_RunLengthNonUniformity	2,612738	0,033149	0,074462	n.s.
log.sigma.5.0.mm.3D_glcm_SumEntropy	2,606061	0,033515	0,075145	n.s.
wavelet.HLH_glrlm_GrayLevelVariance	2,599205	0,033896	0,075857	n.s.
wavelet.HLH_glcm_Id	2,590427	0,03439	0,076818	n.s.
wavelet.LHL_gldm_LargeDependenceLowGrayLevelEmphasis	2,573672	0,035352	0,078677	n.s.
log.sigma.4.0.mm.3D_glcm_Idn	2,57368	0,035352	0,078677	n.s.
wavelet.HHH_glszm_SmallAreaEmphasis	2,569158	0,035616	0,078972	n.s.
log.sigma.3.0.mm.3D_glrlm_LowGrayLevelRunEmphasis	2,569418	0,035601	0,078972	n.s.
wavelet.HLL_firstorder_90Percentile	2,56752	0,035712	0,07904	n.s.
log.sigma.2.0.mm.3D_gldm_LargeDependenceLowGrayLevelEmphasis	2,56139	0,036075	0,079696	n.s.
wavelet.HLH_glcm_DifferenceAverage	2,543713	0,037142	0,081903	n.s.
wavelet.LLH_firstorder_InterquartileRange	2,540875	0,037316	0,082137	n.s.

wavelet.HLL_glcm_ClusterProminence	2,53563	0,03764	0,082699	n.s.
wavelet.HLH_glrlm_ShortRunEmphasis	2,534406	0,037716	0,082715	n.s.
log.sigma.3.0.mm.3D_firstorder_InterquartileRange	2,529431	0,038027	0,083101	n.s.
log.sigma.3.0.mm.3D_glcm_Imc2	2,529383	0,03803	0,083101	n.s.
wavelet.LLH_firstorder_Minimum	2,527692	0,038137	0,083182	n.s.
wavelet.LHH_firstorder_Median	2,524747	0,038322	0,083285	n.s.
log.sigma.4.0.mm.3D_gldm_GrayLevelNonUniformity	2,524907	0,038312	0,083285	n.s.
wavelet.HLL_firstorder_Kurtosis	2,520205	0,03861	0,08376	n.s.
log.sigma.5.0.mm.3D_gldm_SmallDependenceHighGrayLevelEmphasis	2,517738	0,038768	0,083951	n.s.
wavelet.LLH_glrlm_ShortRunHighGrayLevelEmphasis	2,512068	0,039132	0,084588	n.s.
log.sigma.3.0.mm.3D_glszm_ZoneEntropy	2,504705	0,039611	0,085468	n.s.
wavelet.LHL_firstorder_Minimum	2,492807	0,040396	0,087008	n.s.
wavelet.HLL_glszm_LowGrayLevelZoneEmphasis	2,487925	0,040723	0,087478	n.s.
log.sigma.5.0.mm.3D_firstorder_Uniformity	2,487377	0,04076	0,087478	n.s.
original_gldm_DependenceNonUniformityNormalized	2,483583	0,041016	0,087871	n.s.
wavelet.HLH_glcm_DifferenceVariance	2,481412	0,041164	0,08803	n.s.
wavelet.LLL_glrlm_LongRunLowGrayLevelEmphasis	2,475336	0,041579	0,08876	n.s.
log.sigma.5.0.mm.3D_glcm_DifferenceVariance	2,472197	0,041795	0,089043	n.s.
log.sigma.5.0.mm.3D_glcm_DifferenceEntropy	2,471263	0,041859	0,089043	n.s.
log.sigma.5.0.mm.3D_gldm_DependenceEntropy	2,457604	0,042814	0,090915	n.s.
original_firstorder_Skewness	2,456309	0,042906	0,090949	n.s.
wavelet.HLH_glszm_LargeAreaEmphasis	2,447881	0,043508	0,091901	n.s.
log.sigma.3.0.mm.3D_gldm_GrayLevelVariance	2,448569	0,043458	0,091901	n.s.
log.sigma.5.0.mm.3D_glszm_LargeAreaEmphasis	2,435393	0,044415	0,093652	n.s.
log.sigma.3.0.mm.3D_glrlm_RunEntropy	2,433079	0,044585	0,093847	n.s.
log.sigma.5.0.mm.3D_glrlm_GrayLevelNonUniformityNormalized	2,420789	0,0455	0,095605	n.s.
wavelet.HHL_glcm_ClusterShade	2,417997	0,04571	0,09588	n.s.
log.sigma.4.0.mm.3D_firstorder_Range	2,416428	0,045829	0,095962	n.s.
wavelet.LLL_glrlm_LowGrayLevelRunEmphasis	2,412278	0,046145	0,096455	n.s.
wavelet.LHH_glszm_GrayLevelNonUniformity	2,409298	0,046372	0,096763	n.s.
log.sigma.3.0.mm.3D_glcm_SumSquares	2,407459	0,046514	0,09689	n.s.
wavelet.LLL_gldm_LowGrayLevelEmphasis	2,406369	0,046597	0,096897	n.s.
wavelet.LLH_glrlm_HighGrayLevelRunEmphasis	2,404788	0,046719	0,096983	n.s.
log.sigma.3.0.mm.3D_glrlm_GrayLevelVariance	2,402043	0,046932	0,097257	n.s.
log.sigma.2.0.mm.3D_glszm_LargeAreaEmphasis	2,397308	0,047301	0,097853	n.s.
wavelet.HLL_glcm_Correlation	2,391262	0,047776	0,098329	n.s.
log.sigma.1.0.mm.3D_glrlm_LongRunLowGrayLevelEmphasis	2,391714	0,04774	0,098329	n.s.
log.sigma.4.0.mm.3D_glrlm_RunVariance	2,393317	0,047614	0,098329	n.s.
wavelet.HLH_gldm_GrayLevelVariance	2,387826	0,048048	0,09872	n.s.
wavelet.LLL_glcm_ClusterProminence	2,385003	0,048273	0,099013	n.s.
wavelet.HHL_glszm_GrayLevelNonUniformity	2,383433	0,048399	0,099102	n.s.
wavelet.LLL_gldm_LargeDependenceLowGrayLevelEmphasis	2,381047	0,04859	0,099325	n.s.
wavelet.LLL_glrlm_ShortRunLowGrayLevelEmphasis	2,371299	0,04938	0,100708	n.s.
log.sigma.1.0.mm.3D_glszm_SmallAreaEmphasis	2,370632	0,049434	0,100708	n.s.
wavelet.HLH_glcm_SumSquares	2,368119	0,04964	0,100957	n.s.
wavelet.LHH_glszm_ZonePercentage	2,363379	0,050031	0,10158	n.s.

log.sigma.5.0.mm.3D_gldm_DependenceNonUniformityNormalized	2,360692	0,050254	0,101861	n.s.
log.sigma.3.0.mm.3D_glrlm_GrayLevelNonUniformity	2,358967	0,050397	0,10198	n.s.
wavelet.LLH_glcm_JointAverage	2,351084	0,051059	0,102741	n.s.
wavelet.LLH_gldm_HighGrayLevelEmphasis	2,349938	0,051156	0,102741	n.s.
wavelet.LHH_firstorder_RootMeanSquared	2,348707	0,05126	0,102741	n.s.
wavelet.HLH_glcm_ClusterTendency	2,348411	0,051285	0,102741	n.s.
log.sigma.1.0.mm.3D_glcm_Imc1	2,351599	0,051015	0,102741	n.s.
log.sigma.4.0.mm.3D_glrlm_LowGrayLevelRunEmphasis	2,351346	0,051037	0,102741	n.s.
wavelet.HLL_gldm_LowGrayLevelEmphasis	2,346619	0,051437	0,102875	n.s.
original_glcm_MaximumProbability	2,345525	0,05153	0,10289	n.s.
wavelet.HLH_glcm_Contrast	2,343603	0,051695	0,103047	n.s.
wavelet.HLH_glrlm_RunPercentage	2,342552	0,051785	0,103056	n.s.
wavelet.HLL_gldm_SmallDependenceHighGrayLevelEmphasis	2,339798	0,052021	0,103355	n.s.
log.sigma.4.0.mm.3D_glrlm_ShortRunHighGrayLevelEmphasis	2,332167	0,052682	0,104496	n.s.
wavelet.LHL_gldm_SmallDependenceHighGrayLevelEmphasis	2,327221	0,053115	0,105181	n.s.
log.sigma.3.0.mm.3D_gldm_LowGrayLevelEmphasis	2,317364	0,053988	0,106736	n.s.
log.sigma.5.0.mm.3D_glrlm_RunVariance	2,314742	0,054223	0,107024	n.s.
wavelet.HLL_glrlm_LowGrayLevelRunEmphasis	2,301828	0,055394	0,109157	n.s.
wavelet.LHH_firstorder_Mean	2,289087	0,056575	0,111301	n.s.
log.sigma.3.0.mm.3D_glcm_ClusterTendency	2,284698	0,056987	0,111929	n.s.
wavelet.HHL_glszm_ZoneVariance	2,277275	0,057692	0,112945	n.s.
log.sigma.1.0.mm.3D_glrlm_ShortRunLowGrayLevelEmphasis	2,27732	0,057687	0,112945	n.s.
wavelet.LHL_firstorder_Mean	2,272509	0,058149	0,113654	n.s.
log.sigma.1.0.mm.3D_glrlm_LowGrayLevelRunEmphasis	2,24996	0,06036	0,117761	n.s.
log.sigma.1.0.mm.3D_gldm_DependenceNonUniformity	2,249106	0,060445	0,117761	n.s.
log.sigma.1.0.mm.3D_gldm_DependenceNonUniformityNormalized	2,242995	0,06106	0,118766	n.s.
wavelet.HLH_glszm_GrayLevelNonUniformity	2,236318	0,061738	0,119892	n.s.
log.sigma.5.0.mm.3D_glrlm_ShortRunHighGrayLevelEmphasis	2,229706	0,062418	0,121016	n.s.
log.sigma.5.0.mm.3D_glcm_MaximumProbability	2,216668	0,063779	0,123457	n.s.
log.sigma.3.0.mm.3D_firstorder_Variance	2,210161	0,06447	0,124594	n.s.
wavelet.HLL_glszm_ZonePercentage	2,207597	0,064744	0,124923	n.s.
log.sigma.4.0.mm.3D_glcm_ClusterProminence	2,204989	0,065024	0,125263	n.s.
log.sigma.5.0.mm.3D_glcm_Imc2	2,201433	0,065408	0,125801	n.s.
wavelet.HHH_firstorder_Median	2,158144	0,066485	0,127611	n.s.
log.sigma.4.0.mm.3D_glszm_HighGrayLevelZoneEmphasis	2,190049	0,066652	0,127611	n.s.
log.sigma.5.0.mm.3D_glcm_JointEnergy	2,189914	0,066667	0,127611	n.s.
wavelet.HHH_glszm_ZonePercentage	2,18833	0,066842	0,127743	n.s.
wavelet.LLH_glcm_Autocorrelation	2,184854	0,067228	0,128276	n.s.
log.sigma.1.0.mm.3D_gldm_LowGrayLevelEmphasis	2,182947	0,06744	0,128464	n.s.
log.sigma.5.0.mm.3D_gldm_GrayLevelNonUniformity	2,18206	0,06754	0,128464	n.s.
wavelet.LHL_firstorder_RootMeanSquared	2,17939	0,067839	0,128818	n.s.
log.sigma.3.0.mm.3D_gldm_DependenceVariance	2,178491	0,06794	0,128818	n.s.
log.sigma.2.0.mm.3D_glszm_ZoneVariance	2,177208	0,068084	0,128889	n.s.
log.sigma.1.0.mm.3D_gldm_LargeDependenceLowGrayLevelEmphasis	2,169727	0,068932	0,13029	n.s.
log.sigma.4.0.mm.3D_glrlm_HighGrayLevelRunEmphasis	2,159949	0,070057	0,132207	n.s.
log.sigma.5.0.mm.3D_glszm_LargeAreaLowGrayLevelEmphasis	2,156011	0,070515	0,132863	n.s.

wavelet.HLH_glszm_SmallAreaLowGrayLevelEmphasis	2,146334	0,071653	0,134797	n.s.
wavelet.HLL_glrlm_ShortRunLowGrayLevelEmphasis	2,145373	0,071767	0,134801	n.s.
log.sigma.4.0.mm.3D_glszm_SizeZoneNonUniformityNormalized	2,14044	0,072355	0,135694	n.s.
wavelet.HHL_glszm_LargeAreaEmphasis	2,134344	0,073089	0,136856	n.s.
wavelet.LHH_gldm_SmallDependenceHighGrayLevelEmphasis	2,129498	0,073677	0,137744	n.s.
wavelet.HLH_glrlm_LongRunEmphasis	2,127885	0,073874	0,137898	n.s.
wavelet.HLH_glszm_ZoneVariance	2,126226	0,074077	0,138063	n.s.
wavelet.HLL_firstorder_Maximum	2,123905	0,074362	0,13838	n.s.
wavelet.HLH_glszm_GrayLevelVariance	2,11104	0,075961	0,141138	n.s.
log.sigma.4.0.mm.3D_gldm_SmallDependenceLowGrayLevelEmphasis	2,102315	0,077065	0,142969	n.s.
log.sigma.2.0.mm.3D_glszm_GrayLevelNonUniformityNormalized	2,096434	0,077818	0,144144	n.s.
wavelet.LLH_gldm_SmallDependenceHighGrayLevelEmphasis	2,088647	0,078827	0,145788	n.s.
wavelet.HHH_glszm_SizeZoneNonUniformityNormalized	2,053786	0,079502	0,146586	n.s.
log.sigma.4.0.mm.3D_glszm_GrayLevelNonUniformityNormalized	2,083526	0,079497	0,146586	n.s.
wavelet.HLH_glrlm_RunLengthNonUniformityNormalized	2,07025	0,081261	0,149601	n.s.
wavelet.HLL_glszm_SmallAreaHighGrayLevelEmphasis	2,061984	0,082379	0,151427	n.s.
log.sigma.2.0.mm.3D_glrlm_LowGrayLevelRunEmphasis	2,05905	0,08278	0,151931	n.s.
wavelet.HHH_gldm_DependenceVariance	2,055183	0,083311	0,152673	n.s.
log.sigma.4.0.mm.3D_glszm_ZonePercentage	2,049973	0,084031	0,153759	n.s.
wavelet.LLL_firstorder_Skewness	2,046546	0,084508	0,154397	n.s.
log.sigma.4.0.mm.3D_glszm_LargeAreaLowGrayLevelEmphasis	2,043542	0,084929	0,15493	n.s.
wavelet.LLH_gldm_DependenceNonUniformity	2,033143	0,0864	0,157377	n.s.
log.sigma.2.0.mm.3D_gldm_LowGrayLevelEmphasis	2,015211	0,088997	0,161862	n.s.
wavelet.HHH_firstorder_Mean	2,007049	0,090205	0,16381	n.s.
log.sigma.3.0.mm.3D_glcm_ClusterProminence	2,003695	0,090706	0,164472	n.s.
log.sigma.3.0.mm.3D_gldm_SmallDependenceEmphasis	1,986638	0,093295	0,168914	n.s.
log.sigma.3.0.mm.3D_glszm_LargeAreaEmphasis	1,982149	0,093989	0,169913	n.s.
wavelet.HHH_firstorder_RootMeanSquared	1,980682	0,094216	0,17007	n.s.
wavelet.LLH_glcm_Imc1	1,975368	0,095046	0,17131	n.s.
log.sigma.4.0.mm.3D_glszm_SizeZoneNonUniformity	1,974141	0,095239	0,171401	n.s.
wavelet.LHL_glcm_Idmn	1,969278	0,096005	0,17201	n.s.
log.sigma.4.0.mm.3D_glcm_ClusterShade	1,969287	0,096004	0,17201	n.s.
log.sigma.4.0.mm.3D_gldm_LargeDependenceLowGrayLevelEmphasis	1,970507	0,095811	0,17201	n.s.
wavelet.LLH_glszm_SmallAreaHighGrayLevelEmphasis	1,965743	0,096567	0,172247	n.s.
wavelet.LHL_glszm_SmallAreaLowGrayLevelEmphasis	1,967425	0,096299	0,172247	n.s.
log.sigma.4.0.mm.3D_gldm_SmallDependenceHighGrayLevelEmphasis	1,966157	0,096501	0,172247	n.s.
wavelet.HLL_gldm_SmallDependenceLowGrayLevelEmphasis	1,956026	0,098126	0,17477	n.s.
wavelet.LHL_gldm_DependenceNonUniformity	1,947447	0,099524	0,176216	n.s.
wavelet.LHH_glcm_DifferenceVariance	1,949908	0,099121	0,176216	n.s.
wavelet.HLH_firstorder_Maximum	1,948567	0,09934	0,176216	n.s.
log.sigma.5.0.mm.3D_glrlm_LongRunLowGrayLevelEmphasis	1,948107	0,099416	0,176216	n.s.
wavelet.LHH_glcm_DifferenceEntropy	1,943742	0,100133	0,177034	n.s.
wavelet.LHH_glszm_GrayLevelVariance	1,939975	0,100757	0,177876	n.s.
wavelet.HLH_gldm_LargeDependenceEmphasis	1,933927	0,101766	0,179394	n.s.
wavelet.HHH_gldm_SmallDependenceEmphasis	1,932971	0,101926	0,179414	n.s.
log.sigma.2.0.mm.3D_glcm_ClusterProminence	1,929336	0,102538	0,179965	n.s.

log.sigma.3.0.mm.3D_glszm_HighGrayLevelZoneEmphasis	1,930075	0,102414	0,179965	n.s.
log.sigma.3.0.mm.3D_gldm_SmallDependenceLowGrayLevelEmphasis	1,924205	0,103408	0,181228	n.s.
wavelet.LLH_glszm_SmallAreaLowGrayLevelEmphasis	1,916784	0,10468	0,183189	n.s.
wavelet.HHH_glszm_SizeZoneNonUniformity	1,914389	0,105093	0,183646	n.s.
log.sigma.3.0.mm.3D_glszm_ZoneVariance	1,913386	0,105267	0,183683	n.s.
wavelet.LHL_glrlm_RunLengthNonUniformity	1,910692	0,105735	0,184232	n.s.
log.sigma.4.0.mm.3D_gldm_HighGrayLevelEmphasis	1,904892	0,106749	0,185731	n.s.
log.sigma.2.0.mm.3D_gldm_LargeDependenceHighGrayLevelEmphasis	1,902972	0,107087	0,186049	n.s.
wavelet.LHH_glcm_MaximumProbability	1,867489	0,11352	0,196943	n.s.
log.sigma.3.0.mm.3D_glszm_LargeAreaLowGrayLevelEmphasis	1,854225	0,11602	0,200991	n.s.
wavelet.LHH_glszm_SmallAreaHighGrayLevelEmphasis	1,851664	0,116509	0,201548	n.s.
wavelet.HLL_glcm_InverseVariance	1,846125	0,117573	0,203097	n.s.
log.sigma.1.0.mm.3D_firstorder_Skewness	1,843111	0,118156	0,203811	n.s.
wavelet.LLL_glszm_LowGrayLevelZoneEmphasis	1,83885	0,118986	0,204948	n.s.
log.sigma.4.0.mm.3D_glrlm_RunEntropy	1,827505	0,12122	0,208499	n.s.
wavelet.LHH_glszm_SmallAreaEmphasis	1,824162	0,121887	0,209346	n.s.
wavelet.LLH_gldm_LargeDependenceLowGrayLevelEmphasis	1,823214	0,122076	0,209373	n.s.
wavelet.HHL_firstorder_Skewness	1,820629	0,122595	0,209963	n.s.
log.sigma.5.0.mm.3D_glcm_Imc1	1,814459	0,123841	0,211796	n.s.
wavelet.LLH_glcm_MaximumProbability	1,811069	0,124531	0,212629	n.s.
log.sigma.4.0.mm.3D_glcm_JointAverage	1,810333	0,124681	0,212629	n.s.
wavelet.LLH_glrlm_LongRunHighGrayLevelEmphasis	1,80878	0,124999	0,212869	n.s.
wavelet.LLH_glszm_GrayLevelNonUniformityNormalized	1,804544	0,125869	0,213746	n.s.
wavelet.LHL_glcm_ClusterShade	1,783634	0,125851	0,213746	n.s.
log.sigma.5.0.mm.3D_gldm_SmallDependenceEmphasis	1,802052	0,126384	0,214318	n.s.
wavelet.HHH_glszm_GrayLevelNonUniformity	1,800723	0,126659	0,214483	n.s.
log.sigma.3.0.mm.3D_gldm_DependenceNonUniformity	1,797557	0,127317	0,215295	n.s.
log.sigma.5.0.mm.3D_glcm_ClusterShade	1,795415	0,127765	0,215748	n.s.
wavelet.LHH_glcm_Contrast	1,792027	0,128475	0,216644	n.s.
wavelet.LLH_glszm_LowGrayLevelZoneEmphasis	1,786165	0,129713	0,218181	n.s.
log.sigma.1.0.mm.3D_glcm_Idn	1,78584	0,129782	0,218181	n.s.
log.sigma.4.0.mm.3D_gldm_LowGrayLevelEmphasis	1,785145	0,12993	0,218181	n.s.
log.sigma.2.0.mm.3D_firstorder_Maximum	1,784075	0,130158	0,218259	n.s.
log.sigma.1.0.mm.3D_glcm_ClusterShade	1,781079	0,130797	0,219026	n.s.
wavelet.LLH_glrlm_LongRunLowGrayLevelEmphasis	1,777987	0,13146	0,21967	n.s.
original_firstorder_Minimum	1,777585	0,131547	0,21967	n.s.
log.sigma.3.0.mm.3D_glszm_LargeAreaHighGrayLevelEmphasis	1,638071	0,132066	0,220232	n.s.
log.sigma.2.0.mm.3D_glcm_Correlation	1,766517	0,133948	0,223062	n.s.
log.sigma.3.0.mm.3D_glszm_ZonePercentage	1,760178	0,135343	0,225073	n.s.
wavelet.LLH_firstorder_Range	1,754575	0,136587	0,226516	n.s.
log.sigma.5.0.mm.3D_glszm_SmallAreaLowGrayLevelEmphasis	1,755006	0,136491	0,226516	n.s.
log.sigma.5.0.mm.3D_glcm_Idn	1,749047	0,137825	0,228255	n.s.
log.sigma.2.0.mm.3D_firstorder_90Percentile	1,739874	0,139904	0,23138	n.s.
log.sigma.3.0.mm.3D_glrlm_ShortRunHighGrayLevelEmphasis	1,738137	0,140301	0,231718	n.s.
original_glszm_SmallAreaLowGrayLevelEmphasis	1,736286	0,140725	0,2321	n.s.
wavelet.LHL_firstorder_Energy	1,601882	0,142005	0,232934	n.s.

wavelet.LHL_firstorder_TotalEnergy	1,601882	0,142005	0,232934	n.s.
log.sigma.1.0.mm.3D_gldm_SmallDependenceLowGrayLevelEmphasis	1,733178	0,14144	0,232934	n.s.
log.sigma.3.0.mm.3D_glcm_JointAverage	1,732317	0,141639	0,232934	n.s.
log.sigma.4.0.mm.3D_glcm_Imc1	1,719972	0,144517	0,236733	n.s.
wavelet.HHH_glszm_LargeAreaLowGrayLevelEmphasis	1,709286	0,147054	0,240293	n.s.
log.sigma.2.0.mm.3D_glrlm_ShortRunLowGrayLevelEmphasis	1,709137	0,14709	0,240293	n.s.
log.sigma.4.0.mm.3D_glszm_LowGrayLevelZoneEmphasis	1,707587	0,147461	0,240574	n.s.
original_glszm_SizeZoneNonUniformity	1,69779	0,149831	0,244109	n.s.
wavelet.LHH_glcm_DifferenceAverage	1,688872	0,152019	0,247339	n.s.
wavelet.LHL_firstorder_Median	1,684122	0,153197	0,248919	n.s.
log.sigma.2.0.mm.3D_glszm_LargeAreaLowGrayLevelEmphasis	1,676719	0,15505	0,251591	n.s.
wavelet.LLL_glcm_Idmn	1,664091	0,15826	0,256152	n.s.
log.sigma.2.0.mm.3D_glrlm_LongRunHighGrayLevelEmphasis	1,663989	0,158287	0,256152	n.s.
log.sigma.3.0.mm.3D_glrlm_HighGrayLevelRunEmphasis	1,658045	0,15982	0,258286	n.s.
wavelet.HLL_glszm_SizeZoneNonUniformity	1,655936	0,160367	0,258554	n.s.
wavelet.LLL_firstorder_Kurtosis	1,655751	0,160415	0,258554	n.s.
wavelet.HLH_glrlm_RunVariance	1,640667	0,164383	0,264595	n.s.
wavelet.LHH_glcm_JointEntropy	1,631472	0,166847	0,268009	n.s.
log.sigma.1.0.mm.3D_glcm_InverseVariance	1,630847	0,167016	0,268009	n.s.
log.sigma.3.0.mm.3D_gldm_HighGrayLevelEmphasis	1,63027	0,167172	0,268009	n.s.
wavelet.HLL_gldm_DependenceVariance	1,624779	0,168662	0,270039	n.s.
log.sigma.2.0.mm.3D_gldm_SmallDependenceLowGrayLevelEmphasis	1,62395	0,168888	0,270042	n.s.
wavelet.HHH_firstorder_Energy	1,621475	0,169565	0,270406	n.s.
wavelet.HHH_firstorder_TotalEnergy	1,621475	0,169565	0,270406	n.s.
wavelet.HLH_gldm_DependenceEntropy	1,617534	0,170648	0,271773	n.s.
log.sigma.4.0.mm.3D_glcm_Autocorrelation	1,613126	0,171868	0,273353	n.s.
log.sigma.5.0.mm.3D_glszm_ZoneEntropy	1,589704	0,178484	0,283502	n.s.
wavelet.LHH_glcm_Idm	1,588801	0,178744	0,283541	n.s.
wavelet.LHL_glszm_LargeAreaHighGrayLevelEmphasis	1,587857	0,179016	0,283598	n.s.
wavelet.LHL_glcm_Idn	1,585845	0,179597	0,284145	n.s.
wavelet.LLL_glcm_Idn	1,578514	0,181728	0,286764	n.s.
log.sigma.4.0.mm.3D_glrlm_GrayLevelNonUniformity	1,578627	0,181695	0,286764	n.s.
wavelet.HLH_glcm_ClusterProminence	1,572961	0,183359	0,288958	n.s.
wavelet.LHH_glcm_Id	1,571662	0,183742	0,289184	n.s.
log.sigma.1.0.mm.3D_glszm_SizeZoneNonUniformity	1,570532	0,184077	0,289332	n.s.
wavelet.LHH_glcm_ClusterShade	1,553797	0,184686	0,289911	n.s.
log.sigma.2.0.mm.3D_glcm_ClusterShade	1,56272	0,186402	0,292224	n.s.
wavelet.LHL_firstorder_90Percentile	1,557694	0,187912	0,293827	n.s.
wavelet.HHH_glszm_SmallAreaLowGrayLevelEmphasis	1,558104	0,187789	0,293827	n.s.
wavelet.LLH_glszm_SizeZoneNonUniformity	1,551992	0,18964	0,296143	n.s.
wavelet.LHH_firstorder_Skewness	1,541139	0,192969	0,300951	n.s.
wavelet.HHH_gldm_LargeDependenceHighGrayLevelEmphasis	1,539048	0,193616	0,301571	n.s.
wavelet.LHH_glcm_SumSquares	1,536483	0,194413	0,302421	n.s.
wavelet.LLL_gldm_DependenceVariance	1,524116	0,198299	0,308068	n.s.
wavelet.LHH_glszm_SizeZoneNonUniformityNormalized	1,518423	0,200112	0,310484	n.s.
log.sigma.3.0.mm.3D_glcm_Autocorrelation	1,511913	0,202204	0,313326	n.s.

wavelet.HHH_gldm_LargeDependenceLowGrayLevelEmphasis	1,506218	0,204051	0,315375	n.s.
log.sigma.3.0.mm.3D_gldm_SmallDependenceHighGrayLevelEmphasis	1,506787	0,203866	0,315375	n.s.
wavelet.LHL_glrlm_RunEntropy	1,503789	0,204843	0,316194	n.s.
wavelet.LHH_glcm_JointEnergy	1,501963	0,205441	0,316563	n.s.
log.sigma.5.0.mm.3D_glcm_Correlation	1,501452	0,205608	0,316563	n.s.
wavelet.HHH_glrlm_LongRunHighGrayLevelEmphasis	1,492228	0,208653	0,320841	n.s.
wavelet.HHH_gldm_LowGrayLevelEmphasis	1,488109	0,210026	0,32254	n.s.
wavelet.HHL_gldm_SmallDependenceEmphasis	1,484913	0,211097	0,323772	n.s.
log.sigma.1.0.mm.3D_glszm_SmallAreaLowGrayLevelEmphasis	1,481624	0,212205	0,325056	n.s.
wavelet.HLH_gldm_SmallDependenceHighGrayLevelEmphasis	1,477971	0,213441	0,326535	n.s.
wavelet.HHH_glcm_JointAverage	1,476306	0,214007	0,326985	n.s.
wavelet.HHH_gldm_HighGrayLevelEmphasis	1,474501	0,214622	0,327094	n.s.
log.sigma.4.0.mm.3D_gldm_DependenceVariance	1,475182	0,214389	0,327094	n.s.
wavelet.HHH_firstorder_Minimum	1,471882	0,215516	0,327746	n.s.
wavelet.HHH_glrlm_LongRunLowGrayLevelEmphasis	1,470425	0,216016	0,327746	n.s.
wavelet.HHH_glcm_Autocorrelation	1,470068	0,216138	0,327746	n.s.
wavelet.HHH_glszm_LargeAreaEmphasis	1,38583	0,215923	0,327746	n.s.
log.sigma.3.0.mm.3D_gldm_LargeDependenceHighGrayLevelEmphasis	1,462464	0,218763	0,331309	n.s.
wavelet.HHL_firstorder_Maximum	1,460322	0,219507	0,331602	n.s.
log.sigma.4.0.mm.3D_firstorder_Maximum	1,461012	0,219267	0,331602	n.s.
wavelet.HHH_gldm_LargeDependenceEmphasis	1,459064	0,219945	0,331848	n.s.
wavelet.HHH_firstorder_Range	1,451758	0,222507	0,334507	n.s.
wavelet.HHH_glrlm_HighGrayLevelRunEmphasis	1,452538	0,222232	0,334507	n.s.
log.sigma.5.0.mm.3D_glrlm_HighGrayLevelRunEmphasis	1,45166	0,222541	0,334507	n.s.
wavelet.HHH_glrlm_LowGrayLevelRunEmphasis	1,450249	0,22304	0,334837	n.s.
wavelet.HHH_firstorder_Skewness	1,429281	0,226342	0,339372	n.s.
wavelet.HHL_firstorder_Range	1,437304	0,227655	0,340916	n.s.
wavelet.HHH_gldm_DependenceEntropy	1,435176	0,228422	0,341639	n.s.
wavelet.HHH_glrlm_ShortRunHighGrayLevelEmphasis	1,424322	0,23237	0,347113	n.s.
wavelet.HHH_glcm_Idn	1,423189	0,232786	0,347303	n.s.
wavelet.HHH_glrlm_ShortRunLowGrayLevelEmphasis	1,420418	0,233805	0,348392	n.s.
wavelet.LHH_glrlm_ShortRunEmphasis	1,418416	0,234544	0,348631	n.s.
log.sigma.5.0.mm.3D_glszm_ZonePercentage	1,418496	0,234515	0,348631	n.s.
wavelet.HLH_firstorder_Range	1,411795	0,237003	0,351852	n.s.
wavelet.LHH_firstorder_Minimum	1,405883	0,239218	0,354703	n.s.
log.sigma.4.0.mm.3D_gldm_DependenceNonUniformity	1,403844	0,239987	0,355405	n.s.
wavelet.LLH_gldm_LargeDependenceHighGrayLevelEmphasis	1,399109	0,24178	0,35762	n.s.
wavelet.LHH_glcm_Imc1	1,397601	0,242353	0,358029	n.s.
wavelet.HHL_glszm_LargeAreaLowGrayLevelEmphasis	1,395589	0,243121	0,358693	n.s.
log.sigma.3.0.mm.3D_glszm_SmallAreaLowGrayLevelEmphasis	1,384189	0,243399	0,358693	n.s.
wavelet.LLH_glrlm_LowGrayLevelRunEmphasis	1,39325	0,244015	0,359161	n.s.
wavelet.HHL_glrlm_GrayLevelNonUniformity	1,389892	0,245304	0,360619	n.s.
log.sigma.4.0.mm.3D_glszm_ZoneVariance	1,315262	0,246155	0,361428	n.s.
wavelet.LHL_glszm_GrayLevelNonUniformityNormalized	1,382415	0,248197	0,363982	n.s.
wavelet.LHH_glcm_SumEntropy	1,373633	0,251633	0,368573	n.s.
wavelet.HLH_glcm_Imc2	1,365389	0,254898	0,372845	n.s.

wavelet.HLH_glszm_LargeAreaHighGrayLevelEmphasis	1,364706	0,25517	0,372845	n.s.
wavelet.HLL_glcm_ClusterShade	1,360332	0,256919	0,374492	n.s.
wavelet.HHH_glrlm_RunVariance	1,360816	0,256725	0,374492	n.s.
wavelet.LHH_glcm_Imc2	1,355171	0,258996	0,377063	n.s.
wavelet.HHL_gldm_DependenceNonUniformity	1,353486	0,259678	0,377143	n.s.
wavelet.LLH_gldm_LowGrayLevelEmphasis	1,354216	0,259382	0,377143	n.s.
wavelet.HHH_gldm_DependenceNonUniformityNormalized	1,350976	0,260695	0,37771	n.s.
log.sigma.1.0.mm.3D_firstorder_Kurtosis	1,351183	0,260612	0,37771	n.s.
wavelet.HHL_glcm_Correlation	1,346734	0,262424	0,379757	n.s.
wavelet.LHL_glszm_GrayLevelVariance	1,337318	0,266297	0,384899	n.s.
wavelet.HHH_glcm_Idmn	1,333126	0,268037	0,38695	n.s.
wavelet.HHL_gldm_GrayLevelNonUniformity	1,331454	0,268734	0,387491	n.s.
wavelet.LLH_glrlm_ShortRunLowGrayLevelEmphasis	1,3287	0,269885	0,388657	n.s.
wavelet.HHH_gldm_SmallDependenceHighGrayLevelEmphasis	1,318704	0,270188	0,388657	n.s.
wavelet.LLH_glszm_ZoneEntropy	1,325221	0,271345	0,388928	n.s.
wavelet.LHL_firstorder_InterquartileRange	1,326249	0,270913	0,388928	n.s.
log.sigma.5.0.mm.3D_glszm_LargeAreaHighGrayLevelEmphasis	1,261685	0,271319	0,388928	n.s.
wavelet.LHH_glrlm_RunLengthNonUniformityNormalized	1,319239	0,273873	0,392085	n.s.
wavelet.LLH_firstorder_90Percentile	1,317813	0,274479	0,392485	n.s.
wavelet.LHL_glszm_LargeAreaLowGrayLevelEmphasis	1,313228	0,276433	0,394811	n.s.
wavelet.LHL_glrlm_RunVariance	1,310477	0,277612	0,396025	n.s.
wavelet.LLL_firstorder_Minimum	1,307165	0,279037	0,397587	n.s.
wavelet.HHL_glrlm_RunLengthNonUniformity	1,302116	0,281222	0,400226	n.s.
wavelet.LHH_glrlm_RunPercentage	1,291974	0,285655	0,406054	n.s.
log.sigma.5.0.mm.3D_gldm_LargeDependenceLowGrayLevelEmphasis	1,290049	0,286503	0,40678	n.s.
log.sigma.2.0.mm.3D_gldm_DependenceNonUniformityNormalized	1,287041	0,287833	0,408187	n.s.
log.sigma.5.0.mm.3D_glcm_ClusterProminence	1,281171	0,290443	0,411404	n.s.
log.sigma.5.0.mm.3D_glrlm_LongRunHighGrayLevelEmphasis	1,279827	0,291044	0,41177	n.s.
log.sigma.5.0.mm.3D_gldm_DependenceNonUniformity	1,275996	0,292761	0,413714	n.s.
wavelet.HHH_gldm_GrayLevelNonUniformity	1,275083	0,293172	0,413808	n.s.
log.sigma.2.0.mm.3D_glszm_LargeAreaHighGrayLevelEmphasis	1,215459	0,29462	0,415366	n.s.
wavelet.HLH_glszm_LargeAreaLowGrayLevelEmphasis	1,267999	0,296375	0,416674	n.s.
wavelet.HHH_firstorder_Variance	1,266772	0,296932	0,416674	n.s.
log.sigma.4.0.mm.3D_gldm_LargeDependenceHighGrayLevelEmphasis	1,2677	0,29651	0,416674	n.s.
log.sigma.5.0.mm.3D_firstorder_Skewness	1,266778	0,296929	0,416674	n.s.
log.sigma.5.0.mm.3D_glszm_LowGrayLevelZoneEmphasis	1,262012	0,299105	0,419234	n.s.
wavelet.LHH_glcm_ClusterTendency	1,260037	0,30001	0,420014	n.s.
wavelet.LLH_gldm_DependenceVariance	1,252808	0,303344	0,423696	n.s.
log.sigma.2.0.mm.3D_firstorder_Kurtosis	1,252994	0,303258	0,423696	n.s.
original_glszm_LargeAreaHighGrayLevelEmphasis	1,1893	0,308471	0,430358	n.s.
wavelet.LHL_gldm_GrayLevelNonUniformity	1,238741	0,309921	0,431881	n.s.
wavelet.HLH_gldm_DependenceNonUniformity	1,229518	0,314299	0,437475	n.s.
wavelet.HHL_glszm_LargeAreaHighGrayLevelEmphasis	1,228563	0,314755	0,437604	n.s.
log.sigma.3.0.mm.3D_glszm_SmallAreaEmphasis	1,224844	0,316537	0,439574	n.s.
wavelet.HHL_glszm_ZonePercentage	1,221229	0,318277	0,441314	n.s.
wavelet.LHL_glrlm_GrayLevelNonUniformityNormalized	1,220719	0,318523	0,441314	n.s.

wavelet.LHL_glrlm_LongRunEmphasis	1,219579	0,319074	0,441569	n.s.
wavelet.HLH_glcm_Imc1	1,215774	0,320919	0,443612	n.s.
log.sigma.3.0.mm.3D_glcm_ClusterShade	1,213801	0,321878	0,444428	n.s.
wavelet.HLL_glszm_LargeAreaHighGrayLevelEmphasis	1,209397	0,324029	0,446885	n.s.
wavelet.LHH_glrlm_LongRunEmphasis	1,207199	0,325108	0,447348	n.s.
original_glcm_ClusterShade	1,207561	0,32493	0,447348	n.s.
log.sigma.3.0.mm.3D_firstorder_Kurtosis	1,198533	0,329387	0,452719	n.s.
wavelet.HHH_firstorder_10Percentile	1,196036	0,330628	0,453907	n.s.
wavelet.LHH_glszm_HighGrayLevelZoneEmphasis	1,193595	0,331846	0,45506	n.s.
wavelet.LLH_glszm_GrayLevelVariance	1,166147	0,345793	0,473543	n.s.
wavelet.LHH_glrlm_GrayLevelVariance	1,165532	0,346111	0,473543	n.s.
wavelet.HHH_glrlm_RunLengthNonUniformity	1,163314	0,34726	0,474575	n.s.
original_firstorder_Kurtosis	1,157158	0,350464	0,478411	n.s.
wavelet.LHH_glszm_GrayLevelNonUniformityNormalized	1,155791	0,351178	0,478843	n.s.
wavelet.LHH_glcm_ClusterProminence	1,153576	0,352339	0,479882	n.s.
wavelet.LHL_firstorder_Uniformity	1,14768	0,355443	0,483563	n.s.
wavelet.HLL_glszm_SmallAreaEmphasis	1,145226	0,356742	0,484782	n.s.
wavelet.LLL_glszm_SizeZoneNonUniformityNormalized	1,14328	0,357774	0,485636	n.s.
wavelet.HLH_glcm_ClusterShade	1,136433	0,358313	0,485821	n.s.
wavelet.LHL_gldm_DependenceVariance	1,138881	0,360116	0,487169	n.s.
wavelet.LHH_gldm_LargeDependenceEmphasis	1,139042	0,36003	0,487169	n.s.
wavelet.HHL_glrlm_LongRunEmphasis	1,137928	0,360626	0,48731	n.s.
original_gldm_SmallDependenceLowGrayLevelEmphasis	1,134714	0,362347	0,489087	n.s.
wavelet.HHH_firstorder_Kurtosis	1,126101	0,366991	0,494801	n.s.
log.sigma.5.0.mm.3D_glszm_ZoneVariance	1,124307	0,367965	0,495559	n.s.
wavelet.HLL_glcm_Idmn	1,121459	0,369514	0,49709	n.s.
wavelet.LHL_glszm_SizeZoneNonUniformity	1,120269	0,370163	0,497407	n.s.
wavelet.LHL_gldm_DependenceEntropy	1,118311	0,371233	0,497733	n.s.
wavelet.LHH_gldm_SmallDependenceLowGrayLevelEmphasis	1,118604	0,371073	0,497733	n.s.
wavelet.HHL_glrlm_ShortRunEmphasis	1,114764	0,373177	0,499783	n.s.
wavelet.HLH_firstorder_Skewness	1,103972	0,379141	0,507207	n.s.
wavelet.HLL_glcm_Idn	1,101691	0,380411	0,508341	n.s.
wavelet.HHL_glrlm_RunVariance	1,097772	0,382601	0,5107	n.s.
wavelet.HHL_glcm_Idn	1,09692	0,383079	0,510771	n.s.
wavelet.LHH_firstorder_Entropy	1,092364	0,385639	0,513617	n.s.
log.sigma.3.0.mm.3D_glrlm_LongRunHighGrayLevelEmphasis	1,088319	0,387924	0,516089	n.s.
wavelet.HHH_glrlm_RunEntropy	1,085133	0,38973	0,517831	n.s.
wavelet.LLL_glszm_SmallAreaEmphasis	1,084365	0,390166	0,517831	n.s.
log.sigma.2.0.mm.3D_glszm_SmallAreaEmphasis	1,083738	0,390523	0,517831	n.s.
wavelet.LHH_firstorder_Range	1,080817	0,392188	0,519465	n.s.
wavelet.HHL_glrlm_RunLengthNonUniformityNormalized	1,079814	0,392761	0,519653	n.s.
wavelet.LHH_gldm_GrayLevelVariance	1,076422	0,394703	0,521649	n.s.
wavelet.HHH_glcm_Id	1,069614	0,395891	0,522646	n.s.
wavelet.LHH_glszm_LargeAreaLowGrayLevelEmphasis	1,073022	0,396657	0,523083	n.s.
wavelet.HHH_glcm_Idm	1,065107	0,39853	0,524978	n.s.
wavelet.HLH_gldm_SmallDependenceLowGrayLevelEmphasis	1,068847	0,399066	0,52511	n.s.

wavelet.HHL_glcm_Idmn	1,062825	0,402561	0,525986	n.s.
wavelet.LHH_firstorder_90Percentile	1,066123	0,400644	0,525986	n.s.
wavelet.HHH_firstorder_Maximum	1,066518	0,400415	0,525986	n.s.
log.sigma.1.0.mm.3D_glcm_MaximumProbability	1,063906	0,401932	0,525986	n.s.
log.sigma.4.0.mm.3D_glszm_GrayLevelNonUniformity	1,062432	0,40279	0,525986	n.s.
log.sigma.4.0.mm.3D_glszm_LargeAreaHighGrayLevelEmphasis	1,032894	0,401448	0,525986	n.s.
log.sigma.5.0.mm.3D_glcm_Idmn	1,06343	0,402209	0,525986	n.s.
wavelet.HHH_glcm_Correlation	1,054805	0,40461	0,52779	n.s.
wavelet.LHL_firstorder_Entropy	1,055262	0,406983	0,530311	n.s.
wavelet.HLH_glszm_GrayLevelNonUniformityNormalized	1,053475	0,408033	0,530671	n.s.
wavelet.HHH_glcm_DifferenceAverage	1,048484	0,408374	0,530671	n.s.
wavelet.HHH_gldm_DependenceNonUniformity	1,052543	0,408582	0,530671	n.s.
wavelet.LHH_glszm_LargeAreaEmphasis	1,049511	0,41037	0,531995	n.s.
log.sigma.4.0.mm.3D_firstorder_Kurtosis	1,049317	0,410484	0,531995	n.s.
wavelet.HHH_glrlm_LongRunEmphasis	1,046336	0,412249	0,533707	n.s.
wavelet.HHL_glrlm_RunPercentage	1,044457	0,413363	0,534575	n.s.
wavelet.HHH_glrlm_RunPercentage	1,043231	0,414092	0,534943	n.s.
wavelet.LHL_glrlm_GrayLevelNonUniformity	1,040679	0,415612	0,536331	n.s.
wavelet.LHL_glszm_GrayLevelNonUniformity	1,039452	0,416344	0,536701	n.s.
wavelet.LHL_firstorder_RobustMeanAbsoluteDeviation	1,037267	0,41765	0,537808	n.s.
log.sigma.5.0.mm.3D_glrlm_RunEntropy	1,030097	0,421957	0,542774	n.s.
wavelet.LHL_gldm_LargeDependenceEmphasis	1,028068	0,423182	0,543769	n.s.
log.sigma.3.0.mm.3D_firstorder_Maximum	1,025224	0,424903	0,545398	n.s.
log.sigma.5.0.mm.3D_glrlm_GrayLevelNonUniformity	1,022645	0,426468	0,546824	n.s.
wavelet.HHH_glcm_InverseVariance	1,014782	0,428877	0,549327	n.s.
wavelet.LHL_glcm_SumEntropy	1,013368	0,432133	0,55291	n.s.
wavelet.LHL_glszm_ZoneVariance	1,00675	0,436207	0,55753	n.s.
wavelet.LHL_glszm_LargeAreaEmphasis	1,005148	0,437197	0,557826	n.s.
wavelet.LHH_gldm_LargeDependenceHighGrayLevelEmphasis	1,004877	0,437365	0,557826	n.s.
wavelet.LHL_glcm_MaximumProbability	1,002088	0,439094	0,559438	n.s.
wavelet.LLH_gldm_SmallDependenceLowGrayLevelEmphasis	1,000352	0,440172	0,559901	n.s.
wavelet.LLL_glszm_LargeAreaHighGrayLevelEmphasis	1,000005	0,440387	0,559901	n.s.
wavelet.HLH_glcm_Correlation	0,996466	0,442592	0,562111	n.s.
wavelet.LLH_glcm_ClusterProminence	0,995368	0,443278	0,562388	n.s.
wavelet.HLH_glszm_SizeZoneNonUniformityNormalized	0,993982	0,444144	0,562894	n.s.
wavelet.LHH_glrlm_GrayLevelNonUniformityNormalized	0,991815	0,445501	0,56402	n.s.
wavelet.LHH_glcm_InverseVariance	0,991	0,446012	0,564074	n.s.
wavelet.HHL_glszm_GrayLevelVariance	0,986767	0,448674	0,566845	n.s.
log.sigma.4.0.mm.3D_glszm_SmallAreaLowGrayLevelEmphasis	0,983764	0,450568	0,568642	n.s.
wavelet.HLH_firstorder_Kurtosis	0,980095	0,452891	0,570975	n.s.
wavelet.HHH_glszm_LargeAreaHighGrayLevelEmphasis	0,949733	0,457809	0,576571	n.s.
wavelet.LHL_gldm_DependenceNonUniformityNormalized	0,968751	0,460125	0,578278	n.s.
log.sigma.4.0.mm.3D_glcm_Idmn	0,968956	0,459993	0,578278	n.s.
wavelet.HHL_glszm_SizeZoneNonUniformity	0,966297	0,4617	0,579048	n.s.
wavelet.LHH_glszm_ZoneVariance	0,966509	0,461563	0,579048	n.s.
log.sigma.5.0.mm.3D_gldm_DependenceVariance	0,964422	0,462905	0,579957	n.s.

wavelet.HHL_gldm_DependenceVariance	0,962665	0,464038	0,58077	n.s.
wavelet.LHL_glszm_SmallAreaEmphasis	0,959615	0,466007	0,582629	n.s.
wavelet.HHL_firstorder_MeanAbsoluteDeviation	0,957846	0,467152	0,583455	n.s.
log.sigma.3.0.mm.3D_glszm_GrayLevelNonUniformityNormalized	0,955665	0,468566	0,584615	n.s.
wavelet.HLH_glcm_JointAverage	0,951953	0,470979	0,58641	n.s.
wavelet.HLH_gldm_LowGrayLevelEmphasis	0,952556	0,470586	0,58641	n.s.
wavelet.HLH_glrlm_LowGrayLevelRunEmphasis	0,943817	0,476297	0,592419	n.s.
log.sigma.5.0.mm.3D_glrlm_LowGrayLevelRunEmphasis	0,942125	0,477408	0,593188	n.s.
wavelet.HHL_glcm_Id	0,940041	0,478778	0,594277	n.s.
wavelet.HHL_firstorder_Variance	0,938194	0,479996	0,595175	n.s.
wavelet.HLH_gldm_LargeDependenceLowGrayLevelEmphasis	0,936496	0,481116	0,595951	n.s.
wavelet.LHL_firstorder_MeanAbsoluteDeviation	0,934927	0,482153	0,596621	n.s.
wavelet.HLH_glrlm_LongRunLowGrayLevelEmphasis	0,933719	0,482952	0,596997	n.s.
log.sigma.3.0.mm.3D_firstorder_Skewness	0,932949	0,483463	0,597014	n.s.
wavelet.HHH_glcm_MaximumProbability	0,930319	0,485206	0,59733	n.s.
wavelet.HHH_glcm_Contrast	0,928581	0,48455	0,59733	n.s.
log.sigma.1.0.mm.3D_glszm_LargeAreaHighGrayLevelEmphasis	0,930825	0,48487	0,59733	n.s.
wavelet.HHL_firstorder_Entropy	0,926684	0,487624	0,599693	n.s.
original_glszm_GrayLevelNonUniformity	0,924675	0,488964	0,600727	n.s.
wavelet.HLH_glrlm_ShortRunLowGrayLevelEmphasis	0,921831	0,490864	0,60217	n.s.
log.sigma.5.0.mm.3D_gldm_HighGrayLevelEmphasis	0,921421	0,491139	0,60217	n.s.
wavelet.HHL_glrlm_GrayLevelNonUniformityNormalized	0,918995	0,492764	0,602935	n.s.
wavelet.LHL_glcm_JointEntropy	0,919019	0,492747	0,602935	n.s.
wavelet.HHL_glrlm_GrayLevelVariance	0,917689	0,49364	0,603393	n.s.
wavelet.HHL_glcm_Idm	0,914379	0,495865	0,605499	n.s.
wavelet.HLL_glszm_SizeZoneNonUniformityNormalized	0,909434	0,499202	0,608339	n.s.
wavelet.LHH_firstorder_Uniformity	0,909725	0,499005	0,608339	n.s.
wavelet.HLH_gldm_DependenceVariance	0,90761	0,500435	0,609226	n.s.
wavelet.HHH_glrlm_GrayLevelNonUniformityNormalized	0,902653	0,503799	0,612701	n.s.
wavelet.HLH_glrlm_ShortRunHighGrayLevelEmphasis	0,900182	0,505481	0,613662	n.s.
wavelet.HHH_glszm_SmallAreaHighGrayLevelEmphasis	0,899995	0,505608	0,613662	n.s.
wavelet.HHL_firstorder_RobustMeanAbsoluteDeviation	0,896266	0,508153	0,613665	n.s.
wavelet.HHL_glcm_DifferenceEntropy	0,896087	0,508275	0,613665	n.s.
wavelet.LHL_glrlm_RunPercentage	0,897296	0,507449	0,613665	n.s.
wavelet.LHH_firstorder_Variance	0,896303	0,508128	0,613665	n.s.
wavelet.LHH_glszm_LowGrayLevelZoneEmphasis	0,894541	0,509333	0,613665	n.s.
wavelet.HHH_glcm_ClusterTendency	0,896048	0,508302	0,613665	n.s.
wavelet.HHH_gldm_SmallDependenceLowGrayLevelEmphasis	0,894021	0,509689	0,613665	n.s.
log.sigma.5.0.mm.3D_glszm_SizeZoneNonUniformity	0,895188	0,50889	0,613665	n.s.
wavelet.HHH_glrlm_GrayLevelVariance	0,891175	0,51164	0,615399	n.s.
log.sigma.5.0.mm.3D_gldm_LowGrayLevelEmphasis	0,88628	0,515006	0,618829	n.s.
wavelet.HHH_glcm_DifferenceEntropy	0,884986	0,515898	0,619283	n.s.
wavelet.LHH_glrlm_ShortRunLowGrayLevelEmphasis	0,879308	0,519823	0,623373	n.s.
wavelet.HHL_glcm_JointEntropy	0,874152	0,523402	0,624504	n.s.
wavelet.HHL_glcm_DifferenceAverage	0,874534	0,523136	0,624504	n.s.
wavelet.HHL_glszm_ZoneEntropy	0,872423	0,524605	0,624504	n.s.

wavelet.LLH_glszm_SmallAreaEmphasis	0,871978	0,524915	0,624504	n.s.
wavelet.LHL_glszm_SizeZoneNonUniformityNormalized	0,873244	0,524034	0,624504	n.s.
log.sigma.3.0.mm.3D_glszm_SizeZoneNonUniformity	0,875116	0,522732	0,624504	n.s.
log.sigma.5.0.mm.3D_glszm_GrayLevelNonUniformity	0,873691	0,523722	0,624504	n.s.
log.sigma.5.0.mm.3D_glszm_SizeZoneNonUniformityNormalized	0,872231	0,524739	0,624504	n.s.
log.sigma.1.0.mm.3D_glszm_SizeZoneNonUniformityNormalized	0,868697	0,527204	0,626607	n.s.
wavelet.LHL_glcm_Correlation	0,866674	0,528618	0,627669	n.s.
wavelet.HHH_glcm_DifferenceVariance	0,864522	0,530125	0,628385	n.s.
log.sigma.2.0.mm.3D_glszm_GrayLevelNonUniformity	0,864321	0,530265	0,628385	n.s.
wavelet.LLH_firstorder_Kurtosis	0,863175	0,531069	0,628718	n.s.
wavelet.HHH_firstorder_Entropy	0,861786	0,532043	0,629241	n.s.
wavelet.HHH_glszm_ZoneVariance	0,861056	0,532556	0,629241	n.s.
wavelet.HHH_glcm_ClusterProminence	0,859906	0,533364	0,629579	n.s.
wavelet.HHL_gldm_LargeDependenceEmphasis	0,858617	0,534271	0,630031	n.s.
log.sigma.5.0.mm.3D_glcm_JointAverage	0,855303	0,536606	0,632166	n.s.
wavelet.HLH_glcm_InverseVariance	0,852057	0,538898	0,634246	n.s.
wavelet.HHH_glcm_SumSquares	0,849535	0,540682	0,635724	n.s.
wavelet.HHH_glcm_JointEntropy	0,848495	0,541419	0,63597	n.s.
wavelet.HHL_glcm_SumEntropy	0,84364	0,544867	0,638512	n.s.
wavelet.HHL_glcm_Imc1	0,844453	0,544289	0,638512	n.s.
wavelet.HHL_gldm_SmallDependenceHighGrayLevelEmphasis	0,843207	0,545175	0,638512	n.s.
wavelet.HHH_glcm_SumEntropy	0,842275	0,545838	0,638668	n.s.
wavelet.LHH_glrlm_ShortRunHighGrayLevelEmphasis	0,840647	0,546998	0,639403	n.s.
log.sigma.5.0.mm.3D_glszm_GrayLevelNonUniformityNormalized	0,839076	0,548118	0,640091	n.s.
wavelet.LHH_glrlm_RunVariance	0,834481	0,551402	0,643302	n.s.
wavelet.HHL_glcm_DifferenceVariance	0,830396	0,55433	0,644221	n.s.
wavelet.HHL_gldm_GrayLevelVariance	0,831097	0,553827	0,644221	n.s.
wavelet.HHL_gldm_DependenceEntropy	0,831412	0,553601	0,644221	n.s.
wavelet.LHH_gldm_HighGrayLevelEmphasis	0,830819	0,554027	0,644221	n.s.
wavelet.HHL_glcm_JointEnergy	0,826617	0,557046	0,646753	n.s.
wavelet.LHL_glrlm_ShortRunEmphasis	0,825533	0,557826	0,647035	n.s.
wavelet.LHH_glcm_Autocorrelation	0,823467	0,559315	0,648138	n.s.
wavelet.LLH_glcm_ClusterShade	0,820975	0,561113	0,648249	n.s.
wavelet.LHL_firstorder_Kurtosis	0,821596	0,560664	0,648249	n.s.
wavelet.HLH_glrlm_HighGrayLevelRunEmphasis	0,820349	0,561565	0,648249	n.s.
wavelet.HHH_glcm_Imc2	0,822161	0,560256	0,648249	n.s.
log.sigma.1.0.mm.3D_glszm_GrayLevelNonUniformity	0,818173	0,563138	0,649443	n.s.
wavelet.HHL_firstorder_Uniformity	0,81652	0,564335	0,649578	n.s.
wavelet.HHH_gldm_GrayLevelVariance	0,817263	0,563796	0,649578	n.s.
wavelet.HHL_glcm_Contrast	0,813896	0,566236	0,650524	n.s.
wavelet.LHH_gldm_LowGrayLevelEmphasis	0,81425	0,565979	0,650524	n.s.
wavelet.LHH_glrlm_HighGrayLevelRunEmphasis	0,811599	0,567904	0,651817	n.s.
wavelet.HLH_glcm_Autocorrelation	0,809187	0,569657	0,653207	n.s.
wavelet.LHH_glrlm_LowGrayLevelRunEmphasis	0,808196	0,570378	0,653411	n.s.
wavelet.HLH_gldm_HighGrayLevelEmphasis	0,80617	0,571854	0,653858	n.s.
wavelet.LLL_glcm_ClusterShade	0,806859	0,571352	0,653858	n.s.

wavelet.HHL_glrlm_RunEntropy	0,801562	0,575218	0,65708	n.s.
wavelet.HHL_firstorder_Minimum	0,799691	0,576586	0,658018	n.s.
wavelet.LHH_glcm_JointAverage	0,798795	0,577242	0,658143	n.s.
wavelet.LHH_gldm_LargeDependenceLowGrayLevelEmphasis	0,797354	0,578296	0,658722	n.s.
wavelet.LHL_glcm_ClusterTendency	0,794996	0,580025	0,659591	n.s.
wavelet.HHH_glszm_GrayLevelVariance	0,79482	0,580155	0,659591	n.s.
wavelet.LHL_glrlm_RunLengthNonUniformityNormalized	0,792391	0,581938	0,660994	n.s.
wavelet.LHH_glrlm_LongRunHighGrayLevelEmphasis	0,789864	0,583796	0,661739	n.s.
log.sigma.1.0.mm.3D_firstorder_90Percentile	0,790433	0,583378	0,661739	n.s.
log.sigma.3.0.mm.3D_glszm_GrayLevelVariance	0,789257	0,584243	0,661739	n.s.
wavelet.HHL_glcm_Imc2	0,786528	0,586253	0,662033	n.s.
wavelet.LHL_gldm_GrayLevelVariance	0,787069	0,585855	0,662033	n.s.
wavelet.LHL_gldm_SmallDependenceEmphasis	0,787432	0,585587	0,662033	n.s.
log.sigma.5.0.mm.3D_glrlm_ShortRunLowGrayLevelEmphasis	0,78592	0,586702	0,662033	n.s.
wavelet.LHL_glszm_ZoneEntropy	0,784726	0,587582	0,662405	n.s.
wavelet.HHH_firstorder_Uniformity	0,782902	0,588929	0,663302	n.s.
wavelet.HHL_glcm_SumSquares	0,780562	0,590659	0,663962	n.s.
wavelet.LLH_glcm_Correlation	0,779872	0,591169	0,663962	n.s.
wavelet.HLH_glszm_LowGrayLevelZoneEmphasis	0,779928	0,591128	0,663962	n.s.
wavelet.LHH_firstorder_Maximum	0,779007	0,591809	0,664061	n.s.
wavelet.LHL_glrlm_GrayLevelVariance	0,776626	0,593572	0,664377	n.s.
wavelet.LHL_glcm_JointEnergy	0,777674	0,592796	0,664377	n.s.
wavelet.HHH_glcm_Imc1	0,7762	0,593888	0,664377	n.s.
log.sigma.3.0.mm.3D_glszm_SmallAreaHighGrayLevelEmphasis	0,775648	0,594298	0,664377	n.s.
wavelet.LHL_glcm_SumSquares	0,770574	0,598065	0,666732	n.s.
log.sigma.3.0.mm.3D_glcm_Correlation	0,770931	0,597799	0,666732	n.s.
log.sigma.5.0.mm.3D_firstorder_Kurtosis	0,771081	0,597688	0,666732	n.s.
log.sigma.5.0.mm.3D_gldm_LargeDependenceHighGrayLevelEmphasis	0,769554	0,598823	0,66696	n.s.
wavelet.HHL_firstorder_10Percentile	0,76314	0,603602	0,671661	n.s.
wavelet.LLH_firstorder_Maximum	0,758159	0,607323	0,675177	n.s.
wavelet.HHL_glcm_ClusterTendency	0,756733	0,60839	0,675739	n.s.
wavelet.LHH_firstorder_10Percentile	0,755114	0,609602	0,676462	n.s.
wavelet.HHL_gldm_LargeDependenceLowGrayLevelEmphasis	0,750306	0,613207	0,67921	n.s.
wavelet.HLH_firstorder_Median	0,750739	0,612882	0,67921	n.s.
wavelet.HLH_glszm_SizeZoneNonUniformity	0,742307	0,619222	0,685242	n.s.
wavelet.HHL_glcm_ClusterProminence	0,739826	0,621092	0,68668	n.s.
wavelet.HHL_firstorder_90Percentile	0,736883	0,623312	0,687389	n.s.
wavelet.LLH_glszm_SizeZoneNonUniformityNormalized	0,737188	0,623082	0,687389	n.s.
wavelet.LHL_firstorder_10Percentile	0,736706	0,623446	0,687389	n.s.
wavelet.LHL_glcm_DifferenceVariance	0,734506	0,625108	0,687961	n.s.
wavelet.LHH_glrlm_LongRunLowGrayLevelEmphasis	0,735083	0,624672	0,687961	n.s.
wavelet.LHL_firstorder_Variance	0,726346	0,631283	0,694123	n.s.
wavelet.LHL_glcm_ClusterProminence	0,721532	0,634936	0,696232	n.s.
wavelet.LHH_firstorder_Kurtosis	0,722133	0,634479	0,696232	n.s.
wavelet.LLL_glszm_GrayLevelNonUniformity	0,72184	0,634702	0,696232	n.s.
wavelet.HLH_glszm_SmallAreaEmphasis	0,717355	0,638109	0,699075	n.s.

wavelet.LLH_glcm_Idmn	0,714093	0,640592	0,70052	n.s.
wavelet.LHH_glrlm_RunEntropy	0,714782	0,640067	0,70052	n.s.
wavelet.LHH_glcm_Idn	0,707127	0,6459	0,705684	n.s.
wavelet.LLH_glszm_LargeAreaHighGrayLevelEmphasis	0,698642	0,652381	0,712119	n.s.
log.sigma.5.0.mm.3D_glcm_Autocorrelation	0,697366	0,653358	0,712539	n.s.
original_glszm_SmallAreaEmphasis	0,696142	0,654294	0,712914	n.s.
wavelet.LHL_firstorder_Range	0,695219	0,655001	0,713039	n.s.
wavelet.HLH_glrlm_LongRunHighGrayLevelEmphasis	0,688356	0,660258	0,718113	n.s.
wavelet.HHH_glcm_JointEnergy	0,685868	0,662167	0,719539	n.s.
wavelet.LHL_glcm_Contrast	0,681904	0,66521	0,722193	n.s.
wavelet.HLH_gldm_LargeDependenceHighGrayLevelEmphasis	0,674986	0,670525	0,727309	n.s.
original_gldm_DependenceVariance	0,670278	0,674147	0,730579	n.s.
wavelet.LHH_gldm_DependenceNonUniformityNormalized	0,668208	0,67574	0,730989	n.s.
wavelet.HHH_glrlm_RunLengthNonUniformityNormalized	0,668829	0,675262	0,730989	n.s.
wavelet.LLH_firstorder_Skewness	0,6613	0,681061	0,734764	n.s.
wavelet.LHL_glcm_Id	0,661875	0,680618	0,734764	n.s.
log.sigma.2.0.mm.3D_glcm_Idmn	0,661991	0,680528	0,734764	n.s.
wavelet.LHL_glcm_DifferenceAverage	0,660449	0,681716	0,734813	n.s.
log.sigma.2.0.mm.3D_gldm_DependenceNonUniformity	0,649585	0,690094	0,743178	n.s.
wavelet.LHH_gldm_DependenceVariance	0,645838	0,692985	0,745625	n.s.
log.sigma.5.0.mm.3D_gldm_SmallDependenceLowGrayLevelEmphasis	0,644816	0,693774	0,745807	n.s.
wavelet.LHH_glcm_Idmn	0,640155	0,697371	0,749005	n.s.
wavelet.LHL_glcm_DifferenceEntropy	0,63874	0,698464	0,749479	n.s.
wavelet.HHH_firstorder_MeanAbsoluteDeviation	0,637971	0,699057	0,749479	n.s.
log.sigma.1.0.mm.3D_gldm_DependenceVariance	0,633019	0,70288	0,752907	n.s.
wavelet.HHH_firstorder_90Percentile	0,627325	0,707276	0,756943	n.s.
log.sigma.3.0.mm.3D_glcm_Idmn	0,620592	0,712473	0,761828	n.s.
log.sigma.2.0.mm.3D_glcm_Imc1	0,615326	0,716538	0,765494	n.s.
wavelet.HHH_glszm_HighGrayLevelZoneEmphasis	0,613467	0,717972	0,766345	n.s.
wavelet.LHL_glcm_Idm	0,610747	0,72007	0,767905	n.s.
wavelet.LHH_glrlm_GrayLevelNonUniformity	0,608365	0,721907	0,769183	n.s.
wavelet.LLL_glszm_SmallAreaLowGrayLevelEmphasis	0,596457	0,731082	0,778269	n.s.
wavelet.HHL_firstorder_InterquartileRange	0,593948	0,733013	0,778947	n.s.
wavelet.HLH_firstorder_Minimum	0,594021	0,732956	0,778947	n.s.
wavelet.LHH_firstorder_MeanAbsoluteDeviation	0,59096	0,735311	0,780701	n.s.
wavelet.HLH_gldm_DependenceNonUniformityNormalized	0,589677	0,736298	0,78106	n.s.
wavelet.LHH_gldm_DependenceNonUniformity	0,583726	0,740871	0,785218	n.s.
log.sigma.3.0.mm.3D_glszm_GrayLevelNonUniformity	0,581807	0,742344	0,786088	n.s.
wavelet.HLH_glszm_HighGrayLevelZoneEmphasis	0,5707	0,750855	0,794402	n.s.
wavelet.HHL_glrlm_ShortRunHighGrayLevelEmphasis	0,566728	0,753892	0,796915	n.s.
wavelet.HHL_firstorder_Energy	0,568385	0,755862	0,797596	n.s.
wavelet.HHL_firstorder_TotalEnergy	0,568385	0,755862	0,797596	n.s.
original_glszm_SizeZoneNonUniformityNormalized	0,550348	0,766371	0,807978	n.s.
wavelet.LHH_glcm_Correlation	0,54802	0,768139	0,809133	n.s.
wavelet.HHL_gldm_HighGrayLevelEmphasis	0,546777	0,769081	0,809418	n.s.
wavelet.HHL_firstorder_Mean	0,537737	0,775921	0,815903	n.s.

wavelet.HLH_glszm_SmallAreaHighGrayLevelEmphasis	0,536691	0,776711	0,816021	n.s.
wavelet.HHL_glrlm_HighGrayLevelRunEmphasis	0,533156	0,779375	0,817394	n.s.
wavelet.HHL_glcm_Autocorrelation	0,533953	0,778775	0,817394	n.s.
wavelet.LHH_glszm_ZoneEntropy	0,531264	0,780799	0,818174	n.s.
wavelet.HHL_glcm_JointAverage	0,528363	0,78298	0,819746	n.s.
log.sigma.2.0.mm.3D_glszm_SmallAreaLowGrayLevelEmphasis	0,527828	0,783933	0,82003	n.s.
wavelet.HHH_glrlm_ShortRunEmphasis	0,525268	0,785303	0,82075	n.s.
wavelet.HHL_firstorder_RootMeanSquared	0,51849	0,790375	0,82462	n.s.
wavelet.HHL_gldm_DependenceNonUniformityNormalized	0,518665	0,790245	0,82462	n.s.
wavelet.LHL_glcm_Imc1	0,516148	0,792122	0,825727	n.s.
wavelet.HLH_glcm_Idmn	0,510939	0,795999	0,829051	n.s.
log.sigma.4.0.mm.3D_glszm_SmallAreaHighGrayLevelEmphasis	0,504287	0,80093	0,833466	n.s.
wavelet.HHH_glszm_LowGrayLevelZoneEmphasis	0,4962	0,806891	0,838944	n.s.
wavelet.HHL_glcm_MaximumProbability	0,493656	0,808759	0,840161	n.s.
wavelet.HHL_glszm_SmallAreaEmphasis	0,482198	0,817121	0,848115	n.s.
wavelet.LHL_firstorder_Maximum	0,475078	0,822275	0,852729	n.s.
wavelet.HHL_glrlm_LongRunLowGrayLevelEmphasis	0,472548	0,824098	0,85315	n.s.
wavelet.HHH_glrlm_GrayLevelNonUniformity	0,473492	0,823418	0,85315	n.s.
wavelet.LHH_firstorder_RobustMeanAbsoluteDeviation	0,468671	0,826882	0,855297	n.s.
wavelet.HHL_glrlm_LongRunHighGrayLevelEmphasis	0,465648	0,829045	0,856063	n.s.
wavelet.HLH_glcm_Idn	0,466494	0,82844	0,856063	n.s.
wavelet.HHL_gldm_LargeDependenceHighGrayLevelEmphasis	0,464581	0,829807	0,856116	n.s.
wavelet.HHH_firstorder_InterquartileRange	0,46069	0,832578	0,857051	n.s.
log.sigma.3.0.mm.3D_glcm_Imc1	0,46031	0,832848	0,857051	n.s.
log.sigma.5.0.mm.3D_firstorder_Maximum	0,462129	0,831555	0,857051	n.s.
wavelet.LHL_glcm_InverseVariance	0,454778	0,836766	0,860347	n.s.
wavelet.HHL_firstorder_Kurtosis	0,449792	0,840986	0,863823	n.s.
wavelet.LLH_glcm_Idn	0,447931	0,841582	0,863823	n.s.
log.sigma.4.0.mm.3D_firstorder_Skewness	0,432414	0,852344	0,874125	n.s.
wavelet.LHH_gldm_DependenceEntropy	0,429182	0,854558	0,875649	n.s.
log.sigma.4.0.mm.3D_glrlm_LongRunHighGrayLevelEmphasis	0,425076	0,857357	0,87777	n.s.
wavelet.HLH_firstorder_Energy	0,423984	0,863522	0,882581	n.s.
wavelet.HLH_firstorder_TotalEnergy	0,423984	0,863522	0,882581	n.s.
wavelet.LHL_glszm_ZonePercentage	0,400726	0,873601	0,892126	n.s.
wavelet.LLL_gldm_SmallDependenceLowGrayLevelEmphasis	0,394205	0,877842	0,895697	n.s.
wavelet.HHH_glszm_GrayLevelNonUniformityNormalized	0,379385	0,887296	0,904576	n.s.
wavelet.LHH_firstorder_InterquartileRange	0,361733	0,898196	0,914914	n.s.
wavelet.LHL_glcm_Imc2	0,344644	0,90835	0,924474	n.s.
wavelet.HHL_glszm_SmallAreaLowGrayLevelEmphasis	0,333417	0,914791	0,930244	n.s.
wavelet.HHL_firstorder_Median	0,31831	0,923156	0,937817	n.s.
wavelet.HHL_glszm_SizeZoneNonUniformityNormalized	0,317125	0,923797	0,937817	n.s.
wavelet.HLH_firstorder_RootMeanSquared	0,310642	0,927263	0,940543	n.s.
wavelet.HHL_glszm_SmallAreaHighGrayLevelEmphasis	0,294369	0,935655	0,948256	n.s.
wavelet.LHH_gldm_GrayLevelNonUniformity	0,289035	0,938306	0,950144	n.s.
log.sigma.4.0.mm.3D_glszm_SmallAreaEmphasis	0,28147	0,941925	0,953006	n.s.
wavelet.HLH_firstorder_Mean	0,247746	0,957079	0,967525	n.s.

wavelet.LHH_glszm_LargeAreaHighGrayLevelEmphasis	0,235552	0,961999	0,971683	n.s.
wavelet.HHL_glszm_LowGrayLevelZoneEmphasis	0,225054	0,965995	0,972931	n.s.
wavelet.HHL_gldm_LowGrayLevelEmphasis	0,229098	0,964482	0,972931	n.s.
wavelet.HHH_firstorder_RobustMeanAbsoluteDeviation	0,223777	0,966466	0,972931	n.s.
log.sigma.4.0.mm.3D_glcm_Correlation	0,224537	0,966187	0,972931	n.s.
wavelet.HHL_glszm_HighGrayLevelZoneEmphasis	0,221352	0,967352	0,973009	n.s.
wavelet.LHH_glszm_SmallAreaLowGrayLevelEmphasis	0,214723	0,96971	0,974566	n.s.
wavelet.HHL_gldm_SmallDependenceLowGrayLevelEmphasis	0,210967	0,971006	0,975055	n.s.
wavelet.LHH_glrlm_RunLengthNonUniformity	0,195775	0,975945	0,979199	n.s.
wavelet.HHL_glrlm_LowGrayLevelRunEmphasis	0,184493	0,979299	0,981745	n.s.
wavelet.HHL_glrlm_ShortRunLowGrayLevelEmphasis	0,118206	0,993572	0,995226	n.s.
wavelet.LLH_glszm_GrayLevelNonUniformity	0,099807	0,995946	0,996774	n.s.
wavelet.HHL_glcm_InverseVariance	0,051087	0,999396	0,999396	n.s.
