# Supplementary material for: Contrast Agent Dynamics Determine Radiomics Profiles in Oncologic Imaging
Source: Cancers (Basel). 2024 Apr 16;16(8):1519. doi: 10.3390/cancers16081519 (PMC11049400; doi:10.3390/cancers16081519)
Supplement: Supplementary file 1 [file cancers-16-01519-s001.zip › Table S3.pdf]

**Table S3: Linear mixed model p values and cluster membership of all CT radiomics features for lung tumors**

| Feature | F value | p value | FDR | Cluster |
|---|---|---|---|---|
| original_firstorder_Median | 10,96778 | 6,28E-07 | 0,000422 | 2 |
| wavelet.LLL_firstorder_Median | 10,85605 | 7,03E-07 | 0,000422 | 2 |
| original_firstorder_RootMeanSquared | 9,660241 | 2,44E-06 | 0,000586 | 2 |
| original_firstorder_Mean | 9,74699 | 2,23E-06 | 0,000586 | 2 |
| wavelet.LLL_firstorder_Mean | 9,707863 | 2,32E-06 | 0,000586 | 2 |
| wavelet.LHH_gldm_DependenceEntropy | 9,131827 | 4,35E-06 | 0,000746 | 3 |
| wavelet.LLL_firstorder_RootMeanSquared | 9,165788 | 4,19E-06 | 0,000746 | 2 |
| wavelet.LLL_firstorder_90Percentile | 8,430749 | 9,64E-06 | 0,001445 | 2 |
| original_firstorder_90Percentile | 7,764442 | 2,12E-05 | 0,002541 | 2 |
| wavelet.LHL_glrlm_RunVariance | 7,782958 | 2,07E-05 | 0,002541 | 1 |
| wavelet.LHL_glrlm_LongRunEmphasis | 7,655608 | 2,42E-05 | 0,002635 | 1 |
| wavelet.LHH_glrlm_GrayLevelNonUniformityNormalized | 7,518407 | 2,85E-05 | 0,002855 | 1 |
| wavelet.HHL_glcm_SumEntropy | 7,375692 | 3,4E-05 | 0,003141 | 3 |
| wavelet.HHL_glrlm_GrayLevelNonUniformityNormalized | 7,280567 | 3,83E-05 | 0,003282 | 1 |
| wavelet.HHL_glcm_JointEntropy | 7,121436 | 4,67E-05 | 0,003736 | 3 |
| wavelet.HHL_glcm_JointEnergy | 7,054701 | 5,08E-05 | 0,00381 | 1 |
| wavelet.HHL_glcm_DifferenceEntropy | 6,911275 | 6,09E-05 | 0,004061 | 3 |
| wavelet.HHL_glrlm_ShortRunEmphasis | 6,952587 | 5,78E-05 | 0,004061 | 3 |
| wavelet.LHH_firstorder_Uniformity | 6,807018 | 6,96E-05 | 0,004176 | 1 |
| wavelet.LHL_gldm_LargeDependenceEmphasis | 6,835463 | 6,71E-05 | 0,004176 | 1 |
| original_firstorder_10Percentile | 6,615392 | 8,91E-05 | 0,005092 | 2 |
| wavelet.HHL_firstorder_Entropy | 6,567156 | 9,49E-05 | 0,005174 | 3 |
| wavelet.LHH_glcm_JointEnergy | 6,440487 | 0,000112 | 0,005373 | 1 |
| wavelet.HHL_firstorder_Uniformity | 6,445173 | 0,000111 | 0,005373 | 1 |
| wavelet.HHL_glrlm_LongRunEmphasis | 6,446432 | 0,000111 | 0,005373 | 1 |
| wavelet.LHH_firstorder_Entropy | 6,283994 | 0,000138 | 0,005834 | 3 |
| wavelet.LHH_glrlm_ShortRunEmphasis | 6,159534 | 0,000162 | 0,005834 | 3 |
| wavelet.LHH_glrlm_LongRunEmphasis | 6,12467 | 0,00017 | 0,005834 | 1 |
| wavelet.HHL_gldm_LargeDependenceEmphasis | 6,133071 | 0,000168 | 0,005834 | 1 |
| wavelet.HHL_glcm_Idm | 6,139463 | 0,000167 | 0,005834 | 1 |
| wavelet.HHL_glcm_Id | 6,147261 | 0,000165 | 0,005834 | 1 |
| wavelet.HHL_glrlm_RunLengthNonUniformityNormalized | 6,18749 | 0,000156 | 0,005834 | 3 |
| wavelet.HHL_glrlm_RunPercentage | 6,314809 | 0,000132 | 0,005834 | 3 |
| wavelet.LHL_glrlm_RunPercentage | 6,150911 | 0,000164 | 0,005834 | 3 |
| wavelet.LHL_glszm_GrayLevelVariance | 6,193962 | 0,000155 | 0,005834 | 3 |
| wavelet.LHL_glrlm_ShortRunEmphasis | 6,083553 | 0,00018 | 0,005994 | 3 |
| wavelet.LHH_glcm_DifferenceEntropy | 5,948524 | 0,000216 | 0,006858 | 3 |
| wavelet.HHL_firstorder_10Percentile | 5,943619 | 0,000217 | 0,006858 | 1 |
| wavelet.LHH_glcm_SumEntropy | 5,887664 | 0,000234 | 0,00721 | 3 |
| wavelet.LHH_glcm_JointEntropy | 5,803164 | 0,000263 | 0,007697 | 3 |
| log.sigma.5.0.mm.3D_firstorder_Minimum | 5,815146 | 0,000259 | 0,007697 | 3 |

| | | | | |
|---|---|---|---|---|
| wavelet.HHL_glrlm_RunVariance | 5,780023 | 0,000271 | 0,007755 | 1 |
| wavelet.LHH_glrlm_RunEntropy | 5,753042 | 0,000282 | 0,00786 | 3 |
| wavelet.LLL_firstorder_10Percentile | 5,719324 | 0,000295 | 0,008046 | 2 |
| wavelet.LLH_glrlm_RunLengthNonUniformityNormalized | 5,696714 | 0,000304 | 0,008116 | 3 |
| wavelet.HHL_glcm_DifferenceAverage | 5,660633 | 0,00032 | 0,008168 | 3 |
| wavelet.LHL_glcm_DifferenceEntropy | 5,66893 | 0,000316 | 0,008168 | 3 |
| wavelet.LLH_glrlm_ShortRunEmphasis | 5,591275 | 0,000352 | 0,008803 | 3 |
| wavelet.HLL_firstorder_90Percentile | 5,499095 | 0,0004 | 0,009723 | 3 |
| wavelet.LHL_glrlm_RunLengthNonUniformityNormalized | 5,490587 | 0,000405 | 0,009723 | 3 |
| wavelet.LLH_glcm_Id | 5,411843 | 0,000452 | 0,010242 | 1 |
| wavelet.LLH_glrlm_RunPercentage | 5,412776 | 0,000452 | 0,010242 | 3 |
| wavelet.LHL_gldm_DependenceVariance | 5,425975 | 0,000443 | 0,010242 | 1 |
| wavelet.LHL_firstorder_Entropy | 5,385974 | 0,000469 | 0,010425 | 3 |
| wavelet.LLH_glcm_Idm | 5,364684 | 0,000483 | 0,010547 | 1 |
| wavelet.LLH_glcm_DifferenceEntropy | 5,347068 | 0,000496 | 0,010619 | 3 |
| wavelet.LHL_glcm_JointEntropy | 5,285688 | 0,00054 | 0,011378 | 3 |
| wavelet.HHL_glrlm_GrayLevelVariance | 5,200005 | 0,00061 | 0,012629 | 3 |
| wavelet.LLH_glcm_DifferenceAverage | 5,185085 | 0,000624 | 0,012681 | 3 |
| wavelet.LHH_glrlm_RunLengthNonUniformityNormalized | 5,163683 | 0,000643 | 0,012857 | 3 |
| wavelet.HHL_glszm_ZoneEntropy | 5,125777 | 0,000679 | 0,013135 | 3 |
| wavelet.LHL_glcm_DifferenceVariance | 5,128061 | 0,000676 | 0,013135 | 3 |
| wavelet.LHL_glcm_JointEnergy | 5,107074 | 0,000697 | 0,013277 | 1 |
| wavelet.LHH_glrlm_RunPercentage | 5,054076 | 0,000752 | 0,014103 | 3 |
| wavelet.HHL_glcm_DifferenceVariance | 5,020056 | 0,00079 | 0,014583 | 3 |
| wavelet.HHL_gldm_DependenceEntropy | 4,992522 | 0,000822 | 0,014721 | 3 |
| wavelet.HHL_glcm_ClusterTendency | 4,999162 | 0,000814 | 0,014721 | 3 |
| wavelet.LHH_firstorder_InterquartileRange | 4,976198 | 0,000842 | 0,01485 | 3 |
| wavelet.LHH_glrlm_RunVariance | 4,921798 | 0,00091 | 0,015835 | 1 |
| wavelet.HHL_glcm_SumSquares | 4,902678 | 0,000936 | 0,016048 | 3 |
| wavelet.LHH_firstorder_MeanAbsoluteDeviation | 4,870337 | 0,000981 | 0,016281 | 3 |
| wavelet.LHH_gldm_LargeDependenceEmphasis | 4,856712 | 0,001001 | 0,016281 | 1 |
| wavelet.HHL_glrlm_RunEntropy | 4,85455 | 0,001004 | 0,016281 | 3 |
| wavelet.LHL_glrlm_GrayLevelVariance | 4,875862 | 0,000973 | 0,016281 | 3 |
| wavelet.LHH_glcm_MaximumProbability | 4,837244 | 0,00103 | 0,016417 | 1 |
| wavelet.HHL_gldm_GrayLevelVariance | 4,82557 | 0,001047 | 0,016417 | 3 |
| wavelet.HHL_glcm_Contrast | 4,783629 | 0,001114 | 0,016417 | 3 |
| wavelet.LLH_glcm_InverseVariance | 4,762128 | 0,001149 | 0,016417 | 1 |
| wavelet.LHL_firstorder_Uniformity | 4,762847 | 0,001148 | 0,016417 | 1 |
| wavelet.LHL_glcm_Idm | 4,764596 | 0,001145 | 0,016417 | 1 |
| wavelet.LHL_glcm_Id | 4,78182 | 0,001117 | 0,016417 | 1 |
| wavelet.LHL_glcm_SumEntropy | 4,790798 | 0,001102 | 0,016417 | 3 |
| wavelet.LHL_glrlm_GrayLevelNonUniformityNormalized | 4,791027 | 0,001102 | 0,016417 | 1 |
| wavelet.LHL_glrlm_RunEntropy | 4,813122 | 0,001067 | 0,016417 | 3 |
| wavelet.HHL_firstorder_90Percentile | 4,702412 | 0,001255 | 0,017711 | 3 |
| wavelet.LHH_glcm_DifferenceVariance | 4,670072 | 0,001316 | 0,018359 | 3 |
| wavelet.LHH_firstorder_90Percentile | 4,658088 | 0,001339 | 0,018472 | 3 |

| | | | | |
|---|---|---|---|---|
| wavelet.HHL_firstorder_MeanAbsoluteDeviation | 4,601076 | 0,001457 | 0,019343 | 3 |
| wavelet.LLH_gldm_LargeDependenceEmphasis | 4,610737 | 0,001436 | 0,019343 | 1 |
| wavelet.LHL_gldm_GrayLevelVariance | 4,615681 | 0,001426 | 0,019343 | 3 |
| wavelet.LHL_glszm_GrayLevelNonUniformityNormalized | 4,596525 | 0,001467 | 0,019343 | 1 |
| wavelet.LHH_glcm_Idm | 4,558271 | 0,001553 | 0,019819 | 1 |
| wavelet.LLH_glcm_Contrast | 4,565323 | 0,001536 | 0,019819 | 3 |
| wavelet.LHL_glcm_ClusterProminence | 4,572745 | 0,00152 | 0,019819 | 3 |
| wavelet.LHH_glrlm_GrayLevelVariance | 4,544553 | 0,001585 | 0,020015 | 3 |
| wavelet.LHL_firstorder_Variance | 4,515378 | 0,001655 | 0,020685 | 3 |
| wavelet.LHH_glcm_Id | 4,503769 | 0,001684 | 0,020829 | 1 |
| wavelet.LLH_glrlm_LongRunEmphasis | 4,39322 | 0,001986 | 0,024324 | 1 |
| wavelet.LHH_firstorder_10Percentile | 4,348194 | 0,002126 | 0,025765 | 1 |
| wavelet.LHL_glcm_SumSquares | 4,339701 | 0,002153 | 0,025836 | 3 |
| wavelet.LHL_glcm_ClusterTendency | 4,31311 | 0,002241 | 0,026626 | 3 |
| wavelet.LHH_firstorder_RobustMeanAbsoluteDeviation | 4,299907 | 0,002286 | 0,026896 | 3 |
| wavelet.LHH_firstorder_Variance | 4,234527 | 0,002524 | 0,028911 | 3 |
| wavelet.LHH_glcm_DifferenceAverage | 4,220594 | 0,002578 | 0,028911 | 3 |
| wavelet.HHL_firstorder_Variance | 4,220714 | 0,002577 | 0,028911 | 3 |
| wavelet.LHL_glcm_DifferenceAverage | 4,245093 | 0,002484 | 0,028911 | 3 |
| log.sigma.3.0.mm.3D_firstorder_Maximum | 4,229176 | 0,002545 | 0,028911 | 3 |
| wavelet.LHH_gldm_GrayLevelVariance | 4,178983 | 0,002746 | 0,03029 | 3 |
| wavelet.LLH_glcm_DifferenceVariance | 4,177753 | 0,002751 | 0,03029 | 3 |
| wavelet.HHL_glcm_MaximumProbability | 4,159368 | 0,002829 | 0,030687 | 1 |
| wavelet.LLH_gldm_SmallDependenceEmphasis | 4,157265 | 0,002839 | 0,030687 | 3 |
| wavelet.LHL_firstorder_MeanAbsoluteDeviation | 4,099912 | 0,003098 | 0,033196 | 3 |
| wavelet.HLH_glszm_GrayLevelVariance | 4,090009 | 0,003146 | 0,033404 | 3 |
| wavelet.LLH_glszm_ZonePercentage | 4,077575 | 0,003206 | 0,033747 | 3 |
| wavelet.LHL_glcm_Contrast | 4,070714 | 0,00324 | 0,033807 | 3 |
| wavelet.HHL_firstorder_InterquartileRange | 4,043376 | 0,003379 | 0,034951 | 3 |
| wavelet.LLH_firstorder_InterquartileRange | 3,984282 | 0,0037 | 0,037866 | 3 |
| wavelet.LLH_glrlm_RunVariance | 3,980165 | 0,003723 | 0,037866 | 1 |
| wavelet.LHL_glcm_MaximumProbability | 3,969784 | 0,003784 | 0,038153 | 1 |
| wavelet.LHL_gldm_SmallDependenceEmphasis | 3,938696 | 0,003969 | 0,039694 | 3 |
| wavelet.LHH_glcm_ClusterTendency | 3,92338 | 0,004065 | 0,039979 | 3 |
| wavelet.HHL_firstorder_RobustMeanAbsoluteDeviation | 3,926686 | 0,004044 | 0,039979 | 3 |
| wavelet.LHH_glcm_SumSquares | 3,885983 | 0,004307 | 0,042017 | 3 |
| wavelet.LHH_glszm_ZoneEntropy | 3,844916 | 0,00459 | 0,04442 | 3 |
| wavelet.LHH_glcm_Contrast | 3,824213 | 0,00474 | 0,044789 | 3 |
| log.sigma.1.0.mm.3D_glcm_DifferenceVariance | 3,827201 | 0,004718 | 0,044789 | 3 |
| log.sigma.3.0.mm.3D_firstorder_Range | 3,832486 | 0,00468 | 0,044789 | 3 |
| wavelet.HLH_glszm_GrayLevelNonUniformityNormalized | 3,773798 | 0,005127 | 0,048069 | 1 |
| original_gldm_LargeDependenceEmphasis | 3,71031 | 0,005662 | 0,051087 | n.s. |
| original_glcm_Idmn | 3,71134 | 0,005653 | 0,051087 | n.s. |
| original_glrlm_RunVariance | 3,712173 | 0,005646 | 0,051087 | n.s. |
| wavelet.LHL_glcm_InverseVariance | 3,714713 | 0,005623 | 0,051087 | n.s. |
| log.sigma.1.0.mm.3D_glcm_Contrast | 3,719963 | 0,005577 | 0,051087 | n.s. |

| | | | | |
|---|---|---|---|---|
| wavelet.LLH_glcm_JointEntropy | 3,705451 | 0,005705 | 0,051093 | n.s. |
| wavelet.LHL_gldm_DependenceNonUniformityNormalized | 3,6443 | 0,00628 | 0,055821 | n.s. |
| wavelet.LLH_glszm_GrayLevelNonUniformityNormalized | 3,623565 | 0,006488 | 0,057247 | n.s. |
| log.sigma.4.0.mm.3D_firstorder_Maximum | 3,617142 | 0,006554 | 0,057407 | n.s. |
| wavelet.HHL_glcm_Correlation | 3,595978 | 0,006776 | 0,058922 | n.s. |
| wavelet.LHH_glcm_InverseVariance | 3,585727 | 0,006886 | 0,059451 | n.s. |
| wavelet.HHL_glszm_HighGrayLevelZoneEmphasis | 3,579414 | 0,006955 | 0,059617 | n.s. |
| original_glrlm_LongRunEmphasis | 3,562916 | 0,007139 | 0,060756 | n.s. |
| wavelet.LLH_glcm_JointEnergy | 3,553048 | 0,007251 | 0,061276 | n.s. |
| wavelet.LHL_glcm_Imc2 | 3,537499 | 0,007431 | 0,062361 | n.s. |
| log.sigma.5.0.mm.3D_firstorder_90Percentile | 3,518109 | 0,007663 | 0,063857 | n.s. |
| wavelet.HHL_firstorder_Minimum | 3,503984 | 0,007836 | 0,064851 | n.s. |
| original_glrlm_RunPercentage | 3,487732 | 0,008041 | 0,065637 | n.s. |
| log.sigma.5.0.mm.3D_firstorder_RootMeanSquared | 3,489996 | 0,008012 | 0,065637 | n.s. |
| log.sigma.5.0.mm.3D_firstorder_Mean | 3,47248 | 0,008237 | 0,06679 | n.s. |
| wavelet.LHL_firstorder_10Percentile | 3,463213 | 0,008359 | 0,067325 | n.s. |
| wavelet.HHL_glszm_SmallAreaHighGrayLevelEmphasis | 3,445058 | 0,008604 | 0,068832 | n.s. |
| wavelet.LHH_glszm_GrayLevelNonUniformityNormalized | 3,405703 | 0,00916 | 0,072794 | n.s. |
| wavelet.LLH_firstorder_Uniformity | 3,390547 | 0,009384 | 0,074027 | n.s. |
| wavelet.LLH_firstorder_RobustMeanAbsoluteDeviation | 3,386899 | 0,009438 | 0,074027 | n.s. |
| wavelet.LLH_glrlm_GrayLevelNonUniformityNormalized | 3,381013 | 0,009527 | 0,07424 | n.s. |
| wavelet.HHL_glszm_GrayLevelNonUniformityNormalized | 3,359376 | 0,009862 | 0,076352 | n.s. |
| log.sigma.5.0.mm.3D_glcm_Id | 3,351595 | 0,009985 | 0,07681 | n.s. |
| log.sigma.4.0.mm.3D_firstorder_90Percentile | 3,332022 | 0,010303 | 0,078746 | n.s. |
| log.sigma.5.0.mm.3D_glcm_InverseVariance | 3,306058 | 0,010739 | 0,081566 | n.s. |
| log.sigma.5.0.mm.3D_glrlm_RunPercentage | 3,27851 | 0,011224 | 0,084709 | n.s. |
| wavelet.LLH_firstorder_Entropy | 3,263102 | 0,011505 | 0,086115 | n.s. |
| wavelet.LLH_gldm_DependenceNonUniformityNormalized | 3,260451 | 0,011554 | 0,086115 | n.s. |
| wavelet.HHL_glcm_ClusterProminence | 3,25462 | 0,011662 | 0,086388 | n.s. |
| wavelet.LHL_glszm_ZonePercentage | 3,248294 | 0,011781 | 0,086573 | n.s. |
| log.sigma.5.0.mm.3D_glcm_Idm | 3,245641 | 0,011832 | 0,086573 | n.s. |
| original_glrlm_ShortRunEmphasis | 3,231394 | 0,012106 | 0,08804 | n.s. |
| log.sigma.4.0.mm.3D_glrlm_RunLengthNonUniformityNormalized | 3,214366 | 0,012441 | 0,089488 | n.s. |
| log.sigma.5.0.mm.3D_glrlm_RunLengthNonUniformityNormalized | 3,213755 | 0,012454 | 0,089488 | n.s. |
| wavelet.LHH_glcm_ClusterProminence | 3,202853 | 0,012674 | 0,090529 | n.s. |
| wavelet.HHL_gldm_SmallDependenceHighGrayLevelEmphasis | 3,161043 | 0,013557 | 0,096245 | n.s. |
| wavelet.LLH_firstorder_90Percentile | 3,155414 | 0,01368 | 0,096245 | n.s. |
| log.sigma.2.0.mm.3D_glszm_SmallAreaHighGrayLevelEmphasis | 3,153854 | 0,013715 | 0,096245 | n.s. |
| wavelet.HLL_firstorder_Variance | 3,110758 | 0,014703 | 0,101913 | n.s. |
| log.sigma.4.0.mm.3D_firstorder_RootMeanSquared | 3,107637 | 0,014777 | 0,101913 | n.s. |
| log.sigma.5.0.mm.3D_firstorder_Median | 3,113508 | 0,014638 | 0,101913 | n.s. |
| original_glrlm_RunLengthNonUniformityNormalized | 3,101395 | 0,014927 | 0,102358 | n.s. |
| original_glcm_Idm | 3,080238 | 0,015447 | 0,10443 | n.s. |
| wavelet.HHL_glrlm_ShortRunHighGrayLevelEmphasis | 3,07872 | 0,015485 | 0,10443 | n.s. |
| log.sigma.4.0.mm.3D_firstorder_Mean | 3,078497 | 0,01549 | 0,10443 | n.s. |
| wavelet.HHL_glrlm_HighGrayLevelRunEmphasis | 3,050413 | 0,016211 | 0,108678 | n.s. |

| | | | | |
|---|---|---|---|---|
| wavelet.HLH_firstorder_Variance | 3,044146 | 0,016377 | 0,109178 | n.s. |
| wavelet.HHL_gldm_HighGrayLevelEmphasis | 3,037051 | 0,016566 | 0,109227 | n.s. |
| wavelet.LHL_firstorder_RobustMeanAbsoluteDeviation | 3,039736 | 0,016494 | 0,109227 | n.s. |
| log.sigma.3.0.mm.3D_glszm_GrayLevelVariance | 3,033304 | 0,016667 | 0,109292 | n.s. |
| wavelet.HHL_glcm_JointAverage | 3,029448 | 0,016772 | 0,10938 | n.s. |
| original_glcm_Id | 3,021116 | 0,017 | 0,110197 | n.s. |
| wavelet.HHH_firstorder_MeanAbsoluteDeviation | 3,016085 | 0,017139 | 0,110197 | n.s. |
| wavelet.LHL_glszm_ZoneEntropy | 3,013114 | 0,017222 | 0,110197 | n.s. |
| log.sigma.1.0.mm.3D_gldm_SmallDependenceEmphasis | 3,011609 | 0,017264 | 0,110197 | n.s. |
| wavelet.HHL_glcm_Autocorrelation | 3,007476 | 0,01738 | 0,110352 | n.s. |
| wavelet.HLL_glszm_SizeZoneNonUniformityNormalized | 2,985385 | 0,018015 | 0,113781 | n.s. |
| wavelet.HLL_gldm_SmallDependenceEmphasis | 2,972347 | 0,018401 | 0,115193 | n.s. |
| wavelet.HLH_glrlm_RunEntropy | 2,971354 | 0,018431 | 0,115193 | n.s. |
| wavelet.LHH_glszm_GrayLevelVariance | 2,959255 | 0,018797 | 0,116831 | n.s. |
| wavelet.LHL_firstorder_90Percentile | 2,956302 | 0,018888 | 0,116831 | n.s. |
| wavelet.HHH_firstorder_RobustMeanAbsoluteDeviation | 2,942743 | 0,019309 | 0,118825 | n.s. |
| wavelet.HLL_glszm_SmallAreaEmphasis | 2,915467 | 0,020186 | 0,123016 | n.s. |
| wavelet.HLH_glcm_DifferenceVariance | 2,915195 | 0,020195 | 0,123016 | n.s. |
| wavelet.HHL_gldm_SmallDependenceEmphasis | 2,902943 | 0,020603 | 0,123615 | n.s. |
| log.sigma.3.0.mm.3D_glcm_DifferenceEntropy | 2,903438 | 0,020586 | 0,123615 | n.s. |
| log.sigma.5.0.mm.3D_glcm_DifferenceAverage | 2,908782 | 0,020407 | 0,123615 | n.s. |
| wavelet.HLH_glrlm_GrayLevelVariance | 2,891919 | 0,020976 | 0,125231 | n.s. |
| wavelet.HHL_glszm_GrayLevelVariance | 2,884816 | 0,021221 | 0,126064 | n.s. |
| wavelet.LHL_firstorder_TotalEnergy | 2,469 | 0,021754 | 0,127963 | n.s. |
| wavelet.LHL_firstorder_Energy | 2,469 | 0,021754 | 0,127963 | n.s. |
| original_glcm_DifferenceEntropy | 2,858712 | 0,022145 | 0,129626 | n.s. |
| wavelet.LLL_glcm_JointEnergy | 2,855578 | 0,022258 | 0,129659 | n.s. |
| log.sigma.4.0.mm.3D_firstorder_Median | 2,832664 | 0,023107 | 0,133956 | n.s. |
| wavelet.HLH_glcm_ClusterProminence | 2,825407 | 0,023383 | 0,134432 | n.s. |
| log.sigma.4.0.mm.3D_glcm_Id | 2,824612 | 0,023414 | 0,134432 | n.s. |
| original_glcm_JointEnergy | 2,797211 | 0,024487 | 0,136672 | n.s. |
| wavelet.HLL_gldm_GrayLevelVariance | 2,80064 | 0,02435 | 0,136672 | n.s. |
| wavelet.HLL_glrlm_GrayLevelVariance | 2,802848 | 0,024262 | 0,136672 | n.s. |
| wavelet.LHL_firstorder_InterquartileRange | 2,802322 | 0,024283 | 0,136672 | n.s. |
| wavelet.LLL_glcm_MaximumProbability | 2,797955 | 0,024457 | 0,136672 | n.s. |
| log.sigma.1.0.mm.3D_glcm_InverseVariance | 2,808863 | 0,024025 | 0,136672 | n.s. |
| wavelet.HHH_firstorder_Variance | 2,789556 | 0,024796 | 0,137755 | n.s. |
| wavelet.HLH_glcm_ClusterTendency | 2,785028 | 0,02498 | 0,138141 | n.s. |
| wavelet.LLH_firstorder_MeanAbsoluteDeviation | 2,776065 | 0,02535 | 0,138903 | n.s. |
| wavelet.LLH_glcm_SumEntropy | 2,776488 | 0,025332 | 0,138903 | n.s. |
| log.sigma.1.0.mm.3D_glcm_DifferenceEntropy | 2,768738 | 0,025656 | 0,139942 | n.s. |
| log.sigma.5.0.mm.3D_firstorder_Maximum | 2,765653 | 0,025786 | 0,140015 | n.s. |
| wavelet.HLH_glcm_Idmn | 2,761687 | 0,025954 | 0,140293 | n.s. |
| log.sigma.5.0.mm.3D_glrlm_ShortRunEmphasis | 2,758381 | 0,026095 | 0,140423 | n.s. |
| original_glcm_Idn | 2,741017 | 0,026849 | 0,143833 | n.s. |
| log.sigma.4.0.mm.3D_glcm_Idm | 2,736163 | 0,027063 | 0,144338 | n.s. |

| | | | | |
|---|---|---|---|---|
| wavelet.HLL_firstorder_MeanAbsoluteDeviation | 2,719695 | 0,027805 | 0,144923 | n.s. |
| wavelet.HLH_gldm_GrayLevelVariance | 2,72321 | 0,027645 | 0,144923 | n.s. |
| wavelet.HLH_gldm_DependenceEntropy | 2,728553 | 0,027403 | 0,144923 | n.s. |
| wavelet.LLH_glcm_MaximumProbability | 2,715025 | 0,028018 | 0,144923 | n.s. |
| log.sigma.2.0.mm.3D_glszm_HighGrayLevelZoneEmphasis | 2,715856 | 0,02798 | 0,144923 | n.s. |
| log.sigma.4.0.mm.3D_glrlm_RunPercentage | 2,722567 | 0,027674 | 0,144923 | n.s. |
| log.sigma.5.0.mm.3D_gldm_LargeDependenceEmphasis | 2,717845 | 0,027889 | 0,144923 | n.s. |
| log.sigma.3.0.mm.3D_gldm_LargeDependenceEmphasis | 2,699457 | 0,028744 | 0,148037 | n.s. |
| original_glcm_MaximumProbability | 2,68297 | 0,029533 | 0,150145 | n.s. |
| wavelet.HLH_firstorder_Kurtosis | 2,682649 | 0,029548 | 0,150145 | n.s. |
| log.sigma.2.0.mm.3D_glszm_GrayLevelNonUniformityNormalized | 2,680486 | 0,029654 | 0,150145 | n.s. |
| log.sigma.2.0.mm.3D_glszm_SizeZoneNonUniformityNormalized | 2,683788 | 0,029493 | 0,150145 | n.s. |
| wavelet.HHH_glcm_MaximumProbability | 2,67676 | 0,029836 | 0,150276 | n.s. |
| log.sigma.3.0.mm.3D_firstorder_RootMeanSquared | 2,674845 | 0,02993 | 0,150276 | n.s. |
| log.sigma.3.0.mm.3D_firstorder_90Percentile | 2,662272 | 0,030555 | 0,152774 | n.s. |
| log.sigma.1.0.mm.3D_glcm_DifferenceAverage | 2,657092 | 0,030816 | 0,153441 | n.s. |
| log.sigma.3.0.mm.3D_glcm_InverseVariance | 2,65414 | 0,030966 | 0,153551 | n.s. |
| wavelet.HLH_firstorder_Entropy | 2,642446 | 0,031567 | 0,15525 | n.s. |
| log.sigma.1.0.mm.3D_glszm_SizeZoneNonUniformity | 2,642848 | 0,031547 | 0,15525 | n.s. |
| log.sigma.4.0.mm.3D_firstorder_Range | 2,633722 | 0,032024 | 0,156851 | n.s. |
| wavelet.HLH_glcm_DifferenceEntropy | 2,622525 | 0,032619 | 0,158474 | n.s. |
| log.sigma.3.0.mm.3D_firstorder_Mean | 2,623333 | 0,032576 | 0,158474 | n.s. |
| wavelet.HLH_glcm_SumEntropy | 2,619762 | 0,032768 | 0,158555 | n.s. |
| wavelet.HLH_glcm_SumSquares | 2,606242 | 0,033506 | 0,160332 | n.s. |
| wavelet.LLL_glrlm_RunVariance | 2,609836 | 0,033308 | 0,160332 | n.s. |
| log.sigma.1.0.mm.3D_glszm_GrayLevelVariance | 2,605689 | 0,033536 | 0,160332 | n.s. |
| log.sigma.5.0.mm.3D_firstorder_10Percentile | 2,602415 | 0,033717 | 0,160558 | n.s. |
| wavelet.HLL_firstorder_Entropy | 2,584926 | 0,034703 | 0,16395 | n.s. |
| wavelet.LLL_glcm_Idn | 2,585008 | 0,034698 | 0,16395 | n.s. |
| wavelet.HLH_firstorder_MeanAbsoluteDeviation | 2,575976 | 0,035218 | 0,165733 | n.s. |
| original_glcm_InverseVariance | 2,560206 | 0,036146 | 0,168265 | n.s. |
| original_glcm_DifferenceAverage | 2,554995 | 0,036457 | 0,168265 | n.s. |
| wavelet.LHL_firstorder_Maximum | 2,555254 | 0,036442 | 0,168265 | n.s. |
| log.sigma.2.0.mm.3D_firstorder_Range | 2,559504 | 0,036187 | 0,168265 | n.s. |
| log.sigma.4.0.mm.3D_glcm_InverseVariance | 2,558691 | 0,036236 | 0,168265 | n.s. |
| wavelet.HHL_glrlm_LongRunHighGrayLevelEmphasis | 2,531046 | 0,037926 | 0,174373 | n.s. |
| wavelet.LLL_glcm_Idmn | 2,527892 | 0,038124 | 0,174614 | n.s. |
| wavelet.HLL_glcm_JointEntropy | 2,52416 | 0,038359 | 0,175024 | n.s. |
| log.sigma.2.0.mm.3D_glcm_InverseVariance | 2,517504 | 0,038783 | 0,176286 | n.s. |
| wavelet.HHH_glrlm_RunEntropy | 2,510331 | 0,039245 | 0,177044 | n.s. |
| wavelet.LLL_gldm_LargeDependenceEmphasis | 2,511112 | 0,039194 | 0,177044 | n.s. |
| original_gldm_DependenceVariance | 2,490853 | 0,040527 | 0,178551 | n.s. |
| wavelet.HLL_glcm_DifferenceEntropy | 2,490121 | 0,040576 | 0,178551 | n.s. |
| wavelet.HLH_gldm_SmallDependenceEmphasis | 2,48946 | 0,04062 | 0,178551 | n.s. |
| log.sigma.1.0.mm.3D_glrlm_RunVariance | 2,490247 | 0,040568 | 0,178551 | n.s. |
| log.sigma.3.0.mm.3D_glrlm_RunPercentage | 2,493386 | 0,040358 | 0,178551 | n.s. |

| | | | | |
|---|---|---|---|---|
| log.sigma.3.0.mm.3D_glszm_SmallAreaHighGrayLevelEmphasis | 2,494906 | 0,040257 | 0,178551 | n.s. |
| log.sigma.4.0.mm.3D_glrlm_ShortRunEmphasis | 2,491021 | 0,040516 | 0,178551 | n.s. |
| wavelet.HLL_glcm_SumEntropy | 2,477545 | 0,041427 | 0,180774 | n.s. |
| wavelet.LHL_gldm_LargeDependenceLowGrayLevelEmphasis | 2,477779 | 0,041411 | 0,180774 | n.s. |
| wavelet.LHH_glcm_Imc2 | 2,470659 | 0,041901 | 0,182179 | n.s. |
| wavelet.HLH_firstorder_90Percentile | 2,461302 | 0,042554 | 0,183685 | n.s. |
| wavelet.HHH_firstorder_90Percentile | 2,463299 | 0,042414 | 0,183685 | n.s. |
| wavelet.HLH_glcm_JointEntropy | 2,445064 | 0,043711 | 0,188003 | n.s. |
| log.sigma.4.0.mm.3D_glcm_DifferenceAverage | 2,442723 | 0,04388 | 0,188058 | n.s. |
| wavelet.LLL_glrlm_LongRunEmphasis | 2,43901 | 0,04415 | 0,188541 | n.s. |
| wavelet.HLL_glcm_ClusterProminence | 2,430282 | 0,044792 | 0,189378 | n.s. |
| wavelet.LLH_gldm_DependenceVariance | 2,430989 | 0,044739 | 0,189378 | n.s. |
| wavelet.LLH_glrlm_RunEntropy | 2,429906 | 0,044819 | 0,189378 | n.s. |
| wavelet.HHL_firstorder_Skewness | 2,374398 | 0,045848 | 0,191175 | n.s. |
| wavelet.HHL_glszm_ZonePercentage | 2,415729 | 0,045882 | 0,191175 | n.s. |
| log.sigma.1.0.mm.3D_glcm_SumSquares | 2,419851 | 0,04557 | 0,191175 | n.s. |
| log.sigma.3.0.mm.3D_glrlm_RunEntropy | 2,420727 | 0,045505 | 0,191175 | n.s. |
| wavelet.HLL_glcm_ClusterTendency | 2,409202 | 0,04638 | 0,192563 | n.s. |
| wavelet.HLH_glcm_Contrast | 2,407168 | 0,046536 | 0,192563 | n.s. |
| wavelet.HHL_firstorder_Median | 2,361235 | 0,046898 | 0,193394 | n.s. |
| wavelet.HLL_glcm_DifferenceVariance | 2,399757 | 0,04711 | 0,193601 | n.s. |
| wavelet.LHH_gldm_SmallDependenceEmphasis | 2,389845 | 0,047888 | 0,196129 | n.s. |
| log.sigma.1.0.mm.3D_firstorder_90Percentile | 2,373512 | 0,049199 | 0,200813 | n.s. |
| wavelet.LLH_glszm_ZoneEntropy | 2,361341 | 0,0502 | 0,204202 | n.s. |
| wavelet.HLH_firstorder_Uniformity | 2,354791 | 0,050747 | 0,204565 | n.s. |
| wavelet.LLH_glcm_SumSquares | 2,352126 | 0,050971 | 0,204565 | n.s. |
| log.sigma.1.0.mm.3D_gldm_LargeDependenceEmphasis | 2,352814 | 0,050913 | 0,204565 | n.s. |
| log.sigma.3.0.mm.3D_glcm_Imc1 | 2,353877 | 0,050823 | 0,204565 | n.s. |
| log.sigma.2.0.mm.3D_firstorder_Maximum | 2,345374 | 0,051543 | 0,206173 | n.s. |
| log.sigma.1.0.mm.3D_glrlm_RunLengthNonUniformityNormalized | 2,339437 | 0,052052 | 0,20683 | n.s. |
| log.sigma.1.0.mm.3D_glrlm_RunPercentage | 2,33984 | 0,052017 | 0,20683 | n.s. |
| wavelet.HLH_glrlm_GrayLevelNonUniformityNormalized | 2,332672 | 0,052638 | 0,207965 | n.s. |
| log.sigma.1.0.mm.3D_glrlm_ShortRunEmphasis | 2,332139 | 0,052684 | 0,207965 | n.s. |
| log.sigma.4.0.mm.3D_firstorder_Kurtosis | 2,320412 | 0,053717 | 0,211344 | n.s. |
| log.sigma.2.0.mm.3D_glrlm_LongRunEmphasis | 2,315375 | 0,054166 | 0,212417 | n.s. |
| log.sigma.4.0.mm.3D_glszm_GrayLevelVariance | 2,309421 | 0,054703 | 0,213822 | n.s. |
| wavelet.HLL_glcm_SumSquares | 2,296734 | 0,055863 | 0,217478 | n.s. |
| wavelet.LLL_gldm_DependenceVariance | 2,295251 | 0,056001 | 0,217478 | n.s. |
| log.sigma.1.0.mm.3D_glrlm_LongRunEmphasis | 2,289955 | 0,056494 | 0,218685 | n.s. |
| log.sigma.2.0.mm.3D_gldm_LargeDependenceEmphasis | 2,287661 | 0,056709 | 0,218811 | n.s. |
| wavelet.HLL_glrlm_GrayLevelNonUniformityNormalized | 2,267646 | 0,058619 | 0,220656 | n.s. |
| wavelet.LHH_glszm_ZonePercentage | 2,268596 | 0,058526 | 0,220656 | n.s. |
| wavelet.LLH_glcm_Imc2 | 2,27318 | 0,058084 | 0,220656 | n.s. |
| wavelet.HHH_firstorder_InterquartileRange | 2,270559 | 0,058337 | 0,220656 | n.s. |
| wavelet.LHL_glrlm_LongRunLowGrayLevelEmphasis | 2,274518 | 0,057956 | 0,220656 | n.s. |
| log.sigma.1.0.mm.3D_glcm_Idm | 2,276001 | 0,057813 | 0,220656 | n.s. |

| | | | | |
|---|---|---|---|---|
| log.sigma.3.0.mm.3D_gldm_DependenceEntropy | 2,277481 | 0,057672 | 0,220656 | n.s. |
| log.sigma.3.0.mm.3D_glcm_Id | 2,267242 | 0,058658 | 0,220656 | n.s. |
| log.sigma.5.0.mm.3D_glcm_Idn | 2,257097 | 0,059651 | 0,223692 | n.s. |
| wavelet.HLH_glcm_Idn | 2,244418 | 0,060916 | 0,227724 | n.s. |
| log.sigma.1.0.mm.3D_glcm_Id | 2,242174 | 0,061143 | 0,227862 | n.s. |
| log.sigma.4.0.mm.3D_gldm_LargeDependenceEmphasis | 2,239828 | 0,061381 | 0,22804 | n.s. |
| log.sigma.3.0.mm.3D_firstorder_Median | 2,235479 | 0,061824 | 0,228979 | n.s. |
| wavelet.HHH_firstorder_Uniformity | 2,232681 | 0,062111 | 0,229334 | n.s. |
| wavelet.HHL_glcm_InverseVariance | 2,229167 | 0,062474 | 0,229964 | n.s. |
| original_glcm_Contrast | 2,224575 | 0,06295 | 0,23101 | n.s. |
| wavelet.HHH_glrlm_GrayLevelNonUniformityNormalized | 2,213219 | 0,064145 | 0,234676 | n.s. |
| wavelet.HHH_firstorder_Entropy | 2,208398 | 0,064659 | 0,235122 | n.s. |
| log.sigma.1.0.mm.3D_firstorder_Variance | 2,208949 | 0,0646 | 0,235122 | n.s. |
| wavelet.HHH_gldm_GrayLevelVariance | 2,203313 | 0,065205 | 0,235732 | n.s. |
| wavelet.HHH_glrlm_GrayLevelVariance | 2,203182 | 0,065219 | 0,235732 | n.s. |
| log.sigma.3.0.mm.3D_glcm_JointEntropy | 2,200763 | 0,065481 | 0,235967 | n.s. |
| wavelet.HLL_glszm_LowGrayLevelZoneEmphasis | 2,187411 | 0,066944 | 0,239086 | n.s. |
| wavelet.HLH_firstorder_Maximum | 2,190726 | 0,066578 | 0,239086 | n.s. |
| log.sigma.2.0.mm.3D_glszm_GrayLevelVariance | 2,188564 | 0,066816 | 0,239086 | n.s. |
| wavelet.HHH_glcm_ClusterTendency | 2,183606 | 0,067367 | 0,239882 | n.s. |
| wavelet.HHH_glcm_SumEntropy | 2,176824 | 0,068127 | 0,241872 | n.s. |
| log.sigma.1.0.mm.3D_gldm_GrayLevelVariance | 2,17235 | 0,068634 | 0,242237 | n.s. |
| log.sigma.3.0.mm.3D_glcm_Idm | 2,172896 | 0,068572 | 0,242237 | n.s. |
| log.sigma.2.0.mm.3D_glrlm_RunVariance | 2,168925 | 0,069024 | 0,242899 | n.s. |
| wavelet.HLL_glszm_ZonePercentage | 2,162603 | 0,06975 | 0,244736 | n.s. |
| wavelet.LLH_glszm_GrayLevelVariance | 2,155625 | 0,07056 | 0,246857 | n.s. |
| wavelet.HHH_glcm_JointEnergy | 2,152246 | 0,070955 | 0,247519 | n.s. |
| log.sigma.3.0.mm.3D_firstorder_Kurtosis | 2,148108 | 0,071443 | 0,248497 | n.s. |
| wavelet.LHH_firstorder_Minimum | 2,138223 | 0,072621 | 0,251865 | n.s. |
| wavelet.HLL_firstorder_Uniformity | 2,130465 | 0,073559 | 0,253171 | n.s. |
| wavelet.HLL_glrlm_RunEntropy | 2,126282 | 0,07407 | 0,253171 | n.s. |
| wavelet.HLH_glszm_ZoneEntropy | 2,118366 | 0,075046 | 0,253171 | n.s. |
| wavelet.HHL_gldm_LargeDependenceHighGrayLevelEmphasis | 2,12754 | 0,073916 | 0,253171 | n.s. |
| wavelet.LLH_gldm_GrayLevelVariance | 2,12978 | 0,073643 | 0,253171 | n.s. |
| wavelet.LLH_glrlm_GrayLevelVariance | 2,125613 | 0,074152 | 0,253171 | n.s. |
| wavelet.HHH_glcm_DifferenceVariance | 2,110734 | 0,076 | 0,253171 | n.s. |
| wavelet.LLL_firstorder_Uniformity | 2,123434 | 0,07442 | 0,253171 | n.s. |
| wavelet.LLL_glrlm_RunPercentage | 2,114336 | 0,075548 | 0,253171 | n.s. |
| log.sigma.1.0.mm.3D_glcm_ClusterTendency | 2,121714 | 0,074632 | 0,253171 | n.s. |
| log.sigma.2.0.mm.3D_glszm_GrayLevelNonUniformity | 2,115942 | 0,075348 | 0,253171 | n.s. |
| log.sigma.3.0.mm.3D_glcm_SumEntropy | 2,113619 | 0,075638 | 0,253171 | n.s. |
| log.sigma.3.0.mm.3D_glszm_HighGrayLevelZoneEmphasis | 2,107909 | 0,076356 | 0,253171 | n.s. |
| log.sigma.4.0.mm.3D_firstorder_TotalEnergy | 1,904065 | 0,076113 | 0,253171 | n.s. |
| log.sigma.4.0.mm.3D_firstorder_Energy | 1,904065 | 0,076113 | 0,253171 | n.s. |
| log.sigma.4.0.mm.3D_gldm_SmallDependenceEmphasis | 2,10777 | 0,076373 | 0,253171 | n.s. |
| wavelet.LLH_firstorder_Variance | 2,099709 | 0,077398 | 0,255862 | n.s. |

| | | | | |
|---|---|---|---|---|
| wavelet.HLL_gldm_SmallDependenceHighGrayLevelEmphasis | 2,089137 | 0,078763 | 0,259119 | n.s. |
| log.sigma.1.0.mm.3D_glrlm_GrayLevelVariance | 2,088736 | 0,078815 | 0,259119 | n.s. |
| wavelet.HHH_glcm_ClusterProminence | 2,08547 | 0,079242 | 0,259811 | n.s. |
| wavelet.LLL_glrlm_ShortRunEmphasis | 2,078705 | 0,080134 | 0,261735 | n.s. |
| log.sigma.4.0.mm.3D_glszm_SmallAreaHighGrayLevelEmphasis | 2,077711 | 0,080265 | 0,261735 | n.s. |
| wavelet.LLH_gldm_LargeDependenceLowGrayLevelEmphasis | 2,073824 | 0,080783 | 0,262708 | n.s. |
| wavelet.HLL_glszm_ZoneEntropy | 2,064508 | 0,082036 | 0,265038 | n.s. |
| wavelet.HLH_glrlm_RunVariance | 2,065039 | 0,081964 | 0,265038 | n.s. |
| wavelet.HHH_glcm_SumSquares | 2,063585 | 0,082162 | 0,265038 | n.s. |
| wavelet.HLH_glcm_DifferenceAverage | 2,061522 | 0,082442 | 0,26523 | n.s. |
| log.sigma.1.0.mm.3D_firstorder_Range | 2,056812 | 0,083087 | 0,266588 | n.s. |
| wavelet.HLL_glrlm_ShortRunLowGrayLevelEmphasis | 2,042836 | 0,085028 | 0,270024 | n.s. |
| wavelet.HHH_glcm_JointEntropy | 2,04417 | 0,084841 | 0,270024 | n.s. |
| log.sigma.2.0.mm.3D_glcm_DifferenceEntropy | 2,042624 | 0,085058 | 0,270024 | n.s. |
| log.sigma.5.0.mm.3D_glrlm_ShortRunHighGrayLevelEmphasis | 2,042703 | 0,085046 | 0,270024 | n.s. |
| wavelet.HHH_glcm_DifferenceEntropy | 2,038959 | 0,085574 | 0,270947 | n.s. |
| wavelet.HLL_glcm_JointEnergy | 2,027253 | 0,087245 | 0,27551 | n.s. |
| wavelet.HLH_glrlm_LongRunEmphasis | 2,020467 | 0,088228 | 0,277884 | n.s. |
| wavelet.HLL_glrlm_LowGrayLevelRunEmphasis | 2,002011 | 0,090958 | 0,284375 | n.s. |
| wavelet.LHL_glszm_SizeZoneNonUniformity | 2,001732 | 0,091 | 0,284375 | n.s. |
| log.sigma.3.0.mm.3D_glrlm_ShortRunEmphasis | 2,004752 | 0,090547 | 0,284375 | n.s. |
| wavelet.HLL_gldm_SmallDependenceLowGrayLevelEmphasis | 1,987939 | 0,093095 | 0,289415 | n.s. |
| log.sigma.3.0.mm.3D_firstorder_Entropy | 1,988613 | 0,092992 | 0,289415 | n.s. |
| wavelet.HLH_glcm_JointEnergy | 1,980141 | 0,094301 | 0,292405 | n.s. |
| wavelet.LLL_glrlm_GrayLevelNonUniformityNormalized | 1,972448 | 0,095505 | 0,295376 | n.s. |
| wavelet.HLL_gldm_LowGrayLevelEmphasis | 1,970819 | 0,095762 | 0,295409 | n.s. |
| log.sigma.5.0.mm.3D_glcm_JointAverage | 1,961841 | 0,09719 | 0,299046 | n.s. |
| original_firstorder_Uniformity | 1,952429 | 0,09871 | 0,299121 | n.s. |
| wavelet.HLL_glcm_MaximumProbability | 1,958241 | 0,097769 | 0,299121 | n.s. |
| wavelet.HLL_glrlm_RunVariance | 1,958792 | 0,09768 | 0,299121 | n.s. |
| wavelet.HLL_glrlm_RunPercentage | 1,953214 | 0,098582 | 0,299121 | n.s. |
| wavelet.HLH_firstorder_RobustMeanAbsoluteDeviation | 1,955959 | 0,098137 | 0,299121 | n.s. |
| log.sigma.2.0.mm.3D_firstorder_Median | 1,955051 | 0,098284 | 0,299121 | n.s. |
| wavelet.HHL_firstorder_Range | 1,93924 | 0,100879 | 0,299739 | n.s. |
| wavelet.HHH_glcm_Contrast | 1,9464 | 0,099696 | 0,299739 | n.s. |
| log.sigma.2.0.mm.3D_firstorder_RootMeanSquared | 1,939103 | 0,100902 | 0,299739 | n.s. |
| log.sigma.2.0.mm.3D_glrlm_RunPercentage | 1,945022 | 0,099922 | 0,299739 | n.s. |
| log.sigma.3.0.mm.3D_glcm_DifferenceAverage | 1,939041 | 0,100912 | 0,299739 | n.s. |
| log.sigma.4.0.mm.3D_glszm_HighGrayLevelZoneEmphasis | 1,941026 | 0,100582 | 0,299739 | n.s. |
| log.sigma.5.0.mm.3D_firstorder_TotalEnergy | 1,769728 | 0,100912 | 0,299739 | n.s. |
| log.sigma.5.0.mm.3D_firstorder_Energy | 1,769728 | 0,100912 | 0,299739 | n.s. |
| wavelet.LHH_glszm_LargeAreaEmphasis | 1,933887 | 0,101773 | 0,300699 | n.s. |
| wavelet.LLL_glrlm_RunLengthNonUniformityNormalized | 1,934256 | 0,101711 | 0,300699 | n.s. |
| log.sigma.4.0.mm.3D_firstorder_MeanAbsoluteDeviation | 1,932609 | 0,101987 | 0,300699 | n.s. |
| wavelet.LHH_glszm_ZoneVariance | 1,926679 | 0,102988 | 0,302906 | n.s. |
| wavelet.HLL_glcm_Id | 1,920007 | 0,104126 | 0,305485 | n.s. |

| | | | | |
|---|---|---|---|---|
| wavelet.HLL_glrlm_LongRunEmphasis | 1,91856 | 0,104374 | 0,305485 | n.s. |
| wavelet.HLL_glcm_DifferenceAverage | 1,912593 | 0,105404 | 0,30775 | n.s. |
| wavelet.HLL_glcm_Contrast | 1,910229 | 0,105815 | 0,3082 | n.s. |
| wavelet.HLL_firstorder_RobustMeanAbsoluteDeviation | 1,903006 | 0,107081 | 0,309896 | n.s. |
| log.sigma.2.0.mm.3D_gldm_SmallDependenceEmphasis | 1,902486 | 0,107172 | 0,309896 | n.s. |
| log.sigma.5.0.mm.3D_firstorder_Entropy | 1,904196 | 0,106871 | 0,309896 | n.s. |
| wavelet.HLL_glcm_Idm | 1,896694 | 0,108198 | 0,311664 | n.s. |
| log.sigma.3.0.mm.3D_glcm_JointEnergy | 1,896105 | 0,108303 | 0,311664 | n.s. |
| wavelet.LLH_glcm_ClusterTendency | 1,891949 | 0,109046 | 0,313051 | n.s. |
| log.sigma.1.0.mm.3D_firstorder_InterquartileRange | 1,882733 | 0,110711 | 0,317073 | n.s. |
| original_gldm_SmallDependenceEmphasis | 1,879605 | 0,111282 | 0,317194 | n.s. |
| log.sigma.2.0.mm.3D_glszm_ZoneEntropy | 1,880197 | 0,111174 | 0,317194 | n.s. |
| wavelet.LLH_firstorder_10Percentile | 1,869941 | 0,113064 | 0,319436 | n.s. |
| wavelet.HHH_firstorder_10Percentile | 1,869564 | 0,113134 | 0,319436 | n.s. |
| wavelet.LHL_gldm_DependenceNonUniformity | 1,869617 | 0,113124 | 0,319436 | n.s. |
| log.sigma.2.0.mm.3D_firstorder_Mean | 1,871868 | 0,112706 | 0,319436 | n.s. |
| wavelet.HLH_firstorder_Skewness | 1,866147 | 0,113771 | 0,319907 | n.s. |
| log.sigma.2.0.mm.3D_glrlm_ShortRunEmphasis | 1,86581 | 0,113834 | 0,319907 | n.s. |
| wavelet.LHL_gldm_LowGrayLevelEmphasis | 1,862302 | 0,114491 | 0,321004 | n.s. |
| wavelet.LHL_glrlm_LowGrayLevelRunEmphasis | 1,8577 | 0,11536 | 0,322686 | n.s. |
| wavelet.HLL_glrlm_ShortRunEmphasis | 1,853535 | 0,116152 | 0,324145 | n.s. |
| original_glszm_ZonePercentage | 1,850646 | 0,116704 | 0,324178 | n.s. |
| wavelet.HHH_glrlm_GrayLevelNonUniformity | 1,850753 | 0,116684 | 0,324178 | n.s. |
| log.sigma.5.0.mm.3D_gldm_DependenceVariance | 1,847307 | 0,117346 | 0,325207 | n.s. |
| log.sigma.4.0.mm.3D_firstorder_Entropy | 1,842385 | 0,118297 | 0,327089 | n.s. |
| log.sigma.3.0.mm.3D_glcm_DifferenceVariance | 1,840639 | 0,118637 | 0,327274 | n.s. |
| log.sigma.1.0.mm.3D_glszm_ZonePercentage | 1,833954 | 0,119945 | 0,330124 | n.s. |
| wavelet.HLL_firstorder_TotalEnergy | 1,676971 | 0,122072 | 0,3331 | n.s. |
| wavelet.HLL_firstorder_Energy | 1,676971 | 0,122072 | 0,3331 | n.s. |
| wavelet.LHH_firstorder_RootMeanSquared | 1,798156 | 0,122804 | 0,3331 | n.s. |
| wavelet.HLH_glcm_Idm | 1,815399 | 0,12365 | 0,3331 | n.s. |
| wavelet.HHH_glszm_ZonePercentage | 1,81465 | 0,123802 | 0,3331 | n.s. |
| log.sigma.1.0.mm.3D_firstorder_Maximum | 1,819118 | 0,122899 | 0,3331 | n.s. |
| log.sigma.1.0.mm.3D_gldm_SmallDependenceHighGrayLevelEmphasis | 1,819586 | 0,122805 | 0,3331 | n.s. |
| log.sigma.3.0.mm.3D_glrlm_RunLengthNonUniformityNormalized | 1,814685 | 0,123795 | 0,3331 | n.s. |
| log.sigma.4.0.mm.3D_glrlm_RunEntropy | 1,815652 | 0,123599 | 0,3331 | n.s. |
| log.sigma.5.0.mm.3D_glcm_JointEntropy | 1,819891 | 0,122743 | 0,3331 | n.s. |
| wavelet.HHH_glcm_DifferenceAverage | 1,809709 | 0,124808 | 0,335056 | n.s. |
| original_glcm_DifferenceVariance | 1,798271 | 0,127168 | 0,339905 | n.s. |
| wavelet.LLL_glszm_GrayLevelNonUniformity | 1,79821 | 0,127181 | 0,339905 | n.s. |
| wavelet.HLL_glszm_SmallAreaLowGrayLevelEmphasis | 1,796055 | 0,127631 | 0,340349 | n.s. |
| log.sigma.1.0.mm.3D_glcm_JointEntropy | 1,792556 | 0,128364 | 0,341544 | n.s. |
| log.sigma.4.0.mm.3D_glcm_JointEntropy | 1,788562 | 0,129205 | 0,343023 | n.s. |
| log.sigma.5.0.mm.3D_glcm_Contrast | 1,784262 | 0,130118 | 0,344683 | n.s. |
| wavelet.HLL_glrlm_LongRunLowGrayLevelEmphasis | 1,779829 | 0,131065 | 0,344999 | n.s. |
| wavelet.HLH_firstorder_10Percentile | 1,779667 | 0,131099 | 0,344999 | n.s. |

| | | | | |
|---|---|---|---|---|
| wavelet.HLH_glszm_ZonePercentage | 1,780077 | 0,131012 | 0,344999 | n.s. |
| wavelet.HHL_gldm_SmallDependenceLowGrayLevelEmphasis | 1,776333 | 0,131816 | 0,34537 | n.s. |
| log.sigma.2.0.mm.3D_glszm_ZonePercentage | 1,777365 | 0,131594 | 0,34537 | n.s. |
| log.sigma.2.0.mm.3D_glcm_JointEnergy | 1,774764 | 0,132155 | 0,345502 | n.s. |
| wavelet.HLH_firstorder_Mean | 1,75092 | 0,13298 | 0,345904 | n.s. |
| wavelet.LLL_glcm_Idm | 1,770068 | 0,133173 | 0,345904 | n.s. |
| log.sigma.4.0.mm.3D_firstorder_10Percentile | 1,770619 | 0,133053 | 0,345904 | n.s. |
| wavelet.HHH_gldm_SmallDependenceEmphasis | 1,76578 | 0,13411 | 0,346914 | n.s. |
| wavelet.HHH_gldm_GrayLevelNonUniformity | 1,765641 | 0,13414 | 0,346914 | n.s. |
| wavelet.LHH_glrlm_GrayLevelNonUniformity | 1,764013 | 0,134497 | 0,34709 | n.s. |
| original_firstorder_Maximum | 1,759411 | 0,135512 | 0,348959 | n.s. |
| wavelet.HLL_gldm_LargeDependenceEmphasis | 1,754516 | 0,1366 | 0,350257 | n.s. |
| wavelet.HLL_glrlm_RunLengthNonUniformityNormalized | 1,754534 | 0,136596 | 0,350257 | n.s. |
| wavelet.HLL_firstorder_Kurtosis | 1,751573 | 0,137258 | 0,350446 | n.s. |
| wavelet.HLH_glcm_MaximumProbability | 1,752838 | 0,136975 | 0,350446 | n.s. |
| log.sigma.5.0.mm.3D_firstorder_InterquartileRange | 1,746858 | 0,138319 | 0,352404 | n.s. |
| wavelet.HLH_glcm_ClusterShade | 1,738692 | 0,140174 | 0,355007 | n.s. |
| wavelet.HHL_glcm_Idmn | 1,739611 | 0,139964 | 0,355007 | n.s. |
| wavelet.HHH_glcm_Idm | 1,738463 | 0,140228 | 0,355007 | n.s. |
| wavelet.HLH_firstorder_Range | 1,732133 | 0,141681 | 0,357932 | n.s. |
| wavelet.LHL_glszm_LowGrayLevelZoneEmphasis | 1,728409 | 0,142544 | 0,359355 | n.s. |
| wavelet.LLH_gldm_DependenceEntropy | 1,726637 | 0,142956 | 0,359639 | n.s. |
| wavelet.LLH_firstorder_Maximum | 1,724549 | 0,143444 | 0,360109 | n.s. |
| log.sigma.2.0.mm.3D_glszm_LowGrayLevelZoneEmphasis | 1,720843 | 0,144312 | 0,361534 | n.s. |
| wavelet.HLH_glcm_Id | 1,716577 | 0,145319 | 0,363297 | n.s. |
| wavelet.LHL_firstorder_RootMeanSquared | 1,711832 | 0,146446 | 0,365354 | n.s. |
| wavelet.LHH_gldm_SmallDependenceHighGrayLevelEmphasis | 1,708763 | 0,14718 | 0,366422 | n.s. |
| wavelet.LLH_glrlm_LongRunLowGrayLevelEmphasis | 1,702557 | 0,148673 | 0,369375 | n.s. |
| wavelet.LHH_glcm_Imc1 | 1,687668 | 0,152317 | 0,371443 | n.s. |
| wavelet.HLH_gldm_SmallDependenceHighGrayLevelEmphasis | 1,695181 | 0,150468 | 0,371443 | n.s. |
| wavelet.HLH_glrlm_ShortRunEmphasis | 1,677934 | 0,154745 | 0,371443 | n.s. |
| wavelet.HLH_glrlm_RunPercentage | 1,695115 | 0,150484 | 0,371443 | n.s. |
| wavelet.HHL_glcm_Idn | 1,677841 | 0,154768 | 0,371443 | n.s. |
| wavelet.HHL_glszm_GrayLevelNonUniformity | 1,689257 | 0,151924 | 0,371443 | n.s. |
| wavelet.HHH_glcm_Id | 1,678581 | 0,154582 | 0,371443 | n.s. |
| wavelet.HHH_glrlm_RunLengthNonUniformity | 1,678141 | 0,154693 | 0,371443 | n.s. |
| wavelet.HHH_glszm_LargeAreaHighGrayLevelEmphasis | 1,572662 | 0,150503 | 0,371443 | n.s. |
| wavelet.LHL_glrlm_ShortRunLowGrayLevelEmphasis | 1,680882 | 0,154005 | 0,371443 | n.s. |
| wavelet.LLL_glcm_InverseVariance | 1,684539 | 0,153093 | 0,371443 | n.s. |
| log.sigma.2.0.mm.3D_glcm_Id | 1,686983 | 0,152487 | 0,371443 | n.s. |
| log.sigma.3.0.mm.3D_glrlm_GrayLevelNonUniformityNormalized | 1,689798 | 0,15179 | 0,371443 | n.s. |
| log.sigma.3.0.mm.3D_glszm_GrayLevelNonUniformityNormalized | 1,693638 | 0,150846 | 0,371443 | n.s. |
| log.sigma.4.0.mm.3D_firstorder_RobustMeanAbsoluteDeviation | 1,691104 | 0,151469 | 0,371443 | n.s. |
| log.sigma.4.0.mm.3D_glcm_JointAverage | 1,678134 | 0,154694 | 0,371443 | n.s. |
| log.sigma.5.0.mm.3D_firstorder_MeanAbsoluteDeviation | 1,680042 | 0,154216 | 0,371443 | n.s. |
| log.sigma.5.0.mm.3D_firstorder_RobustMeanAbsoluteDeviation | 1,675327 | 0,155401 | 0,372218 | n.s. |

| | | | | |
|---|---|---|---|---|
| log.sigma.1.0.mm.3D_glcm_JointEnergy | 1,668604 | 0,157106 | 0,375552 | n.s. |
| original_gldm_SmallDependenceLowGrayLevelEmphasis | 1,665609 | 0,157871 | 0,375884 | n.s. |
| original_glrlm_GrayLevelNonUniformityNormalized | 1,665823 | 0,157817 | 0,375884 | n.s. |
| wavelet.HHH_glrlm_RunLengthNonUniformityNormalized | 1,662211 | 0,158744 | 0,377213 | n.s. |
| log.sigma.2.0.mm.3D_glrlm_RunLengthNonUniformityNormalized | 1,659747 | 0,159379 | 0,377975 | n.s. |
| log.sigma.4.0.mm.3D_glcm_Correlation | 1,654307 | 0,160791 | 0,38057 | n.s. |
| wavelet.HLL_glszm_GrayLevelNonUniformityNormalized | 1,64769 | 0,162524 | 0,383161 | n.s. |
| log.sigma.2.0.mm.3D_glcm_DifferenceVariance | 1,648484 | 0,162315 | 0,383161 | n.s. |
| log.sigma.4.0.mm.3D_firstorder_InterquartileRange | 1,645285 | 0,163158 | 0,383902 | n.s. |
| wavelet.LLH_firstorder_TotalEnergy | 1,527806 | 0,16441 | 0,385001 | n.s. |
| wavelet.LLH_firstorder_Energy | 1,527806 | 0,16441 | 0,385001 | n.s. |
| log.sigma.5.0.mm.3D_glszm_GrayLevelNonUniformityNormalized | 1,639896 | 0,164588 | 0,385001 | n.s. |
| wavelet.HLL_firstorder_RootMeanSquared | 1,636211 | 0,165573 | 0,386551 | n.s. |
| wavelet.HLL_gldm_LargeDependenceLowGrayLevelEmphasis | 1,632278 | 0,166629 | 0,388263 | n.s. |
| wavelet.HLH_gldm_LargeDependenceEmphasis | 1,629625 | 0,167346 | 0,389177 | n.s. |
| wavelet.HLL_glszm_GrayLevelVariance | 1,625803 | 0,168383 | 0,390077 | n.s. |
| log.sigma.4.0.mm.3D_glcm_SumEntropy | 1,626781 | 0,168117 | 0,390077 | n.s. |
| wavelet.LHH_firstorder_TotalEnergy | 1,512678 | 0,169345 | 0,390796 | n.s. |
| wavelet.LHH_firstorder_Energy | 1,512678 | 0,169345 | 0,390796 | n.s. |
| wavelet.HLL_glcm_Imc2 | 1,619745 | 0,17004 | 0,391129 | n.s. |
| log.sigma.4.0.mm.3D_glrlm_GrayLevelVariance | 1,619376 | 0,170141 | 0,391129 | n.s. |
| log.sigma.3.0.mm.3D_glrlm_GrayLevelVariance | 1,614534 | 0,171477 | 0,393447 | n.s. |
| wavelet.LHH_glrlm_RunLengthNonUniformity | 1,613206 | 0,171845 | 0,393539 | n.s. |
| original_glcm_JointEntropy | 1,60862 | 0,173122 | 0,395337 | n.s. |
| log.sigma.3.0.mm.3D_gldm_SmallDependenceEmphasis | 1,608023 | 0,173289 | 0,395337 | n.s. |
| wavelet.HLH_firstorder_InterquartileRange | 1,601535 | 0,175112 | 0,395945 | n.s. |
| wavelet.HHL_gldm_DependenceVariance | 1,601206 | 0,175205 | 0,395945 | n.s. |
| wavelet.LHL_glrlm_GrayLevelNonUniformity | 1,605836 | 0,173902 | 0,395945 | n.s. |
| log.sigma.2.0.mm.3D_glcm_Idm | 1,604124 | 0,174383 | 0,395945 | n.s. |
| log.sigma.5.0.mm.3D_glcm_Idmn | 1,603528 | 0,17455 | 0,395945 | n.s. |
| wavelet.LLL_glcm_Id | 1,59669 | 0,176486 | 0,398088 | n.s. |
| wavelet.HLL_glszm_SizeZoneNonUniformity | 1,587448 | 0,179134 | 0,398108 | n.s. |
| wavelet.LHH_gldm_GrayLevelNonUniformity | 1,588582 | 0,178807 | 0,398108 | n.s. |
| wavelet.HHH_glrlm_ShortRunEmphasis | 1,594591 | 0,177084 | 0,398108 | n.s. |
| log.sigma.3.0.mm.3D_firstorder_TotalEnergy | 1,487489 | 0,177844 | 0,398108 | n.s. |
| log.sigma.3.0.mm.3D_firstorder_Energy | 1,487489 | 0,177844 | 0,398108 | n.s. |
| log.sigma.3.0.mm.3D_firstorder_10Percentile | 1,587397 | 0,179148 | 0,398108 | n.s. |
| log.sigma.3.0.mm.3D_glrlm_RunVariance | 1,589277 | 0,178607 | 0,398108 | n.s. |
| log.sigma.4.0.mm.3D_glrlm_ShortRunHighGrayLevelEmphasis | 1,589258 | 0,178612 | 0,398108 | n.s. |
| log.sigma.1.0.mm.3D_firstorder_RobustMeanAbsoluteDeviation | 1,582259 | 0,180637 | 0,399933 | n.s. |
| log.sigma.3.0.mm.3D_firstorder_Uniformity | 1,582306 | 0,180623 | 0,399933 | n.s. |
| wavelet.LLL_glcm_DifferenceEntropy | 1,579501 | 0,18144 | 0,400235 | n.s. |
| wavelet.LLL_glszm_ZonePercentage | 1,580044 | 0,181282 | 0,400235 | n.s. |
| wavelet.HLL_firstorder_InterquartileRange | 1,577194 | 0,182115 | 0,400252 | n.s. |
| wavelet.LLH_glszm_SmallAreaLowGrayLevelEmphasis | 1,577748 | 0,181952 | 0,400252 | n.s. |
| wavelet.LHL_glrlm_RunLengthNonUniformity | 1,574363 | 0,182946 | 0,401344 | n.s. |

| | | | | |
|---|---|---|---|---|
| wavelet.LHL_glszm_SmallAreaLowGrayLevelEmphasis | 1,572298 | 0,183554 | 0,401944 | n.s. |
| wavelet.LLL_firstorder_Range | 1,567084 | 0,185099 | 0,403892 | n.s. |
| log.sigma.4.0.mm.3D_glcm_Contrast | 1,567025 | 0,185117 | 0,403892 | n.s. |
| wavelet.HLL_gldm_DependenceEntropy | 1,564014 | 0,186015 | 0,405114 | n.s. |
| log.sigma.5.0.mm.3D_glcm_ClusterShade | 1,561941 | 0,186635 | 0,405729 | n.s. |
| log.sigma.3.0.mm.3D_glszm_ZoneEntropy | 1,55699 | 0,188125 | 0,408227 | n.s. |
| original_glszm_SizeZoneNonUniformity | 1,549833 | 0,190298 | 0,411455 | n.s. |
| wavelet.LLH_firstorder_Range | 1,550177 | 0,190193 | 0,411455 | n.s. |
| wavelet.HHH_glrlm_RunPercentage | 1,548668 | 0,190654 | 0,411483 | n.s. |
| log.sigma.5.0.mm.3D_glrlm_GrayLevelNonUniformityNormalized | 1,54726 | 0,191085 | 0,411673 | n.s. |
| wavelet.LLH_firstorder_RootMeanSquared | 1,545716 | 0,191558 | 0,411953 | n.s. |
| original_firstorder_Kurtosis | 1,539316 | 0,193533 | 0,413975 | n.s. |
| original_glszm_SmallAreaLowGrayLevelEmphasis | 1,540606 | 0,193133 | 0,413975 | n.s. |
| wavelet.HLH_gldm_LowGrayLevelEmphasis | 1,539386 | 0,193511 | 0,413975 | n.s. |
| wavelet.HHL_firstorder_Kurtosis | 1,537508 | 0,194095 | 0,414261 | n.s. |
| log.sigma.5.0.mm.3D_glcm_SumEntropy | 1,536662 | 0,194358 | 0,414261 | n.s. |
| wavelet.HLL_firstorder_Range | 1,528584 | 0,196887 | 0,415713 | n.s. |
| wavelet.HLL_glcm_JointAverage | 1,531747 | 0,195893 | 0,415713 | n.s. |
| wavelet.HLH_glrlm_ShortRunLowGrayLevelEmphasis | 1,529075 | 0,196733 | 0,415713 | n.s. |
| wavelet.LLL_firstorder_Minimum | 1,528806 | 0,196817 | 0,415713 | n.s. |
| log.sigma.3.0.mm.3D_glrlm_LongRunEmphasis | 1,528082 | 0,197045 | 0,415713 | n.s. |
| log.sigma.5.0.mm.3D_glcm_Autocorrelation | 1,527854 | 0,197117 | 0,415713 | n.s. |
| log.sigma.1.0.mm.3D_firstorder_Entropy | 1,525371 | 0,197902 | 0,416635 | n.s. |
| log.sigma.2.0.mm.3D_firstorder_90Percentile | 1,522074 | 0,198948 | 0,418105 | n.s. |
| wavelet.HHL_glrlm_RunLengthNonUniformity | 1,518819 | 0,199986 | 0,418934 | n.s. |
| log.sigma.2.0.mm.3D_glcm_DifferenceAverage | 1,518647 | 0,200041 | 0,418934 | n.s. |
| log.sigma.2.0.mm.3D_glcm_JointEntropy | 1,515804 | 0,200952 | 0,420108 | n.s. |
| wavelet.HLH_glrlm_LowGrayLevelRunEmphasis | 1,513739 | 0,201616 | 0,420522 | n.s. |
| wavelet.HHL_glrlm_ShortRunLowGrayLevelEmphasis | 1,51301 | 0,201851 | 0,420522 | n.s. |
| wavelet.LLH_glszm_GrayLevelNonUniformity | 1,510674 | 0,202605 | 0,421229 | n.s. |
| wavelet.LHL_gldm_GrayLevelNonUniformity | 1,509787 | 0,202892 | 0,421229 | n.s. |
| log.sigma.3.0.mm.3D_glcm_Correlation | 1,507466 | 0,203645 | 0,422062 | n.s. |
| wavelet.HHL_gldm_LowGrayLevelEmphasis | 1,500134 | 0,206041 | 0,426291 | n.s. |
| original_firstorder_Minimum | 1,495559 | 0,207549 | 0,427434 | n.s. |
| wavelet.LHH_firstorder_Mean | 1,494361 | 0,207945 | 0,427434 | n.s. |
| wavelet.HLH_glrlm_RunLengthNonUniformityNormalized | 1,494544 | 0,207885 | 0,427434 | n.s. |
| wavelet.HHL_glrlm_LowGrayLevelRunEmphasis | 1,492936 | 0,208418 | 0,427434 | n.s. |
| wavelet.LHL_firstorder_Range | 1,491996 | 0,20873 | 0,427434 | n.s. |
| wavelet.LLL_gldm_SmallDependenceEmphasis | 1,493853 | 0,208114 | 0,427434 | n.s. |
| wavelet.HHL_gldm_LargeDependenceLowGrayLevelEmphasis | 1,481581 | 0,212219 | 0,42803 | n.s. |
| wavelet.LLH_gldm_SmallDependenceHighGrayLevelEmphasis | 1,483935 | 0,211426 | 0,42803 | n.s. |
| wavelet.LLH_glszm_SizeZoneNonUniformity | 1,481544 | 0,212232 | 0,42803 | n.s. |
| wavelet.HHH_gldm_LargeDependenceEmphasis | 1,486176 | 0,210673 | 0,42803 | n.s. |
| wavelet.HHH_glszm_GrayLevelNonUniformity | 1,481877 | 0,212119 | 0,42803 | n.s. |
| wavelet.LLL_firstorder_Kurtosis | 1,482806 | 0,211806 | 0,42803 | n.s. |
| log.sigma.1.0.mm.3D_firstorder_MeanAbsoluteDeviation | 1,48304 | 0,211727 | 0,42803 | n.s. |

| | | | | |
|---|---|---|---|---|
| log.sigma.2.0.mm.3D_firstorder_Kurtosis | 1,485469 | 0,210911 | 0,42803 | n.s. |
| log.sigma.3.0.mm.3D_glszm_SmallAreaLowGrayLevelEmphasis | 1,484696 | 0,21117 | 0,42803 | n.s. |
| wavelet.HLL_firstorder_Minimum | 1,479838 | 0,212808 | 0,428473 | n.s. |
| original_glszm_LowGrayLevelZoneEmphasis | 1,471848 | 0,215528 | 0,429493 | n.s. |
| wavelet.HLL_glszm_SmallAreaHighGrayLevelEmphasis | 1,474708 | 0,214551 | 0,429493 | n.s. |
| wavelet.LLL_firstorder_Maximum | 1,470995 | 0,21582 | 0,429493 | n.s. |
| log.sigma.1.0.mm.3D_glszm_GrayLevelNonUniformity | 1,476732 | 0,213862 | 0,429493 | n.s. |
| log.sigma.3.0.mm.3D_glszm_ZonePercentage | 1,47298 | 0,215141 | 0,429493 | n.s. |
| log.sigma.5.0.mm.3D_gldm_GrayLevelVariance | 1,474119 | 0,214752 | 0,429493 | n.s. |
| log.sigma.5.0.mm.3D_glrlm_LowGrayLevelRunEmphasis | 1,47408 | 0,214765 | 0,429493 | n.s. |
| wavelet.LLH_firstorder_Median | 1,46815 | 0,216797 | 0,430723 | n.s. |
| original_firstorder_Range | 1,461231 | 0,219191 | 0,434759 | n.s. |
| log.sigma.4.0.mm.3D_gldm_SmallDependenceLowGrayLevelEmphasis | 1,458157 | 0,220262 | 0,436163 | n.s. |
| wavelet.LLH_firstorder_Mean | 1,455706 | 0,221119 | 0,437139 | n.s. |
| wavelet.HLL_glrlm_ShortRunHighGrayLevelEmphasis | 1,452778 | 0,222148 | 0,438158 | n.s. |
| wavelet.LLL_glszm_SizeZoneNonUniformityNormalized | 1,451735 | 0,222515 | 0,438158 | n.s. |
| log.sigma.4.0.mm.3D_firstorder_Variance | 1,451125 | 0,22273 | 0,438158 | n.s. |
| wavelet.LLH_glszm_LowGrayLevelZoneEmphasis | 1,447117 | 0,224148 | 0,440226 | n.s. |
| wavelet.HLH_firstorder_RootMeanSquared | 1,44405 | 0,225239 | 0,441645 | n.s. |
| log.sigma.5.0.mm.3D_glszm_HighGrayLevelZoneEmphasis | 1,435985 | 0,22813 | 0,446583 | n.s. |
| log.sigma.2.0.mm.3D_glrlm_LongRunHighGrayLevelEmphasis | 1,434063 | 0,228824 | 0,447212 | n.s. |
| log.sigma.4.0.mm.3D_glszm_SmallAreaLowGrayLevelEmphasis | 1,432452 | 0,229407 | 0,447623 | n.s. |
| log.sigma.4.0.mm.3D_gldm_GrayLevelVariance | 1,424992 | 0,232124 | 0,45219 | n.s. |
| wavelet.HLL_gldm_HighGrayLevelEmphasis | 1,410369 | 0,237536 | 0,455961 | n.s. |
| wavelet.HLL_glrlm_HighGrayLevelRunEmphasis | 1,411783 | 0,237008 | 0,455961 | n.s. |
| wavelet.HLL_glszm_HighGrayLevelZoneEmphasis | 1,416224 | 0,235356 | 0,455961 | n.s. |
| wavelet.HLH_glrlm_LongRunLowGrayLevelEmphasis | 1,412509 | 0,236737 | 0,455961 | n.s. |
| wavelet.HHL_glrlm_GrayLevelNonUniformity | 1,408489 | 0,23824 | 0,455961 | n.s. |
| wavelet.LLH_gldm_LowGrayLevelEmphasis | 1,409151 | 0,237992 | 0,455961 | n.s. |
| wavelet.LLL_glszm_SmallAreaEmphasis | 1,414734 | 0,235909 | 0,455961 | n.s. |
| log.sigma.2.0.mm.3D_glcm_Contrast | 1,410261 | 0,237576 | 0,455961 | n.s. |
| log.sigma.2.0.mm.3D_glrlm_ShortRunHighGrayLevelEmphasis | 1,409989 | 0,237678 | 0,455961 | n.s. |
| log.sigma.3.0.mm.3D_glrlm_ShortRunHighGrayLevelEmphasis | 1,408805 | 0,238121 | 0,455961 | n.s. |
| log.sigma.5.0.mm.3D_firstorder_Variance | 1,416986 | 0,235073 | 0,455961 | n.s. |
| wavelet.HHL_glrlm_LongRunLowGrayLevelEmphasis | 1,407139 | 0,238747 | 0,456204 | n.s. |
| wavelet.HLL_glcm_Autocorrelation | 1,399581 | 0,241601 | 0,458129 | n.s. |
| wavelet.LLH_glrlm_LowGrayLevelRunEmphasis | 1,399416 | 0,241663 | 0,458129 | n.s. |
| wavelet.LHL_gldm_SmallDependenceHighGrayLevelEmphasis | 1,400187 | 0,241371 | 0,458129 | n.s. |
| log.sigma.5.0.mm.3D_firstorder_Range | 1,401198 | 0,240987 | 0,458129 | n.s. |
| log.sigma.5.0.mm.3D_gldm_LowGrayLevelEmphasis | 1,400587 | 0,241219 | 0,458129 | n.s. |
| log.sigma.4.0.mm.3D_glcm_Imc1 | 1,397644 | 0,242337 | 0,458628 | n.s. |
| log.sigma.5.0.mm.3D_gldm_HighGrayLevelEmphasis | 1,396715 | 0,242691 | 0,458628 | n.s. |
| wavelet.HLH_gldm_LargeDependenceLowGrayLevelEmphasis | 1,391962 | 0,244509 | 0,460613 | n.s. |
| wavelet.HHL_gldm_GrayLevelNonUniformity | 1,392745 | 0,244209 | 0,460613 | n.s. |
| wavelet.HHL_glszm_LowGrayLevelZoneEmphasis | 1,389175 | 0,24558 | 0,461645 | n.s. |
| log.sigma.5.0.mm.3D_glrlm_HighGrayLevelRunEmphasis | 1,388538 | 0,245826 | 0,461645 | n.s. |

| | | | | |
|---|---|---|---|---|
| wavelet.LLL_glcm_JointEntropy | 1,384264 | 0,247479 | 0,464023 | n.s. |
| wavelet.HHL_glszm_SmallAreaLowGrayLevelEmphasis | 1,374874 | 0,251146 | 0,465002 | n.s. |
| wavelet.LLH_firstorder_Minimum | 1,37873 | 0,249634 | 0,465002 | n.s. |
| wavelet.LLL_glcm_DifferenceVariance | 1,374572 | 0,251264 | 0,465002 | n.s. |
| log.sigma.1.0.mm.3D_firstorder_Uniformity | 1,374478 | 0,251301 | 0,465002 | n.s. |
| log.sigma.1.0.mm.3D_glcm_SumEntropy | 1,374438 | 0,251317 | 0,465002 | n.s. |
| log.sigma.1.0.mm.3D_glszm_SizeZoneNonUniformityNormalized | 1,37365 | 0,251627 | 0,465002 | n.s. |
| log.sigma.2.0.mm.3D_gldm_SmallDependenceHighGrayLevelEmphasis | 1,378866 | 0,249581 | 0,465002 | n.s. |
| log.sigma.4.0.mm.3D_glcm_Autocorrelation | 1,376268 | 0,250598 | 0,465002 | n.s. |
| log.sigma.5.0.mm.3D_glcm_Imc1 | 1,373017 | 0,251876 | 0,465002 | n.s. |
| log.sigma.5.0.mm.3D_glszm_GrayLevelVariance | 1,379211 | 0,249446 | 0,465002 | n.s. |
| wavelet.HHL_firstorder_RootMeanSquared | 1,370352 | 0,252928 | 0,465685 | n.s. |
| log.sigma.4.0.mm.3D_glrlm_ShortRunLowGrayLevelEmphasis | 1,370114 | 0,253022 | 0,465685 | n.s. |
| log.sigma.4.0.mm.3D_glrlm_LowGrayLevelRunEmphasis | 1,366013 | 0,25465 | 0,467963 | n.s. |
| wavelet.HLL_glcm_InverseVariance | 1,36289 | 0,255895 | 0,469532 | n.s. |
| wavelet.HLH_glszm_LowGrayLevelZoneEmphasis | 1,357525 | 0,258047 | 0,472037 | n.s. |
| wavelet.LLH_glcm_Idmn | 1,357763 | 0,257951 | 0,472037 | n.s. |
| log.sigma.5.0.mm.3D_gldm_LargeDependenceLowGrayLevelEmphasis | 1,351733 | 0,260388 | 0,475595 | n.s. |
| wavelet.LLH_glcm_Imc1 | 1,3501 | 0,261052 | 0,476082 | n.s. |
| wavelet.LHL_glcm_Imc1 | 1,348751 | 0,261601 | 0,47636 | n.s. |
| wavelet.HHH_glrlm_LongRunEmphasis | 1,347622 | 0,262061 | 0,476475 | n.s. |
| log.sigma.4.0.mm.3D_glcm_SumSquares | 1,344724 | 0,263247 | 0,477906 | n.s. |
| wavelet.HLH_glcm_Imc2 | 1,334936 | 0,267284 | 0,478005 | n.s. |
| wavelet.LHL_gldm_DependenceEntropy | 1,340151 | 0,265126 | 0,478005 | n.s. |
| wavelet.LLL_glcm_Contrast | 1,339317 | 0,26547 | 0,478005 | n.s. |
| log.sigma.1.0.mm.3D_glszm_SmallAreaHighGrayLevelEmphasis | 1,335004 | 0,267256 | 0,478005 | n.s. |
| log.sigma.2.0.mm.3D_gldm_SmallDependenceLowGrayLevelEmphasis | 1,338634 | 0,265752 | 0,478005 | n.s. |
| log.sigma.2.0.mm.3D_glszm_SizeZoneNonUniformity | 1,334997 | 0,267259 | 0,478005 | n.s. |
| log.sigma.3.0.mm.3D_glcm_Contrast | 1,337094 | 0,266389 | 0,478005 | n.s. |
| log.sigma.4.0.mm.3D_gldm_HighGrayLevelEmphasis | 1,337544 | 0,266203 | 0,478005 | n.s. |
| log.sigma.4.0.mm.3D_glrlm_HighGrayLevelRunEmphasis | 1,339597 | 0,265355 | 0,478005 | n.s. |
| log.sigma.4.0.mm.3D_glrlm_GrayLevelNonUniformityNormalized | 1,337894 | 0,266058 | 0,478005 | n.s. |
| log.sigma.1.0.mm.3D_firstorder_Median | 1,328542 | 0,269951 | 0,48134 | n.s. |
| log.sigma.4.0.mm.3D_glcm_ClusterTendency | 1,329116 | 0,269711 | 0,48134 | n.s. |
| wavelet.HHH_glszm_SizeZoneNonUniformity | 1,327176 | 0,270524 | 0,481645 | n.s. |
| wavelet.HLH_glszm_SizeZoneNonUniformity | 1,323785 | 0,27195 | 0,482039 | n.s. |
| log.sigma.2.0.mm.3D_glcm_SumEntropy | 1,324669 | 0,271578 | 0,482039 | n.s. |
| log.sigma.5.0.mm.3D_glcm_SumSquares | 1,325242 | 0,271336 | 0,482039 | n.s. |
| wavelet.HLL_gldm_DependenceVariance | 1,319236 | 0,273874 | 0,48333 | n.s. |
| wavelet.LLH_glcm_JointAverage | 1,315419 | 0,275498 | 0,48333 | n.s. |
| wavelet.LLH_glrlm_ShortRunLowGrayLevelEmphasis | 1,316302 | 0,275122 | 0,48333 | n.s. |
| wavelet.HHH_gldm_DependenceEntropy | 1,317834 | 0,27447 | 0,48333 | n.s. |
| log.sigma.2.0.mm.3D_glrlm_ShortRunLowGrayLevelEmphasis | 1,316034 | 0,275236 | 0,48333 | n.s. |
| log.sigma.2.0.mm.3D_glrlm_GrayLevelVariance | 1,31711 | 0,274778 | 0,48333 | n.s. |
| log.sigma.3.0.mm.3D_glcm_ClusterProminence | 1,320314 | 0,273417 | 0,48333 | n.s. |
| wavelet.HHL_firstorder_Mean | 1,31332 | 0,276394 | 0,483488 | n.s. |

| | | | | |
|---|---|---|---|---|
| log.sigma.4.0.mm.3D_gldm_LowGrayLevelEmphasis | 1,314164 | 0,276033 | 0,483488 | n.s. |
| original_glszm_GrayLevelVariance | 1,310017 | 0,27781 | 0,485258 | n.s. |
| log.sigma.2.0.mm.3D_gldm_DependenceEntropy | 1,308074 | 0,278645 | 0,486009 | n.s. |
| wavelet.LHL_firstorder_Mean | 1,300581 | 0,281889 | 0,490953 | n.s. |
| log.sigma.1.0.mm.3D_firstorder_Kurtosis | 1,296454 | 0,283689 | 0,492659 | n.s. |
| log.sigma.4.0.mm.3D_firstorder_Minimum | 1,297017 | 0,283443 | 0,492659 | n.s. |
| log.sigma.2.0.mm.3D_firstorder_Skewness | 1,295273 | 0,284206 | 0,492844 | n.s. |
| wavelet.HLL_glrlm_LongRunHighGrayLevelEmphasis | 1,290734 | 0,286201 | 0,495586 | n.s. |
| original_gldm_DependenceNonUniformityNormalized | 1,288938 | 0,286993 | 0,496212 | n.s. |
| wavelet.HHL_glszm_SmallAreaEmphasis | 1,284505 | 0,288958 | 0,496212 | n.s. |
| wavelet.LLH_glrlm_RunLengthNonUniformity | 1,284312 | 0,289044 | 0,496212 | n.s. |
| log.sigma.1.0.mm.3D_glrlm_GrayLevelNonUniformityNormalized | 1,286462 | 0,288089 | 0,496212 | n.s. |
| log.sigma.5.0.mm.3D_firstorder_Skewness | 1,285967 | 0,288309 | 0,496212 | n.s. |
| log.sigma.5.0.mm.3D_glcm_ClusterTendency | 1,28633 | 0,288148 | 0,496212 | n.s. |
| wavelet.LLH_gldm_DependenceNonUniformity | 1,283075 | 0,289594 | 0,496447 | n.s. |
| wavelet.HLL_firstorder_Mean | 1,278641 | 0,291574 | 0,497614 | n.s. |
| wavelet.LHH_glcm_ClusterShade | 1,279952 | 0,290987 | 0,497614 | n.s. |
| log.sigma.2.0.mm.3D_firstorder_Entropy | 1,277147 | 0,292244 | 0,497614 | n.s. |
| log.sigma.2.0.mm.3D_glcm_ClusterProminence | 1,276915 | 0,292348 | 0,497614 | n.s. |
| log.sigma.5.0.mm.3D_glcm_Imc2 | 1,277386 | 0,292137 | 0,497614 | n.s. |
| log.sigma.2.0.mm.3D_firstorder_Uniformity | 1,271128 | 0,294956 | 0,501342 | n.s. |
| wavelet.LLH_glszm_LargeAreaEmphasis | 1,266847 | 0,296898 | 0,503929 | n.s. |
| wavelet.LLH_glszm_ZoneVariance | 1,264636 | 0,297906 | 0,504925 | n.s. |
| wavelet.HHL_glszm_LargeAreaEmphasis | 1,262851 | 0,298721 | 0,505592 | n.s. |
| wavelet.HHL_glszm_ZoneVariance | 1,260394 | 0,299846 | 0,506782 | n.s. |
| log.sigma.3.0.mm.3D_glszm_GrayLevelNonUniformity | 1,256906 | 0,30145 | 0,508776 | n.s. |
| wavelet.LHL_glszm_LargeAreaEmphasis | 1,250106 | 0,304598 | 0,513367 | n.s. |
| wavelet.LHL_glszm_ZoneVariance | 1,248636 | 0,305282 | 0,513799 | n.s. |
| wavelet.HLH_glcm_InverseVariance | 1,245822 | 0,306595 | 0,515286 | n.s. |
| log.sigma.5.0.mm.3D_glrlm_GrayLevelVariance | 1,243302 | 0,307775 | 0,516546 | n.s. |
| wavelet.HHL_glszm_SizeZoneNonUniformityNormalized | 1,240691 | 0,309002 | 0,516813 | n.s. |
| log.sigma.2.0.mm.3D_glszm_SmallAreaLowGrayLevelEmphasis | 1,240494 | 0,309095 | 0,516813 | n.s. |
| log.sigma.4.0.mm.3D_glszm_ZonePercentage | 1,240214 | 0,309227 | 0,516813 | n.s. |
| wavelet.HLL_gldm_DependenceNonUniformityNormalized | 1,238955 | 0,30982 | 0,517085 | n.s. |
| wavelet.HHH_glszm_ZoneEntropy | 1,237466 | 0,310523 | 0,517539 | n.s. |
| log.sigma.5.0.mm.3D_firstorder_Uniformity | 1,235062 | 0,311661 | 0,518715 | n.s. |
| wavelet.HLL_glcm_Imc1 | 1,228198 | 0,314929 | 0,521262 | n.s. |
| wavelet.LLH_glcm_ClusterProminence | 1,220871 | 0,314921 | 0,521262 | n.s. |
| log.sigma.1.0.mm.3D_firstorder_RootMeanSquared | 1,230201 | 0,313973 | 0,521262 | n.s. |
| log.sigma.2.0.mm.3D_gldm_DependenceNonUniformityNormalized | 1,22965 | 0,314236 | 0,521262 | n.s. |
| wavelet.LLL_glszm_GrayLevelVariance | 1,226963 | 0,31552 | 0,521521 | n.s. |
| wavelet.HLL_firstorder_10Percentile | 1,223179 | 0,317337 | 0,523083 | n.s. |
| log.sigma.1.0.mm.3D_glrlm_RunLengthNonUniformity | 1,223767 | 0,317054 | 0,523083 | n.s. |
| wavelet.HHH_gldm_SmallDependenceHighGrayLevelEmphasis | 1,222205 | 0,317806 | 0,523138 | n.s. |
| original_gldm_DependenceNonUniformity | 1,216999 | 0,320324 | 0,525169 | n.s. |
| wavelet.HLH_glszm_SmallAreaLowGrayLevelEmphasis | 1,218405 | 0,319642 | 0,525169 | n.s. |

| | | | | |
|---|---|---|---|---|
| log.sigma.4.0.mm.3D_glcm_ClusterShade | 1,216037 | 0,320791 | 0,525169 | n.s. |
| log.sigma.4.0.mm.3D_glszm_SizeZoneNonUniformity | 1,216657 | 0,32049 | 0,525169 | n.s. |
| log.sigma.1.0.mm.3D_glcm_ClusterProminence | 1,214022 | 0,321771 | 0,526056 | n.s. |
| log.sigma.2.0.mm.3D_glrlm_LowGrayLevelRunEmphasis | 1,213053 | 0,322243 | 0,52611 | n.s. |
| wavelet.LLH_glrlm_GrayLevelNonUniformity | 1,209974 | 0,323747 | 0,527848 | n.s. |
| wavelet.LLL_gldm_SmallDependenceHighGrayLevelEmphasis | 1,208647 | 0,324397 | 0,52819 | n.s. |
| original_gldm_SmallDependenceHighGrayLevelEmphasis | 1,205772 | 0,325809 | 0,528535 | n.s. |
| wavelet.LLH_glcm_Correlation | 1,206994 | 0,325208 | 0,528535 | n.s. |
| log.sigma.2.0.mm.3D_glrlm_GrayLevelNonUniformityNormalized | 1,205526 | 0,32593 | 0,528535 | n.s. |
| wavelet.HLL_gldm_LargeDependenceHighGrayLevelEmphasis | 1,204052 | 0,326656 | 0,528998 | n.s. |
| wavelet.HHL_glszm_LargeAreaLowGrayLevelEmphasis | 1,202162 | 0,327589 | 0,529794 | n.s. |
| wavelet.HHH_gldm_DependenceVariance | 1,200115 | 0,328602 | 0,530004 | n.s. |
| log.sigma.4.0.mm.3D_gldm_SmallDependenceHighGrayLevelEmphasis | 1,200562 | 0,32838 | 0,530004 | n.s. |
| log.sigma.2.0.mm.3D_glrlm_HighGrayLevelRunEmphasis | 1,194704 | 0,331292 | 0,533625 | n.s. |
| wavelet.HLH_gldm_SmallDependenceLowGrayLevelEmphasis | 1,192801 | 0,332243 | 0,534439 | n.s. |
| log.sigma.5.0.mm.3D_glrlm_ShortRunLowGrayLevelEmphasis | 1,191305 | 0,332992 | 0,534927 | n.s. |
| original_firstorder_TotalEnergy | 1,139883 | 0,335965 | 0,538262 | n.s. |
| original_firstorder_Energy | 1,139883 | 0,335965 | 0,538262 | n.s. |
| wavelet.LLL_gldm_DependenceNonUniformity | 1,183986 | 0,336675 | 0,53868 | n.s. |
| wavelet.LHH_gldm_LargeDependenceLowGrayLevelEmphasis | 1,180335 | 0,338525 | 0,540919 | n.s. |
| wavelet.LLL_glcm_ClusterProminence | 1,131409 | 0,340855 | 0,543917 | n.s. |
| log.sigma.4.0.mm.3D_glszm_GrayLevelNonUniformityNormalized | 1,174409 | 0,341546 | 0,544296 | n.s. |
| original_glrlm_RunLengthNonUniformity | 1,173445 | 0,342039 | 0,544359 | n.s. |
| original_glrlm_ShortRunLowGrayLevelEmphasis | 1,167791 | 0,344945 | 0,548256 | n.s. |
| log.sigma.2.0.mm.3D_gldm_LowGrayLevelEmphasis | 1,165199 | 0,346283 | 0,549656 | n.s. |
| log.sigma.4.0.mm.3D_glszm_ZoneEntropy | 1,164314 | 0,346741 | 0,549656 | n.s. |
| original_glcm_SumSquares | 1,16252 | 0,347671 | 0,550403 | n.s. |
| wavelet.HLL_glcm_Correlation | 1,160292 | 0,34883 | 0,55118 | n.s. |
| wavelet.LHH_glrlm_LongRunLowGrayLevelEmphasis | 1,15857 | 0,349727 | 0,55118 | n.s. |
| log.sigma.1.0.mm.3D_firstorder_TotalEnergy | 1,115795 | 0,349999 | 0,55118 | n.s. |
| log.sigma.1.0.mm.3D_firstorder_Energy | 1,115795 | 0,349999 | 0,55118 | n.s. |
| original_glcm_ClusterTendency | 1,149796 | 0,354326 | 0,553239 | n.s. |
| wavelet.LLH_glcm_Idn | 1,148528 | 0,354995 | 0,553239 | n.s. |
| wavelet.LLL_firstorder_TotalEnergy | 1,107879 | 0,354702 | 0,553239 | n.s. |
| wavelet.LLL_firstorder_Energy | 1,107879 | 0,354702 | 0,553239 | n.s. |
| log.sigma.1.0.mm.3D_glcm_Idn | 1,153514 | 0,352371 | 0,553239 | n.s. |
| log.sigma.1.0.mm.3D_glszm_SmallAreaEmphasis | 1,15423 | 0,351996 | 0,553239 | n.s. |
| log.sigma.2.0.mm.3D_glcm_ClusterShade | 1,150334 | 0,354043 | 0,553239 | n.s. |
| log.sigma.5.0.mm.3D_gldm_SmallDependenceEmphasis | 1,152557 | 0,352874 | 0,553239 | n.s. |
| wavelet.HLH_glcm_Imc1 | 1,146312 | 0,356166 | 0,553626 | n.s. |
| wavelet.LHL_glcm_ClusterShade | 1,146879 | 0,355866 | 0,553626 | n.s. |
| original_glcm_ClusterProminence | 1,145019 | 0,356851 | 0,553671 | n.s. |
| wavelet.LLH_gldm_GrayLevelNonUniformity | 1,144516 | 0,357118 | 0,553671 | n.s. |
| wavelet.LLL_glcm_SumSquares | 1,138256 | 0,36045 | 0,555686 | n.s. |
| wavelet.LLL_glcm_ClusterTendency | 1,137727 | 0,360733 | 0,555686 | n.s. |
| log.sigma.1.0.mm.3D_firstorder_Mean | 1,140312 | 0,359353 | 0,555686 | n.s. |

| | | | | |
|---|---|---|---|---|
| log.sigma.1.0.mm.3D_glrlm_RunEntropy | 1,140145 | 0,359442 | 0,555686 | n.s. |
| log.sigma.3.0.mm.3D_glrlm_LongRunHighGrayLevelEmphasis | 1,138936 | 0,360087 | 0,555686 | n.s. |
| wavelet.HLH_glrlm_RunLengthNonUniformity | 1,130744 | 0,364482 | 0,560023 | n.s. |
| wavelet.LLL_glszm_SmallAreaHighGrayLevelEmphasis | 1,130779 | 0,364463 | 0,560023 | n.s. |
| wavelet.HLL_gldm_DependenceNonUniformity | 1,128012 | 0,365957 | 0,56157 | n.s. |
| wavelet.HHH_gldm_DependenceNonUniformity | 1,126809 | 0,366608 | 0,561851 | n.s. |
| wavelet.LHH_glrlm_LowGrayLevelRunEmphasis | 1,125889 | 0,367106 | 0,561897 | n.s. |
| wavelet.LHH_gldm_LowGrayLevelEmphasis | 1,121444 | 0,369522 | 0,562299 | n.s. |
| wavelet.LHH_glszm_LowGrayLevelZoneEmphasis | 1,123251 | 0,368539 | 0,562299 | n.s. |
| wavelet.LHH_glszm_LargeAreaLowGrayLevelEmphasis | 1,12273 | 0,368822 | 0,562299 | n.s. |
| wavelet.LHH_glszm_GrayLevelNonUniformity | 1,124402 | 0,367913 | 0,562299 | n.s. |
| wavelet.LHL_glcm_Idmn | 1,121097 | 0,369712 | 0,562299 | n.s. |
| wavelet.LLL_glcm_DifferenceAverage | 1,118077 | 0,371361 | 0,564093 | n.s. |
| wavelet.LHH_glrlm_ShortRunLowGrayLevelEmphasis | 1,114548 | 0,373296 | 0,565599 | n.s. |
| wavelet.HHH_gldm_DependenceNonUniformityNormalized | 1,114944 | 0,373078 | 0,565599 | n.s. |
| original_firstorder_Entropy | 1,110692 | 0,375419 | 0,567384 | n.s. |
| log.sigma.5.0.mm.3D_glcm_Correlation | 1,110694 | 0,375418 | 0,567384 | n.s. |
| wavelet.HHH_glszm_GrayLevelNonUniformityNormalized | 1,109201 | 0,376242 | 0,567913 | n.s. |
| wavelet.LLL_firstorder_InterquartileRange | 1,107277 | 0,377307 | 0,56803 | n.s. |
| wavelet.LLL_glrlm_RunLengthNonUniformity | 1,106518 | 0,377728 | 0,56803 | n.s. |
| log.sigma.2.0.mm.3D_glcm_MaximumProbability | 1,106496 | 0,37774 | 0,56803 | n.s. |
| original_firstorder_Variance | 1,080227 | 0,392525 | 0,568483 | n.s. |
| original_gldm_GrayLevelVariance | 1,087016 | 0,388662 | 0,568483 | n.s. |
| original_gldm_GrayLevelNonUniformity | 1,08382 | 0,390476 | 0,568483 | n.s. |
| original_glrlm_LowGrayLevelRunEmphasis | 1,079824 | 0,392755 | 0,568483 | n.s. |
| original_glrlm_GrayLevelVariance | 1,086736 | 0,38882 | 0,568483 | n.s. |
| original_glszm_ZoneEntropy | 1,100673 | 0,380979 | 0,568483 | n.s. |
| wavelet.LHH_glrlm_ShortRunHighGrayLevelEmphasis | 1,096593 | 0,383262 | 0,568483 | n.s. |
| wavelet.HLH_gldm_GrayLevelNonUniformity | 1,080458 | 0,392393 | 0,568483 | n.s. |
| wavelet.HHL_gldm_DependenceNonUniformityNormalized | 1,097295 | 0,382869 | 0,568483 | n.s. |
| wavelet.HHL_glszm_LargeAreaHighGrayLevelEmphasis | 1,049304 | 0,390881 | 0,568483 | n.s. |
| wavelet.LLH_glrlm_ShortRunHighGrayLevelEmphasis | 1,09659 | 0,383263 | 0,568483 | n.s. |
| wavelet.LHL_glcm_Idn | 1,09409 | 0,384667 | 0,568483 | n.s. |
| wavelet.LLL_firstorder_Variance | 1,09602 | 0,383583 | 0,568483 | n.s. |
| wavelet.LLL_gldm_HighGrayLevelEmphasis | 1,093737 | 0,384866 | 0,568483 | n.s. |
| wavelet.LLL_gldm_GrayLevelVariance | 1,098457 | 0,382218 | 0,568483 | n.s. |
| wavelet.LLL_gldm_GrayLevelNonUniformity | 1,085722 | 0,389396 | 0,568483 | n.s. |
| wavelet.LLL_glcm_Autocorrelation | 1,094279 | 0,384561 | 0,568483 | n.s. |
| wavelet.LLL_glcm_JointAverage | 1,088446 | 0,387852 | 0,568483 | n.s. |
| wavelet.LLL_glrlm_ShortRunHighGrayLevelEmphasis | 1,093893 | 0,384778 | 0,568483 | n.s. |
| wavelet.LLL_glrlm_GrayLevelVariance | 1,099332 | 0,381729 | 0,568483 | n.s. |
| wavelet.LLL_glrlm_HighGrayLevelRunEmphasis | 1,091698 | 0,386015 | 0,568483 | n.s. |
| wavelet.LLL_glrlm_LongRunHighGrayLevelEmphasis | 1,081897 | 0,391572 | 0,568483 | n.s. |
| wavelet.LLL_glrlm_GrayLevelNonUniformity | 1,079044 | 0,393201 | 0,568483 | n.s. |
| log.sigma.2.0.mm.3D_glrlm_LongRunLowGrayLevelEmphasis | 1,091123 | 0,386339 | 0,568483 | n.s. |
| log.sigma.2.0.mm.3D_glrlm_RunLengthNonUniformity | 1,101574 | 0,380476 | 0,568483 | n.s. |

| | | | | |
|---|---|---|---|---|
| log.sigma.2.0.mm.3D_glszm_SmallAreaEmphasis | 1,090753 | 0,386548 | 0,568483 | n.s. |
| log.sigma.3.0.mm.3D_gldm_SmallDependenceHighGrayLevelEmphasis | 1,083011 | 0,390937 | 0,568483 | n.s. |
| log.sigma.3.0.mm.3D_glszm_SmallAreaEmphasis | 1,092538 | 0,385541 | 0,568483 | n.s. |
| log.sigma.4.0.mm.3D_glcm_DifferenceEntropy | 1,089614 | 0,387191 | 0,568483 | n.s. |
| log.sigma.4.0.mm.3D_glcm_Imc2 | 1,080123 | 0,392584 | 0,568483 | n.s. |
| log.sigma.5.0.mm.3D_glcm_JointEnergy | 1,089934 | 0,38701 | 0,568483 | n.s. |
| log.sigma.5.0.mm.3D_glszm_SizeZoneNonUniformity | 1,092129 | 0,385772 | 0,568483 | n.s. |
| log.sigma.4.0.mm.3D_glszm_LargeAreaLowGrayLevelEmphasis | 1,044592 | 0,393896 | 0,568803 | n.s. |
| wavelet.LHL_glszm_GrayLevelNonUniformity | 1,074459 | 0,39583 | 0,570909 | n.s. |
| wavelet.LHH_firstorder_Range | 1,072277 | 0,397086 | 0,572032 | n.s. |
| original_firstorder_RobustMeanAbsoluteDeviation | 1,060057 | 0,404176 | 0,572445 | n.s. |
| original_gldm_HighGrayLevelEmphasis | 1,063138 | 0,402379 | 0,572445 | n.s. |
| original_glcm_Autocorrelation | 1,06295 | 0,402489 | 0,572445 | n.s. |
| original_glrlm_ShortRunHighGrayLevelEmphasis | 1,066755 | 0,400278 | 0,572445 | n.s. |
| original_glrlm_HighGrayLevelRunEmphasis | 1,059748 | 0,404356 | 0,572445 | n.s. |
| wavelet.HLH_glrlm_GrayLevelNonUniformity | 1,059454 | 0,404528 | 0,572445 | n.s. |
| wavelet.LLH_gldm_HighGrayLevelEmphasis | 1,061606 | 0,403272 | 0,572445 | n.s. |
| wavelet.LLH_glcm_Autocorrelation | 1,07007 | 0,39836 | 0,572445 | n.s. |
| wavelet.LLH_glrlm_HighGrayLevelRunEmphasis | 1,059627 | 0,404427 | 0,572445 | n.s. |
| wavelet.HHH_glrlm_RunVariance | 1,068987 | 0,398986 | 0,572445 | n.s. |
| wavelet.HHH_glszm_LargeAreaEmphasis | 1,034698 | 0,400278 | 0,572445 | n.s. |
| wavelet.HHH_glszm_ZoneVariance | 1,032521 | 0,401691 | 0,572445 | n.s. |
| log.sigma.3.0.mm.3D_glcm_Imc2 | 1,062965 | 0,40248 | 0,572445 | n.s. |
| log.sigma.4.0.mm.3D_glszm_SmallAreaEmphasis | 1,061782 | 0,403169 | 0,572445 | n.s. |
| log.sigma.5.0.mm.3D_glszm_ZoneEntropy | 1,060247 | 0,404064 | 0,572445 | n.s. |
| original_glrlm_GrayLevelNonUniformity | 1,057219 | 0,405835 | 0,573619 | n.s. |
| original_firstorder_InterquartileRange | 1,040084 | 0,415967 | 0,574241 | n.s. |
| original_gldm_LowGrayLevelEmphasis | 1,055407 | 0,406898 | 0,574241 | n.s. |
| original_gldm_LargeDependenceHighGrayLevelEmphasis | 1,034586 | 0,419257 | 0,574241 | n.s. |
| original_glcm_ClusterShade | 1,046733 | 0,409422 | 0,574241 | n.s. |
| original_glrlm_LongRunHighGrayLevelEmphasis | 1,045193 | 0,412927 | 0,574241 | n.s. |
| wavelet.HLL_glrlm_RunLengthNonUniformity | 1,040302 | 0,415837 | 0,574241 | n.s. |
| wavelet.HLL_glrlm_GrayLevelNonUniformity | 1,029557 | 0,422283 | 0,574241 | n.s. |
| wavelet.HLL_glszm_LargeAreaEmphasis | 1,031556 | 0,421079 | 0,574241 | n.s. |
| wavelet.HLL_glszm_ZoneVariance | 1,031366 | 0,421193 | 0,574241 | n.s. |
| wavelet.LHH_firstorder_Skewness | 1,041221 | 0,415289 | 0,574241 | n.s. |
| wavelet.LHH_gldm_DependenceNonUniformity | 1,032427 | 0,420554 | 0,574241 | n.s. |
| wavelet.LHH_glszm_LargeAreaHighGrayLevelEmphasis | 1,018755 | 0,410703 | 0,574241 | n.s. |
| wavelet.HHL_firstorder_Maximum | 1,047082 | 0,411807 | 0,574241 | n.s. |
| wavelet.LLH_glszm_LargeAreaHighGrayLevelEmphasis | 1,001101 | 0,42245 | 0,574241 | n.s. |
| wavelet.HHH_glcm_Correlation | 1,038482 | 0,416924 | 0,574241 | n.s. |
| wavelet.LLL_gldm_LargeDependenceHighGrayLevelEmphasis | 1,046289 | 0,412277 | 0,574241 | n.s. |
| wavelet.LLL_glcm_ClusterShade | 1,048533 | 0,408345 | 0,574241 | n.s. |
| wavelet.LLL_glszm_HighGrayLevelZoneEmphasis | 1,042803 | 0,414347 | 0,574241 | n.s. |
| log.sigma.1.0.mm.3D_gldm_GrayLevelNonUniformity | 1,032437 | 0,420548 | 0,574241 | n.s. |
| log.sigma.1.0.mm.3D_glrlm_ShortRunHighGrayLevelEmphasis | 1,045818 | 0,412556 | 0,574241 | n.s. |

| | | | | |
|---|---|---|---|---|
| log.sigma.2.0.mm.3D_gldm_HighGrayLevelEmphasis | 1,0363 | 0,418229 | 0,574241 | n.s. |
| log.sigma.2.0.mm.3D_gldm_DependenceNonUniformity | 1,050237 | 0,409941 | 0,574241 | n.s. |
| log.sigma.2.0.mm.3D_gldm_LargeDependenceLowGrayLevelEmphasis | 1,032772 | 0,420347 | 0,574241 | n.s. |
| log.sigma.2.0.mm.3D_glcm_SumSquares | 1,038502 | 0,416912 | 0,574241 | n.s. |
| log.sigma.2.0.mm.3D_glszm_LargeAreaHighGrayLevelEmphasis | 1,014052 | 0,413812 | 0,574241 | n.s. |
| log.sigma.3.0.mm.3D_firstorder_Variance | 1,037335 | 0,41761 | 0,574241 | n.s. |
| log.sigma.3.0.mm.3D_gldm_GrayLevelVariance | 1,032964 | 0,420231 | 0,574241 | n.s. |
| log.sigma.3.0.mm.3D_gldm_DependenceNonUniformityNormalized | 1,049269 | 0,410513 | 0,574241 | n.s. |
| log.sigma.4.0.mm.3D_gldm_LargeDependenceHighGrayLevelEmphasis | 1,043757 | 0,41378 | 0,574241 | n.s. |
| log.sigma.4.0.mm.3D_glcm_Idn | 1,029242 | 0,422473 | 0,574241 | n.s. |
| log.sigma.5.0.mm.3D_glcm_MaximumProbability | 1,038 | 0,417211 | 0,574241 | n.s. |
| log.sigma.5.0.mm.3D_glrlm_RunEntropy | 1,037938 | 0,417249 | 0,574241 | n.s. |
| log.sigma.5.0.mm.3D_glszm_LargeAreaHighGrayLevelEmphasis | 1,000959 | 0,422546 | 0,574241 | n.s. |
| log.sigma.5.0.mm.3D_glszm_GrayLevelNonUniformity | 1,039525 | 0,416301 | 0,574241 | n.s. |
| original_glcm_JointAverage | 1,028089 | 0,423169 | 0,574438 | n.s. |
| original_glszm_SmallAreaHighGrayLevelEmphasis | 1,022167 | 0,426759 | 0,575286 | n.s. |
| wavelet.HLL_glszm_LargeAreaHighGrayLevelEmphasis | 0,995906 | 0,425947 | 0,575286 | n.s. |
| wavelet.LHH_glszm_SizeZoneNonUniformityNormalized | 1,021673 | 0,42706 | 0,575286 | n.s. |
| wavelet.HLH_glszm_LargeAreaEmphasis | 0,99481 | 0,426687 | 0,575286 | n.s. |
| wavelet.HLH_glszm_ZoneVariance | 0,994912 | 0,426619 | 0,575286 | n.s. |
| log.sigma.1.0.mm.3D_glszm_LargeAreaHighGrayLevelEmphasis | 0,99607 | 0,425837 | 0,575286 | n.s. |
| log.sigma.2.0.mm.3D_glcm_ClusterTendency | 1,021525 | 0,42715 | 0,575286 | n.s. |
| original_glszm_GrayLevelNonUniformity | 1,012654 | 0,432571 | 0,576359 | n.s. |
| original_glszm_HighGrayLevelZoneEmphasis | 1,018388 | 0,429061 | 0,576359 | n.s. |
| wavelet.HLL_gldm_GrayLevelNonUniformity | 1,012062 | 0,432935 | 0,576359 | n.s. |
| wavelet.HLL_glszm_LargeAreaLowGrayLevelEmphasis | 1,010801 | 0,43371 | 0,576359 | n.s. |
| wavelet.LHL_glszm_SizeZoneNonUniformityNormalized | 1,015104 | 0,431069 | 0,576359 | n.s. |
| wavelet.LLL_glszm_LargeAreaEmphasis | 1,017365 | 0,429686 | 0,576359 | n.s. |
| wavelet.LLL_glszm_ZoneVariance | 1,017544 | 0,429577 | 0,576359 | n.s. |
| log.sigma.1.0.mm.3D_firstorder_Minimum | 1,011152 | 0,433494 | 0,576359 | n.s. |
| log.sigma.2.0.mm.3D_glrlm_RunEntropy | 1,013539 | 0,432028 | 0,576359 | n.s. |
| log.sigma.3.0.mm.3D_firstorder_MeanAbsoluteDeviation | 1,013896 | 0,431809 | 0,576359 | n.s. |
| log.sigma.3.0.mm.3D_glszm_LargeAreaHighGrayLevelEmphasis | 0,987549 | 0,43161 | 0,576359 | n.s. |
| log.sigma.5.0.mm.3D_glszm_ZonePercentage | 1,010863 | 0,433672 | 0,576359 | n.s. |
| wavelet.HHH_firstorder_Maximum | 1,007361 | 0,43583 | 0,578535 | n.s. |
| log.sigma.1.0.mm.3D_glrlm_HighGrayLevelRunEmphasis | 1,005974 | 0,436686 | 0,579032 | n.s. |
| wavelet.HHH_gldm_SmallDependenceLowGrayLevelEmphasis | 1,003083 | 0,438476 | 0,580395 | n.s. |
| log.sigma.1.0.mm.3D_gldm_HighGrayLevelEmphasis | 1,002752 | 0,438682 | 0,580395 | n.s. |
| log.sigma.4.0.mm.3D_glcm_ClusterProminence | 1,001159 | 0,43967 | 0,581062 | n.s. |
| wavelet.HLL_firstorder_Maximum | 0,991679 | 0,445586 | 0,582674 | n.s. |
| wavelet.HLH_firstorder_Minimum | 0,992971 | 0,444777 | 0,582674 | n.s. |
| wavelet.HLH_gldm_DependenceNonUniformity | 0,992665 | 0,444968 | 0,582674 | n.s. |
| wavelet.HLH_glszm_SmallAreaHighGrayLevelEmphasis | 0,991845 | 0,445482 | 0,582674 | n.s. |
| wavelet.HHH_glcm_ClusterShade | 0,993708 | 0,442063 | 0,582674 | n.s. |
| wavelet.LLL_firstorder_RobustMeanAbsoluteDeviation | 0,995278 | 0,443334 | 0,582674 | n.s. |
| log.sigma.1.0.mm.3D_glcm_Autocorrelation | 0,997803 | 0,441758 | 0,582674 | n.s. |

| | | | | |
|---|---|---|---|---|
| log.sigma.2.0.mm.3D_gldm_GrayLevelVariance | 0,993758 | 0,444284 | 0,582674 | n.s. |
| log.sigma.3.0.mm.3D_glcm_SumSquares | 0,991426 | 0,445745 | 0,582674 | n.s. |
| log.sigma.4.0.mm.3D_glszm_LargeAreaHighGrayLevelEmphasis | 0,97085 | 0,443065 | 0,582674 | n.s. |
| wavelet.LHL_glszm_LargeAreaHighGrayLevelEmphasis | 0,962454 | 0,448893 | 0,585662 | n.s. |
| wavelet.LLL_glszm_LargeAreaHighGrayLevelEmphasis | 0,962051 | 0,449174 | 0,585662 | n.s. |
| log.sigma.1.0.mm.3D_glszm_HighGrayLevelZoneEmphasis | 0,98541 | 0,449529 | 0,585662 | n.s. |
| log.sigma.3.0.mm.3D_glcm_ClusterTendency | 0,98469 | 0,449983 | 0,585662 | n.s. |
| wavelet.LHH_glszm_HighGrayLevelZoneEmphasis | 0,983394 | 0,450802 | 0,586092 | n.s. |
| original_glcm_SumEntropy | 0,974408 | 0,456507 | 0,587778 | n.s. |
| original_glszm_LargeAreaEmphasis | 0,975108 | 0,456061 | 0,587778 | n.s. |
| original_glszm_ZoneVariance | 0,976137 | 0,455406 | 0,587778 | n.s. |
| wavelet.HLH_glszm_LargeAreaHighGrayLevelEmphasis | 0,953672 | 0,455037 | 0,587778 | n.s. |
| wavelet.LLH_glcm_ClusterShade | 0,976051 | 0,45546 | 0,587778 | n.s. |
| wavelet.HHH_glcm_Idn | 0,979714 | 0,453133 | 0,587778 | n.s. |
| log.sigma.2.0.mm.3D_firstorder_TotalEnergy | 0,952095 | 0,456146 | 0,587778 | n.s. |
| log.sigma.2.0.mm.3D_firstorder_Energy | 0,952095 | 0,456146 | 0,587778 | n.s. |
| log.sigma.2.0.mm.3D_glcm_Autocorrelation | 0,978977 | 0,453601 | 0,587778 | n.s. |
| log.sigma.1.0.mm.3D_gldm_LargeDependenceLowGrayLevelEmphasis | 0,973637 | 0,456999 | 0,587781 | n.s. |
| wavelet.LHH_firstorder_Median | 0,964641 | 0,460706 | 0,590018 | n.s. |
| log.sigma.1.0.mm.3D_glrlm_LongRunHighGrayLevelEmphasis | 0,968648 | 0,46019 | 0,590018 | n.s. |
| log.sigma.2.0.mm.3D_glszm_LargeAreaEmphasis | 0,94576 | 0,460615 | 0,590018 | n.s. |
| log.sigma.2.0.mm.3D_glszm_ZoneVariance | 0,947363 | 0,459481 | 0,590018 | n.s. |
| log.sigma.5.0.mm.3D_glrlm_LongRunLowGrayLevelEmphasis | 0,961991 | 0,464472 | 0,594207 | n.s. |
| wavelet.LHH_glszm_SmallAreaLowGrayLevelEmphasis | 0,957615 | 0,467301 | 0,596555 | n.s. |
| wavelet.HHH_glcm_Idmn | 0,957946 | 0,467087 | 0,596555 | n.s. |
| log.sigma.1.0.mm.3D_glszm_GrayLevelNonUniformityNormalized | 0,955681 | 0,468555 | 0,59752 | n.s. |
| wavelet.LHH_gldm_SmallDependenceLowGrayLevelEmphasis | 0,948394 | 0,4733 | 0,597887 | n.s. |
| wavelet.HLH_glcm_JointAverage | 0,949233 | 0,472753 | 0,597887 | n.s. |
| wavelet.HLH_glszm_GrayLevelNonUniformity | 0,953986 | 0,469656 | 0,597887 | n.s. |
| wavelet.LLH_gldm_SmallDependenceLowGrayLevelEmphasis | 0,947103 | 0,474145 | 0,597887 | n.s. |
| wavelet.LLH_glrlm_LongRunHighGrayLevelEmphasis | 0,950665 | 0,471818 | 0,597887 | n.s. |
| wavelet.LLH_glszm_HighGrayLevelZoneEmphasis | 0,952017 | 0,470937 | 0,597887 | n.s. |
| wavelet.HHH_glrlm_ShortRunLowGrayLevelEmphasis | 0,947922 | 0,473609 | 0,597887 | n.s. |
| log.sigma.1.0.mm.3D_gldm_LargeDependenceHighGrayLevelEmphasis | 0,946829 | 0,474323 | 0,597887 | n.s. |
| log.sigma.1.0.mm.3D_glszm_ZoneVariance | 0,926673 | 0,47423 | 0,597887 | n.s. |
| log.sigma.2.0.mm.3D_firstorder_Variance | 0,952795 | 0,47043 | 0,597887 | n.s. |
| log.sigma.3.0.mm.3D_glcm_MaximumProbability | 0,952859 | 0,470389 | 0,597887 | n.s. |
| log.sigma.1.0.mm.3D_glszm_LargeAreaEmphasis | 0,925366 | 0,475171 | 0,598326 | n.s. |
| wavelet.HHH_gldm_LargeDependenceHighGrayLevelEmphasis | 0,942595 | 0,477099 | 0,600125 | n.s. |
| log.sigma.4.0.mm.3D_firstorder_Uniformity | 0,937459 | 0,48048 | 0,603745 | n.s. |
| original_glszm_LargeAreaHighGrayLevelEmphasis | 0,917164 | 0,481096 | 0,603887 | n.s. |
| wavelet.HHH_glrlm_LongRunHighGrayLevelEmphasis | 0,932128 | 0,484006 | 0,606904 | n.s. |
| wavelet.LHH_glszm_SmallAreaHighGrayLevelEmphasis | 0,924401 | 0,489147 | 0,609744 | n.s. |
| wavelet.LLH_glszm_SmallAreaHighGrayLevelEmphasis | 0,923808 | 0,489543 | 0,609744 | n.s. |
| wavelet.HHH_gldm_LowGrayLevelEmphasis | 0,92446 | 0,489107 | 0,609744 | n.s. |
| wavelet.HHH_glrlm_LowGrayLevelRunEmphasis | 0,926097 | 0,488016 | 0,609744 | n.s. |

| | | | | |
|---|---|---|---|---|
| wavelet.LHL_glszm_SmallAreaEmphasis | 0,925332 | 0,488526 | 0,609744 | n.s. |
| wavelet.LLL_glcm_Imc1 | 0,923381 | 0,489828 | 0,609744 | n.s. |
| log.sigma.2.0.mm.3D_glcm_Correlation | 0,926713 | 0,487605 | 0,609744 | n.s. |
| wavelet.HHL_gldm_DependenceNonUniformity | 0,921384 | 0,491163 | 0,610141 | n.s. |
| wavelet.LLH_gldm_LargeDependenceHighGrayLevelEmphasis | 0,921937 | 0,490793 | 0,610141 | n.s. |
| log.sigma.1.0.mm.3D_glrlm_LongRunLowGrayLevelEmphasis | 0,918339 | 0,493204 | 0,612042 | n.s. |
| wavelet.LLL_glcm_SumEntropy | 0,916239 | 0,494614 | 0,613158 | n.s. |
| log.sigma.3.0.mm.3D_glcm_Idmn | 0,915465 | 0,495134 | 0,61317 | n.s. |
| log.sigma.2.0.mm.3D_glcm_JointAverage | 0,914339 | 0,495892 | 0,613474 | n.s. |
| wavelet.HHH_glcm_InverseVariance | 0,907836 | 0,498615 | 0,616208 | n.s. |
| wavelet.HLH_glszm_HighGrayLevelZoneEmphasis | 0,908048 | 0,500139 | 0,616821 | n.s. |
| log.sigma.2.0.mm.3D_gldm_LargeDependenceHighGrayLevelEmphasis | 0,908645 | 0,499735 | 0,616821 | n.s. |
| log.sigma.5.0.mm.3D_gldm_LargeDependenceHighGrayLevelEmphasis | 0,906391 | 0,501262 | 0,617571 | n.s. |
| log.sigma.3.0.mm.3D_glszm_LowGrayLevelZoneEmphasis | 0,905623 | 0,501782 | 0,617578 | n.s. |
| wavelet.LLL_glszm_LargeAreaLowGrayLevelEmphasis | 0,904703 | 0,502406 | 0,617712 | n.s. |
| log.sigma.1.0.mm.3D_gldm_DependenceNonUniformity | 0,902151 | 0,50414 | 0,61921 | n.s. |
| wavelet.HLH_glszm_SmallAreaEmphasis | 0,899145 | 0,506187 | 0,621089 | n.s. |
| log.sigma.1.0.mm.3D_glcm_JointAverage | 0,896738 | 0,507831 | 0,622469 | n.s. |
| wavelet.LHH_firstorder_Maximum | 0,890098 | 0,512379 | 0,625835 | n.s. |
| wavelet.LHH_glcm_JointAverage | 0,889818 | 0,512572 | 0,625835 | n.s. |
| wavelet.HLH_gldm_DependenceNonUniformityNormalized | 0,891211 | 0,511615 | 0,625835 | n.s. |
| wavelet.LLL_firstorder_Entropy | 0,889685 | 0,512663 | 0,625835 | n.s. |
| wavelet.LLL_gldm_DependenceEntropy | 0,886642 | 0,514756 | 0,627487 | n.s. |
| log.sigma.4.0.mm.3D_gldm_LargeDependenceLowGrayLevelEmphasis | 0,886199 | 0,515062 | 0,627487 | n.s. |
| wavelet.LLL_glszm_SizeZoneNonUniformity | 0,882451 | 0,517648 | 0,629998 | n.s. |
| wavelet.LHL_firstorder_Kurtosis | 0,875826 | 0,522238 | 0,63494 | n.s. |
| wavelet.HLH_firstorder_Median | 0,871944 | 0,523523 | 0,635857 | n.s. |
| wavelet.LLL_glszm_SmallAreaLowGrayLevelEmphasis | 0,871017 | 0,525585 | 0,637717 | n.s. |
| wavelet.HLH_glszm_SizeZoneNonUniformityNormalized | 0,86957 | 0,526594 | 0,638153 | n.s. |
| log.sigma.3.0.mm.3D_gldm_DependenceNonUniformity | 0,868977 | 0,527008 | 0,638153 | n.s. |
| wavelet.HHH_gldm_LargeDependenceLowGrayLevelEmphasis | 0,866089 | 0,529027 | 0,639952 | n.s. |
| log.sigma.5.0.mm.3D_glszm_SmallAreaHighGrayLevelEmphasis | 0,861809 | 0,532027 | 0,642933 | n.s. |
| wavelet.LHH_glszm_SmallAreaEmphasis | 0,858856 | 0,534103 | 0,643497 | n.s. |
| log.sigma.5.0.mm.3D_glcm_DifferenceEntropy | 0,85958 | 0,533593 | 0,643497 | n.s. |
| log.sigma.5.0.mm.3D_glszm_LargeAreaLowGrayLevelEmphasis | 0,846666 | 0,533595 | 0,643497 | n.s. |
| wavelet.HHH_glcm_Autocorrelation | 0,856406 | 0,535828 | 0,643848 | n.s. |
| wavelet.HHH_glcm_JointAverage | 0,856875 | 0,535497 | 0,643848 | n.s. |
| wavelet.HHH_glrlm_LongRunLowGrayLevelEmphasis | 0,856156 | 0,536004 | 0,643848 | n.s. |
| original_gldm_DependenceEntropy | 0,855353 | 0,536571 | 0,643885 | n.s. |
| wavelet.HLH_glrlm_ShortRunHighGrayLevelEmphasis | 0,853915 | 0,537585 | 0,644457 | n.s. |
| wavelet.LLL_glszm_LowGrayLevelZoneEmphasis | 0,851833 | 0,539056 | 0,645576 | n.s. |
| wavelet.LLH_firstorder_Kurtosis | 0,850686 | 0,539868 | 0,645904 | n.s. |
| wavelet.HHH_gldm_HighGrayLevelEmphasis | 0,849026 | 0,541044 | 0,646022 | n.s. |
| log.sigma.4.0.mm.3D_glrlm_LongRunHighGrayLevelEmphasis | 0,849657 | 0,540596 | 0,646022 | n.s. |
| log.sigma.1.0.mm.3D_glcm_MaximumProbability | 0,846962 | 0,542507 | 0,646483 | n.s. |
| log.sigma.5.0.mm.3D_gldm_GrayLevelNonUniformity | 0,847253 | 0,5423 | 0,646483 | n.s. |

| | | | | |
|---|---|---|---|---|
| wavelet.HLL_glszm_GrayLevelNonUniformity | 0,841978 | 0,54605 | 0,649533 | n.s. |
| wavelet.LLL_gldm_SmallDependenceLowGrayLevelEmphasis | 0,841838 | 0,546149 | 0,649533 | n.s. |
| log.sigma.1.0.mm.3D_glrlm_GrayLevelNonUniformity | 0,838537 | 0,548503 | 0,651687 | n.s. |
| wavelet.LHH_glrlm_HighGrayLevelRunEmphasis | 0,837233 | 0,549434 | 0,652147 | n.s. |
| log.sigma.3.0.mm.3D_glszm_SizeZoneNonUniformity | 0,836072 | 0,550264 | 0,652487 | n.s. |
| wavelet.LHH_gldm_HighGrayLevelEmphasis | 0,831987 | 0,553189 | 0,65447 | n.s. |
| wavelet.HHH_glrlm_HighGrayLevelRunEmphasis | 0,831455 | 0,55357 | 0,65447 | n.s. |
| wavelet.LLL_gldm_DependenceNonUniformityNormalized | 0,831451 | 0,553573 | 0,65447 | n.s. |
| wavelet.HHH_firstorder_Median | 0,824923 | 0,557178 | 0,656792 | n.s. |
| log.sigma.1.0.mm.3D_glcm_Idmn | 0,826474 | 0,557149 | 0,656792 | n.s. |
| log.sigma.1.0.mm.3D_glcm_Imc2 | 0,827494 | 0,556415 | 0,656792 | n.s. |
| log.sigma.2.0.mm.3D_firstorder_10Percentile | 0,823667 | 0,55917 | 0,658493 | n.s. |
| wavelet.HHL_glcm_Imc2 | 0,819422 | 0,562235 | 0,660158 | n.s. |
| log.sigma.4.0.mm.3D_glszm_ZoneVariance | 0,810236 | 0,561707 | 0,660158 | n.s. |
| log.sigma.5.0.mm.3D_glcm_ClusterProminence | 0,820745 | 0,561279 | 0,660158 | n.s. |
| wavelet.HLH_glcm_Correlation | 0,81863 | 0,562807 | 0,660184 | n.s. |
| wavelet.HLL_glcm_Idn | 0,812871 | 0,56698 | 0,66443 | n.s. |
| wavelet.LHL_glcm_JointAverage | 0,809854 | 0,569172 | 0,66474 | n.s. |
| log.sigma.1.0.mm.3D_glcm_ClusterShade | 0,810352 | 0,56881 | 0,66474 | n.s. |
| log.sigma.1.0.mm.3D_glszm_LargeAreaLowGrayLevelEmphasis | 0,808734 | 0,569987 | 0,66474 | n.s. |
| log.sigma.1.0.mm.3D_glszm_ZoneEntropy | 0,808696 | 0,570014 | 0,66474 | n.s. |
| log.sigma.5.0.mm.3D_glszm_ZoneVariance | 0,800974 | 0,568946 | 0,66474 | n.s. |
| log.sigma.4.0.mm.3D_gldm_DependenceNonUniformity | 0,805559 | 0,572299 | 0,666757 | n.s. |
| original_glrlm_LongRunLowGrayLevelEmphasis | 0,792688 | 0,58172 | 0,67511 | n.s. |
| wavelet.HHH_glrlm_ShortRunHighGrayLevelEmphasis | 0,79451 | 0,580382 | 0,67511 | n.s. |
| log.sigma.3.0.mm.3D_glszm_ZoneVariance | 0,784761 | 0,581697 | 0,67511 | n.s. |
| log.sigma.4.0.mm.3D_glcm_MaximumProbability | 0,793449 | 0,581161 | 0,67511 | n.s. |
| wavelet.LHH_glcm_Correlation | 0,79183 | 0,58235 | 0,675189 | n.s. |
| wavelet.LHH_firstorder_Kurtosis | 0,790608 | 0,583249 | 0,675578 | n.s. |
| log.sigma.1.0.mm.3D_glcm_Correlation | 0,78703 | 0,585884 | 0,677453 | n.s. |
| log.sigma.5.0.mm.3D_gldm_DependenceNonUniformity | 0,786876 | 0,585997 | 0,677453 | n.s. |
| log.sigma.3.0.mm.3D_glrlm_HighGrayLevelRunEmphasis | 0,785932 | 0,586692 | 0,677604 | n.s. |
| log.sigma.4.0.mm.3D_glrlm_GrayLevelNonUniformity | 0,78491 | 0,587446 | 0,677823 | n.s. |
| log.sigma.4.0.mm.3D_glszm_LargeAreaEmphasis | 0,776173 | 0,58849 | 0,678375 | n.s. |
| wavelet.LHH_gldm_DependenceNonUniformityNormalized | 0,772042 | 0,596974 | 0,684865 | n.s. |
| wavelet.LHH_glcm_Autocorrelation | 0,772181 | 0,596871 | 0,684865 | n.s. |
| wavelet.LHL_firstorder_Minimum | 0,772975 | 0,596281 | 0,684865 | n.s. |
| log.sigma.4.0.mm.3D_glcm_JointEnergy | 0,774179 | 0,595387 | 0,684865 | n.s. |
| log.sigma.5.0.mm.3D_glszm_SizeZoneNonUniformityNormalized | 0,774022 | 0,595503 | 0,684865 | n.s. |
| log.sigma.1.0.mm.3D_firstorder_Skewness | 0,770073 | 0,598438 | 0,685023 | n.s. |
| log.sigma.1.0.mm.3D_gldm_DependenceEntropy | 0,769552 | 0,598825 | 0,685023 | n.s. |
| log.sigma.2.0.mm.3D_firstorder_Minimum | 0,77033 | 0,598247 | 0,685023 | n.s. |
| wavelet.LLH_glszm_LargeAreaLowGrayLevelEmphasis | 0,763903 | 0,603033 | 0,68918 | n.s. |
| log.sigma.3.0.mm.3D_glszm_LargeAreaEmphasis | 0,753295 | 0,606704 | 0,692716 | n.s. |
| log.sigma.4.0.mm.3D_glcm_Idmn | 0,754467 | 0,610087 | 0,695916 | n.s. |
| wavelet.LHL_firstorder_Median | 0,749356 | 0,613921 | 0,699625 | n.s. |

| | | | | |
|---|---|---|---|---|
| log.sigma.5.0.mm.3D_glszm_LargeAreaEmphasis | 0,742959 | 0,614981 | 0,700168 | n.s. |
| log.sigma.3.0.mm.3D_gldm_LowGrayLevelEmphasis | 0,740194 | 0,620814 | 0,706139 | n.s. |
| wavelet.HHH_firstorder_Kurtosis | 0,738842 | 0,621834 | 0,70663 | n.s. |
| wavelet.LHH_gldm_DependenceVariance | 0,735805 | 0,624127 | 0,707812 | n.s. |
| wavelet.HLH_glrlm_HighGrayLevelRunEmphasis | 0,73512 | 0,624644 | 0,707812 | n.s. |
| wavelet.HHH_firstorder_Range | 0,736622 | 0,623509 | 0,707812 | n.s. |
| log.sigma.3.0.mm.3D_glrlm_GrayLevelNonUniformity | 0,732999 | 0,626247 | 0,708959 | n.s. |
| wavelet.HLH_gldm_HighGrayLevelEmphasis | 0,729464 | 0,628922 | 0,711316 | n.s. |
| log.sigma.5.0.mm.3D_glszm_LowGrayLevelZoneEmphasis | 0,726281 | 0,631333 | 0,71337 | n.s. |
| log.sigma.3.0.mm.3D_gldm_LargeDependenceLowGrayLevelEmphasis | 0,723646 | 0,633331 | 0,714955 | n.s. |
| wavelet.HLH_glcm_Autocorrelation | 0,720938 | 0,635387 | 0,715929 | n.s. |
| log.sigma.1.0.mm.3D_glszm_SmallAreaLowGrayLevelEmphasis | 0,721001 | 0,635339 | 0,715929 | n.s. |
| original_glszm_LargeAreaLowGrayLevelEmphasis | 0,70924 | 0,644289 | 0,725278 | n.s. |
| log.sigma.3.0.mm.3D_glrlm_LowGrayLevelRunEmphasis | 0,700775 | 0,650751 | 0,731866 | n.s. |
| wavelet.HHH_firstorder_RootMeanSquared | 0,697708 | 0,65285 | 0,73354 | n.s. |
| wavelet.HHH_glcm_Imc1 | 0,693657 | 0,656197 | 0,73661 | n.s. |
| log.sigma.3.0.mm.3D_firstorder_RobustMeanAbsoluteDeviation | 0,690075 | 0,658941 | 0,738309 | n.s. |
| log.sigma.4.0.mm.3D_gldm_DependenceEntropy | 0,690621 | 0,658522 | 0,738309 | n.s. |
| wavelet.HHH_firstorder_Mean | 0,689176 | 0,65963 | 0,738392 | n.s. |
| log.sigma.2.0.mm.3D_firstorder_MeanAbsoluteDeviation | 0,687936 | 0,660581 | 0,738767 | n.s. |
| log.sigma.2.0.mm.3D_gldm_GrayLevelNonUniformity | 0,683283 | 0,664151 | 0,741377 | n.s. |
| log.sigma.4.0.mm.3D_glszm_SizeZoneNonUniformityNormalized | 0,68342 | 0,664046 | 0,741377 | n.s. |
| wavelet.LLL_glrlm_RunEntropy | 0,678682 | 0,667684 | 0,744629 | n.s. |
| log.sigma.4.0.mm.3D_glrlm_LongRunLowGrayLevelEmphasis | 0,677611 | 0,668508 | 0,744855 | n.s. |
| log.sigma.5.0.mm.3D_glcm_DifferenceVariance | 0,676287 | 0,669525 | 0,745297 | n.s. |
| wavelet.HHH_glszm_LargeAreaLowGrayLevelEmphasis | 0,672827 | 0,671674 | 0,746997 | n.s. |
| original_glrlm_RunEntropy | 0,668262 | 0,675698 | 0,750081 | n.s. |
| wavelet.LLL_glcm_Imc2 | 0,668908 | 0,675201 | 0,750081 | n.s. |
| wavelet.HLL_glcm_Idmn | 0,666993 | 0,676676 | 0,750472 | n.s. |
| log.sigma.4.0.mm.3D_gldm_GrayLevelNonUniformity | 0,663297 | 0,679522 | 0,752933 | n.s. |
| log.sigma.3.0.mm.3D_glrlm_RunLengthNonUniformity | 0,658325 | 0,683354 | 0,75648 | n.s. |
| log.sigma.5.0.mm.3D_glrlm_LongRunHighGrayLevelEmphasis | 0,653687 | 0,686929 | 0,759737 | n.s. |
| wavelet.HHH_glszm_SizeZoneNonUniformityNormalized | 0,648491 | 0,690937 | 0,762764 | n.s. |
| wavelet.LHL_glszm_LargeAreaLowGrayLevelEmphasis | 0,649108 | 0,690461 | 0,762764 | n.s. |
| wavelet.HHH_glcm_Imc2 | 0,646252 | 0,692665 | 0,763969 | n.s. |
| log.sigma.3.0.mm.3D_glcm_Idn | 0,641458 | 0,696366 | 0,767345 | n.s. |
| wavelet.HHH_glszm_SmallAreaEmphasis | 0,63988 | 0,697583 | 0,767981 | n.s. |
| log.sigma.4.0.mm.3D_glcm_DifferenceVariance | 0,6373 | 0,699575 | 0,769468 | n.s. |
| original_firstorder_Skewness | 0,636279 | 0,700363 | 0,76963 | n.s. |
| log.sigma.5.0.mm.3D_gldm_SmallDependenceLowGrayLevelEmphasis | 0,63527 | 0,701142 | 0,769781 | n.s. |
| wavelet.LHL_glrlm_ShortRunHighGrayLevelEmphasis | 0,633229 | 0,702718 | 0,770102 | n.s. |
| wavelet.LHL_glszm_SmallAreaHighGrayLevelEmphasis | 0,633284 | 0,702676 | 0,770102 | n.s. |
| log.sigma.4.0.mm.3D_glszm_LowGrayLevelZoneEmphasis | 0,63181 | 0,703813 | 0,770599 | n.s. |
| log.sigma.3.0.mm.3D_glrlm_ShortRunLowGrayLevelEmphasis | 0,629956 | 0,705245 | 0,771463 | n.s. |
| log.sigma.1.0.mm.3D_gldm_LowGrayLevelEmphasis | 0,625229 | 0,708895 | 0,774748 | n.s. |
| log.sigma.1.0.mm.3D_glrlm_LowGrayLevelRunEmphasis | 0,62108 | 0,712097 | 0,77754 | n.s. |

| | | | | |
|---|---|---|---|---|
| log.sigma.5.0.mm.3D_glrlm_LongRunEmphasis | 0,619682 | 0,713176 | 0,77801 | n.s. |
| wavelet.LHL_glszm_HighGrayLevelZoneEmphasis | 0,615224 | 0,716616 | 0,781053 | n.s. |
| log.sigma.4.0.mm.3D_firstorder_Skewness | 0,610469 | 0,720285 | 0,784339 | n.s. |
| wavelet.LLH_firstorder_Skewness | 0,608093 | 0,722117 | 0,784909 | n.s. |
| log.sigma.5.0.mm.3D_gldm_SmallDependenceHighGrayLevelEmphasis | 0,60837 | 0,721903 | 0,784909 | n.s. |
| wavelet.HHL_glcm_ClusterShade | 0,598394 | 0,729876 | 0,79114 | n.s. |
| wavelet.LHL_gldm_HighGrayLevelEmphasis | 0,598525 | 0,72949 | 0,79114 | n.s. |
| wavelet.LHL_glrlm_HighGrayLevelRunEmphasis | 0,599369 | 0,72884 | 0,79114 | n.s. |
| wavelet.LLL_glrlm_ShortRunLowGrayLevelEmphasis | 0,596375 | 0,731145 | 0,79114 | n.s. |
| log.sigma.5.0.mm.3D_glrlm_GrayLevelNonUniformity | 0,596827 | 0,730797 | 0,79114 | n.s. |
| original_gldm_LargeDependenceLowGrayLevelEmphasis | 0,593211 | 0,73358 | 0,792682 | n.s. |
| wavelet.HLL_firstorder_Median | 0,592806 | 0,733891 | 0,792682 | n.s. |
| original_firstorder_MeanAbsoluteDeviation | 0,588549 | 0,737165 | 0,795502 | n.s. |
| wavelet.LHL_glcm_Autocorrelation | 0,582983 | 0,741441 | 0,799397 | n.s. |
| log.sigma.3.0.mm.3D_firstorder_InterquartileRange | 0,580078 | 0,74367 | 0,801081 | n.s. |
| original_glcm_Correlation | 0,576977 | 0,746048 | 0,802203 | n.s. |
| wavelet.LLL_glrlm_LowGrayLevelRunEmphasis | 0,577331 | 0,745776 | 0,802203 | n.s. |
| wavelet.LHL_firstorder_Skewness | 0,571443 | 0,750287 | 0,806038 | n.s. |
| log.sigma.2.0.mm.3D_glcm_Imc2 | 0,568496 | 0,752541 | 0,807736 | n.s. |
| wavelet.LLL_glszm_ZoneEntropy | 0,566982 | 0,753698 | 0,808255 | n.s. |
| wavelet.LLL_gldm_LowGrayLevelEmphasis | 0,564967 | 0,755238 | 0,808527 | n.s. |
| log.sigma.1.0.mm.3D_glszm_LowGrayLevelZoneEmphasis | 0,564886 | 0,755299 | 0,808527 | n.s. |
| wavelet.HLH_glszm_LargeAreaLowGrayLevelEmphasis | 0,560949 | 0,758304 | 0,81102 | n.s. |
| log.sigma.1.0.mm.3D_firstorder_10Percentile | 0,559617 | 0,759319 | 0,811116 | n.s. |
| log.sigma.3.0.mm.3D_firstorder_Skewness | 0,559058 | 0,759745 | 0,811116 | n.s. |
| wavelet.LLL_firstorder_Skewness | 0,556661 | 0,761571 | 0,812343 | n.s. |
| original_glszm_GrayLevelNonUniformityNormalized | 0,553885 | 0,763684 | 0,813873 | n.s. |
| log.sigma.2.0.mm.3D_gldm_DependenceVariance | 0,543702 | 0,771411 | 0,821378 | n.s. |
| log.sigma.3.0.mm.3D_gldm_SmallDependenceLowGrayLevelEmphasis | 0,541389 | 0,773161 | 0,822512 | n.s. |
| wavelet.HLH_gldm_DependenceVariance | 0,5388 | 0,775118 | 0,823864 | n.s. |
| wavelet.LLL_glcm_Correlation | 0,536703 | 0,776701 | 0,824815 | n.s. |
| log.sigma.1.0.mm.3D_glrlm_ShortRunLowGrayLevelEmphasis | 0,534381 | 0,778453 | 0,825945 | n.s. |
| log.sigma.4.0.mm.3D_glrlm_LongRunEmphasis | 0,528746 | 0,782693 | 0,829709 | n.s. |
| original_glcm_Imc1 | 0,523508 | 0,786622 | 0,831726 | n.s. |
| log.sigma.3.0.mm.3D_gldm_HighGrayLevelEmphasis | 0,523036 | 0,786976 | 0,831726 | n.s. |
| log.sigma.4.0.mm.3D_gldm_DependenceVariance | 0,524308 | 0,786023 | 0,831726 | n.s. |
| log.sigma.5.0.mm.3D_gldm_DependenceEntropy | 0,522513 | 0,787367 | 0,831726 | n.s. |
| log.sigma.3.0.mm.3D_glrlm_LongRunLowGrayLevelEmphasis | 0,52115 | 0,788387 | 0,83207 | n.s. |
| log.sigma.2.0.mm.3D_glcm_Idmn | 0,519369 | 0,789718 | 0,832743 | n.s. |
| log.sigma.3.0.mm.3D_glcm_Autocorrelation | 0,51831 | 0,790509 | 0,832845 | n.s. |
| wavelet.HLL_firstorder_Skewness | 0,515468 | 0,792629 | 0,832885 | n.s. |
| wavelet.LHL_glrlm_LongRunHighGrayLevelEmphasis | 0,515935 | 0,792281 | 0,832885 | n.s. |
| log.sigma.3.0.mm.3D_glcm_ClusterShade | 0,516972 | 0,791508 | 0,832885 | n.s. |
| wavelet.HHH_firstorder_Skewness | 0,514622 | 0,793847 | 0,833435 | n.s. |
| original_glcm_Imc2 | 0,506281 | 0,799455 | 0,836654 | n.s. |
| wavelet.HLH_glrlm_LongRunHighGrayLevelEmphasis | 0,505946 | 0,799702 | 0,836654 | n.s. |

| | | | | |
|---|---|---|---|---|
| wavelet.LLH_glszm_SizeZoneNonUniformityNormalized | 0,505951 | 0,799699 | 0,836654 | n.s. |
| wavelet.LLL_glrlm_LongRunLowGrayLevelEmphasis | 0,508272 | 0,797979 | 0,836654 | n.s. |
| wavelet.LHL_gldm_LargeDependenceHighGrayLevelEmphasis | 0,502415 | 0,802313 | 0,837925 | n.s. |
| wavelet.LLL_glszm_GrayLevelNonUniformityNormalized | 0,503139 | 0,801778 | 0,837925 | n.s. |
| log.sigma.3.0.mm.3D_gldm_GrayLevelNonUniformity | 0,49918 | 0,804699 | 0,839686 | n.s. |
| wavelet.LLL_firstorder_MeanAbsoluteDeviation | 0,495728 | 0,807238 | 0,841604 | n.s. |
| wavelet.HLH_gldm_LargeDependenceHighGrayLevelEmphasis | 0,490911 | 0,81077 | 0,843996 | n.s. |
| log.sigma.3.0.mm.3D_gldm_DependenceVariance | 0,490679 | 0,81094 | 0,843996 | n.s. |
| log.sigma.4.0.mm.3D_glrlm_RunLengthNonUniformity | 0,483524 | 0,816158 | 0,848691 | n.s. |
| wavelet.HHH_glszm_GrayLevelVariance | 0,48018 | 0,818585 | 0,850478 | n.s. |
| wavelet.LHL_gldm_SmallDependenceLowGrayLevelEmphasis | 0,47824 | 0,81999 | 0,851201 | n.s. |
| log.sigma.1.0.mm.3D_gldm_SmallDependenceLowGrayLevelEmphasis | 0,475701 | 0,821825 | 0,852317 | n.s. |
| log.sigma.1.0.mm.3D_glcm_Imc1 | 0,474785 | 0,822486 | 0,852317 | n.s. |
| wavelet.LHH_gldm_LargeDependenceHighGrayLevelEmphasis | 0,464191 | 0,830085 | 0,8582 | n.s. |
| wavelet.LHL_glcm_Correlation | 0,465111 | 0,829428 | 0,8582 | n.s. |
| log.sigma.5.0.mm.3D_firstorder_Kurtosis | 0,463878 | 0,830308 | 0,8582 | n.s. |
| wavelet.LHH_glrlm_LongRunHighGrayLevelEmphasis | 0,461333 | 0,832121 | 0,859333 | n.s. |
| wavelet.HHL_glszm_SizeZoneNonUniformity | 0,454042 | 0,837286 | 0,863924 | n.s. |
| log.sigma.3.0.mm.3D_glcm_JointAverage | 0,446885 | 0,842314 | 0,868365 | n.s. |
| log.sigma.2.0.mm.3D_firstorder_RobustMeanAbsoluteDeviation | 0,44453 | 0,843959 | 0,86907 | n.s. |
| log.sigma.5.0.mm.3D_glrlm_RunVariance | 0,44383 | 0,844447 | 0,86907 | n.s. |
| log.sigma.1.0.mm.3D_gldm_DependenceNonUniformityNormalized | 0,429218 | 0,854534 | 0,878698 | n.s. |
| wavelet.HHH_glszm_SmallAreaLowGrayLevelEmphasis | 0,422791 | 0,858907 | 0,882439 | n.s. |
| log.sigma.2.0.mm.3D_glcm_Idn | 0,421491 | 0,859787 | 0,882587 | n.s. |
| log.sigma.1.0.mm.3D_gldm_DependenceVariance | 0,411166 | 0,866713 | 0,888936 | n.s. |
| wavelet.LLH_glszm_SmallAreaEmphasis | 0,393434 | 0,878341 | 0,900093 | n.s. |
| log.sigma.5.0.mm.3D_glrlm_RunLengthNonUniformity | 0,391236 | 0,879757 | 0,900775 | n.s. |
| log.sigma.2.0.mm.3D_firstorder_InterquartileRange | 0,388168 | 0,881725 | 0,90202 | n.s. |
| log.sigma.3.0.mm.3D_glszm_LargeAreaLowGrayLevelEmphasis | 0,392302 | 0,884456 | 0,904044 | n.s. |
| wavelet.LLL_gldm_LargeDependenceLowGrayLevelEmphasis | 0,378296 | 0,88798 | 0,906873 | n.s. |
| wavelet.HHH_firstorder_Minimum | 0,369621 | 0,893376 | 0,911608 | n.s. |
| log.sigma.4.0.mm.3D_glrlm_RunVariance | 0,365651 | 0,895812 | 0,913317 | n.s. |
| original_glszm_SizeZoneNonUniformityNormalized | 0,36332 | 0,89794 | 0,913782 | n.s. |
| wavelet.HHL_glcm_Imc1 | 0,35999 | 0,899251 | 0,913782 | n.s. |
| log.sigma.2.0.mm.3D_glszm_LargeAreaLowGrayLevelEmphasis | 0,362447 | 0,897764 | 0,913782 | n.s. |
| log.sigma.3.0.mm.3D_glszm_SizeZoneNonUniformityNormalized | 0,359886 | 0,899313 | 0,913782 | n.s. |
| log.sigma.5.0.mm.3D_gldm_DependenceNonUniformityNormalized | 0,355341 | 0,902042 | 0,915779 | n.s. |
| wavelet.HHH_glszm_LowGrayLevelZoneEmphasis | 0,34325 | 0,909159 | 0,922224 | n.s. |
| wavelet.HLL_glcm_ClusterShade | 0,341888 | 0,909948 | 0,922245 | n.s. |
| log.sigma.3.0.mm.3D_gldm_LargeDependenceHighGrayLevelEmphasis | 0,334646 | 0,914095 | 0,925666 | n.s. |
| wavelet.HLH_firstorder_TotalEnergy | 0,33521 | 0,91866 | 0,928721 | n.s. |
| wavelet.HLH_firstorder_Energy | 0,33521 | 0,91866 | 0,928721 | n.s. |
| original_glszm_SmallAreaEmphasis | 0,32488 | 0,920212 | 0,929507 | n.s. |
| log.sigma.4.0.mm.3D_gldm_DependenceNonUniformityNormalized | 0,31519 | 0,924838 | 0,933395 | n.s. |
| wavelet.HHH_glszm_HighGrayLevelZoneEmphasis | 0,295967 | 0,934851 | 0,942063 | n.s. |
| log.sigma.2.0.mm.3D_glrlm_GrayLevelNonUniformity | 0,295675 | 0,934998 | 0,942063 | n.s. |

| | | | | |
|---|---|---|---|---|
| wavelet.LHH_glszm_SizeZoneNonUniformity | 0,279115 | 0,943104 | 0,94929 | n.s. |
| log.sigma.5.0.mm.3D_glszm_SmallAreaEmphasis | 0,277746 | 0,943752 | 0,94929 | n.s. |
| log.sigma.2.0.mm.3D_glcm_Imc1 | 0,271687 | 0,94658 | 0,951337 | n.s. |
| wavelet.LHH_glcm_Idmn | 0,209114 | 0,971634 | 0,975699 | n.s. |
| wavelet.HHH_glszm_SmallAreaHighGrayLevelEmphasis | 0,198729 | 0,975023 | 0,978284 | n.s. |
| wavelet.LHH_glcm_Idn | 0,177779 | 0,981167 | 0,983626 | n.s. |
| log.sigma.4.0.mm.3D_glszm_GrayLevelNonUniformity | 0,138552 | 0,990167 | 0,99182 | n.s. |
| log.sigma.3.0.mm.3D_firstorder_Minimum | 0,120047 | 0,993299 | 0,994127 | n.s. |
| log.sigma.5.0.mm.3D_glszm_SmallAreaLowGrayLevelEmphasis | 0,114131 | 0,994154 | 0,994154 | n.s. |
