# Supplementary material for: Contrast Agent Dynamics Determine Radiomics Profiles in Oncologic Imaging
Source: Cancers (Basel). 2024 Apr 16;16(8):1519. doi: 10.3390/cancers16081519 (PMC11049400; doi:10.3390/cancers16081519)
Supplement: Supplementary file 1 [file cancers-16-01519-s001.zip › Table S4.pdf]

Table S4: Linear mixed model p values and cluster membership of all MRI radiomics features for pzPC tumors

Feature	F value	p value	FDR	Cluster
original_firstorder_90Percentile	69,47609	1,07E-21	5,99E-19	1
log-sigma-2-0-mm-3D_glcm_Idm	69,89825	9,21E-22	5,99E-19	3
log-sigma-2-0-mm-3D_glcm_Id	64,01842	5,76E-21	2,15E-18	3
log-sigma-2-0-mm-3D_firstorder_Entropy	63,02263	7,94E-21	2,22E-18	1
wavelet-LLH_glcm_Idm	61,81057	1,27E-20	2,37E-18	3
wavelet-LLL_firstorder_90Percentile	61,91743	1,18E-20	2,37E-18	1
original_firstorder_Mean	58,05434	4,44E-20	6,2E-18	1
original_firstorder_RootMeanSquared	58,42168	3,9E-20	6,2E-18	1
original_firstorder_Median	56,64438	7,33E-20	6,71E-18	1
log-sigma-2-0-mm-3D_glszm_ZoneEntropy	57,02776	6,48E-20	6,71E-18	1
wavelet-LLH_firstorder_Entropy	56,60559	7,8E-20	6,71E-18	1
wavelet-LLL_firstorder_Median	56,8411	6,84E-20	6,71E-18	1
wavelet-LLL_firstorder_RootMeanSquared	56,90395	6,68E-20	6,71E-18	1
original_glrlm_GrayLevelNonUniformityNormalized	56,1764	8,51E-20	6,8E-18	3
wavelet-LLL_firstorder_Mean	56,02629	9,18E-20	6,84E-18	1
wavelet-LLH_glcm_Id	55,69819	1,08E-19	7,55E-18	3
log-sigma-2-0-mm-3D_glrlm_GrayLevelNonUniformityNormalized	54,04089	1,8E-19	1,12E-17	3
wavelet-LLL_firstorder_Maximum	54,18471	1,81E-19	1,12E-17	1
log-sigma-2-0-mm-3D_glcm_DifferenceEntropy	53,27027	2,54E-19	1,49E-17	1
original_firstorder_Uniformity	52,34588	3,56E-19	1,99E-17	3
log-sigma-3-0-mm-3D_glcm_Idm	51,98838	4,05E-19	2,16E-17	3
log-sigma-3-0-mm-3D_glcm_Id	50,90694	6,2E-19	3,15E-17	3
original_firstorder_Maximum	49,0485	1,34E-18	6,44E-17	1
wavelet-LLH_glcm_DifferenceEntropy	49,09891	1,38E-18	6,44E-17	1
original_firstorder_Entropy	48,31295	1,81E-18	8,1E-17	1
log-sigma-3-0-mm-3D_firstorder_Entropy	48,02772	2,05E-18	8,81E-17	1
wavelet-LLH_glcm_JointEntropy	47,78395	2,32E-18	9,6E-17	1
log-sigma-2-0-mm-3D_glrlm_RunEntropy	47,54083	2,54E-18	1,01E-16	1
wavelet-LLH_glcm_SumEntropy	47,43525	2,69E-18	1,04E-16	1
log-sigma-3-0-mm-3D_glcm_DifferenceEntropy	47,07877	3,05E-18	1,07E-16	1
wavelet-LLL_glcm_Id	47,03958	3,03E-18	1,07E-16	3
wavelet-LLL_glrlm_RunEntropy	47,17956	2,96E-18	1,07E-16	1
wavelet-LLL_glszm_ZonePercentage	46,78524	3,48E-18	1,18E-16	1
wavelet-LLH_glrlm_RunLengthNonUniformityNormalized	45,43837	6,12E-18	2,01E-16	1
original_glcm_Idm	44,96776	7,45E-18	2,38E-16	3
wavelet-LLL_firstorder_Entropy	44,35401	9,94E-18	3,01E-16	1
wavelet-LLL_gldm_SmallDependenceEmphasis	44,35754	9,95E-18	3,01E-16	1
log-sigma-2-0-mm-3D_firstorder_Uniformity	43,72652	1,24E-17	3,64E-16	3
original_glcm_Id	43,11542	1,7E-17	4,76E-16	3
log-sigma-2-0-mm-3D_glrlm_RunLengthNonUniformityNormalized	43,19965	1,68E-17	4,76E-16	1
wavelet-LLL_glcm_Idm	43,02896	1,76E-17	4,81E-16	3
wavelet-LLH_glrlm_GrayLevelNonUniformityNormalized	43,09248	1,89E-17	5,04E-16	3
original_glrlm_RunEntropy	42,67574	2,13E-17	5,55E-16	1

wavelet-LLH_firstorder_Uniformity	41,90289	3,33E-17	8,45E-16	3
log-sigma-2-0-mm-3D_glcm_SumEntropy	40,89633	4,89E-17	1,19E-15	1
wavelet-LLL_firstorder_10Percentile	40,87485	4,92E-17	1,19E-15	1
wavelet-LLH_glrlm_RunPercentage	40,60266	5,56E-17	1,32E-15	1
log-sigma-2-0-mm-3D_glcm_Imc2	40,03437	7,39E-17	1,72E-15	1
log-sigma-3-0-mm-3D_glrlm_GrayLevelNonUniformityNormalized	39,89052	7,84E-17	1,79E-15	3
wavelet-LLL_glszm_GrayLevelNonUniformityNormalized	39,63107	8,92E-17	1,99E-15	3
log-sigma-3-0-mm-3D_glcm_SumEntropy	39,41408	1E-16	2,17E-15	1
wavelet-LLH_glrlm_RunEntropy	39,39225	1,02E-16	2,17E-15	1
wavelet-LLL_glrlm_GrayLevelNonUniformityNormalized	39,3082	1,03E-16	2,17E-15	3
log-sigma-3-0-mm-3D_glrlm_RunEntropy	38,95926	1,26E-16	2,61E-15	1
original_glcm_DifferenceEntropy	38,76168	1,37E-16	2,78E-15	1
log-sigma-3-0-mm-3D_glrlm_RunLengthNonUniformityNormalized	38,68686	1,41E-16	2,81E-15	1
original_firstorder_10Percentile	38,54152	1,53E-16	3E-15	1
wavelet-LLL_firstorder_Uniformity	38,43133	1,58E-16	3,05E-15	3
log-sigma-3-0-mm-3D_glcm_JointEntropy	38,31396	1,72E-16	3,26E-15	1
wavelet-LLL_glcm_InverseVariance	38,06806	1,89E-16	3,52E-15	3
wavelet-LLL_glcm_Imc1	37,66907	2,4E-16	4,4E-15	3
log-sigma-2-0-mm-3D_glcm_DifferenceAverage	37,51197	2,5E-16	4,51E-15	1
log-sigma-2-0-mm-3D_glcm_JointEntropy	37,30305	2,87E-16	5,09E-15	1
log-sigma-2-0-mm-3D_firstorder_Minimum	36,9195	3,43E-16	6E-15	3
log-sigma-2-0-mm-3D_firstorder_10Percentile	36,59708	3,94E-16	6,77E-15	3
wavelet-LLL_glszm_ZoneEntropy	36,50001	4,39E-16	7,43E-15	1
log-sigma-3-0-mm-3D_glrlm_ShortRunEmphasis	36,27658	4,79E-16	7,99E-15	1
log-sigma-2-0-mm-3D_firstorder_Range	35,74799	6,41E-16	1,04E-14	1
log-sigma-3-0-mm-3D_firstorder_Uniformity	35,74039	6,34E-16	1,04E-14	3
log-sigma-2-0-mm-3D_glrlm_RunPercentage	35,5998	6,99E-16	1,12E-14	1
log-sigma-2-0-mm-3D_firstorder_MeanAbsoluteDeviation	35,17859	8,48E-16	1,32E-14	1
wavelet-LLL_firstorder_Minimum	35,23962	8,46E-16	1,32E-14	1
wavelet-LLH_firstorder_MeanAbsoluteDeviation	35,00494	9,81E-16	1,5E-14	1
wavelet-LLH_firstorder_10Percentile	34,64779	1,11E-15	1,68E-14	3
wavelet-LLH_firstorder_RobustMeanAbsoluteDeviation	34,65701	1,18E-15	1,76E-14	1
log-sigma-2-0-mm-3D_glszm_GrayLevelNonUniformityNormalized	34,26341	1,38E-15	2,02E-14	3
log-sigma-3-0-mm-3D_firstorder_10Percentile	34,29313	1,39E-15	2,02E-14	3
wavelet-LLH_glrlm_ShortRunEmphasis	34,0727	1,57E-15	2,25E-14	1
wavelet-LLH_glszm_ZoneEntropy	34,06717	1,61E-15	2,28E-14	1
wavelet-LLH_glcm_DifferenceAverage	33,67042	2,03E-15	2,84E-14	1
original_glcm_InverseVariance	33,52593	2,14E-15	2,96E-14	3
log-sigma-2-0-mm-3D_firstorder_RobustMeanAbsoluteDeviation	33,24799	2,43E-15	3,27E-14	1
log-sigma-2-0-mm-3D_glrlm_ShortRunEmphasis	33,30867	2,43E-15	3,27E-14	1
log-sigma-3-0-mm-3D_glszm_ZoneEntropy	33,19895	2,61E-15	3,47E-14	1
original_firstorder_Minimum	32,99457	2,91E-15	3,83E-14	1
wavelet-LLH_firstorder_Minimum	32,76205	3,32E-15	4,32E-14	3
original_glrlm_RunLengthNonUniformityNormalized	32,73831	3,36E-15	4,32E-14	1
wavelet-LLL_firstorder_MeanAbsoluteDeviation	32,63001	3,52E-15	4,47E-14	1
log-sigma-3-0-mm-3D_firstorder_Minimum	32,32186	4,24E-15	5,32E-14	3
wavelet-LLL_glszm_SizeZoneNonUniformityNormalized	31,43018	7,07E-15	8,78E-14	1

log-sigma-3-0-mm-3D_glrlm_RunPercentage	31,33368	7,51E-15	9,23E-14	1
log-sigma-3-0-mm-3D_firstorder_MeanAbsoluteDeviation	31,09902	8,64E-15	1,05E-13	1
original_glrlm_ShortRunEmphasis	30,88291	9,87E-15	1,19E-13	1
log-sigma-3-0-mm-3D_glszm_LowGrayLevelZoneEmphasis	30,79777	1,03E-14	1,23E-13	3
original_glrlm_RunPercentage	30,78757	1,05E-14	1,23E-13	1
log-sigma-3-0-mm-3D_glcm_DifferenceAverage	30,7181	1,07E-14	1,24E-13	1
wavelet-LLL_glcm_DifferenceEntropy	30,60851	1,17E-14	1,35E-13	1
wavelet-LLL_firstorder_RobustMeanAbsoluteDeviation	30,47468	1,24E-14	1,41E-13	1
wavelet-LHL_firstorder_Entropy	30,09656	1,59E-14	1,79E-13	1
wavelet-LLL_glrlm_RunLengthNonUniformityNormalized	29,83484	1,86E-14	2,08E-13	1
log-sigma-3-0-mm-3D_firstorder_RobustMeanAbsoluteDeviation	29,7317	1,97E-14	2,18E-13	1
wavelet-HLL_glcm_Id	29,59979	2,12E-14	2,32E-13	3
wavelet-LLH_firstorder_InterquartileRange	29,16901	2,88E-14	3,12E-13	1
wavelet-LHL_glrlm_RunEntropy	29,07949	2,98E-14	3,21E-13	1
wavelet-LLL_gldm_DependenceNonUniformityNormalized	28,99244	3,14E-14	3,34E-13	1
log-sigma-2-0-mm-3D_glszm_ZonePercentage	28,96461	3,17E-14	3,35E-13	1
wavelet-LLL_firstorder_InterquartileRange	28,90698	3,26E-14	3,4E-13	1
wavelet-HLL_glcm_Idm	28,82827	3,42E-14	3,54E-13	3
wavelet-HLL_glrlm_GrayLevelNonUniformityNormalized	28,58111	4,03E-14	4,13E-13	3
wavelet-HLL_firstorder_Uniformity	28,55975	4,08E-14	4,15E-13	3
log-sigma-2-0-mm-3D_firstorder_InterquartileRange	28,47109	4,2E-14	4,22E-13	1
wavelet-LLL_firstorder_Range	28,50735	4,23E-14	4,22E-13	1
original_glszm_ZoneEntropy	28,34262	4,75E-14	4,69E-13	1
log-sigma-4-0-mm-3D_firstorder_10Percentile	28,25903	4,95E-14	4,85E-13	3
log-sigma-2-0-mm-3D_firstorder_Median	28,04312	5,32E-14	5,14E-13	3
log-sigma-3-0-mm-3D_glcm_Imc2	28,06742	5,34E-14	5,14E-13	1
wavelet-LLL_glrlm_ShortRunEmphasis	28,02913	5,75E-14	5,49E-13	1
log-sigma-3-0-mm-3D_firstorder_InterquartileRange	27,6709	7,31E-14	6,93E-13	1
wavelet-LLH_glcm_JointAverage	27,59769	7,69E-14	7,22E-13	1
wavelet-HLH_glrlm_GrayLevelNonUniformityNormalized	27,57101	7,92E-14	7,38E-13	3
log-sigma-3-0-mm-3D_glszm_GrayLevelNonUniformityNormalized	27,36454	9,04E-14	8,35E-13	3
log-sigma-4-0-mm-3D_glszm_ZoneEntropy	27,23336	9,68E-14	8,87E-13	1
log-sigma-2-0-mm-3D_firstorder_Mean	26,94551	1,09E-13	9,85E-13	3
log-sigma-3-0-mm-3D_firstorder_Mean	27,01682	1,09E-13	9,85E-13	3
log-sigma-3-0-mm-3D_firstorder_RootMeanSquared	26,95918	1,13E-13	1,01E-12	3
wavelet-LLH_firstorder_Range	26,97481	1,15E-13	1,02E-12	1
log-sigma-2-0-mm-3D_firstorder_RootMeanSquared	26,62422	1,35E-13	1,19E-12	3
wavelet-LLL_glszm_SmallAreaEmphasis	26,64322	1,38E-13	1,21E-12	1
wavelet-LLL_glrlm_RunPercentage	26,66673	1,4E-13	1,21E-12	1
wavelet-HLH_glrlm_RunEntropy	26,49251	1,59E-13	1,37E-12	1
log-sigma-4-0-mm-3D_firstorder_Minimum	26,32129	1,76E-13	1,5E-12	3
wavelet-LLL_glcm_JointAverage	26,11326	2E-13	1,69E-12	1
log-sigma-3-0-mm-3D_firstorder_Range	26,03529	2,14E-13	1,8E-12	1
wavelet-HLL_glcm_SumEntropy	26,02657	2,17E-13	1,81E-12	1
log-sigma-3-0-mm-3D_glcm_JointEnergy	25,94065	2,23E-13	1,84E-12	3
wavelet-HLL_firstorder_Entropy	25,97655	2,22E-13	1,84E-12	1
log-sigma-3-0-mm-3D_glcm_MaximumProbability	25,91708	2,25E-13	1,84E-12	3

original_glcm_SumEntropy	25,91118	2,34E-13	1,89E-12	1
original_glcm_Imc1	25,88756	2,38E-13	1,92E-12	3
original_firstorder_MeanAbsoluteDeviation	25,73952	2,58E-13	2,04E-12	1
log-sigma-3-0-mm-3D_firstorder_Median	25,73083	2,56E-13	2,04E-12	3
wavelet-LLL_glcm_DifferenceAverage	25,69276	2,6E-13	2,05E-12	1
wavelet-HLL_glrlm_RunLengthNonUniformityNormalized	25,4766	3,13E-13	2,45E-12	1
wavelet-HLH_firstorder_Entropy	25,44865	3,25E-13	2,53E-12	1
wavelet-LLH_glcm_Imc2	25,35283	3,34E-13	2,58E-12	1
wavelet-HLH_glcm_SumEntropy	25,28044	3,65E-13	2,79E-12	1
wavelet-HLL_glrlm_RunPercentage	25,13619	3,97E-13	3,02E-12	1
log-sigma-4-0-mm-3D_firstorder_RobustMeanAbsoluteDeviation	25,12203	3,99E-13	3,02E-12	1
log-sigma-5-0-mm-3D_firstorder_10Percentile	25,09387	4,08E-13	3,06E-12	3
original_glszm_GrayLevelNonUniformityNormalized	25,05771	4,14E-13	3,07E-12	3
log-sigma-4-0-mm-3D_glcm_Idm	25,02187	4,17E-13	3,07E-12	3
wavelet-HLH_glcm_JointEntropy	25,0844	4,17E-13	3,07E-12	1
original_glszm_ZonePercentage	24,70817	5,37E-13	3,92E-12	1
original_glcm_JointAverage	24,50444	6,08E-13	4,41E-12	1
log-sigma-4-0-mm-3D_glcm_Id	24,4279	6,3E-13	4,54E-12	3
log-sigma-4-0-mm-3D_firstorder_Entropy	24,15874	7,86E-13	5,63E-12	1
wavelet-LHL_glcm_InverseVariance	23,98779	8,91E-13	6,35E-12	3
log-sigma-5-0-mm-3D_firstorder_Minimum	23,95075	9,18E-13	6,5E-12	3
log-sigma-4-0-mm-3D_firstorder_RootMeanSquared	23,91589	9,32E-13	6,56E-12	3
log-sigma-4-0-mm-3D_firstorder_Mean	23,90178	9,42E-13	6,58E-12	3
log-sigma-2-0-mm-3D_gldm_DependenceNonUniformityNormalized	23,86109	9,89E-13	6,87E-12	1
log-sigma-4-0-mm-3D_glcm_JointEntropy	23,76778	1,05E-12	7,2E-12	1
wavelet-HLL_glrlm_ShortRunEmphasis	23,76137	1,05E-12	7,2E-12	1
log-sigma-4-0-mm-3D_firstorder_Median	23,5944	1,18E-12	8,03E-12	3
log-sigma-4-0-mm-3D_glcm_DifferenceEntropy	23,55447	1,22E-12	8,27E-12	1
log-sigma-4-0-mm-3D_firstorder_InterquartileRange	23,4992	1,27E-12	8,56E-12	1
wavelet-LHL_glcm_Id	23,31654	1,45E-12	9,73E-12	3
log-sigma-2-0-mm-3D_glcm_JointEnergy	23,15269	1,57E-12	1,05E-11	3
log-sigma-2-0-mm-3D_gldm_SmallDependenceEmphasis	23,08724	1,69E-12	1,11E-11	1
wavelet-LHL_glrlm_GrayLevelNonUniformityNormalized	23,06649	1,74E-12	1,15E-11	3
original_glcm_JointEnergy	22,99186	1,85E-12	1,21E-11	3
original_glcm_MaximumProbability	22,89141	1,99E-12	1,29E-11	3
log-sigma-4-0-mm-3D_firstorder_MeanAbsoluteDeviation	22,80746	2,12E-12	1,37E-11	1
original_firstorder_RobustMeanAbsoluteDeviation	22,71442	2,24E-12	1,44E-11	1
wavelet-HHL_firstorder_Entropy	22,64756	2,4E-12	1,53E-11	1
log-sigma-4-0-mm-3D_glcm_SumEntropy	22,61173	2,46E-12	1,56E-11	1
wavelet-HLH_firstorder_90Percentile	22,39955	2,9E-12	1,83E-11	1
wavelet-LLH_glszm_ZonePercentage	22,25512	3,19E-12	2E-11	1
wavelet-HLH_glszm_ZoneEntropy	22,26515	3,21E-12	2E-11	1
log-sigma-5-0-mm-3D_firstorder_RootMeanSquared	22,17768	3,39E-12	2,1E-11	3
wavelet-LHL_glszm_GrayLevelNonUniformityNormalized	22,18397	3,4E-12	2,1E-11	3
wavelet-LLH_glrlm_LongRunEmphasis	22,11954	3,53E-12	2,16E-11	3
wavelet-HLL_firstorder_10Percentile	22,10992	3,54E-12	2,16E-11	3
log-sigma-5-0-mm-3D_firstorder_Mean	22,09554	3,61E-12	2,19E-11	3

log-sigma-4-0-mm-3D_glrlm_RunLengthNonUniformityNormalized	21,87512	4,2E-12	2,54E-11	1
original_glcm_DifferenceAverage	21,71589	4,77E-12	2,87E-11	1
wavelet-LHL_glcm_Idm	21,71644	4,84E-12	2,9E-11	3
wavelet-HLL_glszm_ZonePercentage	21,68914	4,94E-12	2,94E-11	1
original_firstorder_Range	21,66284	5,03E-12	2,98E-11	1
wavelet-LLL_glcm_MaximumProbability	21,60235	5,32E-12	3,13E-11	3
log-sigma-5-0-mm-3D_firstorder_Median	21,43104	6,03E-12	3,53E-11	3
log-sigma-4-0-mm-3D_glcm_DifferenceAverage	21,38425	6,21E-12	3,62E-11	1
wavelet-HLL_glcm_DifferenceEntropy	21,38496	6,3E-12	3,65E-11	1
log-sigma-3-0-mm-3D_glcm_JointAverage	21,37013	6,36E-12	3,66E-11	1
log-sigma-3-0-mm-3D_glszm_ZonePercentage	21,35378	6,38E-12	3,66E-11	1
log-sigma-2-0-mm-3D_glcm_MaximumProbability	21,13024	7,29E-12	4,16E-11	3
wavelet-LHL_firstorder_Uniformity	21,05211	8,12E-12	4,61E-11	3
wavelet-HLH_glcm_DifferenceEntropy	20,99512	8,63E-12	4,88E-11	1
wavelet-LLL_glcm_JointEnergy	20,90151	9,25E-12	5,2E-11	3
log-sigma-2-0-mm-3D_glcm_JointAverage	20,73143	1,05E-11	5,87E-11	1
wavelet-LHL_glcm_DifferenceEntropy	20,66073	1,12E-11	6,23E-11	1
log-sigma-5-0-mm-3D_glcm_Idm	20,67223	1,14E-11	6,32E-11	3
wavelet-HLL_glrlm_LongRunEmphasis	20,60217	1,17E-11	6,42E-11	3
wavelet-LLL_glrlm_LongRunEmphasis	20,56474	1,2E-11	6,58E-11	3
wavelet-LLH_gldm_LargeDependenceEmphasis	20,54786	1,21E-11	6,58E-11	3
wavelet-LLL_gldm_DependenceEntropy	20,53146	1,24E-11	6,75E-11	1
wavelet-HLH_firstorder_MeanAbsoluteDeviation	20,49297	1,29E-11	6,94E-11	1
wavelet-HHL_firstorder_Uniformity	20,48001	1,29E-11	6,94E-11	3
original_glrlm_LongRunEmphasis	20,45575	1,31E-11	7,02E-11	3
log-sigma-2-0-mm-3D_glszm_LowGrayLevelZoneEmphasis	20,4097	1,33E-11	7,07E-11	3
wavelet-HLH_firstorder_RobustMeanAbsoluteDeviation	20,39991	1,39E-11	7,39E-11	1
wavelet-LHL_glszm_ZonePercentage	20,36946	1,41E-11	7,44E-11	1
wavelet-LHL_glcm_SumEntropy	20,34418	1,44E-11	7,58E-11	1
wavelet-HLH_firstorder_Uniformity	20,22161	1,63E-11	8,49E-11	3
log-sigma-5-0-mm-3D_glcm_Id	20,09902	1,82E-11	9,44E-11	3
log-sigma-2-0-mm-3D_glcm_Imc1	19,89699	2,08E-11	1,07E-10	3
wavelet-HLL_glrlm_RunEntropy	19,77881	2,28E-11	1,18E-10	1
log-sigma-3-0-mm-3D_gldm_SmallDependenceEmphasis	19,64281	2,51E-11	1,29E-10	1
wavelet-HHL_glcm_Id	19,47932	2,93E-11	1,5E-10	3
original_glcm_Imc2	19,35039	3,26E-11	1,66E-10	1
original_firstorder_InterquartileRange	19,25991	3,46E-11	1,75E-10	1
wavelet-LLL_glcm_SumEntropy	19,20372	3,7E-11	1,86E-10	1
wavelet-HHL_glrlm_GrayLevelNonUniformityNormalized	18,99703	4,39E-11	2,2E-10	3
log-sigma-4-0-mm-3D_glrlm_ShortRunEmphasis	18,95345	4,43E-11	2,21E-10	1
original_gldm_SmallDependenceEmphasis	18,93838	4,6E-11	2,29E-10	1
original_glcm_JointEntropy	18,76134	5,37E-11	2,66E-10	1
wavelet-LHL_firstorder_10Percentile	18,71027	5,56E-11	2,73E-10	3
wavelet-HHL_glcm_Idm	18,71759	5,57E-11	2,73E-10	3
wavelet-LLH_glcm_MaximumProbability	18,75829	5,8E-11	2,83E-10	3
wavelet-HLH_glcm_JointEnergy	18,69313	5,82E-11	2,83E-10	3
wavelet-HLH_glszm_ZonePercentage	18,65488	5,84E-11	2,83E-10	1

log-sigma-5-0-mm-3D_glcm_DifferenceEntropy	18,64795	5,92E-11	2,85E-10	1
log-sigma-4-0-mm-3D_glrlm_RunPercentage	18,56459	6,26E-11	3E-10	1
log-sigma-3-0-mm-3D_gldm_DependenceNonUniformityNormalized	18,55369	6,33E-11	3,02E-10	1
log-sigma-2-0-mm-3D_glrlm_LongRunLowGrayLevelEmphasis	18,50111	6,61E-11	3,13E-10	3
wavelet-LLH_glszm_GrayLevelNonUniformityNormalized	18,54364	6,6E-11	3,13E-10	3
log-sigma-5-0-mm-3D_glcm_DifferenceAverage	18,43276	7,19E-11	3,39E-10	1
wavelet-HHL_glrlm_RunEntropy	18,39838	7,35E-11	3,45E-10	1
wavelet-HLH_glrlm_ShortRunEmphasis	18,30649	7,9E-11	3,7E-10	1
wavelet-HLL_firstorder_RobustMeanAbsoluteDeviation	18,20602	8,53E-11	3,96E-10	1
wavelet-HLL_glcm_DifferenceAverage	18,20389	8,54E-11	3,96E-10	1
wavelet-HLL_firstorder_MeanAbsoluteDeviation	18,18043	8,74E-11	4,04E-10	1
log-sigma-5-0-mm-3D_firstorder_Entropy	18,12311	9,35E-11	4,3E-10	1
log-sigma-5-0-mm-3D_glcm_JointEntropy	18,09477	9,57E-11	4,39E-10	1
log-sigma-5-0-mm-3D_glrlm_ShortRunEmphasis	18,06292	9,63E-11	4,39E-10	1
wavelet-LLH_gldm_SmallDependenceEmphasis	18,05579	9,8E-11	4,45E-10	1
log-sigma-5-0-mm-3D_glrlm_RunLengthNonUniformityNormalized	17,98021	1,04E-10	4,71E-10	1
wavelet-HLL_glcm_Imc2	17,92684	1,11E-10	4,99E-10	1
wavelet-HLL_glrlm_RunVariance	17,91771	1,11E-10	5E-10	3
log-sigma-2-0-mm-3D_gldm_GrayLevelVariance	17,81128	1,2E-10	5,38E-10	1
log-sigma-4-0-mm-3D_firstorder_Range	17,81933	1,22E-10	5,41E-10	1
log-sigma-4-0-mm-3D_glrlm_RunEntropy	17,68146	1,38E-10	6,11E-10	1
wavelet-HHL_glcm_JointEntropy	17,66972	1,39E-10	6,16E-10	1
log-sigma-2-0-mm-3D_glrlm_GrayLevelVariance	17,62657	1,42E-10	6,25E-10	1
log-sigma-2-0-mm-3D_firstorder_Variance	17,54727	1,52E-10	6,68E-10	1
wavelet-LLH_glrlm_GrayLevelVariance	17,51117	1,61E-10	7,03E-10	1
wavelet-LHL_glrlm_RunPercentage	17,49545	1,62E-10	7,05E-10	1
wavelet-LLH_glrlm_RunVariance	17,46545	1,66E-10	7,19E-10	3
wavelet-HLL_glszm_ZoneEntropy	17,43151	1,72E-10	7,44E-10	1
log-sigma-2-0-mm-3D_glcm_SumSquares	17,39997	1,74E-10	7,47E-10	1
wavelet-LLH_firstorder_Variance	17,39912	1,78E-10	7,63E-10	1
wavelet-LLH_gldm_GrayLevelVariance	17,33853	1,88E-10	8,02E-10	1
wavelet-LHL_glrlm_RunLengthNonUniformityNormalized	17,27052	1,98E-10	8,43E-10	1
wavelet-LHL_glrlm_ShortRunEmphasis	17,21607	2,08E-10	8,81E-10	1
wavelet-LHH_glszm_ZoneEntropy	17,17676	2,17E-10	9,16E-10	1
wavelet-LLL_glrlm_LongRunLowGrayLevelEmphasis	17,07821	2,35E-10	9,88E-10	3
wavelet-LHL_glszm_ZoneEntropy	17,08034	2,37E-10	9,91E-10	1
log-sigma-4-0-mm-3D_glszm_LowGrayLevelZoneEmphasis	17,06499	2,38E-10	9,94E-10	3
wavelet-LHH_glrlm_RunEntropy	16,96171	2,64E-10	1,1E-09	1
log-sigma-2-0-mm-3D_glszm_GrayLevelVariance	16,92691	2,68E-10	1,11E-09	1
log-sigma-4-0-mm-3D_firstorder_Uniformity	16,88931	2,74E-10	1,13E-09	3
log-sigma-4-0-mm-3D_gldm_DependenceNonUniformityNormalized	16,90308	2,77E-10	1,14E-09	1
wavelet-HLL_firstorder_InterquartileRange	16,84861	2,87E-10	1,18E-09	1
wavelet-LLL_glrlm_RunVariance	16,85659	2,88E-10	1,18E-09	3
wavelet-HHL_glcm_SumEntropy	16,76885	3,14E-10	1,28E-09	1
wavelet-LLH_glszm_GrayLevelVariance	16,75136	3,19E-10	1,29E-09	1
wavelet-HLL_gldm_SmallDependenceEmphasis	16,72174	3,24E-10	1,31E-09	1
log-sigma-4-0-mm-3D_glszm_ZonePercentage	16,67394	3,36E-10	1,35E-09	1

wavelet-LLL_gldm_LowGrayLevelEmphasis	16,69512	3,35E-10	1,35E-09	3
wavelet-LHL_firstorder_Range	16,66609	3,45E-10	1,38E-09	1
log-sigma-4-0-mm-3D_glrlm_GrayLevelNonUniformityNormalized	16,63308	3,5E-10	1,39E-09	3
wavelet-LLL_glrlm_LowGrayLevelRunEmphasis	16,64826	3,5E-10	1,39E-09	3
log-sigma-5-0-mm-3D_firstorder_90Percentile	16,59219	3,62E-10	1,43E-09	3
wavelet-LLL_glrlm_ShortRunLowGrayLevelEmphasis	16,44507	4,23E-10	1,66E-09	3
wavelet-LHL_firstorder_Minimum	16,39019	4,45E-10	1,74E-09	3
log-sigma-5-0-mm-3D_glrlm_RunPercentage	16,33274	4,62E-10	1,8E-09	1
wavelet-HLL_glcm_JointEntropy	16,35172	4,63E-10	1,8E-09	1
wavelet-HLL_gldm_LargeDependenceEmphasis	16,32207	4,73E-10	1,84E-09	3
wavelet-HLH_firstorder_InterquartileRange	16,32216	4,78E-10	1,85E-09	1
log-sigma-4-0-mm-3D_glcm_JointAverage	16,24855	5,08E-10	1,96E-09	1
log-sigma-2-0-mm-3D_glrlm_LongRunEmphasis	16,22114	5,2E-10	2E-09	3
wavelet-HLH_glcm_Idm	16,10596	5,91E-10	2,26E-09	3
wavelet-HLH_glrlm_RunPercentage	16,06384	6,04E-10	2,3E-09	1
wavelet-LLH_glszm_LowGrayLevelZoneEmphasis	15,93547	6,84E-10	2,6E-09	3
log-sigma-2-0-mm-3D_glcm_Contrast	15,87952	7,05E-10	2,67E-09	1
wavelet-HLL_glcm_Imc1	15,88221	7,21E-10	2,72E-09	3
wavelet-LLH_glrlm_ShortRunHighGrayLevelEmphasis	15,85382	7,4E-10	2,79E-09	1
log-sigma-2-0-mm-3D_glcm_ClusterTendency	15,8055	7,64E-10	2,87E-09	1
original_glrlm_RunVariance	15,6844	8,67E-10	3,23E-09	3
wavelet-LHL_firstorder_Median	15,6795	8,65E-10	3,23E-09	3
wavelet-LHL_gldm_SmallDependenceEmphasis	15,64578	9E-10	3,34E-09	1
wavelet-HLH_glcm_Id	15,66048	9,04E-10	3,35E-09	3
log-sigma-5-0-mm-3D_glcm_SumEntropy	15,60818	9,37E-10	3,46E-09	1
wavelet-LHH_firstorder_Entropy	15,57106	9,73E-10	3,58E-09	1
wavelet-LLH_glcm_Contrast	15,55914	9,83E-10	3,6E-09	1
wavelet-LLH_gldm_HighGrayLevelEmphasis	15,52307	1,02E-09	3,71E-09	1
log-sigma-2-0-mm-3D_gldm_LargeDependenceEmphasis	15,49702	1,04E-09	3,78E-09	3
wavelet-HLH_glrlm_RunLengthNonUniformityNormalized	15,4879	1,05E-09	3,8E-09	1
wavelet-LLH_glrlm_HighGrayLevelRunEmphasis	15,42932	1,11E-09	4,03E-09	1
log-sigma-2-0-mm-3D_gldm_LargeDependenceLowGrayLevelEmphasis	14,11987	1,19E-09	4,28E-09	3
wavelet-HLH_gldm_SmallDependenceEmphasis	15,30624	1,25E-09	4,48E-09	1
log-sigma-3-0-mm-3D_glcm_SumSquares	15,26884	1,29E-09	4,61E-09	1
log-sigma-3-0-mm-3D_gldm_GrayLevelVariance	15,26796	1,29E-09	4,61E-09	1
wavelet-LHH_glrlm_GrayLevelNonUniformityNormalized	15,26896	1,31E-09	4,65E-09	3
wavelet-LHH_glcm_SumEntropy	15,18472	1,42E-09	5,03E-09	1
log-sigma-3-0-mm-3D_glrlm_GrayLevelVariance	15,16751	1,42E-09	5,04E-09	1
wavelet-HHL_glcm_DifferenceEntropy	15,14359	1,47E-09	5,19E-09	1
wavelet-LLH_gldm_DependenceNonUniformityNormalized	15,11792	1,5E-09	5,27E-09	1
wavelet-LHL_glrlm_LongRunEmphasis	15,12008	1,5E-09	5,27E-09	3
log-sigma-2-0-mm-3D_glcm_DifferenceVariance	15,0908	1,52E-09	5,31E-09	1
wavelet-HLL_glcm_InverseVariance	15,07275	1,55E-09	5,38E-09	3
wavelet-LLH_glcm_SumSquares	15,06978	1,58E-09	5,5E-09	1
log-sigma-3-0-mm-3D_firstorder_Variance	15,02266	1,64E-09	5,68E-09	1
log-sigma-3-0-mm-3D_glszm_GrayLevelVariance	15,01093	1,66E-09	5,72E-09	1
wavelet-LHL_glcm_Imc1	15,02013	1,66E-09	5,72E-09	3

wavelet-HHL_glrlm_RunPercentage	14,99585	1,7E-09	5,82E-09	1
wavelet-LLL_glszm_GrayLevelVariance	14,93802	1,78E-09	6,08E-09	1
log-sigma-3-0-mm-3D_glrlm_ShortRunLowGrayLevelEmphasis	14,9306	1,8E-09	6,15E-09	3
log-sigma-5-0-mm-3D_firstorder_MeanAbsoluteDeviation	14,92442	1,82E-09	6,19E-09	1
wavelet-HHL_glcm_MaximumProbability	14,9094	1,86E-09	6,3E-09	3
original_gldm_LargeDependenceEmphasis	14,79506	2,07E-09	6,98E-09	3
original_gldm_DependenceNonUniformityNormalized	14,78975	2,08E-09	7,02E-09	1
wavelet-HHL_glrlm_RunLengthNonUniformityNormalized	14,76727	2,13E-09	7,14E-09	1
log-sigma-3-0-mm-3D_glrlm_LowGrayLevelRunEmphasis	14,71105	2,24E-09	7,48E-09	3
wavelet-HLH_firstorder_Range	14,61613	2,49E-09	8,3E-09	1
wavelet-LLH_glcm_DifferenceVariance	14,51427	2,77E-09	9,22E-09	1
log-sigma-2-0-mm-3D_glszm_HighGrayLevelZoneEmphasis	14,48538	2,8E-09	9,29E-09	1
wavelet-LLH_glcm_Imc1	14,39496	3,08E-09	1,02E-08	3
log-sigma-3-0-mm-3D_glszm_HighGrayLevelZoneEmphasis	14,36578	3,17E-09	1,05E-08	1
wavelet-HLL_glszm_GrayLevelNonUniformityNormalized	14,35754	3,19E-09	1,05E-08	3
wavelet-HLH_glcm_Imc2	14,32857	3,29E-09	1,08E-08	1
log-sigma-5-0-mm-3D_firstorder_Range	14,27098	3,51E-09	1,15E-08	1
wavelet-HHL_glrlm_ShortRunEmphasis	14,26244	3,53E-09	1,15E-08	1
wavelet-HLL_glcm_JointEnergy	14,2007	3,78E-09	1,23E-08	3
log-sigma-2-0-mm-3D_glcm_InverseVariance	14,10585	3,99E-09	1,29E-08	3
log-sigma-3-0-mm-3D_glcm_Contrast	14,11367	4,07E-09	1,31E-08	1
wavelet-HLH_glcm_DifferenceAverage	14,09693	4,22E-09	1,36E-08	1
log-sigma-2-0-mm-3D_glrlm_LowGrayLevelRunEmphasis	14,08211	4,24E-09	1,36E-08	3
wavelet-LLL_glrlm_GrayLevelVariance	14,03842	4,4E-09	1,41E-08	1
wavelet-LLL_glszm_LowGrayLevelZoneEmphasis	14,01656	4,52E-09	1,44E-08	3
log-sigma-5-0-mm-3D_glszm_ZoneEntropy	14,00816	4,6E-09	1,46E-08	1
wavelet-LLL_firstorder_Variance	13,98467	4,66E-09	1,48E-08	1
wavelet-LLL_gldm_GrayLevelVariance	13,95214	4,81E-09	1,52E-08	1
log-sigma-4-0-mm-3D_glcm_MaximumProbability	13,91849	4,9E-09	1,55E-08	3
wavelet-LLH_glszm_HighGrayLevelZoneEmphasis	13,8736	5,28E-09	1,66E-08	1
log-sigma-3-0-mm-3D_glcm_ClusterTendency	13,84541	5,4E-09	1,7E-08	1
wavelet-LLH_glcm_ClusterTendency	13,82156	5,58E-09	1,75E-08	1
wavelet-LLH_glcm_JointEnergy	13,83085	5,89E-09	1,84E-08	3
wavelet-LLH_glcm_Autocorrelation	13,70889	6,26E-09	1,95E-08	1
wavelet-HHL_glcm_JointEnergy	13,70178	6,33E-09	1,97E-08	3
wavelet-LHH_glcm_JointEntropy	13,62216	6,88E-09	2,13E-08	1
log-sigma-5-0-mm-3D_firstorder_RobustMeanAbsoluteDeviation	13,53279	7,51E-09	2,32E-08	1
wavelet-HLH_glrlm_LongRunEmphasis	13,50464	7,74E-09	2,38E-08	3
log-sigma-5-0-mm-3D_gldm_DependenceNonUniformityNormalized	13,47224	7,92E-09	2,43E-08	1
wavelet-LHL_glcm_JointEntropy	13,39699	8,7E-09	2,66E-08	1
wavelet-HLL_firstorder_Minimum	13,3784	8,81E-09	2,69E-08	3
wavelet-HHL_glszm_ZoneEntropy	13,28141	9,83E-09	2,99E-08	1
wavelet-HLH_glcm_JointAverage	13,24961	1,02E-08	3,09E-08	1
wavelet-LLL_glcm_SumSquares	13,22069	1,04E-08	3,14E-08	1
wavelet-LHL_glcm_JointAverage	13,21205	1,05E-08	3,18E-08	1
wavelet-LHL_firstorder_MeanAbsoluteDeviation	13,12831	1,15E-08	3,47E-08	1
log-sigma-4-0-mm-3D_gldm_SmallDependenceEmphasis	13,10204	1,16E-08	3,49E-08	1

log-sigma-3-0-mm-3D_glrlm_ShortRunHighGrayLevelEmphasis	13,05476	1,24E-08	3,73E-08	1
wavelet-HLL_glcm_MaximumProbability	13,04326	1,26E-08	3,78E-08	3
log-sigma-2-0-mm-3D_glrlm_ShortRunHighGrayLevelEmphasis	12,96819	1,36E-08	4,06E-08	1
log-sigma-3-0-mm-3D_glrlm_HighGrayLevelRunEmphasis	12,93688	1,41E-08	4,2E-08	1
wavelet-HHL_glcm_DifferenceAverage	12,93189	1,43E-08	4,23E-08	1
wavelet-LHH_firstorder_Uniformity	12,92858	1,44E-08	4,25E-08	3
wavelet-HHL_glrlm_LongRunEmphasis	12,91713	1,45E-08	4,26E-08	3
wavelet-LHH_glcm_DifferenceEntropy	12,91578	1,46E-08	4,29E-08	1
wavelet-LLH_glrlm_LongRunHighGrayLevelEmphasis	12,90559	1,47E-08	4,31E-08	1
log-sigma-3-0-mm-3D_gldm_LargeDependenceEmphasis	12,87052	1,51E-08	4,42E-08	3
original_glrlm_GrayLevelVariance	12,7169	1,78E-08	5,19E-08	1
original_gldm_GrayLevelVariance	12,6995	1,81E-08	5,27E-08	1
original_glrlm_LongRunHighGrayLevelEmphasis	12,69154	1,83E-08	5,3E-08	1
log-sigma-4-0-mm-3D_glrlm_LowGrayLevelRunEmphasis	12,69885	1,83E-08	5,3E-08	3
wavelet-HHL_glcm_InverseVariance	12,69757	1,84E-08	5,3E-08	3
log-sigma-3-0-mm-3D_glcm_Idn	12,68791	1,85E-08	5,33E-08	3
log-sigma-2-0-mm-3D_gldm_LowGrayLevelEmphasis	12,66636	1,9E-08	5,44E-08	3
log-sigma-3-0-mm-3D_gldm_HighGrayLevelEmphasis	12,66514	1,9E-08	5,44E-08	1
log-sigma-4-0-mm-3D_firstorder_90Percentile	12,63482	1,93E-08	5,53E-08	3
log-sigma-2-0-mm-3D_glrlm_HighGrayLevelRunEmphasis	12,58765	2,06E-08	5,86E-08	1
log-sigma-5-0-mm-3D_glszm_LowGrayLevelZoneEmphasis	12,59384	2,06E-08	5,86E-08	3
original_glszm_GrayLevelVariance	12,56212	2,11E-08	5,98E-08	1
wavelet-LLH_gldm_DependenceVariance	12,52912	2,2E-08	6,21E-08	3
wavelet-HHL_glcm_Imc2	12,53177	2,2E-08	6,21E-08	1
wavelet-LHL_glrlm_RunVariance	12,52261	2,22E-08	6,25E-08	3
original_firstorder_Variance	12,47842	2,31E-08	6,48E-08	1
log-sigma-2-0-mm-3D_gldm_HighGrayLevelEmphasis	12,37625	2,6E-08	7,28E-08	1
wavelet-HLL_firstorder_Range	12,36475	2,64E-08	7,38E-08	1
wavelet-LLL_glcm_ClusterTendency	12,32149	2,76E-08	7,69E-08	1
original_glszm_LowGrayLevelZoneEmphasis	12,29823	2,81E-08	7,82E-08	3
wavelet-LLL_gldm_LargeDependenceEmphasis	12,24366	3,01E-08	8,36E-08	3
log-sigma-5-0-mm-3D_firstorder_InterquartileRange	12,21965	3,11E-08	8,59E-08	1
log-sigma-2-0-mm-3D_glrlm_ShortRunLowGrayLevelEmphasis	12,20813	3,14E-08	8,65E-08	3
log-sigma-3-0-mm-3D_gldm_LowGrayLevelEmphasis	12,20386	3,14E-08	8,65E-08	3
original_gldm_HighGrayLevelEmphasis	12,173	3,23E-08	8,88E-08	1
original_glrlm_HighGrayLevelRunEmphasis	12,17082	3,24E-08	8,89E-08	1
log-sigma-2-0-mm-3D_gldm_DependenceVariance	12,11653	3,49E-08	9,53E-08	3
log-sigma-5-0-mm-3D_glcm_JointAverage	12,10267	3,54E-08	9,66E-08	1
log-sigma-5-0-mm-3D_firstorder_Maximum	12,05695	3,63E-08	9,88E-08	3
wavelet-LHH_firstorder_MeanAbsoluteDeviation	12,06306	3,71E-08	1,01E-07	1
log-sigma-5-0-mm-3D_glrlm_GrayLevelNonUniformityNormalized	12,06173	3,73E-08	1,01E-07	3
wavelet-LHH_firstorder_Range	12,06012	3,72E-08	1,01E-07	1
log-sigma-5-0-mm-3D_glszm_ZonePercentage	12,0119	3,87E-08	1,04E-07	1
original_glrlm_ShortRunHighGrayLevelEmphasis	11,98762	3,98E-08	1,07E-07	1
wavelet-LLH_glszm_SmallAreaHighGrayLevelEmphasis	11,95522	4,18E-08	1,12E-07	1
log-sigma-4-0-mm-3D_glcm_JointEnergy	11,77045	5,04E-08	1,35E-07	3
log-sigma-5-0-mm-3D_firstorder_Uniformity	11,78418	5,11E-08	1,36E-07	3

wavelet-HLL_gldm_DependenceNonUniformityNormalized	11,70471	5,57E-08	1,48E-07	1
log-sigma-2-0-mm-3D_glrlm_RunVariance	11,69658	5,59E-08	1,48E-07	3
original_glcm_SumSquares	11,68612	5,6E-08	1,48E-07	1
wavelet-LLL_glcm_Contrast	11,67009	5,66E-08	1,49E-07	1
original_gldm_DependenceEntropy	11,57484	6,45E-08	1,7E-07	1
log-sigma-5-0-mm-3D_glrlm_RunEntropy	11,57173	6,47E-08	1,7E-07	1
log-sigma-4-0-mm-3D_glcm_Contrast	11,55216	6,56E-08	1,72E-07	1
wavelet-LHL_glcm_MaximumProbability	11,55722	6,56E-08	1,72E-07	3
log-sigma-3-0-mm-3D_glrlm_LongRunLowGrayLevelEmphasis	11,50518	6,84E-08	1,79E-07	3
wavelet-LHL_gldm_DependenceEntropy	11,51877	6,88E-08	1,79E-07	1
wavelet-HHL_gldm_LargeDependenceEmphasis	11,4742	7,21E-08	1,87E-07	3
wavelet-LHH_firstorder_10Percentile	11,46869	7,28E-08	1,89E-07	3
wavelet-HLH_glszm_LowGrayLevelZoneEmphasis	11,45536	7,47E-08	1,93E-07	3
original_glszm_HighGrayLevelZoneEmphasis	11,40709	7,74E-08	2E-07	1
log-sigma-3-0-mm-3D_glcm_DifferenceVariance	11,37621	8,02E-08	2,07E-07	1
wavelet-HHL_glrlm_RunVariance	11,34538	8,38E-08	2,15E-07	3
log-sigma-3-0-mm-3D_glcm_Imc1	11,33312	8,48E-08	2,17E-07	3
original_glcm_Autocorrelation	11,31227	8,61E-08	2,2E-07	1
original_glcm_ClusterTendency	11,30981	8,67E-08	2,21E-07	1
wavelet-HLH_glszm_HighGrayLevelZoneEmphasis	11,29806	8,88E-08	2,26E-07	1
wavelet-LHL_gldm_LargeDependenceEmphasis	11,27954	9,03E-08	2,3E-07	3
wavelet-HLH_firstorder_Minimum	11,25779	9,33E-08	2,37E-07	3
wavelet-LHL_glcm_JointEnergy	11,2398	9,49E-08	2,4E-07	3
wavelet-HLH_firstorder_Maximum	11,21063	9,82E-08	2,48E-07	1
original_gldm_DependenceVariance	11,14579	1,06E-07	2,66E-07	3
log-sigma-4-0-mm-3D_gldm_LowGrayLevelEmphasis	11,09878	1,12E-07	2,81E-07	3
wavelet-LHH_glcm_JointEnergy	11,09391	1,13E-07	2,84E-07	3
wavelet-LLH_firstorder_90Percentile	11,07087	1,17E-07	2,94E-07	1
wavelet-HLH_firstorder_10Percentile	10,99246	1,28E-07	3,19E-07	3
wavelet-LLH_gldm_SmallDependenceHighGrayLevelEmphasis	10,90913	1,4E-07	3,48E-07	1
wavelet-HLH_gldm_LargeDependenceEmphasis	10,9031	1,4E-07	3,49E-07	3
log-sigma-2-0-mm-3D_glszm_SmallAreaHighGrayLevelEmphasis	10,8263	1,53E-07	3,79E-07	1
wavelet-LLL_glrlm_LongRunHighGrayLevelEmphasis	10,7676	1,64E-07	4,06E-07	1
log-sigma-5-0-mm-3D_glcm_Contrast	10,74578	1,69E-07	4,16E-07	1
wavelet-LHH_firstorder_Maximum	10,75227	1,69E-07	4,16E-07	1
wavelet-LLL_glcm_DifferenceVariance	10,70473	1,75E-07	4,31E-07	1
wavelet-HLH_firstorder_Variance	10,71113	1,77E-07	4,34E-07	1
log-sigma-4-0-mm-3D_firstorder_Variance	10,69565	1,79E-07	4,39E-07	1
wavelet-LLL_glrlm_HighGrayLevelRunEmphasis	10,68953	1,8E-07	4,39E-07	1
log-sigma-3-0-mm-3D_glszm_SmallAreaHighGrayLevelEmphasis	10,68463	1,81E-07	4,41E-07	1
log-sigma-4-0-mm-3D_gldm_GrayLevelVariance	10,67817	1,83E-07	4,44E-07	1
wavelet-HLH_glcm_SumSquares	10,68241	1,83E-07	4,44E-07	1
wavelet-LLL_glszm_HighGrayLevelZoneEmphasis	10,66944	1,84E-07	4,46E-07	1
wavelet-LLL_gldm_HighGrayLevelEmphasis	10,66615	1,85E-07	4,47E-07	1
wavelet-LLH_firstorder_Maximum	10,67184	1,86E-07	4,47E-07	1
wavelet-LLL_glrlm_ShortRunHighGrayLevelEmphasis	10,66081	1,86E-07	4,47E-07	1
log-sigma-4-0-mm-3D_glszm_GrayLevelNonUniformityNormalized	10,6271	1,94E-07	4,64E-07	3

wavelet-HHH_firstorder_90Percentile	10,63518	1,94E-07	4,64E-07	1
log-sigma-4-0-mm-3D_glrlm_ShortRunLowGrayLevelEmphasis	10,61492	1,99E-07	4,74E-07	3
log-sigma-5-0-mm-3D_glcm_MaximumProbability	10,5902	2,08E-07	4,96E-07	3
wavelet-HLH_glcm_ClusterTendency	10,53004	2,2E-07	5,24E-07	1
log-sigma-3-0-mm-3D_glcm_Autocorrelation	10,47608	2,34E-07	5,55E-07	1
wavelet-HLH_glcm_Contrast	10,44306	2,44E-07	5,79E-07	1
log-sigma-4-0-mm-3D_glcm_Idn	10,39237	2,59E-07	6,11E-07	3
wavelet-HLL_glcm_Contrast	10,38549	2,59E-07	6,11E-07	1
log-sigma-4-0-mm-3D_glrlm_GrayLevelVariance	10,37169	2,65E-07	6,24E-07	1
wavelet-HHL_glcm_Imc1	10,36928	2,67E-07	6,28E-07	3
log-sigma-3-0-mm-3D_glrlm_LongRunHighGrayLevelEmphasis	10,35311	2,72E-07	6,36E-07	1
wavelet-LHH_firstorder_RobustMeanAbsoluteDeviation	10,3552	2,71E-07	6,36E-07	1
wavelet-HLH_glrlm_GrayLevelVariance	10,3514	2,73E-07	6,37E-07	1
log-sigma-4-0-mm-3D_glcm_SumSquares	10,34356	2,74E-07	6,39E-07	1
wavelet-LHH_glszm_ZonePercentage	10,32917	2,8E-07	6,51E-07	1
original_glrlm_LongRunLowGrayLevelEmphasis	10,30347	2,87E-07	6,66E-07	3
original_glcm_DifferenceVariance	10,29392	2,88E-07	6,68E-07	1
log-sigma-5-0-mm-3D_gldm_SmallDependenceEmphasis	10,29086	2,9E-07	6,7E-07	1
wavelet-HLH_glszm_GrayLevelVariance	10,2935	2,93E-07	6,76E-07	1
log-sigma-2-0-mm-3D_glrlm_LongRunHighGrayLevelEmphasis	10,2674	3,02E-07	6,94E-07	1
wavelet-LHH_glszm_GrayLevelNonUniformityNormalized	10,25629	3,08E-07	7,07E-07	3
wavelet-HHL_firstorder_MeanAbsoluteDeviation	10,25086	3,08E-07	7,07E-07	1
log-sigma-4-0-mm-3D_glcm_Imc2	10,20932	3,15E-07	7,2E-07	1
wavelet-HLH_glrlm_ShortRunHighGrayLevelEmphasis	10,21757	3,22E-07	7,34E-07	1
wavelet-LHL_glcm_DifferenceAverage	10,19878	3,28E-07	7,46E-07	1
log-sigma-3-0-mm-3D_gldm_DependenceVariance	10,15149	3,46E-07	7,87E-07	3
original_glcm_Contrast	10,13796	3,49E-07	7,92E-07	1
wavelet-HLL_gldm_DependenceVariance	10,05371	3,92E-07	8,88E-07	3
log-sigma-2-0-mm-3D_glszm_SmallAreaEmphasis	10,05309	3,97E-07	8,97E-07	1
original_glrlm_LowGrayLevelRunEmphasis	10,03885	3,99E-07	8,99E-07	3
wavelet-LLL_glcm_Autocorrelation	10,01731	4,07E-07	9,15E-07	1
wavelet-LHH_glrlm_ShortRunEmphasis	10,00633	4,16E-07	9,33E-07	1
wavelet-LLL_glszm_SmallAreaHighGrayLevelEmphasis	9,994766	4,18E-07	9,37E-07	1
wavelet-HHH_firstorder_MeanAbsoluteDeviation	9,982343	4,3E-07	9,6E-07	1
original_glszm_SmallAreaEmphasis	9,934929	4,47E-07	9,97E-07	1
log-sigma-5-0-mm-3D_glcm_Idn	9,909257	4,68E-07	1,04E-06	3
wavelet-HLH_gldm_GrayLevelVariance	9,877995	4,89E-07	1,09E-06	1
wavelet-HLH_glcm_DifferenceVariance	9,873763	4,91E-07	1,09E-06	1
wavelet-HLH_glrlm_HighGrayLevelRunEmphasis	9,867036	4,96E-07	1,1E-06	1
log-sigma-2-0-mm-3D_glcm_Autocorrelation	9,854211	5,01E-07	1,11E-06	1
original_glrlm_ShortRunLowGrayLevelEmphasis	9,846866	5,06E-07	1,12E-06	3
wavelet-LHL_firstorder_RobustMeanAbsoluteDeviation	9,72318	5,91E-07	1,3E-06	1
wavelet-LHH_firstorder_90Percentile	9,725334	5,92E-07	1,3E-06	1
log-sigma-3-0-mm-3D_gldm_LargeDependenceLowGrayLevelEmphasis	9,635602	6,47E-07	1,42E-06	3
wavelet-HLH_gldm_HighGrayLevelEmphasis	9,636806	6,61E-07	1,45E-06	1
original_gldm_LowGrayLevelEmphasis	9,610957	6,8E-07	1,49E-06	3
wavelet-HHL_glszm_GrayLevelNonUniformityNormalized	9,60968	6,84E-07	1,49E-06	3

wavelet-HLL_gldm_GrayLevelVariance	9,526368	7,53E-07	1,64E-06	1
log-sigma-4-0-mm-3D_gldm_LargeDependenceEmphasis	9,509489	7,71E-07	1,67E-06	3
wavelet-LHH_firstorder_InterquartileRange	9,514552	7,71E-07	1,67E-06	1
log-sigma-5-0-mm-3D_gldm_LargeDependenceEmphasis	9,478462	7,99E-07	1,73E-06	3
wavelet-LLH_firstorder_Mean	9,463769	8,27E-07	1,78E-06	3
wavelet-LLL_gldm_DependenceVariance	9,457116	8,27E-07	1,78E-06	3
wavelet-HLL_firstorder_Variance	9,435543	8,45E-07	1,82E-06	1
wavelet-HLL_glrlm_GrayLevelVariance	9,391676	8,94E-07	1,92E-06	1
log-sigma-2-0-mm-3D_gldm_SmallDependenceHighGrayLevelEmphasis	9,378245	9,06E-07	1,94E-06	1
wavelet-LLL_gldm_SmallDependenceHighGrayLevelEmphasis	9,370444	9,15E-07	1,96E-06	1
log-sigma-3-0-mm-3D_firstorder_90Percentile	9,345429	9,43E-07	2,01E-06	3
wavelet-HLL_glszm_LowGrayLevelZoneEmphasis	9,349632	9,5E-07	2,02E-06	3
log-sigma-4-0-mm-3D_glrlm_ShortRunHighGrayLevelEmphasis	9,319561	9,85E-07	2,08E-06	1
wavelet-HLL_glcm_JointAverage	9,321878	9,82E-07	2,08E-06	1
wavelet-HHL_firstorder_InterquartileRange	9,323286	9,83E-07	2,08E-06	1
log-sigma-3-0-mm-3D_glrlm_LongRunEmphasis	9,309237	9,95E-07	2,1E-06	3
log-sigma-3-0-mm-3D_gldm_SmallDependenceHighGrayLevelEmphasis	9,267344	1,05E-06	2,21E-06	1
log-sigma-5-0-mm-3D_glrlm_ShortRunHighGrayLevelEmphasis	9,269763	1,05E-06	2,21E-06	1
log-sigma-4-0-mm-3D_glrlm_HighGrayLevelRunEmphasis	9,262502	1,06E-06	2,23E-06	1
wavelet-HLH_glszm_GrayLevelNonUniformityNormalized	9,25971	1,08E-06	2,27E-06	3
log-sigma-5-0-mm-3D_glcm_JointEnergy	9,242714	1,1E-06	2,31E-06	3
log-sigma-4-0-mm-3D_gldm_HighGrayLevelEmphasis	9,224145	1,11E-06	2,33E-06	1
wavelet-HLH_glrlm_RunVariance	9,223537	1,12E-06	2,33E-06	3
log-sigma-4-0-mm-3D_glszm_GrayLevelVariance	9,219377	1,12E-06	2,33E-06	1
wavelet-LLL_gldm_LargeDependenceLowGrayLevelEmphasis	9,193639	1,13E-06	2,35E-06	3
wavelet-HLH_gldm_LargeDependenceLowGrayLevelEmphasis	9,214656	1,14E-06	2,36E-06	3
wavelet-LHH_glszm_GrayLevelVariance	9,204925	1,15E-06	2,37E-06	1
wavelet-LHL_firstorder_InterquartileRange	9,190121	1,16E-06	2,4E-06	1
log-sigma-4-0-mm-3D_glrlm_LongRunLowGrayLevelEmphasis	9,130547	1,23E-06	2,53E-06	3
wavelet-LHH_glrlm_LongRunEmphasis	9,107475	1,3E-06	2,67E-06	3
wavelet-LHH_glszm_LowGrayLevelZoneEmphasis	9,098403	1,32E-06	2,7E-06	3
wavelet-HHL_firstorder_RobustMeanAbsoluteDeviation	9,067238	1,37E-06	2,8E-06	1
wavelet-HLH_glcm_Autocorrelation	9,056863	1,39E-06	2,84E-06	1
wavelet-HLL_glcm_SumSquares	9,023359	1,44E-06	2,93E-06	1
wavelet-HLL_firstorder_Mean	9,016903	1,46E-06	2,97E-06	3
log-sigma-4-0-mm-3D_glcm_DifferenceVariance	9,00496	1,47E-06	3E-06	1
wavelet-LHH_glrlm_RunPercentage	8,944857	1,6E-06	3,25E-06	1
log-sigma-5-0-mm-3D_glrlm_HighGrayLevelRunEmphasis	8,937361	1,62E-06	3,28E-06	1
wavelet-HHL_gldm_DependenceVariance	8,927117	1,64E-06	3,31E-06	3
wavelet-LLL_glcm_JointEntropy	8,895615	1,71E-06	3,46E-06	1
log-sigma-4-0-mm-3D_glszm_HighGrayLevelZoneEmphasis	8,853981	1,8E-06	3,63E-06	1
original_glszm_SmallAreaHighGrayLevelEmphasis	8,842857	1,82E-06	3,66E-06	1
log-sigma-4-0-mm-3D_glcm_ClusterTendency	8,818921	1,88E-06	3,79E-06	1
log-sigma-5-0-mm-3D_gldm_HighGrayLevelEmphasis	8,790823	1,96E-06	3,93E-06	1
log-sigma-3-0-mm-3D_glszm_SmallAreaEmphasis	8,727022	2,11E-06	4,22E-06	1
wavelet-LHH_glcm_Idm	8,7115	2,18E-06	4,36E-06	3

log-sigma-5-0-mm-3D_glszm_GrayLevelVariance	8,706165	2,19E-06	4,37E-06	1
wavelet-LLH_firstorder_RootMeanSquared	8,712109	2,19E-06	4,37E-06	3
log-sigma-2-0-mm-3D_gldm_DependenceNonUniformity	8,66464	2,32E-06	4,61E-06	1
log-sigma-4-0-mm-3D_gldm_DependenceVariance	8,657398	2,33E-06	4,63E-06	3
wavelet-HLH_glrlm_LongRunLowGrayLevelEmphasis	8,649712	2,38E-06	4,72E-06	3
wavelet-LHH_glcm_Id	8,527826	2,78E-06	5,5E-06	3
wavelet-LHH_glrlm_RunLengthNonUniformityNormalized	8,446733	3,09E-06	6,1E-06	1
wavelet-HLH_glcm_MaximumProbability	8,398918	3,34E-06	6,58E-06	3
wavelet-LHL_gldm_DependenceNonUniformityNormalized	8,372096	3,42E-06	6,72E-06	1
log-sigma-4-0-mm-3D_glcm_Autocorrelation	8,342774	3,55E-06	6,98E-06	1
wavelet-LHL_glcm_Imc2	8,309067	3,72E-06	7,3E-06	1
wavelet-LLH_glszm_SizeZoneNonUniformity	8,291466	3,81E-06	7,46E-06	1
wavelet-LLH_firstorder_Median	8,275861	3,91E-06	7,65E-06	3
wavelet-LHL_firstorder_Mean	8,179574	4,42E-06	8,62E-06	3
log-sigma-5-0-mm-3D_glcm_Autocorrelation	8,086157	5,03E-06	9,8E-06	1
wavelet-HLL_glcm_ClusterTendency	8,067426	5,13E-06	9,98E-06	1
original_gldm_SmallDependenceHighGrayLevelEmphasis	8,038167	5,32E-06	1,03E-05	1
wavelet-HLL_glcm_DifferenceVariance	8,036616	5,36E-06	1,04E-05	1
wavelet-HLL_glszm_GrayLevelVariance	7,962577	5,93E-06	1,15E-05	1
wavelet-LLH_glcm_InverseVariance	7,562072	6,12E-06	1,18E-05	3
wavelet-LHH_gldm_SmallDependenceEmphasis	7,943077	6,13E-06	1,18E-05	1
wavelet-LHH_firstorder_Minimum	7,937326	6,18E-06	1,19E-05	3
wavelet-HHL_gldm_DependenceEntropy	7,922332	6,32E-06	1,21E-05	1
wavelet-LHH_glcm_JointAverage	7,862887	6,85E-06	1,31E-05	1
log-sigma-5-0-mm-3D_glszm_HighGrayLevelZoneEmphasis	7,845032	7,01E-06	1,34E-05	1
wavelet-LHH_glcm_DifferenceAverage	7,810857	7,36E-06	1,41E-05	1
original_glszm_SizeZoneNonUniformityNormalized	7,724558	8,27E-06	1,58E-05	1
wavelet-HHL_glszm_ZonePercentage	7,68426	8,77E-06	1,67E-05	1
wavelet-HLH_gldm_SmallDependenceHighGrayLevelEmphasis	7,637638	9,35E-06	1,78E-05	1
wavelet-LLL_gldm_DependenceNonUniformity	7,486189	1,16E-05	2,2E-05	1
wavelet-LLH_glcm_Correlation	7,47165	1,18E-05	2,23E-05	1
log-sigma-5-0-mm-3D_gldm_DependenceVariance	7,446915	1,22E-05	2,3E-05	3
wavelet-HLL_firstorder_RootMeanSquared	7,43692	1,24E-05	2,34E-05	3
wavelet-LHL_glszm_HighGrayLevelZoneEmphasis	7,406782	1,29E-05	2,44E-05	1
wavelet-HHL_firstorder_Range	7,392082	1,32E-05	2,49E-05	1
wavelet-HLH_glcm_Imc1	7,352135	1,4E-05	2,62E-05	3
wavelet-LHH_glcm_Imc2	7,320711	1,46E-05	2,74E-05	1
wavelet-LHL_glszm_SizeZoneNonUniformityNormalized	7,26455	1,58E-05	2,96E-05	1
log-sigma-5-0-mm-3D_glrlm_GrayLevelVariance	7,253395	1,61E-05	3,01E-05	1
log-sigma-4-0-mm-3D_gldm_LargeDependenceLowGrayLevelEmphasis	7,200247	1,7E-05	3,17E-05	3
wavelet-LHL_glcm_ClusterTendency	7,21353	1,7E-05	3,17E-05	1
log-sigma-5-0-mm-3D_firstorder_Variance	7,203195	1,73E-05	3,21E-05	1
log-sigma-5-0-mm-3D_gldm_GrayLevelVariance	7,183053	1,78E-05	3,3E-05	1
wavelet-LLH_glrlm_ShortRunLowGrayLevelEmphasis	7,142054	1,9E-05	3,52E-05	3
wavelet-LHH_glrlm_GrayLevelVariance	7,134948	1,91E-05	3,53E-05	1
log-sigma-5-0-mm-3D_glcm_DifferenceVariance	7,093685	2,02E-05	3,73E-05	1
wavelet-LHH_glcm_MaximumProbability	7,089382	2,04E-05	3,77E-05	3

log-sigma-5-0-mm-3D_glrlm_LongRunEmphasis	7,014627	2,26E-05	4,16E-05	3
wavelet-HLL_glcm_Correlation	6,984381	2,36E-05	4,34E-05	1
wavelet-LHL_glrlm_ShortRunHighGrayLevelEmphasis	6,981177	2,38E-05	4,36E-05	1
wavelet-LHL_glrlm_HighGrayLevelRunEmphasis	6,970968	2,41E-05	4,42E-05	1
wavelet-LHL_gldm_HighGrayLevelEmphasis	6,939399	2,52E-05	4,62E-05	1
wavelet-LLL_glszm_SizeZoneNonUniformity	6,899922	2,68E-05	4,89E-05	1
wavelet-LLH_gldm_DependenceNonUniformity	6,88793	2,73E-05	4,98E-05	1
wavelet-HHH_firstorder_10Percentile	6,883206	2,75E-05	5E-05	3
log-sigma-4-0-mm-3D_glrlm_LongRunHighGrayLevelEmphasis	6,867856	2,81E-05	5,1E-05	1
wavelet-LHL_glszm_SmallAreaEmphasis	6,863419	2,82E-05	5,12E-05	1
log-sigma-4-0-mm-3D_glrlm_GrayLevelNonUniformity	6,851113	2,88E-05	5,22E-05	3
wavelet-LHL_glrlm_LongRunHighGrayLevelEmphasis	6,823333	2,99E-05	5,41E-05	1
wavelet-HHH_firstorder_RobustMeanAbsoluteDeviation	6,813165	3,04E-05	5,5E-05	1
wavelet-LHH_glcm_ClusterTendency	6,788371	3,15E-05	5,68E-05	1
wavelet-HLH_glrlm_LongRunHighGrayLevelEmphasis	6,78144	3,19E-05	5,74E-05	1
wavelet-LHL_glszm_SmallAreaHighGrayLevelEmphasis	6,770197	3,23E-05	5,81E-05	1
wavelet-LHH_glszm_HighGrayLevelZoneEmphasis	6,762421	3,28E-05	5,88E-05	1
wavelet-LLL_glszm_SmallAreaLowGrayLevelEmphasis	6,746375	3,34E-05	5,98E-05	3
original_glszm_SizeZoneNonUniformity	6,742895	3,37E-05	6,02E-05	1
log-sigma-2-0-mm-3D_glszm_SizeZoneNonUniformity	6,725901	3,45E-05	6,16E-05	1
wavelet-LLH_glcm_ClusterProminence	6,722312	3,46E-05	6,18E-05	1
log-sigma-4-0-mm-3D_gldm_DependenceEntropy	6,717154	3,5E-05	6,24E-05	1
wavelet-LHL_glszm_GrayLevelVariance	6,708909	3,54E-05	6,29E-05	1
log-sigma-5-0-mm-3D_glcm_SumSquares	6,705924	3,55E-05	6,3E-05	1
log-sigma-4-0-mm-3D_glszm_SmallAreaHighGrayLevelEmphasis	6,687693	3,65E-05	6,46E-05	1
original_gldm_DependenceNonUniformity	6,685198	3,67E-05	6,49E-05	1
wavelet-LHH_glszm_SmallAreaHighGrayLevelEmphasis	6,679613	3,7E-05	6,53E-05	1
log-sigma-4-0-mm-3D_glszm_SmallAreaEmphasis	6,653318	3,83E-05	6,75E-05	1
log-sigma-5-0-mm-3D_glrlm_GrayLevelNonUniformity	6,630119	3,98E-05	7,01E-05	3
wavelet-HHL_firstorder_10Percentile	6,615734	4,06E-05	7,14E-05	3
log-sigma-5-0-mm-3D_glszm_GrayLevelNonUniformityNormalized	6,610044	4,13E-05	7,26E-05	3
wavelet-LHL_firstorder_RootMeanSquared	6,545966	4,49E-05	7,87E-05	3
wavelet-LHH_gldm_GrayLevelVariance	6,472571	5,02E-05	8,79E-05	1
wavelet-LHH_firstorder_Variance	6,444866	5,24E-05	9,15E-05	1
wavelet-HHL_firstorder_90Percentile	6,416826	5,46E-05	9,53E-05	1
wavelet-LHH_gldm_LargeDependenceEmphasis	6,413194	5,49E-05	9,55E-05	3
log-sigma-3-0-mm-3D_gldm_DependenceNonUniformity	6,409795	5,52E-05	9,58E-05	1
wavelet-LLH_glrlm_LowGrayLevelRunEmphasis	6,41583	5,51E-05	9,58E-05	3
original_gldm_LargeDependenceLowGrayLevelEmphasis	6,312079	6,3E-05	0,000109	3
wavelet-HLL_glszm_SizeZoneNonUniformityNormalized	6,288982	6,55E-05	0,000113	1
wavelet-LLH_gldm_DependenceEntropy	6,289016	6,62E-05	0,000114	1
wavelet-HLH_glrlm_LowGrayLevelRunEmphasis	6,281626	6,71E-05	0,000116	3
wavelet-HLH_gldm_LowGrayLevelEmphasis	6,268198	6,85E-05	0,000118	3
log-sigma-4-0-mm-3D_firstorder_Maximum	6,249196	6,96E-05	0,00012	3
log-sigma-4-0-mm-3D_glrlm_LongRunEmphasis	6,177695	7,8E-05	0,000134	3
log-sigma-2-0-mm-3D_firstorder_Energy	4,721889	8,14E-05	0,000139	3
log-sigma-2-0-mm-3D_firstorder_TotalEnergy	4,721889	8,14E-05	0,000139	3

wavelet-HHL_gldm_DependenceNonUniformityNormalized	6,125713	8,46E-05	0,000145	1
wavelet-LHH_glrlm_ShortRunHighGrayLevelEmphasis	6,12275	8,5E-05	0,000145	1
wavelet-LHH_glcm_SumSquares	6,065474	9,27E-05	0,000158	1
log-sigma-3-0-mm-3D_gldm_DependenceEntropy	6,049652	9,51E-05	0,000162	1
wavelet-LHL_gldm_DependenceVariance	6,046741	9,54E-05	0,000162	3
wavelet-HLH_glszm_SmallAreaHighGrayLevelEmphasis	5,908471	0,000118	0,0002	1
wavelet-LHH_glrlm_HighGrayLevelRunEmphasis	5,907385	0,000118	0,0002	1
wavelet-LHL_glrlm_GrayLevelVariance	5,891234	0,000121	0,000204	1
wavelet-LHL_firstorder_Variance	5,876249	0,000124	0,000209	1
wavelet-LHH_glszm_LargeAreaLowGrayLevelEmphasis	5,86925	0,000125	0,00021	3
wavelet-LHL_gldm_GrayLevelVariance	5,811634	0,000137	0,00023	1
wavelet-LHH_gldm_HighGrayLevelEmphasis	5,809522	0,000137	0,000231	1
wavelet-LHL_glszm_LowGrayLevelZoneEmphasis	5,804345	0,000138	0,000232	3
wavelet-LHH_glrlm_RunVariance	5,795728	0,00014	0,000235	3
wavelet-LHL_glcm_SumSquares	5,773802	0,000145	0,000242	1
wavelet-HLL_gldm_DependenceEntropy	5,762733	0,000148	0,000247	1
log-sigma-4-0-mm-3D_gldm_SmallDependenceHighGrayLevelEmphasis	5,738407	0,000153	0,000255	1
wavelet-HLL_gldm_LargeDependenceLowGrayLevelEmphasis	5,692415	0,000164	0,000273	3
wavelet-LHH_glcm_DifferenceVariance	5,66735	0,000171	0,000285	1
log-sigma-5-0-mm-3D_glcm_ClusterTendency	5,662707	0,000172	0,000286	1
wavelet-LLH_gldm_LowGrayLevelEmphasis	5,659181	0,000175	0,00029	3
log-sigma-3-0-mm-3D_glszm_SizeZoneNonUniformity	5,61841	0,000184	0,000305	1
wavelet-HLL_glszm_HighGrayLevelZoneEmphasis	5,607619	0,000188	0,00031	1
wavelet-LHL_glcm_Autocorrelation	5,597459	0,000191	0,000315	1
wavelet-LHL_gldm_SmallDependenceHighGrayLevelEmphasis	5,560335	0,000202	0,000333	1
wavelet-HHL_gldm_SmallDependenceEmphasis	5,543183	0,000208	0,000342	1
wavelet-HHH_glrlm_RunEntropy	5,52594	0,000214	0,000351	1
log-sigma-4-0-mm-3D_gldm_DependenceNonUniformity	5,521656	0,000215	0,000353	1
log-sigma-3-0-mm-3D_firstorder_Energy	4,34387	0,000217	0,000355	3
log-sigma-3-0-mm-3D_firstorder_TotalEnergy	4,34387	0,000217	0,000355	3
wavelet-LLL_gldm_LargeDependenceHighGrayLevelEmphasis	5,509868	0,000219	0,000358	1
wavelet-LLH_firstorder_Energy	4,328956	0,000225	0,000367	2
wavelet-LLH_firstorder_TotalEnergy	4,328955	0,000225	0,000367	2
wavelet-HLL_glrlm_ShortRunHighGrayLevelEmphasis	5,467583	0,000234	0,00038	1
wavelet-HLL_firstorder_90Percentile	5,464844	0,000234	0,000381	1
wavelet-HLL_glrlm_HighGrayLevelRunEmphasis	5,43064	0,000248	0,000402	1
wavelet-LLH_glrlm_GrayLevelNonUniformity	5,418155	0,000253	0,000409	3
wavelet-HLL_firstorder_Maximum	5,417393	0,000253	0,000409	1
wavelet-HLL_gldm_HighGrayLevelEmphasis	5,414295	0,000254	0,000411	1
wavelet-LHH_glcm_Autocorrelation	5,413834	0,000255	0,000411	1
wavelet-HLL_gldm_DependenceNonUniformity	5,402413	0,000259	0,000418	1
wavelet-HHH_firstorder_InterquartileRange	5,393223	0,000263	0,000423	1
wavelet-LHL_glcm_DifferenceVariance	5,385514	0,000266	0,000427	1
wavelet-HLL_glszm_SmallAreaLowGrayLevelEmphasis	5,381786	0,000267	0,000428	3
wavelet-LLL_glcm_Imc2	5,381079	0,000268	0,000429	1
log-sigma-5-0-mm-3D_glrlm_LongRunLowGrayLevelEmphasis	5,364341	0,000277	0,000444	3
log-sigma-3-0-mm-3D_glrlm_GrayLevelNonUniformity	5,349445	0,000282	0,00045	3

log-sigma-5-0-mm-3D_glszm_SmallAreaHighGrayLevelEmphasis	5,344827	0,000283	0,000452	1
log-sigma-3-0-mm-3D_glrlm_RunVariance	5,317546	0,000296	0,000471	3
wavelet-LLL_firstorder_Energy	4,204444	0,00031	0,000492	1
wavelet-LLL_firstorder_TotalEnergy	4,204444	0,00031	0,000492	1
log-sigma-2-0-mm-3D_firstorder_90Percentile	5,275256	0,000314	0,000497	3
log-sigma-5-0-mm-3D_gldm_SmallDependenceHighGrayLevelEmphasis	5,269027	0,000319	0,000505	1
log-sigma-2-0-mm-3D_glcm_ClusterProminence	5,212539	0,000349	0,000552	1
wavelet-LHH_glcm_Contrast	5,20748	0,000353	0,000558	1
original_firstorder_Energy	4,14128	0,000364	0,000574	1
original_firstorder_TotalEnergy	4,14128	0,000364	0,000574	1
wavelet-HLH_glrlm_ShortRunLowGrayLevelEmphasis	5,183697	0,000368	0,000578	3
wavelet-HLL_glrlm_LongRunHighGrayLevelEmphasis	5,177518	0,00037	0,000581	1
wavelet-LLH_glszm_SmallAreaEmphasis	5,141593	0,000395	0,00062	1
wavelet-HLL_glszm_SmallAreaHighGrayLevelEmphasis	5,13306	0,000397	0,000622	1
wavelet-HLL_glszm_SmallAreaEmphasis	5,095355	0,000425	0,000665	1
wavelet-HLH_gldm_DependenceEntropy	5,033144	0,000468	0,00073	1
log-sigma-5-0-mm-3D_glrlm_LongRunHighGrayLevelEmphasis	4,985761	0,000504	0,000786	1
log-sigma-4-0-mm-3D_glszm_SizeZoneNonUniformity	4,967839	0,000518	0,000806	1
log-sigma-4-0-mm-3D_firstorder_Skewness	4,915184	0,000564	0,000877	3
log-sigma-2-0-mm-3D_glcm_Idn	4,912012	0,000568	0,000882	3
log-sigma-4-0-mm-3D_firstorder_Energy	3,959924	0,000578	0,000895	3
log-sigma-4-0-mm-3D_firstorder_TotalEnergy	3,959924	0,000578	0,000895	3
wavelet-LHL_glrlm_LongRunLowGrayLevelEmphasis	4,865027	0,000613	0,000948	3
wavelet-LHH_glszm_SmallAreaEmphasis	4,847388	0,000632	0,000975	1
log-sigma-2-0-mm-3D_gldm_DependenceEntropy	4,837817	0,000641	0,000989	1
wavelet-LHH_glszm_SizeZoneNonUniformity	4,834774	0,000644	0,000992	1
wavelet-HLL_gldm_SmallDependenceHighGrayLevelEmphasis	4,825926	0,000652	0,001003	1
log-sigma-4-0-mm-3D_glszm_GrayLevelNonUniformity	4,806571	0,000674	0,001035	1
wavelet-HLL_glcm_Autocorrelation	4,786564	0,000696	0,001067	1
log-sigma-5-0-mm-3D_gldm_DependenceNonUniformity	4,73631	0,000756	0,001158	1
log-sigma-5-0-mm-3D_glrlm_LowGrayLevelRunEmphasis	4,701345	0,000802	0,001227	3
wavelet-HLL_glszm_SizeZoneNonUniformity	4,692068	0,000813	0,001241	1
wavelet-HHH_firstorder_Range	4,688189	0,000819	0,001249	1
original_gldm_LargeDependenceHighGrayLevelEmphasis	4,684277	0,000823	0,001253	1
wavelet-LLH_glrlm_LongRunLowGrayLevelEmphasis	4,690604	0,000827	0,001259	3
wavelet-HHL_firstorder_Maximum	4,650006	0,000871	0,001323	1
wavelet-LHL_gldm_DependenceNonUniformity	4,642162	0,000883	0,001339	1
wavelet-HLH_glcm_ClusterProminence	4,637126	0,000888	0,001345	1
log-sigma-2-0-mm-3D_glszm_GrayLevelNonUniformity	4,589032	0,000963	0,001457	1
wavelet-LHH_gldm_SmallDependenceHighGrayLevelEmphasis	4,58545	0,000967	0,00146	1
wavelet-HHL_glcm_Idn	4,586608	0,000967	0,00146	3
log-sigma-2-0-mm-3D_glrlm_GrayLevelNonUniformity	4,523185	0,001074	0,001618	3
wavelet-LHL_glrlm_LowGrayLevelRunEmphasis	4,513247	0,001091	0,001642	3
log-sigma-4-0-mm-3D_firstorder_Kurtosis	4,474238	0,001162	0,001746	3
log-sigma-5-0-mm-3D_firstorder_Energy	3,668134	0,001206	0,001808	3
log-sigma-5-0-mm-3D_firstorder_TotalEnergy	3,668134	0,001206	0,001808	3
wavelet-HHL_glcm_Contrast	4,4441	0,001223	0,001831	1

wavelet-HHH_firstorder_Minimum	4,388739	0,001342	0,002006	3
wavelet-HHL_glszm_LargeAreaLowGrayLevelEmphasis	4,380058	0,001357	0,002026	3
wavelet-LHL_glrlm_ShortRunLowGrayLevelEmphasis	4,349838	0,001431	0,002133	3
log-sigma-5-0-mm-3D_gldm_LowGrayLevelEmphasis	4,34981	0,001434	0,002135	3
wavelet-LHL_firstorder_Energy	3,593634	0,001453	0,002158	3
wavelet-LHL_firstorder_TotalEnergy	3,593634	0,001453	0,002158	3
log-sigma-2-0-mm-3D_glszm_SizeZoneNonUniformityNormalized	4,279267	0,0016	0,002372	1
wavelet-LHL_gldm_LowGrayLevelEmphasis	4,282351	0,001602	0,002372	3
log-sigma-5-0-mm-3D_glcm_Imc2	4,239514	0,001731	0,002561	1
wavelet-LHL_glcm_Contrast	4,22228	0,001769	0,002613	1
wavelet-HLH_glrlm_GrayLevelNonUniformity	4,170687	0,001933	0,002851	3
wavelet-LHH_firstorder_Mean	4,166118	0,001945	0,002865	1
original_glcm_Correlation	4,158833	0,00197	0,002897	1
wavelet-LHH_glrlm_LongRunHighGrayLevelEmphasis	4,14228	0,002027	0,002978	1
wavelet-LHH_firstorder_RootMeanSquared	4,09198	0,002204	0,003234	1
wavelet-LHL_firstorder_Maximum	4,07941	0,002253	0,003297	1
wavelet-HHH_glcm_SumEntropy	4,080417	0,00225	0,003297	1
wavelet-HHL_glcm_JointAverage	4,072393	0,00228	0,003333	1
wavelet-HHL_gldm_DependenceNonUniformity	4,064983	0,002309	0,003371	1
wavelet-LLH_glcm_Idn	4,049565	0,00237	0,003454	3
log-sigma-5-0-mm-3D_glrlm_RunVariance	4,02901	0,002449	0,003565	3
wavelet-LHH_glrlm_GrayLevelNonUniformity	4,02637	0,002465	0,003584	3
log-sigma-5-0-mm-3D_gldm_LargeDependenceLowGrayLevelEmphasis	4,007591	0,002565	0,003724	3
log-sigma-3-0-mm-3D_glcm_ClusterProminence	3,985694	0,002635	0,00382	1
log-sigma-3-0-mm-3D_firstorder_Kurtosis	3,968929	0,002709	0,003924	3
log-sigma-3-0-mm-3D_glszm_GrayLevelNonUniformity	3,967171	0,002724	0,00394	1
wavelet-LHL_glszm_SizeZoneNonUniformity	3,941789	0,002844	0,004108	1
log-sigma-5-0-mm-3D_glrlm_ShortRunLowGrayLevelEmphasis	3,920465	0,00295	0,004256	3
wavelet-HHL_glszm_LowGrayLevelZoneEmphasis	3,912998	0,002988	0,004305	3
log-sigma-5-0-mm-3D_glszm_SizeZoneNonUniformity	3,90846	0,003002	0,00432	1
wavelet-HHH_firstorder_Variance	3,883307	0,003142	0,004516	1
wavelet-HLH_glszm_GrayLevelNonUniformity	3,832048	0,003429	0,004921	1
wavelet-LLH_gldm_GrayLevelNonUniformity	3,816092	0,003524	0,00505	3
wavelet-HLL_glrlm_LongRunLowGrayLevelEmphasis	3,807563	0,003574	0,005117	3
wavelet-LHH_firstorder_TotalEnergy	3,212505	0,003724	0,005323	2
wavelet-LHH_glcm_ClusterProminence	3,773163	0,003788	0,005408	1
wavelet-LHL_glszm_LargeAreaLowGrayLevelEmphasis	3,75889	0,003875	0,005525	3
wavelet-LHL_gldm_LargeDependenceLowGrayLevelEmphasis	3,75187	0,003908	0,005566	3
log-sigma-4-0-mm-3D_glrlm_RunVariance	3,735016	0,004043	0,00575	3
wavelet-HHH_glszm_ZoneEntropy	3,722666	0,004136	0,005875	1
wavelet-HHL_firstorder_Minimum	3,691927	0,004359	0,006185	3
wavelet-HHH_firstorder_Entropy	3,663284	0,004579	0,006489	1
wavelet-HHL_glcm_SumSquares	3,650472	0,004679	0,006622	1
wavelet-LLH_firstorder_Kurtosis	3,641092	0,004757	0,006723	3
wavelet-HHH_glszm_SmallAreaEmphasis	3,623657	0,004903	0,006921	1
log-sigma-2-0-mm-3D_gldm_SmallDependenceLowGrayLevelEmphasis	3,596395	0,005136	0,007241	1

log-sigma-3-0-mm-3D_glcm_Idmn	3,569949	0,005375	0,007569	3
wavelet-LHH_glcm_Imc1	3,560037	0,005468	0,00769	3
wavelet-LLH_glszm_GrayLevelNonUniformity	3,515143	0,005912	0,008304	1
wavelet-LLH_glrlm_RunLengthNonUniformity	3,489642	0,006178	0,008667	1
wavelet-HHH_glrlm_GrayLevelNonUniformityNormalized	3,485983	0,006218	0,008711	3
wavelet-HHL_glrlm_GrayLevelNonUniformity	3,475803	0,006329	0,008855	3
wavelet-HHH_glcm_DifferenceEntropy	3,439721	0,006737	0,009415	1
original_glszm_GrayLevelNonUniformity	3,437185	0,006767	0,009445	2
log-sigma-2-0-mm-3D_glrlm_RunLengthNonUniformity	3,416999	0,007007	0,009768	1
wavelet-HHH_glcm_JointEnergy	3,410843	0,007084	0,009863	3
wavelet-LHH_gldm_DependenceEntropy	3,407439	0,007125	0,009908	1
original_glrlm_GrayLevelNonUniformity	3,398978	0,007229	0,01004	3
wavelet-LHH_glrlm_LongRunLowGrayLevelEmphasis	3,38598	0,007403	0,010268	3
wavelet-LLL_gldm_SmallDependenceLowGrayLevelEmphasis	3,376213	0,00752	0,010419	3
wavelet-LHL_firstorder_90Percentile	3,358996	0,007746	0,010718	1
wavelet-HHH_firstorder_Uniformity	3,358542	0,007756	0,010718	3
wavelet-LHH_gldm_SmallDependenceLowGrayLevelEmphasis	3,350409	0,007865	0,010856	1
wavelet-HHL_firstorder_Variance	3,345313	0,007934	0,010937	1
wavelet-HLL_glcm_ClusterProminence	3,327581	0,00817	0,011236	1
wavelet-HLL_glrlm_GrayLevelNonUniformity	3,328597	0,00817	0,011236	3
log-sigma-2-0-mm-3D_firstorder_Kurtosis	3,319164	0,008308	0,01141	3
wavelet-LHH_glrlm_LowGrayLevelRunEmphasis	3,315248	0,008369	0,011476	3
wavelet-HLH_glcm_Correlation	3,31481	0,008376	0,011476	1
wavelet-HHL_gldm_GrayLevelVariance	3,296379	0,008639	0,011822	1
log-sigma-5-0-mm-3D_glcm_Idmn	3,287456	0,008774	0,011992	3
wavelet-LLL_glcm_ClusterProminence	2,854529	0,008827	0,01205	1
wavelet-HHL_glszm_GrayLevelVariance	3,280594	0,008881	0,012109	1
wavelet-HHH_glcm_JointEntropy	3,256007	0,009273	0,012627	1
wavelet-HHL_glrlm_GrayLevelVariance	3,254584	0,009292	0,012638	1
wavelet-HHH_glcm_Idmn	3,251948	0,00934	0,012687	1
log-sigma-3-0-mm-3D_glrlm_RunLengthNonUniformity	3,248017	0,0094	0,012754	1
log-sigma-4-0-mm-3D_gldm_SmallDependenceLowGrayLevelEmphasis	3,245194	0,009445	0,012799	1
wavelet-LHH_glrlm_ShortRunLowGrayLevelEmphasis	3,243695	0,00948	0,012831	3
wavelet-HHH_glszm_GrayLevelNonUniformityNormalized	3,239776	0,00954	0,012897	3
wavelet-HLL_firstorder_Energy	2,816163	0,009669	0,01304	2
wavelet-HLL_firstorder_TotalEnergy	2,816163	0,009669	0,01304	2
wavelet-LHL_firstorder_Kurtosis	3,231167	0,009682	0,013041	3
wavelet-HLL_glrlm_LowGrayLevelRunEmphasis	3,22764	0,00974	0,013103	3
log-sigma-2-0-mm-3D_gldm_GrayLevelNonUniformity	3,219747	0,009875	0,01327	3
wavelet-LHH_firstorder_Energy	3,212506	0,010005	0,013428	2
original_glcm_ClusterProminence	3,210387	0,010021	0,013433	1
wavelet-HHH_glszm_GrayLevelVariance	3,160962	0,010947	0,014657	1
wavelet-LLH_gldm_LargeDependenceLowGrayLevelEmphasis	3,165039	0,011011	0,014725	3
wavelet-HHH_glcm_Idn	3,15655	0,011032	0,014736	1
wavelet-HHH_glcm_Imc2	3,126266	0,01163	0,015516	1
log-sigma-4-0-mm-3D_glcm_Imc1	3,11407	0,011847	0,015768	3
wavelet-HLL_glrlm_ShortRunLowGrayLevelEmphasis	3,116047	0,011839	0,015768	3

wavelet-HLH_gldm_GrayLevelNonUniformity	3,109426	0,011981	0,015927	3
log-sigma-3-0-mm-3D_glcm_Correlation	3,108214	0,012001	0,015935	1
wavelet-LHH_gldm_LowGrayLevelEmphasis	3,09668	0,01226	0,016259	3
log-sigma-3-0-mm-3D_glcm_InverseVariance	3,024107	0,012567	0,016647	3
wavelet-HLL_glrlm_RunLengthNonUniformity	3,081517	0,012582	0,016647	1
wavelet-LHH_firstorder_Median	3,077116	0,012677	0,016753	1
wavelet-LHL_glszm_SmallAreaLowGrayLevelEmphasis	3,072952	0,012757	0,016819	3
wavelet-HHL_glrlm_ShortRunLowGrayLevelEmphasis	3,074042	0,012752	0,016819	3
wavelet-HHL_glrlm_LowGrayLevelRunEmphasis	3,066801	0,012916	0,017008	3
wavelet-HHL_glrlm_LongRunLowGrayLevelEmphasis	3,055927	0,013165	0,017316	3
wavelet-HLL_gldm_GrayLevelNonUniformity	3,053716	0,013208	0,017352	3
wavelet-HHL_glcm_ClusterTendency	3,046166	0,013379	0,017556	1
wavelet-HHH_glszm_LowGrayLevelZoneEmphasis	3,020682	0,013999	0,018348	3
wavelet-HHH_firstorder_Maximum	2,99754	0,014579	0,019086	1
wavelet-HHL_glszm_SizeZoneNonUniformity	2,984541	0,014913	0,0195	1
wavelet-LHH_gldm_GrayLevelNonUniformity	2,983609	0,014941	0,019513	3
wavelet-LHL_glcm_Idn	2,980454	0,015023	0,019598	3
wavelet-HLH_glszm_SizeZoneNonUniformity	2,96803	0,01535	0,020001	1
wavelet-HHH_glszm_ZonePercentage	2,966555	0,015393	0,020035	1
wavelet-LLL_glrlm_GrayLevelNonUniformity	2,964206	0,015454	0,020091	3
wavelet-HHL_gldm_LargeDependenceLowGrayLevelEmphasis	2,947721	0,015911	0,02066	3
log-sigma-5-0-mm-3D_glszm_GrayLevelNonUniformity	2,936019	0,016234	0,021055	1
wavelet-LHL_glcm_ClusterProminence	2,932743	0,016316	0,021137	1
wavelet-LHL_glrlm_GrayLevelNonUniformity	2,929342	0,016435	0,021266	3
original_glrlm_RunLengthNonUniformity	2,927668	0,016485	0,021287	1
wavelet-HHL_gldm_GrayLevelNonUniformity	2,927504	0,016489	0,021287	3
wavelet-HHL_glcm_DifferenceVariance	2,865067	0,018401	0,023728	1
wavelet-LLH_gldm_LargeDependenceHighGrayLevelEmphasis	2,860197	0,018561	0,023907	1
original_glszm_SmallAreaLowGrayLevelEmphasis	2,854503	0,018695	0,024052	3
log-sigma-5-0-mm-3D_glszm_SmallAreaEmphasis	2,852891	0,018765	0,024115	1
wavelet-HHL_glcm_Idmn	2,850608	0,018879	0,024233	3
wavelet-HHL_gldm_LowGrayLevelEmphasis	2,837993	0,019311	0,024759	3
log-sigma-4-0-mm-3D_glrlm_RunLengthNonUniformity	2,827145	0,019671	0,025192	1
wavelet-HLL_gldm_LowGrayLevelEmphasis	2,822618	0,01983	0,025366	3
wavelet-HLH_glrlm_RunLengthNonUniformity	2,820896	0,019895	0,02542	1
original_gldm_GrayLevelNonUniformity	2,806028	0,02042	0,026061	3
wavelet-LHH_gldm_LargeDependenceLowGrayLevelEmphasis	2,744163	0,022781	0,029041	3
wavelet-LLH_glszm_LargeAreaLowGrayLevelEmphasis	2,739137	0,022938	0,029208	3
wavelet-LLL_gldm_GrayLevelNonUniformity	2,703881	0,024449	0,031097	3
wavelet-LHL_firstorder_Skewness	2,690117	0,025045	0,031818	3
log-sigma-4-0-mm-3D_glcm_ClusterProminence	2,658607	0,026458	0,033576	1
wavelet-LHL_glrlm_RunLengthNonUniformity	2,654704	0,026676	0,033814	1
wavelet-LHL_gldm_GrayLevelNonUniformity	2,6494	0,026923	0,034088	3
wavelet-LHH_glrlm_RunLengthNonUniformity	2,601923	0,029282	0,037033	1
log-sigma-5-0-mm-3D_glrlm_RunLengthNonUniformity	2,596536	0,029556	0,037337	1
log-sigma-3-0-mm-3D_gldm_GrayLevelNonUniformity	2,595242	0,029617	0,037373	3
wavelet-HHL_glrlm_RunLengthNonUniformity	2,582714	0,030293	0,038183	1

wavelet-HLH_glszm_SizeZoneNonUniformityNormalized	2,577406	0,030474	0,038367	3
wavelet-HHH_glszm_SmallAreaHighGrayLevelEmphasis	2,572575	0,030837	0,038781	1
wavelet-LLL_glrlm_RunLengthNonUniformity	2,565839	0,03121	0,039205	1
wavelet-HLH_gldm_DependenceNonUniformity	2,554872	0,031821	0,039928	1
log-sigma-3-0-mm-3D_firstorder_Maximum	2,548394	0,032154	0,0403	3
wavelet-HLL_firstorder_Median	2,485033	0,035993	0,045061	3
wavelet-HHH_gldm_SmallDependenceEmphasis	2,464156	0,037347	0,046704	1
log-sigma-2-0-mm-3D_glcm_Correlation	2,448752	0,038373	0,047934	1
log-sigma-4-0-mm-3D_glcm_Idmn	2,445389	0,038602	0,048166	3
wavelet-HHH_glszm_SizeZoneNonUniformity	2,44213	0,038827	0,048393	1
wavelet-LHL_glcm_Idmn	2,432731	0,039481	0,049153	3
wavelet-HHH_glcm_JointAverage	2,431315	0,039579	0,049221	1
log-sigma-5-0-mm-3D_gldm_GrayLevelNonUniformity	2,417645	0,04054	0,05036	n.s.
wavelet-LHL_gldm_LargeDependenceHighGrayLevelEmphasis	2,394953	0,042196	0,052357	n.s.
wavelet-HHL_glszm_SmallAreaHighGrayLevelEmphasis	2,394391	0,042242	0,052357	n.s.
log-sigma-4-0-mm-3D_gldm_GrayLevelNonUniformity	2,370767	0,044035	0,054519	n.s.
wavelet-HHL_glszm_HighGrayLevelZoneEmphasis	2,361048	0,044805	0,055411	n.s.
wavelet-HLL_gldm_LargeDependenceHighGrayLevelEmphasis	2,354241	0,04534	0,056011	n.s.
wavelet-HLH_gldm_DependenceNonUniformityNormalized	2,32259	0,047944	0,059162	n.s.
wavelet-HHL_glrlm_ShortRunHighGrayLevelEmphasis	2,31569	0,048537	0,059828	n.s.
wavelet-HHL_gldm_SmallDependenceHighGrayLevelEmphasis	2,280844	0,051605	0,06354	n.s.
wavelet-LHH_glszm_GrayLevelNonUniformity	2,262659	0,053301	0,065556	n.s.
wavelet-HHH_glrlm_LongRunHighGrayLevelEmphasis	2,22239	0,057215	0,070293	n.s.
wavelet-HHH_glrlm_GrayLevelVariance	2,212241	0,058246	0,071481	n.s.
wavelet-HLL_glszm_LargeAreaLowGrayLevelEmphasis	2,203706	0,059073	0,072416	n.s.
wavelet-HHH_gldm_DependenceVariance	2,200618	0,059455	0,072805	n.s.
wavelet-HHH_glrlm_GrayLevelNonUniformity	2,19015	0,060561	0,074078	n.s.
wavelet-HHH_glszm_HighGrayLevelZoneEmphasis	2,181846	0,061445	0,075078	n.s.
wavelet-HHH_glcm_DifferenceVariance	2,1741	0,062288	0,076023	n.s.
wavelet-HHL_glrlm_HighGrayLevelRunEmphasis	2,171566	0,062564	0,076278	n.s.
wavelet-HHH_gldm_LargeDependenceHighGrayLevelEmphasis	2,159448	0,063915	0,07784	n.s.
wavelet-HHL_gldm_HighGrayLevelEmphasis	2,1579	0,064085	0,077962	n.s.
wavelet-HHH_glrlm_ShortRunLowGrayLevelEmphasis	2,129159	0,067412	0,08192	n.s.
wavelet-HHH_glcm_Correlation	2,114914	0,069139	0,083927	n.s.
wavelet-HLL_glcm_ClusterShade	2,092527	0,071832	0,087102	n.s.
wavelet-HLL_glcm_Idn	2,091913	0,071957	0,087159	n.s.
log-sigma-3-0-mm-3D_gldm_SmallDependenceLowGrayLevelEmphasis	2,089235	0,072274	0,087448	n.s.
wavelet-HHH_gldm_DependenceNonUniformity	2,082903	0,07311	0,088365	n.s.
log-sigma-3-0-mm-3D_glszm_SizeZoneNonUniformityNormalized	2,074016	0,074192	0,089575	n.s.
wavelet-HLL_glcm_Idmn	2,072049	0,074504	0,089854	n.s.
original_glcm_Idn	2,051807	0,077198	0,093004	n.s.
wavelet-HHH_firstorder_Median	2,04912	0,077508	0,093276	n.s.
wavelet-HHH_gldm_GrayLevelNonUniformity	2,045191	0,078101	0,093889	n.s.
wavelet-HHH_gldm_DependenceEntropy	2,037697	0,079132	0,095027	n.s.
wavelet-LHH_glszm_LargeAreaEmphasis	2,032392	0,079854	0,095791	n.s.
wavelet-HLH_firstorder_TotalEnergy	1,877233	0,080569	0,096545	n.s.
wavelet-LHH_gldm_DependenceNonUniformity	2,025628	0,080819	0,096741	n.s.

log-sigma-3-0-mm-3D_gldm_LargeDependenceHighGrayLevelEmphasis	2,018023	0,081887	0,097914	n.s.
wavelet-HLH_glcm_InverseVariance	2,0093	0,083199	0,099377	n.s.
wavelet-HHH_glrlm_LowGrayLevelRunEmphasis	2,004635	0,083837	0,100032	n.s.
log-sigma-4-0-mm-3D_glcm_Correlation	1,997258	0,084911	0,101205	n.s.
wavelet-HLL_gldm_SmallDependenceLowGrayLevelEmphasis	1,972526	0,088654	0,105554	n.s.
wavelet-HHH_glszm_LargeAreaLowGrayLevelEmphasis	1,967081	0,089468	0,10641	n.s.
wavelet-LLL_glcm_Idn	1,942304	0,093459	0,111039	n.s.
wavelet-HHH_glrlm_HighGrayLevelRunEmphasis	1,928481	0,095728	0,113614	n.s.
wavelet-HLH_firstorder_Kurtosis	1,925107	0,09626	0,114124	n.s.
log-sigma-2-0-mm-3D_glszm_LargeAreaHighGrayLevelEmphasis	1,921118	0,096966	0,114774	n.s.
wavelet-HHH_gldm_LowGrayLevelEmphasis	1,920853	0,097014	0,114774	n.s.
log-sigma-5-0-mm-3D_glcm_InverseVariance	1,91846	0,097446	0,115164	n.s.
original_firstorder_Skewness	1,917161	0,097608	0,115233	n.s.
wavelet-HLH_gldm_SmallDependenceLowGrayLevelEmphasis	1,908784	0,099053	0,116816	n.s.
log-sigma-2-0-mm-3D_firstorder_Maximum	1,905015	0,09968	0,117431	n.s.
wavelet-HHH_glcm_Autocorrelation	1,902351	0,100174	0,117889	n.s.
wavelet-HHH_gldm_HighGrayLevelEmphasis	1,898357	0,100871	0,118585	n.s.
log-sigma-5-0-mm-3D_glcm_ClusterProminence	1,893277	0,101716	0,119452	n.s.
wavelet-LHH_glcm_Correlation	1,8876	0,102744	0,120533	n.s.
log-sigma-2-0-mm-3D_glszm_SmallAreaLowGrayLevelEmphasis	1,881276	0,103791	0,121634	n.s.
wavelet-HLH_firstorder_Energy	1,877233	0,104647	0,122508	n.s.
wavelet-HLH_glszm_LargeAreaLowGrayLevelEmphasis	1,875281	0,104953	0,122737	n.s.
wavelet-HHH_gldm_GrayLevelVariance	1,871152	0,105747	0,123537	n.s.
wavelet-HHH_glrlm_RunLengthNonUniformity	1,826966	0,114153	0,133219	n.s.
wavelet-HHL_glcm_Autocorrelation	1,820555	0,115413	0,134408	n.s.
wavelet-HHH_glrlm_ShortRunHighGrayLevelEmphasis	1,820959	0,115331	0,134408	n.s.
wavelet-HHH_glrlm_ShortRunEmphasis	1,810557	0,117427	0,136611	n.s.
wavelet-HHH_glcm_Imc1	1,807634	0,118021	0,137159	n.s.
log-sigma-3-0-mm-3D_firstorder_Skewness	1,781701	0,123346	0,143199	n.s.
log-sigma-4-0-mm-3D_gldm_LargeDependenceHighGrayLevelEmphasis	1,773172	0,125231	0,145237	n.s.
wavelet-LHH_glcm_Idmn	1,752902	0,129672	0,150231	n.s.
wavelet-LLL_glszm_LargeAreaLowGrayLevelEmphasis	1,747701	0,130751	0,151324	n.s.
wavelet-HLH_firstorder_RootMeanSquared	1,709195	0,139743	0,161564	n.s.
wavelet-LHL_glcm_Correlation	1,704404	0,1409	0,162671	n.s.
wavelet-HHH_glcm_ClusterTendency	1,703989	0,140991	0,162671	n.s.
log-sigma-5-0-mm-3D_glcm_ClusterShade	1,699191	0,142039	0,163711	n.s.
log-sigma-2-0-mm-3D_glszm_LargeAreaLowGrayLevelEmphasis	1,68737	0,144991	0,166942	n.s.
log-sigma-5-0-mm-3D_glszm_SmallAreaLowGrayLevelEmphasis	1,685184	0,145548	0,16741	n.s.
log-sigma-3-0-mm-3D_glszm_LargeAreaHighGrayLevelEmphasis	1,681121	0,146601	0,168448	n.s.
wavelet-LLL_firstorder_Kurtosis	1,670032	0,149311	0,171386	n.s.
wavelet-LLH_glszm_LargeAreaEmphasis	1,664055	0,150882	0,172989	n.s.
wavelet-HLH_glcm_ClusterShade	1,663267	0,151017	0,172989	n.s.
wavelet-HHH_glcm_InverseVariance	1,641802	0,15317	0,175276	n.s.
wavelet-HHH_glrlm_LongRunLowGrayLevelEmphasis	1,6534	0,153683	0,175682	n.s.
log-sigma-4-0-mm-3D_glszm_LargeAreaLowGrayLevelEmphasis	1,649423	0,154672	0,176632	n.s.
wavelet-HHL_glszm_LargeAreaEmphasis	1,636287	0,158194	0,18047	n.s.

wavelet-HHH_glszm_LargeAreaEmphasis	1,625341	0,161167	0,183674	n.s.
log-sigma-4-0-mm-3D_glcm_InverseVariance	1,614522	0,164292	0,187045	n.s.
wavelet-HHL_glszm_SmallAreaEmphasis	1,612101	0,16482	0,187455	n.s.
wavelet-HLL_glszm_LargeAreaEmphasis	1,597534	0,168882	0,19188	n.s.
wavelet-HLH_glszm_SmallAreaEmphasis	1,589753	0,171154	0,194264	n.s.
log-sigma-5-0-mm-3D_glszm_LargeAreaLowGrayLevelEmphasis	1,575378	0,175294	0,198762	n.s.
wavelet-HHH_gldm_LargeDependenceLowGrayLevelEmphasis	1,573215	0,175973	0,199329	n.s.
wavelet-HHH_glcm_SumSquares	1,56333	0,17893	0,202474	n.s.
log-sigma-5-0-mm-3D_glszm_LargeAreaHighGrayLevelEmphasis	1,559891	0,179954	0,203427	n.s.
wavelet-HHH_gldm_LargeDependenceEmphasis	1,555317	0,181361	0,204809	n.s.
wavelet-HHH_glcm_MaximumProbability	1,549178	0,183242	0,206725	n.s.
wavelet-HHH_glcm_Idm	1,546292	0,184121	0,207507	n.s.
wavelet-HHH_gldm_DependenceNonUniformityNormalized	1,539989	0,18608	0,209504	n.s.
wavelet-LLH_glszm_LargeAreaHighGrayLevelEmphasis	1,529229	0,18945	0,213084	n.s.
log-sigma-2-0-mm-3D_glszm_LargeAreaEmphasis	1,523228	0,191318	0,214969	n.s.
wavelet-LLL_glszm_GrayLevelNonUniformity	1,520324	0,192296	0,21585	n.s.
log-sigma-3-0-mm-3D_glszm_SmallAreaLowGrayLevelEmphasis	1,502512	0,194588	0,218158	n.s.
wavelet-HLH_gldm_LargeDependenceHighGrayLevelEmphasis	1,512725	0,194742	0,218158	n.s.
wavelet-HHH_glcm_DifferenceAverage	1,510128	0,195587	0,218885	n.s.
wavelet-HHH_glszm_GrayLevelNonUniformity	1,50327	0,197836	0,22118	n.s.
wavelet-LLL_glcm_Idmn	1,497008	0,199907	0,223273	n.s.
log-sigma-5-0-mm-3D_glcm_Imc1	1,490292	0,202057	0,225449	n.s.
wavelet-HHL_firstorder_TotalEnergy	1,41571	0,204081	0,22748	n.s.
log-sigma-3-0-mm-3D_glszm_LargeAreaLowGrayLevelEmphasis	1,480322	0,205471	0,228801	n.s.
log-sigma-5-0-mm-3D_gldm_SmallDependenceLowGrayLevelEmphasis	1,47136	0,208584	0,232036	n.s.
wavelet-HLH_glszm_LargeAreaEmphasis	1,465714	0,210534	0,233973	n.s.
log-sigma-5-0-mm-3D_glszm_LargeAreaEmphasis	1,4611	0,21213	0,235513	n.s.
log-sigma-4-0-mm-3D_glszm_LargeAreaHighGrayLevelEmphasis	1,460171	0,212483	0,235671	n.s.
wavelet-LHH_gldm_LargeDependenceHighGrayLevelEmphasis	1,457215	0,213536	0,236604	n.s.
wavelet-HHH_glcm_Id	1,448775	0,216531	0,239685	n.s.
wavelet-HHH_glcm_Contrast	1,445934	0,217544	0,240568	n.s.
log-sigma-3-0-mm-3D_glszm_LargeAreaEmphasis	1,443308	0,218451	0,241333	n.s.
wavelet-HHH_glrlm_LongRunEmphasis	1,44097	0,219338	0,242072	n.s.
wavelet-HLH_glcm_Idmn	1,433893	0,221897	0,244656	n.s.
wavelet-LLH_glszm_ZoneVariance	1,428968	0,223671	0,246368	n.s.
wavelet-HHH_gldm_SmallDependenceHighGrayLevelEmphasis	1,417423	0,227974	0,250862	n.s.
wavelet-HHL_firstorder_Energy	1,415711	0,228637	0,251344	n.s.
wavelet-HHL_firstorder_Kurtosis	1,41446	0,229094	0,251599	n.s.
log-sigma-4-0-mm-3D_glszm_LargeAreaEmphasis	1,410011	0,230737	0,252821	n.s.
wavelet-HLL_glszm_ZoneVariance	1,410257	0,230644	0,252821	n.s.
wavelet-HHL_glszm_ZoneVariance	1,409685	0,230886	0,252821	n.s.
wavelet-HHH_glrlm_RunPercentage	1,402147	0,233769	0,255728	n.s.
wavelet-LHL_glszm_LargeAreaEmphasis	1,401133	0,234117	0,255859	n.s.
wavelet-LLL_glszm_LargeAreaEmphasis	1,399457	0,234738	0,256286	n.s.
original_glszm_LargeAreaEmphasis	1,39494	0,236497	0,257906	n.s.
wavelet-HHL_glrlm_LongRunHighGrayLevelEmphasis	1,394556	0,236683	0,257906	n.s.
log-sigma-2-0-mm-3D_glszm_ZoneVariance	1,390249	0,238314	0,25943	n.s.

wavelet-LHH_glszm_ZoneVariance	1,38912	0,238784	0,25969	n.s.
wavelet-HHH_glrlm_RunLengthNonUniformityNormalized	1,380496	0,242176	0,263122	n.s.
wavelet-HHL_glszm_SizeZoneNonUniformityNormalized	1,365841	0,247998	0,269186	n.s.
wavelet-LLH_glszm_SizeZoneNonUniformityNormalized	1,356141	0,251817	0,273067	n.s.
log-sigma-5-0-mm-3D_gldm_LargeDependenceHighGrayLevelEmphasis	1,351608	0,253797	0,274414	n.s.
wavelet-HLH_firstorder_Mean	1,352189	0,253563	0,274414	n.s.
wavelet-HLH_glszm_LargeAreaHighGrayLevelEmphasis	1,351882	0,253689	0,274414	n.s.
wavelet-HLH_firstorder_Skewness	1,347343	0,255493	0,275982	n.s.
original_glcm_Idmn	1,346135	0,256062	0,276149	n.s.
wavelet-LHL_glszm_GrayLevelNonUniformity	1,345951	0,256141	0,276149	n.s.
wavelet-HHH_firstorder_Kurtosis	1,326824	0,264146	0,284504	n.s.
wavelet-HHH_firstorder_Skewness	1,325347	0,264701	0,284827	n.s.
wavelet-LLL_glcm_Correlation	1,317995	0,267933	0,288028	n.s.
log-sigma-3-0-mm-3D_glszm_ZoneVariance	1,305601	0,273285	0,293499	n.s.
wavelet-HHL_glcm_ClusterProminence	1,303606	0,274152	0,294148	n.s.
wavelet-HHH_glszm_ZoneVariance	1,301096	0,275301	0,295097	n.s.
log-sigma-4-0-mm-3D_glcm_ClusterShade	1,293929	0,278378	0,29811	n.s.
original_glszm_ZoneVariance	1,290786	0,279841	0,29939	n.s.
log-sigma-2-0-mm-3D_glcm_Idmn	1,284764	0,282576	0,301738	n.s.
wavelet-HHL_glcm_ClusterShade	1,28491	0,282501	0,301738	n.s.
wavelet-HHL_glszm_SmallAreaLowGrayLevelEmphasis	1,259189	0,29423	0,313883	n.s.
wavelet-LLH_glszm_SmallAreaLowGrayLevelEmphasis	1,245363	0,30071	0,320377	n.s.
wavelet-LLL_glszm_ZoneVariance	1,24507	0,30089	0,320377	n.s.
wavelet-LLH_gldm_SmallDependenceLowGrayLevelEmphasis	1,239062	0,303793	0,32316	n.s.
wavelet-LHH_gldm_DependenceNonUniformityNormalized	1,236182	0,305177	0,324125	n.s.
wavelet-HLH_glszm_ZoneVariance	1,235958	0,30528	0,324125	n.s.
wavelet-LHL_glszm_ZoneVariance	1,233072	0,306654	0,325274	n.s.
log-sigma-4-0-mm-3D_glszm_ZoneVariance	1,229992	0,308141	0,326542	n.s.
wavelet-HHH_glcm_ClusterProminence	1,218529	0,313753	0,332174	n.s.
wavelet-HHH_glrlm_RunVariance	1,205656	0,320154	0,33863	n.s.
wavelet-LLH_firstorder_Skewness	1,201453	0,32225	0,340525	n.s.
log-sigma-5-0-mm-3D_firstorder_Skewness	1,200646	0,322612	0,340586	n.s.
wavelet-HHL_gldm_LargeDependenceHighGrayLevelEmphasis	1,182526	0,331881	0,350041	n.s.
wavelet-LLL_glszm_LargeAreaHighGrayLevelEmphasis	1,163399	0,341824	0,360188	n.s.
log-sigma-5-0-mm-3D_glszm_ZoneVariance	1,162189	0,342448	0,360506	n.s.
wavelet-HHL_firstorder_RootMeanSquared	1,160063	0,343602	0,36138	n.s.
wavelet-LLL_glcm_ClusterShade	1,153741	0,344372	0,36185	n.s.
original_glszm_LargeAreaLowGrayLevelEmphasis	1,157033	0,345141	0,362317	n.s.
log-sigma-5-0-mm-3D_firstorder_Kurtosis	1,153763	0,346915	0,363837	n.s.
wavelet-HHH_glszm_LargeAreaHighGrayLevelEmphasis	1,150903	0,348475	0,365132	n.s.
wavelet-HHL_glszm_LargeAreaHighGrayLevelEmphasis	1,110266	0,370743	0,3881	n.s.
wavelet-LHL_gldm_SmallDependenceLowGrayLevelEmphasis	1,109493	0,371179	0,388193	n.s.
original_firstorder_Kurtosis	1,105322	0,373493	0,390248	n.s.
wavelet-LLL_firstorder_Skewness	1,094962	0,379391	0,39604	n.s.
log-sigma-5-0-mm-3D_glszm_SizeZoneNonUniformityNormalized	1,079893	0,388131	0,404786	n.s.
wavelet-LHH_glszm_LargeAreaHighGrayLevelEmphasis	1,073155	0,392031	0,408473	n.s.

log-sigma-2-0-mm-3D_gldm_LargeDependenceHighGrayLevelEmphasis	1,069589	0,394124	0,41027	n.s.
wavelet-LLH_glcm_ClusterShade	1,046679	0,407721	0,42403	n.s.
wavelet-LHH_glcm_InverseVariance	1,023426	0,421902	0,43837	n.s.
wavelet-LHH_glszm_SizeZoneNonUniformityNormalized	0,976751	0,449762	0,466884	n.s.
original_glcm_ClusterShade	0,961683	0,459668	0,476283	n.s.
original_glszm_LargeAreaHighGrayLevelEmphasis	0,964568	0,459415	0,476283	n.s.
log-sigma-5-0-mm-3D_glcm_Correlation	0,944102	0,472942	0,489583	n.s.
wavelet-HHH_glcm_ClusterShade	0,905148	0,499322	0,516412	n.s.
wavelet-HHH_firstorder_Energy	0,875373	0,52018	0,536991	n.s.
wavelet-HHH_firstorder_TotalEnergy	0,87538	0,520175	0,536991	n.s.
wavelet-LHL_glszm_LargeAreaHighGrayLevelEmphasis	0,86592	0,52688	0,543406	n.s.
log-sigma-4-0-mm-3D_glszm_SizeZoneNonUniformityNormalized	0,862246	0,529468	0,545091	n.s.
wavelet-LHH_gldm_DependenceVariance	0,862258	0,529489	0,545091	n.s.
wavelet-HLH_gldm_DependenceVariance	0,859143	0,531707	0,54687	n.s.
wavelet-HHH_firstorder_Mean	0,85721	0,533097	0,547796	n.s.
wavelet-HLL_glszm_GrayLevelNonUniformity	0,846465	0,540821	0,555223	n.s.
wavelet-HHH_firstorder_RootMeanSquared	0,814068	0,564446	0,578946	n.s.
wavelet-HHL_glszm_GrayLevelNonUniformity	0,807619	0,569208	0,583294	n.s.
wavelet-HLL_glszm_LargeAreaHighGrayLevelEmphasis	0,787293	0,584328	0,598241	n.s.
wavelet-HHH_glszm_SizeZoneNonUniformityNormalized	0,776507	0,592402	0,605952	n.s.
log-sigma-5-0-mm-3D_gldm_DependenceEntropy	0,747846	0,614131	0,627604	n.s.
wavelet-HLL_firstorder_Skewness	0,743969	0,61707	0,630031	n.s.
wavelet-LHH_firstorder_Kurtosis	0,735244	0,623761	0,636282	n.s.
wavelet-HHH_glszm_SmallAreaLowGrayLevelEmphasis	0,726726	0,630297	0,642363	n.s.
wavelet-HHL_firstorder_Skewness	0,68616	0,661656	0,673708	n.s.
wavelet-HLL_firstorder_Kurtosis	0,657431	0,684028	0,695854	n.s.
wavelet-LHH_firstorder_Skewness	0,655163	0,685797	0,696524	n.s.
wavelet-HHL_firstorder_Mean	0,654989	0,685933	0,696524	n.s.
wavelet-HHL_gldm_SmallDependenceLowGrayLevelEmphasis	0,632577	0,703425	0,713638	n.s.
wavelet-HLH_glcm_Idn	0,59523	0,732526	0,742488	n.s.
log-sigma-3-0-mm-3D_glcm_ClusterShade	0,592155	0,734919	0,744239	n.s.
wavelet-HHL_glcm_Correlation	0,579364	0,744831	0,753594	n.s.
wavelet-HHH_gldm_SmallDependenceLowGrayLevelEmphasis	0,573651	0,749245	0,757374	n.s.
wavelet-LHH_glszm_SmallAreaLowGrayLevelEmphasis	0,525528	0,786021	0,793832	n.s.
wavelet-LHH_glcm_Idn	0,472467	0,825277	0,832726	n.s.
wavelet-HLH_glszm_SmallAreaLowGrayLevelEmphasis	0,464137	0,831279	0,837503	n.s.
wavelet-HHL_firstorder_Median	0,463812	0,831511	0,837503	n.s.
original_gldm_SmallDependenceLowGrayLevelEmphasis	0,453441	0,838875	0,84416	n.s.
wavelet-LHL_glcm_ClusterShade	0,426457	0,857625	0,862253	n.s.
log-sigma-4-0-mm-3D_glszm_SmallAreaLowGrayLevelEmphasis	0,425408	0,858915	0,862774	n.s.
wavelet-HLH_firstorder_Median	0,40298	0,873336	0,876472	n.s.
log-sigma-2-0-mm-3D_firstorder_Skewness	0,250497	0,956703	0,959277	n.s.
log-sigma-2-0-mm-3D_glcm_ClusterShade	0,245664	0,958693	0,960412	n.s.
wavelet-LLH_glcm_Idmn	0,185676	0,979414	0,98029	n.s.
wavelet-LHH_glcm_ClusterShade	0,139246	0,990285	0,990285	n.s.
