# Supplementary material for: Contrast Agent Dynamics Determine Radiomics Profiles in Oncologic Imaging
Source: Cancers (Basel). 2024 Apr 16;16(8):1519. doi: 10.3390/cancers16081519 (PMC11049400; doi:10.3390/cancers16081519)
Supplement: Supplementary file 1 [file cancers-16-01519-s001.zip › Table S5.pdf]

Table S5: Linear mixed model p values and cluster membership of all MRI radiomics features for NSCLC tumors

Feature	F value	p value	FDR	Cluster
log-sigma-2-0-mm-3D_glcm_InverseVariance	64,7362	4,1E-45	4,59E-42	3
wavelet-LHL_gldm_SmallDependenceEmphasis	60,4905	3,79E-43	2,12E-40	1
wavelet-HHL_glrlm_RunEntropy	59,5331	1,2E-42	4,48E-40	1
wavelet-LHL_glszm_ZonePercentage	58,2808	4,31E-42	1,21E-39	1
wavelet-HHL_glszm_ZonePercentage	57,5298	1,01E-41	2,25E-39	1
log-sigma-2-0-mm-3D_glszm_ZonePercentage	56,5264	3,18E-41	4E-39	1
log-sigma-2-0-mm-3D_glcm_Id	56,7525	2,4E-41	4E-39	3
log-sigma-2-0-mm-3D_glcm_DifferenceEntropy	56,4929	3,22E-41	4E-39	1
original_glszm_ZonePercentage	56,5546	3,05E-41	4E-39	1
wavelet-HHL_firstorder_Entropy	56,1841	4,88E-41	5,46E-39	1
wavelet-HHL_glcm_InverseVariance	55,6432	8,83E-41	8,97E-39	3
log-sigma-2-0-mm-3D_firstorder_Entropy	55,1849	1,42E-40	1,26E-38	1
log-sigma-2-0-mm-3D_glcm_Idm	55,1681	1,47E-40	1,26E-38	3
wavelet-HHL_glcm_DifferenceEntropy	55,158	1,59E-40	1,27E-38	1
wavelet-HLH_glcm_Idm	53,9813	6,07E-40	4,52E-38	3
wavelet-HLH_glcm_Id	53,8051	7,45E-40	5,2E-38	3
wavelet-HLL_glszm_ZonePercentage	53,6838	8,34E-40	5,49E-38	1
wavelet-HLH_firstorder_Entropy	52,7478	2,48E-39	1,5E-37	1
wavelet-HHL_firstorder_10Percentile	52,7332	2,56E-39	1,5E-37	3
wavelet-HHL_glcm_SumEntropy	52,7222	2,74E-39	1,53E-37	1
log-sigma-2-0-mm-3D_gldm_SmallDependenceEmphasis	52,3147	4,2E-39	2,24E-37	1
wavelet-LHH_glcm_Idm	51,761	8,02E-39	4,08E-37	3
wavelet-HLH_glcm_SumEntropy	51,6075	9,51E-39	4,62E-37	1
wavelet-HLH_glrlm_RunLengthNonUniformityNormalized	51,6115	1,04E-38	4,82E-37	1
log-sigma-2-0-mm-3D_glrlm_RunEntropy	51,522	1,12E-38	4,84E-37	1
original_glcm_DifferenceEntropy	51,4432	1,2E-38	4,84E-37	1
wavelet-LHL_glrlm_RunEntropy	51,5281	1,2E-38	4,84E-37	1
wavelet-LHH_glcm_Id	51,4161	1,21E-38	4,84E-37	3
log-sigma-3-0-mm-3D_glcm_InverseVariance	51,2175	1,56E-38	6E-37	3
wavelet-HLH_glcm_JointEntropy	51,1512	1,64E-38	6,13E-37	1
wavelet-HLL_gldm_SmallDependenceEmphasis	51,0479	1,9E-38	6,85E-37	1
wavelet-HHL_gldm_SmallDependenceEmphasis	50,9725	2,06E-38	7,2E-37	1
log-sigma-3-0-mm-3D_glcm_Id	50,9572	2,22E-38	7,51E-37	3
original_gldm_SmallDependenceEmphasis	50,8618	2,35E-38	7,73E-37	1
wavelet-LHL_glcm_DifferenceEntropy	50,6466	3,37E-38	1,08E-36	1
wavelet-LHH_firstorder_Entropy	50,5192	3,57E-38	1,11E-36	1
wavelet-HHL_firstorder_RobustMeanAbsoluteDeviation	50,3671	4,33E-38	1,31E-36	1
log-sigma-2-0-mm-3D_glcm_SumEntropy	50,313	4,54E-38	1,33E-36	1
log-sigma-3-0-mm-3D_glcm_Idm	50,3554	4,63E-38	1,33E-36	3
log-sigma-3-0-mm-3D_glcm_DifferenceEntropy	50,1951	5,18E-38	1,45E-36	1
wavelet-LHL_firstorder_Entropy	50,1789	5,94E-38	1,61E-36	1
wavelet-LHH_glcm_JointEntropy	50,0902	6,04E-38	1,61E-36	1
wavelet-HLH_glcm_DifferenceEntropy	49,8568	8,05E-38	2,05E-36	1

wavelet-HHL_firstorder_InterquartileRange	49,8548	8,07E-38	2,05E-36	1
wavelet-HLH_glrlm_RunPercentage	49,6418	1,13E-37	2,74E-36	1
wavelet-LHH_glcm_MaximumProbability	49,5972	1,1E-37	2,74E-36	3
wavelet-LHH_glcm_SumEntropy	49,4916	1,26E-37	2,99E-36	1
wavelet-HHL_firstorder_MeanAbsoluteDeviation	49,3502	1,51E-37	3,52E-36	1
wavelet-HHL_glcm_Id	49,3279	1,57E-37	3,59E-36	3
wavelet-LHH_firstorder_Uniformity	49,2159	1,75E-37	3,9E-36	3
original_glcm_InverseVariance	49,083	2,04E-37	4,47E-36	3
wavelet-HHH_firstorder_Uniformity	49,0623	2,12E-37	4,57E-36	3
wavelet-LHL_gldm_DependenceNonUniformityNormalized	49,0171	2,24E-37	4,73E-36	1
wavelet-HLH_firstorder_Uniformity	48,9619	2,45E-37	5,07E-36	3
wavelet-HHH_glrlm_GrayLevelNonUniformityNormalized	48,7804	3,01E-37	6,13E-36	3
original_glcm_Id	48,7162	3,21E-37	6,41E-36	3
wavelet-HLL_glrlm_RunEntropy	48,4219	4,87E-37	9,55E-36	1
wavelet-LHL_glcm_InverseVariance	48,341	5,42E-37	1,05E-35	3
log-sigma-3-0-mm-3D_firstorder_Entropy	48,2032	5,98E-37	1,13E-35	1
wavelet-LHL_glcm_Id	48,117	7,07E-37	1,32E-35	3
log-sigma-3-0-mm-3D_glszm_ZonePercentage	48,0077	8,14E-37	1,49E-35	1
wavelet-HHH_glcm_JointEnergy	47,9124	8,86E-37	1,57E-35	3
wavelet-HLL_firstorder_Entropy	47,9206	8,83E-37	1,57E-35	1
wavelet-LHH_firstorder_RobustMeanAbsoluteDeviation	47,7421	1,1E-36	1,92E-35	1
wavelet-HLH_glcm_MaximumProbability	47,6113	1,31E-36	2,26E-35	3
wavelet-HLH_firstorder_RobustMeanAbsoluteDeviation	47,5636	1,39E-36	2,36E-35	1
wavelet-HHH_glrlm_ShortRunEmphasis	47,526	1,48E-36	2,46E-35	1
wavelet-HLL_glcm_DifferenceEntropy	47,3914	1,72E-36	2,83E-35	1
wavelet-LHH_glcm_DifferenceEntropy	47,3444	1,8E-36	2,92E-35	1
wavelet-LLL_gldm_SmallDependenceEmphasis	47,273	2,04E-36	3,26E-35	1
wavelet-LLL_glcm_DifferenceEntropy	47,2412	2,07E-36	3,26E-35	1
log-sigma-2-0-mm-3D_gldm_DependenceNonUniformityNormalized	47,1195	2,48E-36	3,85E-35	1
wavelet-LHH_glrlm_RunLengthNonUniformityNormalized	47,0906	2,55E-36	3,9E-35	1
wavelet-HLH_firstorder_InterquartileRange	47,0673	2,6E-36	3,93E-35	1
wavelet-HLL_glcm_SumEntropy	47,0193	2,76E-36	4,11E-35	1
wavelet-LLL_glszm_SizeZoneNonUniformityNormalized	46,9114	3,15E-36	4,63E-35	1
wavelet-HHL_glcm_DifferenceAverage	46,8846	3,27E-36	4,74E-35	1
wavelet-LLL_gldm_DependenceNonUniformityNormalized	46,7444	3,99E-36	5,72E-35	1
original_firstorder_Entropy	46,4696	5,72E-36	8,1E-35	1
wavelet-HLH_firstorder_MeanAbsoluteDeviation	46,3863	6,12E-36	8,56E-35	1
log-sigma-3-0-mm-3D_gldm_SmallDependenceEmphasis	46,2614	7,35E-36	1E-34	1
original_glcm_DifferenceAverage	46,2343	7,32E-36	1E-34	1
wavelet-HHH_glrlm_RunLengthNonUniformityNormalized	46,0953	8,99E-36	1,21E-34	1
wavelet-HLL_glcm_Id	46,0429	9,36E-36	1,25E-34	3
wavelet-LHH_firstorder_MeanAbsoluteDeviation	45,9853	1,01E-35	1,33E-34	1
wavelet-HHL_firstorder_90Percentile	45,959	1,05E-35	1,37E-34	1
wavelet-HHH_glcm_SumEntropy	45,7962	1,29E-35	1,66E-34	1
wavelet-HHH_glrlm_RunPercentage	45,726	1,45E-35	1,83E-34	1
wavelet-HHH_firstorder_Entropy	45,7005	1,46E-35	1,83E-34	1
wavelet-LHL_glcm_SumEntropy	45,768	1,48E-35	1,84E-34	1

log-sigma-2-0-mm-3D_glcm_JointEntropy	45,585	1,7E-35	2,08E-34	1
wavelet-HHH_glcm_JointEntropy	45,5769	1,71E-35	2,08E-34	1
original_firstorder_90Percentile	45,4938	1,93E-35	2,32E-34	1
wavelet-LHH_firstorder_90Percentile	45,3548	2,26E-35	2,68E-34	1
log-sigma-2-0-mm-3D_glcm_DifferenceAverage	45,3138	2,39E-35	2,81E-34	1
wavelet-HLH_glrlm_ShortRunEmphasis	45,3027	2,62E-35	3,05E-34	1
log-sigma-3-0-mm-3D_glcm_SumEntropy	45,1078	3,06E-35	3,53E-34	1
wavelet-HHH_glcm_MaximumProbability	44,9756	3,73E-35	4,26E-34	3
wavelet-LHH_glrlm_RunPercentage	44,9782	3,8E-35	4,29E-34	1
log-sigma-2-0-mm-3D_firstorder_MeanAbsoluteDeviation	44,9247	3,91E-35	4,37E-34	1
wavelet-HHL_glcm_Idm	44,8711	4,36E-35	4,82E-34	3
original_glrlm_RunEntropy	44,8898	4,4E-35	4,82E-34	1
wavelet-HLH_glrlm_GrayLevelNonUniformityNormalized	44,632	5,79E-35	6,29E-34	3
log-sigma-2-0-mm-3D_glrlm_RunLengthNonUniformityNormalized	44,5879	6,34E-35	6,82E-34	1
wavelet-HHH_glcm_DifferenceEntropy	44,3121	8,8E-35	9,37E-34	1
wavelet-HLL_glcm_InverseVariance	44,2938	8,97E-35	9,38E-34	3
wavelet-LLL_firstorder_90Percentile	44,3077	8,94E-35	9,38E-34	1
wavelet-LHL_glszm_SmallAreaEmphasis	44,1199	1,13E-34	1,17E-33	1
wavelet-LLL_glszm_SmallAreaEmphasis	44,1084	1,15E-34	1,18E-33	1
wavelet-HHL_glcm_JointEntropy	44,1199	1,22E-34	1,24E-33	1
wavelet-LHH_firstorder_InterquartileRange	43,9652	1,39E-34	1,4E-33	1
wavelet-LLL_glcm_Id	43,892	1,51E-34	1,51E-33	3
wavelet-LLL_glcm_DifferenceAverage	43,7968	1,71E-34	1,69E-33	1
wavelet-HHH_glcm_Id	43,5861	2,28E-34	2,23E-33	3
log-sigma-3-0-mm-3D_glcm_JointEntropy	43,5658	2,3E-34	2,24E-33	1
wavelet-HHH_glcm_Idm	43,5661	2,34E-34	2,25E-33	3
log-sigma-3-0-mm-3D_glcm_DifferenceAverage	43,4142	2,85E-34	2,73E-33	1
wavelet-LHL_firstorder_90Percentile	43,3859	3,06E-34	2,9E-33	1
log-sigma-2-0-mm-3D_firstorder_RobustMeanAbsoluteDeviation	43,2589	3,47E-34	3,26E-33	1
original_glcm_Idm	43,1849	3,81E-34	3,55E-33	3
original_gldm_DependenceNonUniformityNormalized	43,1941	3,88E-34	3,58E-33	1
wavelet-HLL_gldm_DependenceNonUniformityNormalized	43,1766	3,98E-34	3,64E-33	1
wavelet-LHL_glcm_DifferenceAverage	43,1525	4,11E-34	3,74E-33	1
wavelet-LHH_firstorder_10Percentile	42,8912	5,73E-34	5,16E-33	3
wavelet-LHL_firstorder_MeanAbsoluteDeviation	42,8692	5,97E-34	5,34E-33	1
wavelet-LLL_glszm_ZonePercentage	42,8468	6,29E-34	5,58E-33	1
wavelet-LHH_glrlm_GrayLevelNonUniformityNormalized	42,7578	6,77E-34	5,96E-33	3
wavelet-LHL_glszm_SizeZoneNonUniformityNormalized	42,7484	6,84E-34	5,97E-33	1
wavelet-HHH_gldm_LargeDependenceEmphasis	42,7261	7,29E-34	6,32E-33	3
original_glcm_SumEntropy	42,7143	7,43E-34	6,39E-33	1
log-sigma-3-0-mm-3D_glrlm_RunEntropy	42,5849	8,94E-34	7,63E-33	1
log-sigma-2-0-mm-3D_firstorder_InterquartileRange	42,4589	1E-33	8,49E-33	1
wavelet-HLH_firstorder_90Percentile	42,4524	1,04E-33	8,76E-33	1
log-sigma-3-0-mm-3D_gldm_DependenceNonUniformityNormalized	42,4012	1,2E-33	9,97E-33	1
wavelet-LHL_glcm_Idm	42,1243	1,63E-33	1,35E-32	3
wavelet-LHL_firstorder_InterquartileRange	42,0929	1,66E-33	1,37E-32	1
wavelet-LHL_firstorder_RobustMeanAbsoluteDeviation	42,0274	1,82E-33	1,48E-32	1

wavelet-LLL_firstorder_Entropy	41,8996	2,22E-33	1,8E-32	1
wavelet-LLL_glrlm_RunEntropy	41,8144	2,5E-33	2,01E-32	1
log-sigma-3-0-mm-3D_glrlm_RunLengthNonUniformityNormalized	41,857	2,53E-33	2,02E-32	1
wavelet-HLL_firstorder_90Percentile	41,78	2,54E-33	2,02E-32	1
wavelet-HHH_firstorder_RobustMeanAbsoluteDeviation	41,5632	3,38E-33	2,66E-32	1
log-sigma-2-0-mm-3D_glrlm_GrayLevelNonUniformityNormalized	41,5118	3,54E-33	2,77E-32	3
wavelet-HLH_gldm_LargeDependenceEmphasis	41,5446	3,76E-33	2,92E-32	3
wavelet-HLH_firstorder_10Percentile	41,1155	6,23E-33	4,81E-32	3
wavelet-HHH_glrlm_LongRunEmphasis	41,0253	7,2E-33	5,52E-32	3
wavelet-LHH_glcm_DifferenceAverage	40,8985	8,32E-33	6,33E-32	1
log-sigma-3-0-mm-3D_glrlm_GrayLevelNonUniformityNormalized	40,7751	9,57E-33	7,23E-32	3
log-sigma-3-0-mm-3D_firstorder_MeanAbsoluteDeviation	40,7018	1,06E-32	7,98E-32	1
wavelet-HLL_glcm_Idm	40,4696	1,49E-32	1,11E-31	3
wavelet-LHH_glrlm_ShortRunEmphasis	39,8662	3,5E-32	2,59E-31	1
wavelet-HHH_firstorder_InterquartileRange	39,7857	3,82E-32	2,81E-31	1
log-sigma-4-0-mm-3D_glcm_InverseVariance	39,6072	4,87E-32	3,56E-31	3
wavelet-HHH_firstorder_MeanAbsoluteDeviation	39,5928	5E-32	3,63E-31	1
log-sigma-3-0-mm-3D_firstorder_RobustMeanAbsoluteDeviation	39,519	5,45E-32	3,93E-31	1
wavelet-LLH_glcm_InverseVariance	39,4413	6,2E-32	4,44E-31	3
wavelet-HLH_glcm_DifferenceAverage	39,4104	6,53E-32	4,65E-31	1
log-sigma-4-0-mm-3D_glcm_DifferenceEntropy	39,342	7,01E-32	4,96E-31	1
log-sigma-4-0-mm-3D_glcm_Id	39,2914	7,65E-32	5,38E-31	3
log-sigma-4-0-mm-3D_glcm_Idm	39,2651	7,95E-32	5,56E-31	3
wavelet-LLH_glcm_DifferenceEntropy	39,0967	9,65E-32	6,7E-31	1
wavelet-HLH_glrlm_RunEntropy	39,1382	9,75E-32	6,73E-31	1
log-sigma-3-0-mm-3D_firstorder_Uniformity	38,8733	1,34E-31	9,19E-31	3
log-sigma-4-0-mm-3D_firstorder_Entropy	38,8643	1,37E-31	9,35E-31	1
original_firstorder_MeanAbsoluteDeviation	38,6901	1,81E-31	1,22E-30	1
wavelet-HHH_glrlm_RunVariance	38,6181	1,99E-31	1,34E-30	3
wavelet-HLH_glcm_JointEnergy	38,6038	2,06E-31	1,38E-30	3
wavelet-HHL_gldm_DependenceNonUniformityNormalized	38,4458	2,46E-31	1,64E-30	1
log-sigma-3-0-mm-3D_firstorder_InterquartileRange	38,2556	3,2E-31	2,11E-30	1
log-sigma-2-0-mm-3D_firstorder_Uniformity	38,2421	3,24E-31	2,13E-30	3
wavelet-HLL_firstorder_MeanAbsoluteDeviation	38,2006	3,49E-31	2,28E-30	1
log-sigma-2-0-mm-3D_glszm_GrayLevelNonUniformityNormalized	38,1936	3,95E-31	2,57E-30	3
original_firstorder_InterquartileRange	38,0688	4,32E-31	2,79E-30	1
wavelet-LLH_glcm_Id	38,0368	4,48E-31	2,88E-30	3
wavelet-HHH_firstorder_90Percentile	37,9077	5,29E-31	3,38E-30	1
original_firstorder_RobustMeanAbsoluteDeviation	37,8712	5,71E-31	3,63E-30	1
log-sigma-4-0-mm-3D_glszm_ZonePercentage	37,7463	6,68E-31	4,22E-30	1
wavelet-LLL_glcm_InverseVariance	37,6306	7,8E-31	4,9E-30	3
log-sigma-2-0-mm-3D_glrlm_RunPercentage	37,5357	9,32E-31	5,81E-30	1
wavelet-LHH_gldm_LargeDependenceEmphasis	37,5266	9,35E-31	5,81E-30	3
log-sigma-3-0-mm-3D_glrlm_RunPercentage	37,5703	9,53E-31	5,89E-30	1
log-sigma-2-0-mm-3D_glszm_ZoneEntropy	37,3301	1,25E-30	7,66E-30	1
wavelet-LLH_glcm_Idm	37,2823	1,31E-30	8,02E-30	3
log-sigma-2-0-mm-3D_glrlm_ShortRunEmphasis	37,2843	1,32E-30	8,02E-30	1

wavelet-HHL_glrlm_GrayLevelNonUniformityNormalized	37,2548	1,4E-30	8,47E-30	3
wavelet-HLH_glrlm_RunVariance	37,2157	1,51E-30	9,07E-30	3
log-sigma-4-0-mm-3D_glcm_DifferenceAverage	37,0685	1,73E-30	1,03E-29	1
wavelet-LLH_firstorder_Entropy	36,6532	3,1E-30	1,84E-29	1
log-sigma-4-0-mm-3D_glcm_SumEntropy	36,6473	3,2E-30	1,89E-29	1
log-sigma-3-0-mm-3D_glrlm_ShortRunEmphasis	36,6784	3,37E-30	1,98E-29	1
wavelet-LLL_glcm_Idm	36,6036	3,4E-30	1,99E-29	3
wavelet-HLL_firstorder_RobustMeanAbsoluteDeviation	36,5894	3,47E-30	2,02E-29	1
wavelet-HLL_glcm_DifferenceAverage	36,5418	3,72E-30	2,15E-29	1
log-sigma-4-0-mm-3D_gldm_SmallDependenceEmphasis	36,3952	4,61E-30	2,66E-29	1
wavelet-LHH_glcm_JointEnergy	36,376	4,7E-30	2,69E-29	3
log-sigma-4-0-mm-3D_glcm_JointEntropy	36,2524	5,64E-30	3,22E-29	1
log-sigma-3-0-mm-3D_glszm_SizeZoneNonUniformityNormalized	36,1931	6,11E-30	3,47E-29	1
wavelet-HLL_firstorder_InterquartileRange	36,0847	7,22E-30	4,08E-29	1
wavelet-HLH_glrlm_LongRunEmphasis	35,938	9,53E-30	5,35E-29	3
wavelet-LLL_glcm_SumEntropy	35,7522	1,19E-29	6,65E-29	1
wavelet-HHH_firstorder_10Percentile	35,5595	1,55E-29	8,63E-29	3
wavelet-HLL_glcm_JointEntropy	35,4647	1,83E-29	1,01E-28	1
original_glcm_JointEntropy	35,4547	1,85E-29	1,02E-28	1
wavelet-LLH_glcm_DifferenceAverage	35,3794	2,01E-29	1,1E-28	1
wavelet-LLL_firstorder_MeanAbsoluteDeviation	35,2506	2,5E-29	1,36E-28	1
wavelet-LHL_glcm_JointEntropy	35,0682	3,39E-29	1,84E-28	1
wavelet-LLH_firstorder_MeanAbsoluteDeviation	34,9685	3,69E-29	1,99E-28	1
wavelet-LHH_gldm_DependenceVariance	34,9132	4,1E-29	2,2E-28	3
wavelet-LLH_glszm_ZonePercentage	34,837	4,57E-29	2,44E-28	1
wavelet-HHL_glrlm_RunLengthNonUniformityNormalized	34,7764	4,83E-29	2,57E-28	1
original_glszm_GrayLevelNonUniformityNormalized	34,7897	5,05E-29	2,67E-28	3
log-sigma-5-0-mm-3D_glcm_DifferenceEntropy	34,7622	5,07E-29	2,67E-28	1
wavelet-LHH_glrlm_RunEntropy	34,7718	5,19E-29	2,72E-28	1
wavelet-LLH_gldm_SmallDependenceEmphasis	34,7215	5,38E-29	2,81E-28	1
log-sigma-2-0-mm-3D_firstorder_90Percentile	34,5931	6,47E-29	3,36E-28	1
wavelet-HHL_gldm_DependenceEntropy	34,579	6,75E-29	3,49E-28	1
wavelet-HLL_glrlm_RunLengthNonUniformityNormalized	34,5002	7,42E-29	3,82E-28	1
original_glrlm_RunLengthNonUniformityNormalized	34,3743	8,98E-29	4,61E-28	1
wavelet-LLL_firstorder_RobustMeanAbsoluteDeviation	34,2367	1,11E-28	5,68E-28	1
wavelet-HHL_firstorder_Uniformity	34,0257	1,54E-28	7,85E-28	3
log-sigma-4-0-mm-3D_glrlm_RunEntropy	34,0028	1,59E-28	8,04E-28	1
wavelet-LLH_glcm_SumEntropy	33,9273	1,7E-28	8,54E-28	1
log-sigma-4-0-mm-3D_glrlm_RunLengthNonUniformityNormalized	33,9172	1,85E-28	9,21E-28	1
wavelet-LLL_firstorder_RootMeanSquared	33,8806	1,84E-28	9,21E-28	1
wavelet-HHH_glrlm_RunEntropy	33,7911	2,18E-28	1,08E-27	1
wavelet-LLL_glszm_GrayLevelNonUniformityNormalized	33,6327	2,77E-28	1,37E-27	3
wavelet-LHH_glrlm_RunVariance	33,5789	2,99E-28	1,47E-27	3
wavelet-HHL_glszm_ZoneEntropy	33,5413	3,31E-28	1,62E-27	1
wavelet-LHL_glrlm_RunLengthNonUniformityNormalized	33,4099	3,7E-28	1,81E-27	1
wavelet-LLL_firstorder_InterquartileRange	33,2812	4,64E-28	2,26E-27	1
wavelet-HHH_gldm_DependenceEntropy	33,2584	4,77E-28	2,31E-27	1

log-sigma-2-0-mm-3D_firstorder_10Percentile	33,2184	5,06E-28	2,44E-27	3
wavelet-LHL_glszm_GrayLevelNonUniformityNormalized	33,1987	5,75E-28	2,76E-27	3
wavelet-HHH_glszm_ZoneEntropy	32,9691	7,6E-28	3,63E-27	1
wavelet-HLH_gldm_DependenceVariance	32,969	7,8E-28	3,71E-27	3
wavelet-HHH_glcm_DifferenceAverage	32,8475	8,8E-28	4,17E-27	1
log-sigma-3-0-mm-3D_glszm_SmallAreaEmphasis	32,8279	9,23E-28	4,36E-27	1
wavelet-LHL_firstorder_10Percentile	32,8055	9,37E-28	4,4E-27	3
wavelet-LLH_glrlm_RunLengthNonUniformityNormalized	32,8007	9,88E-28	4,62E-27	1
wavelet-LLH_glcm_JointEntropy	32,6565	1,15E-27	5,35E-27	1
log-sigma-5-0-mm-3D_glcm_Idm	32,5586	1,33E-27	6,19E-27	3
original_glrlm_GrayLevelNonUniformityNormalized	32,5282	1,44E-27	6,67E-27	3
log-sigma-5-0-mm-3D_glcm_Id	32,2826	2,03E-27	9,36E-27	3
wavelet-LLH_firstorder_RobustMeanAbsoluteDeviation	32,2493	2,23E-27	1,02E-26	1
log-sigma-4-0-mm-3D_gldm_DependenceNonUniformityNormalized	32,014	3,31E-27	1,51E-26	1
wavelet-LLH_firstorder_InterquartileRange	31,961	3,49E-27	1,59E-26	1
log-sigma-4-0-mm-3D_firstorder_MeanAbsoluteDeviation	31,9299	3,54E-27	1,6E-26	1
original_firstorder_RootMeanSquared	31,9065	3,68E-27	1,66E-26	1
wavelet-HLH_gldm_SmallDependenceEmphasis	31,864	3,99E-27	1,79E-26	1
original_glszm_HighGrayLevelZoneEmphasis	31,8018	4,77E-27	2,13E-26	1
wavelet-LHH_glrlm_LongRunEmphasis	31,6186	5,91E-27	2,63E-26	3
wavelet-HLH_glszm_GrayLevelNonUniformityNormalized	31,5812	5,97E-27	2,65E-26	3
original_firstorder_Uniformity	31,5933	6,03E-27	2,67E-26	3
wavelet-LLH_glrlm_RunEntropy	31,5535	6,39E-27	2,81E-26	1
wavelet-HHL_glszm_GrayLevelNonUniformityNormalized	31,4784	8,08E-27	3,54E-26	3
wavelet-HLH_glszm_ZoneEntropy	31,3554	8,72E-27	3,81E-26	1
wavelet-HLL_glrlm_GrayLevelNonUniformityNormalized	31,3339	8,91E-27	3,88E-26	3
log-sigma-5-0-mm-3D_glcm_JointEntropy	31,341	9,09E-27	3,94E-26	1
original_glszm_SmallAreaHighGrayLevelEmphasis	31,1542	1,31E-26	5,65E-26	1
log-sigma-4-0-mm-3D_glrlm_RunPercentage	31,0682	1,44E-26	6,19E-26	1
wavelet-LLH_gldm_DependenceNonUniformityNormalized	30,9595	1,65E-26	7,08E-26	1
original_glcm_JointAverage	30,9956	1,66E-26	7,1E-26	1
wavelet-HLL_glszm_SmallAreaEmphasis	30,7116	2,33E-26	9,92E-26	1
wavelet-HLH_gldm_DependenceEntropy	30,6895	2,5E-26	1,06E-25	1
log-sigma-5-0-mm-3D_firstorder_Entropy	30,6839	2,54E-26	1,07E-25	1
wavelet-LLL_glrlm_RunLengthNonUniformityNormalized	30,5802	3,02E-26	1,27E-25	1
wavelet-HLL_glszm_GrayLevelNonUniformityNormalized	30,551	3,09E-26	1,29E-25	3
wavelet-HLL_glrlm_ShortRunEmphasis	30,4952	3,33E-26	1,39E-25	1
wavelet-HLL_gldm_DependenceEntropy	30,4922	3,39E-26	1,41E-25	1
wavelet-HHL_glrlm_RunPercentage	30,4076	3,75E-26	1,55E-25	1
wavelet-HLL_firstorder_10Percentile	30,3963	3,85E-26	1,59E-25	3
wavelet-LLL_firstorder_Mean	30,382	3,93E-26	1,62E-25	1
wavelet-LHL_gldm_DependenceEntropy	30,3442	4,31E-26	1,76E-25	1
original_glrlm_ShortRunHighGrayLevelEmphasis	30,3177	4,8E-26	1,96E-25	1
wavelet-LHL_glrlm_GrayLevelNonUniformityNormalized	30,2822	4,99E-26	2,03E-25	3
wavelet-LLH_firstorder_90Percentile	30,2075	5,17E-26	2,1E-25	1
wavelet-LLL_gldm_SmallDependenceHighGrayLevelEmphasis	30,2577	5,19E-26	2,1E-25	1
wavelet-LHH_gldm_DependenceEntropy	30,2204	5,22E-26	2,1E-25	1

original_gldm_SmallDependenceHighGrayLevelEmphasis	30,2411	5,37E-26	2,15E-25	1
wavelet-HLL_glrlm_RunPercentage	30,1266	5,97E-26	2,38E-25	1
original_glrlm_RunPercentage	30,1132	6,14E-26	2,44E-25	1
original_firstorder_Mean	30,0182	6,98E-26	2,77E-25	1
wavelet-LHH_firstorder_Variance	30,0141	7,08E-26	2,8E-25	1
wavelet-HHL_glrlm_ShortRunEmphasis	29,8326	9,28E-26	3,65E-25	1
wavelet-LLL_glszm_SmallAreaHighGrayLevelEmphasis	29,8359	1,01E-25	3,95E-25	1
original_glrlm_ShortRunEmphasis	29,7366	1,11E-25	4,31E-25	1
wavelet-LHH_glrlm_GrayLevelVariance	29,7336	1,1E-25	4,31E-25	1
original_glrlm_HighGrayLevelRunEmphasis	29,7548	1,16E-25	4,52E-25	1
wavelet-LLL_glrlm_GrayLevelNonUniformityNormalized	29,676	1,22E-25	4,71E-25	3
log-sigma-5-0-mm-3D_gldm_SmallDependenceEmphasis	29,6599	1,23E-25	4,75E-25	1
wavelet-HLL_firstorder_Uniformity	29,6004	1,35E-25	5,2E-25	3
log-sigma-4-0-mm-3D_glrlm_GrayLevelNonUniformityNormalized	29,5855	1,38E-25	5,3E-25	3
log-sigma-4-0-mm-3D_firstorder_RobustMeanAbsoluteDeviation	29,5754	1,4E-25	5,35E-25	1
log-sigma-4-0-mm-3D_glrlm_ShortRunEmphasis	29,6035	1,42E-25	5,41E-25	1
original_gldm_HighGrayLevelEmphasis	29,5743	1,55E-25	5,87E-25	1
log-sigma-5-0-mm-3D_glcm_SumEntropy	29,4683	1,71E-25	6,47E-25	1
wavelet-LLH_firstorder_10Percentile	29,4632	1,72E-25	6,47E-25	3
wavelet-HLL_glszm_SizeZoneNonUniformityNormalized	29,425	1,79E-25	6,7E-25	1
wavelet-LHH_glcm_ClusterTendency	29,417	1,83E-25	6,85E-25	1
wavelet-LHH_gldm_GrayLevelVariance	29,388	1,91E-25	7,13E-25	1
wavelet-LLH_glrlm_RunPercentage	29,3322	2,19E-25	8,13E-25	1
wavelet-LLL_firstorder_Uniformity	29,2788	2,29E-25	8,48E-25	3
wavelet-LHL_glrlm_RunPercentage	29,2609	2,31E-25	8,51E-25	1
log-sigma-5-0-mm-3D_glcm_DifferenceAverage	29,2262	2,44E-25	8,96E-25	1
log-sigma-2-0-mm-3D_firstorder_Maximum	29,1766	2,81E-25	1,03E-24	1
wavelet-LLH_glrlm_ShortRunEmphasis	28,9226	4,2E-25	1,54E-24	1
log-sigma-4-0-mm-3D_firstorder_Uniformity	28,8849	4,25E-25	1,55E-24	3
log-sigma-4-0-mm-3D_firstorder_InterquartileRange	28,8125	4,75E-25	1,72E-24	1
wavelet-LLL_glrlm_RunPercentage	28,8439	4,74E-25	1,72E-24	1
log-sigma-3-0-mm-3D_glcm_Contrast	28,807	4,83E-25	1,74E-24	1
wavelet-LHL_glrlm_ShortRunEmphasis	28,766	5,1E-25	1,83E-24	1
log-sigma-5-0-mm-3D_glrlm_RunLengthNonUniformityNormalized	28,6084	6,71E-25	2,4E-24	1
log-sigma-2-0-mm-3D_glrlm_GrayLevelVariance	28,5771	6,98E-25	2,49E-24	1
wavelet-LHL_firstorder_Uniformity	28,601	7,19E-25	2,56E-24	3
log-sigma-2-0-mm-3D_firstorder_Variance	28,5247	7,59E-25	2,69E-24	1
wavelet-LHH_gldm_SmallDependenceEmphasis	28,4672	8,4E-25	2,97E-24	1
log-sigma-2-0-mm-3D_gldm_GrayLevelVariance	28,4469	8,6E-25	3,03E-24	1
wavelet-LLL_glszm_HighGrayLevelZoneEmphasis	28,4348	9,38E-25	3,3E-24	1
wavelet-LLL_glrlm_ShortRunEmphasis	28,4079	9,56E-25	3,35E-24	1
wavelet-LHH_glcm_SumSquares	28,343	1,03E-24	3,59E-24	1
wavelet-LHH_gldm_DependenceNonUniformityNormalized	28,3101	1,09E-24	3,81E-24	1
wavelet-LLH_gldm_GrayLevelVariance	28,1914	1,31E-24	4,55E-24	1
log-sigma-2-0-mm-3D_glszm_GrayLevelVariance	28,2065	1,33E-24	4,6E-24	1
wavelet-LLH_firstorder_Variance	28,1795	1,33E-24	4,6E-24	1
wavelet-HLH_glszm_ZonePercentage	28,1299	1,47E-24	5,04E-24	1

wavelet-LLH_glrlm_GrayLevelVariance	28,0369	1,68E-24	5,76E-24	1
wavelet-LLH_glrlm_GrayLevelNonUniformityNormalized	27,9913	1,8E-24	6,15E-24	3
log-sigma-2-0-mm-3D_glszm_SizeZoneNonUniformityNormalized	27,9029	2,07E-24	7,07E-24	1
log-sigma-2-0-mm-3D_glszm_SmallAreaEmphasis	27,9035	2,08E-24	7,07E-24	1
original_glcm_Contrast	27,8947	2,12E-24	7,19E-24	1
original_glszm_SizeZoneNonUniformityNormalized	27,8867	2,15E-24	7,25E-24	1
original_glszm_SmallAreaEmphasis	27,8595	2,29E-24	7,72E-24	1
log-sigma-3-0-mm-3D_glszm_GrayLevelNonUniformityNormalized	27,8504	2,34E-24	7,86E-24	3
log-sigma-4-0-mm-3D_glszm_ZoneEntropy	27,7965	2,56E-24	8,58E-24	1
wavelet-LLL_glrlm_ShortRunHighGrayLevelEmphasis	27,6974	3,11E-24	1,04E-23	1
wavelet-LLL_glcm_JointAverage	27,6057	3,6E-24	1,2E-23	1
wavelet-HHH_gldm_SmallDependenceEmphasis	27,5727	3,63E-24	1,2E-23	1
wavelet-HLH_glcm_InverseVariance	27,4854	4,17E-24	1,38E-23	3
log-sigma-5-0-mm-3D_glrlm_RunPercentage	27,49	4,21E-24	1,39E-23	1
wavelet-LLL_glrlm_HighGrayLevelRunEmphasis	27,3948	5,09E-24	1,68E-23	1
log-sigma-5-0-mm-3D_glszm_ZoneEntropy	27,3673	5,3E-24	1,74E-23	1
wavelet-LLL_glcm_Contrast	27,2975	5,64E-24	1,84E-23	1
wavelet-LLL_gldm_HighGrayLevelEmphasis	27,302	5,93E-24	1,93E-23	1
wavelet-HLL_glszm_ZoneEntropy	27,2409	6,33E-24	2,06E-23	1
log-sigma-5-0-mm-3D_glszm_ZonePercentage	27,2226	6,39E-24	2,07E-23	1
wavelet-LHH_glszm_GrayLevelNonUniformityNormalized	27,0687	8,15E-24	2,63E-23	3
log-sigma-3-0-mm-3D_gldm_GrayLevelVariance	27,0247	8,76E-24	2,82E-23	1
log-sigma-3-0-mm-3D_firstorder_Variance	27,0123	8,94E-24	2,87E-23	1
wavelet-LLL_firstorder_Median	26,9237	1,04E-23	3,34E-23	1
wavelet-LLL_glszm_ZoneEntropy	26,906	1,09E-23	3,49E-23	1
log-sigma-3-0-mm-3D_glrlm_GrayLevelVariance	26,8758	1,12E-23	3,58E-23	1
wavelet-HLH_glszm_GrayLevelVariance	26,8698	1,16E-23	3,68E-23	1
wavelet-LLH_firstorder_Uniformity	26,6087	1,77E-23	5,62E-23	3
log-sigma-2-0-mm-3D_glcm_SumSquares	26,5332	1,99E-23	6,29E-23	1
log-sigma-2-0-mm-3D_firstorder_Range	26,5199	2,15E-23	6,77E-23	1
original_glrlm_LongRunHighGrayLevelEmphasis	26,5287	2,16E-23	6,78E-23	1
log-sigma-3-0-mm-3D_glcm_DifferenceVariance	26,4791	2,18E-23	6,83E-23	1
wavelet-LHH_glcm_Contrast	26,4647	2,25E-23	7,03E-23	1
original_firstorder_Median	26,3296	2,79E-23	8,7E-23	1
wavelet-LHH_glszm_GrayLevelVariance	26,3339	2,81E-23	8,73E-23	1
log-sigma-2-0-mm-3D_gldm_DependenceEntropy	26,3264	2,86E-23	8,83E-23	1
log-sigma-3-0-mm-3D_glcm_SumSquares	26,3124	2,86E-23	8,83E-23	1
log-sigma-2-0-mm-3D_glcm_DifferenceVariance	26,3127	2,89E-23	8,92E-23	1
wavelet-LLH_firstorder_Minimum	26,3007	2,94E-23	9,04E-23	3
log-sigma-5-0-mm-3D_firstorder_MeanAbsoluteDeviation	26,2481	3,27E-23	1E-22	1
log-sigma-2-0-mm-3D_glcm_Contrast	26,1695	3,68E-23	1,13E-22	1
original_glcm_Autocorrelation	26,207	3,71E-23	1,13E-22	1
log-sigma-3-0-mm-3D_glszm_ZoneEntropy	26,0864	4,29E-23	1,3E-22	1
log-sigma-3-0-mm-3D_firstorder_Range	26,103	4,3E-23	1,3E-22	1
wavelet-HHL_glrlm_GrayLevelVariance	25,9774	5,09E-23	1,54E-22	1
log-sigma-2-0-mm-3D_glcm_ClusterTendency	25,9614	5,17E-23	1,56E-22	1
wavelet-LHH_glcm_DifferenceVariance	25,9237	5,56E-23	1,67E-22	1

wavelet-LLL_glrlm_LongRunHighGrayLevelEmphasis	25,9514	5,56E-23	1,67E-22	1
wavelet-HHL_gldm_GrayLevelVariance	25,922	5,58E-23	1,67E-22	1
log-sigma-5-0-mm-3D_glrlm_ShortRunEmphasis	25,8092	6,74E-23	2,01E-22	1
wavelet-HHL_firstorder_Variance	25,8054	6,79E-23	2,02E-22	1
wavelet-LLH_glcm_SumSquares	25,7781	7,1E-23	2,1E-22	1
log-sigma-4-0-mm-3D_glcm_Contrast	25,6668	8,5E-23	2,51E-22	1
wavelet-HHL_glcm_ClusterTendency	25,6644	8,61E-23	2,54E-22	1
log-sigma-3-0-mm-3D_firstorder_10Percentile	25,6531	8,89E-23	2,62E-22	3
wavelet-LLH_glcm_JointAverage	25,6379	8,97E-23	2,63E-22	1
wavelet-HHL_glszm_GrayLevelVariance	25,6411	9,08E-23	2,66E-22	1
wavelet-HHL_glcm_SumSquares	25,3915	1,37E-22	3,99E-22	1
log-sigma-5-0-mm-3D_glcm_InverseVariance	25,359	1,47E-22	4,29E-22	3
log-sigma-3-0-mm-3D_glrlm_LongRunEmphasis	25,3235	1,66E-22	4,82E-22	3
log-sigma-5-0-mm-3D_glrlm_RunEntropy	25,2512	1,78E-22	5,15E-22	1
wavelet-LLL_firstorder_Maximum	25,219	1,89E-22	5,46E-22	1
wavelet-LLL_glrlm_LongRunEmphasis	25,214	1,9E-22	5,48E-22	3
wavelet-LLL_glcm_JointEntropy	25,1874	1,95E-22	5,6E-22	1
log-sigma-3-0-mm-3D_glcm_ClusterTendency	25,1553	2,01E-22	5,76E-22	1
wavelet-LLH_glcm_ClusterTendency	25,0699	2,36E-22	6,75E-22	1
wavelet-HHL_glcm_Contrast	25,0027	2,64E-22	7,54E-22	1
wavelet-HHH_glszm_ZonePercentage	24,9536	2,9E-22	8,24E-22	1
wavelet-LHL_glrlm_GrayLevelVariance	24,9356	2,97E-22	8,44E-22	1
wavelet-LHL_gldm_GrayLevelVariance	24,9024	3,15E-22	8,9E-22	1
wavelet-LHL_firstorder_Variance	24,8882	3,22E-22	9,1E-22	1
original_glszm_ZoneEntropy	24,8639	3,37E-22	9,5E-22	1
wavelet-HLH_glrlm_GrayLevelVariance	24,7791	3,88E-22	1,09E-21	1
wavelet-LHL_glcm_Contrast	24,7772	3,9E-22	1,09E-21	1
wavelet-LHH_glszm_ZonePercentage	24,7614	4E-22	1,12E-21	1
wavelet-LLL_glcm_Imc1	24,7551	4,08E-22	1,14E-21	3
wavelet-LLL_gldm_DependenceEntropy	24,6546	4,83E-22	1,34E-21	1
original_glcm_DifferenceVariance	24,5851	5,41E-22	1,5E-21	1
log-sigma-3-0-mm-3D_gldm_LargeDependenceEmphasis	24,6264	5,5E-22	1,52E-21	3
wavelet-LLL_glrlm_RunVariance	24,5088	6,35E-22	1,75E-21	3
wavelet-LHL_glszm_GrayLevelVariance	24,4248	7,22E-22	1,99E-21	1
log-sigma-3-0-mm-3D_glcm_MaximumProbability	24,3555	8,14E-22	2,24E-21	3
log-sigma-5-0-mm-3D_firstorder_RobustMeanAbsoluteDeviation	24,3336	8,35E-22	2,29E-21	1
wavelet-LHL_firstorder_Maximum	24,2001	1,08E-21	2,96E-21	1
log-sigma-3-0-mm-3D_firstorder_Maximum	24,1809	1,1E-21	3E-21	1
wavelet-HLH_gldm_GrayLevelVariance	24,0449	1,38E-21	3,74E-21	1
wavelet-HLH_firstorder_Variance	24,0295	1,41E-21	3,83E-21	1
log-sigma-4-0-mm-3D_glszm_SizeZoneNonUniformityNormalized	23,9638	1,58E-21	4,27E-21	1
original_glcm_SumSquares	23,9631	1,6E-21	4,32E-21	1
wavelet-LLL_glcm_Autocorrelation	23,9605	1,66E-21	4,46E-21	1
log-sigma-3-0-mm-3D_gldm_DependenceVariance	23,9932	1,67E-21	4,48E-21	3
log-sigma-3-0-mm-3D_glcm_JointEnergy	23,9243	1,67E-21	4,48E-21	3
wavelet-LHH_glcm_InverseVariance	23,905	1,74E-21	4,66E-21	3
original_gldm_DependenceEntropy	23,8776	1,85E-21	4,92E-21	1

wavelet-LLH_glcm_Contrast	23,8509	1,91E-21	5,09E-21	1
log-sigma-5-0-mm-3D_glrlm_GrayLevelNonUniformityNormalized	23,6926	2,56E-21	6,8E-21	3
wavelet-LHL_firstorder_Range	23,6864	2,62E-21	6,93E-21	1
log-sigma-3-0-mm-3D_glszm_GrayLevelVariance	23,628	2,94E-21	7,76E-21	1
wavelet-HHH_glszm_GrayLevelNonUniformityNormalized	23,5956	2,99E-21	7,87E-21	3
log-sigma-3-0-mm-3D_glrlm_RunVariance	23,5134	3,74E-21	9,83E-21	3
wavelet-LLL_glcm_DifferenceVariance	23,4085	4,14E-21	1,09E-20	1
wavelet-HLH_gldm_DependenceNonUniformityNormalized	23,3437	4,73E-21	1,24E-20	1
original_firstorder_Maximum	23,2459	5,63E-21	1,47E-20	1
wavelet-LHL_glcm_DifferenceVariance	23,1957	6,09E-21	1,59E-20	1
log-sigma-5-0-mm-3D_firstorder_InterquartileRange	23,1575	6,45E-21	1,68E-20	1
wavelet-HHL_glcm_Imc2	22,9621	9,12E-21	2,37E-20	1
log-sigma-2-0-mm-3D_glrlm_LongRunEmphasis	22,9477	9,53E-21	2,47E-20	3
wavelet-HHL_firstorder_Range	22,9425	9,64E-21	2,49E-20	1
wavelet-LHL_glcm_SumSquares	22,8018	1,21E-20	3,1E-20	1
wavelet-LHH_glszm_ZoneEntropy	22,7766	1,26E-20	3,25E-20	1
wavelet-LLH_glszm_GrayLevelNonUniformityNormalized	22,7175	1,39E-20	3,57E-20	3
log-sigma-5-0-mm-3D_firstorder_Uniformity	22,7201	1,4E-20	3,57E-20	3
original_firstorder_Range	22,6908	1,51E-20	3,86E-20	1
log-sigma-4-0-mm-3D_gldm_DependenceEntropy	22,648	1,59E-20	4,05E-20	1
wavelet-LHL_glszm_ZoneEntropy	22,6405	1,63E-20	4,15E-20	1
wavelet-LLL_firstorder_Range	22,5283	2,01E-20	5,1E-20	1
original_firstorder_Variance	22,4778	2,15E-20	5,44E-20	1
original_gldm_GrayLevelVariance	22,4472	2,27E-20	5,73E-20	1
wavelet-LLH_glszm_GrayLevelVariance	22,4383	2,29E-20	5,76E-20	1
original_glrlm_GrayLevelVariance	22,2869	3,02E-20	7,59E-20	1
log-sigma-2-0-mm-3D_glcm_MaximumProbability	22,2741	3,05E-20	7,65E-20	3
wavelet-LLH_firstorder_Range	22,2664	3,1E-20	7,76E-20	1
log-sigma-5-0-mm-3D_firstorder_10Percentile	22,2046	3,5E-20	8,72E-20	3
log-sigma-4-0-mm-3D_gldm_LargeDependenceEmphasis	22,1612	3,98E-20	9,9E-20	3
log-sigma-2-0-mm-3D_gldm_LargeDependenceEmphasis	21,9807	5,32E-20	1,32E-19	3
log-sigma-4-0-mm-3D_firstorder_Range	21,941	5,69E-20	1,41E-19	1
log-sigma-2-0-mm-3D_gldm_DependenceVariance	21,9011	6,26E-20	1,55E-19	3
log-sigma-4-0-mm-3D_glrlm_LongRunEmphasis	21,8526	6,86E-20	1,69E-19	3
original_glcm_ClusterTendency	21,8285	6,88E-20	1,7E-19	1
wavelet-HLH_glcm_ClusterTendency	21,7552	7,79E-20	1,91E-19	1
wavelet-HHL_glcm_DifferenceVariance	21,7488	7,88E-20	1,93E-19	1
log-sigma-3-0-mm-3D_gldm_DependenceEntropy	21,7481	7,9E-20	1,93E-19	1
original_glrlm_LongRunEmphasis	21,7353	8,1E-20	1,98E-19	3
log-sigma-4-0-mm-3D_firstorder_10Percentile	21,5747	1,08E-19	2,63E-19	3
wavelet-HHH_glrlm_GrayLevelVariance	21,4855	1,26E-19	3,06E-19	1
wavelet-LLL_glcm_SumSquares	21,4548	1,35E-19	3,26E-19	1
log-sigma-4-0-mm-3D_glszm_GrayLevelNonUniformityNormalized	21,4191	1,46E-19	3,52E-19	3
wavelet-HHL_glrlm_LongRunEmphasis	21,3907	1,47E-19	3,54E-19	3
log-sigma-4-0-mm-3D_glrlm_RunVariance	21,3931	1,57E-19	3,78E-19	3
wavelet-LHL_glrlm_LongRunEmphasis	21,1083	2,45E-19	5,9E-19	3
log-sigma-2-0-mm-3D_glcm_Imc2	21,0988	2,57E-19	6,16E-19	1

wavelet-HLL_glrlm_LongRunEmphasis	21,066	2,7E-19	6,47E-19	3
log-sigma-5-0-mm-3D_gldm_DependenceNonUniformityNormalized	20,9748	3,21E-19	7,66E-19	1
log-sigma-5-0-mm-3D_gldm_LargeDependenceEmphasis	20,8261	4,33E-19	1,03E-18	3
wavelet-HHL_glcm_JointAverage	20,7987	4,46E-19	1,06E-18	1
log-sigma-2-0-mm-3D_glrlm_RunVariance	20,7194	5,21E-19	1,24E-18	3
wavelet-HHH_firstorder_Variance	20,6465	5,77E-19	1,37E-18	1
wavelet-LLH_glszm_SizeZoneNonUniformityNormalized	20,624	5,91E-19	1,4E-18	1
wavelet-LHL_glcm_Imc2	20,5516	6,88E-19	1,62E-18	1
wavelet-HLH_glcm_SumSquares	20,5423	7,03E-19	1,65E-18	1
wavelet-HHL_firstorder_Minimum	20,4905	7,83E-19	1,84E-18	3
log-sigma-5-0-mm-3D_glszm_SmallAreaEmphasis	20,4939	8,33E-19	1,95E-18	1
wavelet-LHL_glcm_ClusterTendency	20,4111	8,9E-19	2,08E-18	1
wavelet-HHL_glrlm_RunVariance	20,3914	9,05E-19	2,11E-18	3
log-sigma-4-0-mm-3D_glcm_DifferenceVariance	20,3014	1,08E-18	2,52E-18	1
log-sigma-4-0-mm-3D_glcm_MaximumProbability	20,2987	1,1E-18	2,56E-18	3
log-sigma-5-0-mm-3D_glrlm_LongRunEmphasis	20,1647	1,45E-18	3,37E-18	3
log-sigma-5-0-mm-3D_glrlm_RunVariance	20,0613	1,77E-18	4,1E-18	3
log-sigma-4-0-mm-3D_firstorder_Variance	20,0327	1,78E-18	4,1E-18	1
log-sigma-4-0-mm-3D_gldm_GrayLevelVariance	20,0114	1,85E-18	4,26E-18	1
original_glrlm_RunVariance	20,0125	1,87E-18	4,3E-18	3
wavelet-LLL_gldm_DependenceVariance	20,0126	1,92E-18	4,41E-18	3
log-sigma-4-0-mm-3D_glcm_SumSquares	19,9748	1,98E-18	4,53E-18	1
wavelet-LLL_glcm_ClusterTendency	19,8935	2,33E-18	5,34E-18	1
wavelet-LLL_firstorder_Variance	19,8849	2,36E-18	5,37E-18	1
wavelet-LLL_gldm_GrayLevelVariance	19,8859	2,35E-18	5,37E-18	1
log-sigma-4-0-mm-3D_glrlm_GrayLevelVariance	19,8616	2,44E-18	5,55E-18	1
wavelet-HLH_glcm_Imc2	19,8585	2,47E-18	5,6E-18	1
wavelet-LHL_glrlm_RunVariance	19,8227	2,59E-18	5,87E-18	3
log-sigma-4-0-mm-3D_glcm_JointEnergy	19,8256	2,61E-18	5,89E-18	3
wavelet-LLL_glrlm_GrayLevelVariance	19,7986	2,77E-18	6,24E-18	1
wavelet-LLH_glszm_ZoneEntropy	19,78	2,86E-18	6,43E-18	1
wavelet-HLL_firstorder_Range	19,7757	2,92E-18	6,55E-18	1
wavelet-HHH_gldm_GrayLevelVariance	19,7291	3,13E-18	7E-18	1
wavelet-LLH_glrlm_LongRunEmphasis	19,6174	3,97E-18	8,89E-18	3
wavelet-LLH_gldm_LargeDependenceEmphasis	19,6095	4,05E-18	9,04E-18	3
wavelet-LLH_gldm_DependenceVariance	19,6052	4,09E-18	9,1E-18	3
wavelet-HLL_glcm_Imc2	19,5579	4,33E-18	9,62E-18	1
wavelet-HHH_glcm_ClusterTendency	19,5419	4,43E-18	9,82E-18	1
log-sigma-3-0-mm-3D_firstorder_90Percentile	19,3751	6,04E-18	1,34E-17	1
wavelet-LLL_gldm_LargeDependenceEmphasis	19,3006	7,12E-18	1,57E-17	3
wavelet-HHH_glcm_SumSquares	19,271	7,34E-18	1,62E-17	1
log-sigma-5-0-mm-3D_glcm_Contrast	19,2611	7,42E-18	1,63E-17	1
original_glszm_GrayLevelVariance	19,2394	7,89E-18	1,73E-17	1
log-sigma-4-0-mm-3D_glcm_ClusterTendency	19,1905	8,51E-18	1,87E-17	1
wavelet-HLL_glcm_JointAverage	19,1835	8,82E-18	1,93E-17	1
log-sigma-5-0-mm-3D_firstorder_Minimum	19,1607	9,23E-18	2,01E-17	3
log-sigma-5-0-mm-3D_glszm_SizeZoneNonUniformityNormalized	19,1208	9,7E-18	2,11E-17	1

wavelet-HHH_glcm_Contrast	18,9847	1,26E-17	2,73E-17	1
wavelet-HHL_glcm_MaximumProbability	18,9724	1,32E-17	2,86E-17	3
wavelet-HLH_glcm_Contrast	18,8571	1,61E-17	3,48E-17	1
wavelet-HLL_firstorder_Minimum	18,7352	2,05E-17	4,43E-17	3
original_gldm_DependenceVariance	18,6941	2,24E-17	4,83E-17	3
log-sigma-3-0-mm-3D_glcm_JointAverage	18,6054	2,65E-17	5,71E-17	1
wavelet-LLL_glszm_GrayLevelVariance	18,5688	2,77E-17	5,96E-17	1
log-sigma-4-0-mm-3D_gldm_DependenceVariance	18,532	3,11E-17	6,68E-17	3
wavelet-HLL_glrlm_RunVariance	18,5018	3,14E-17	6,73E-17	3
wavelet-HHL_gldm_LargeDependenceEmphasis	18,2787	4,71E-17	1,01E-16	3
wavelet-LLH_glcm_MaximumProbability	18,2763	4,85E-17	1,04E-16	3
wavelet-HHL_gldm_DependenceVariance	18,0974	6,71E-17	1,43E-16	3
wavelet-LLH_glrlm_RunVariance	17,982	8,66E-17	1,84E-16	3
log-sigma-4-0-mm-3D_firstorder_Minimum	17,8071	1,21E-16	2,57E-16	3
log-sigma-2-0-mm-3D_glcm_JointAverage	17,7072	1,45E-16	3,08E-16	1
original_glcm_Imc2	17,693	1,48E-16	3,12E-16	1
log-sigma-5-0-mm-3D_firstorder_Range	17,685	1,51E-16	3,19E-16	1
wavelet-HLL_gldm_LargeDependenceEmphasis	17,6661	1,54E-16	3,25E-16	3
original_gldm_LargeDependenceEmphasis	17,6416	1,63E-16	3,42E-16	3
wavelet-HLL_gldm_DependenceVariance	17,6409	1,64E-16	3,43E-16	3
wavelet-HHL_glszm_SmallAreaEmphasis	17,6201	1,67E-16	3,5E-16	1
log-sigma-5-0-mm-3D_gldm_DependenceVariance	17,6219	1,77E-16	3,7E-16	3
log-sigma-2-0-mm-3D_glcm_JointEnergy	17,5741	1,82E-16	3,79E-16	3
log-sigma-4-0-mm-3D_glszm_SmallAreaEmphasis	17,5001	2,16E-16	4,49E-16	1
wavelet-HLH_glcm_DifferenceVariance	17,4903	2,16E-16	4,49E-16	1
wavelet-HLL_glszm_GrayLevelVariance	17,4111	2,5E-16	5,2E-16	1
wavelet-HLL_glcm_MaximumProbability	17,3022	3,11E-16	6,43E-16	3
wavelet-HLL_glrlm_GrayLevelVariance	17,2922	3,14E-16	6,5E-16	1
log-sigma-3-0-mm-3D_firstorder_Minimum	17,3121	3,17E-16	6,53E-16	3
log-sigma-4-0-mm-3D_glcm_JointAverage	17,2459	3,54E-16	7,29E-16	1
wavelet-HLL_gldm_GrayLevelVariance	17,2223	3,6E-16	7,4E-16	1
wavelet-HLL_firstorder_Variance	17,2171	3,64E-16	7,46E-16	1
wavelet-HHL_glszm_SizeZoneNonUniformityNormalized	16,9792	5,78E-16	1,18E-15	1
wavelet-LLH_glcm_JointEnergy	16,9817	5,77E-16	1,18E-15	3
wavelet-HHL_firstorder_Maximum	16,9846	5,87E-16	1,2E-15	1
wavelet-LLH_glszm_SmallAreaEmphasis	16,9693	5,92E-16	1,2E-15	1
log-sigma-3-0-mm-3D_glcm_Imc2	16,8859	7E-16	1,42E-15	1
wavelet-LLH_glcm_DifferenceVariance	16,859	7,32E-16	1,48E-15	1
log-sigma-5-0-mm-3D_gldm_DependenceEntropy	16,8093	8,1E-16	1,64E-15	1
original_glcm_MaximumProbability	16,7977	8,22E-16	1,66E-15	3
log-sigma-5-0-mm-3D_glcm_JointEnergy	16,7851	8,46E-16	1,71E-15	3
wavelet-HHH_glcm_DifferenceVariance	16,7457	9,14E-16	1,84E-15	1
wavelet-LHL_gldm_LargeDependenceEmphasis	16,7239	9,42E-16	1,9E-15	3
wavelet-LHL_gldm_DependenceVariance	16,7089	9,79E-16	1,97E-15	3
wavelet-HLH_firstorder_Range	16,6999	1E-15	2,01E-15	1
wavelet-LHL_glcm_Imc1	16,6789	1,04E-15	2,08E-15	3
wavelet-LLH_gldm_SmallDependenceHighGrayLevelEmphasis	16,6329	1,14E-15	2,27E-15	1

wavelet-LLL_glcm_MaximumProbability	16,3554	1,97E-15	3,92E-15	3
log-sigma-5-0-mm-3D_glcm_SumSquares	16,2442	2,45E-15	4,87E-15	1
wavelet-LHH_glcm_Imc2	16,2231	2,55E-15	5,07E-15	1
log-sigma-2-0-mm-3D_firstorder_Minimum	16,2089	2,67E-15	5,3E-15	3
log-sigma-5-0-mm-3D_gldm_GrayLevelVariance	16,161	2,89E-15	5,71E-15	1
log-sigma-5-0-mm-3D_firstorder_Variance	16,1249	3,1E-15	6,12E-15	1
wavelet-LHL_glcm_JointAverage	16,116	3,18E-15	6,27E-15	1
log-sigma-5-0-mm-3D_glrlm_GrayLevelVariance	15,9714	4,2E-15	8,27E-15	1
log-sigma-2-0-mm-3D_gldm_SmallDependenceHighGrayLevelEmphasis	15,9147	4,79E-15	9,41E-15	1
log-sigma-5-0-mm-3D_glcm_ClusterTendency	15,816	5,71E-15	1,12E-14	1
wavelet-HLL_glcm_ClusterTendency	15,8057	5,77E-15	1,13E-14	1
log-sigma-5-0-mm-3D_glszm_GrayLevelNonUniformityNormalized	15,7966	6,03E-15	1,18E-14	3
wavelet-LHL_firstorder_Minimum	15,6871	7,43E-15	1,45E-14	3
log-sigma-5-0-mm-3D_glcm_JointAverage	15,552	9,81E-15	1,91E-14	1
wavelet-HHL_glcm_JointEnergy	15,4856	1,12E-14	2,17E-14	3
wavelet-LHH_firstorder_Maximum	15,456	1,17E-14	2,26E-14	1
wavelet-HHL_glszm_LowGrayLevelZoneEmphasis	15,4123	1,33E-14	2,59E-14	3
wavelet-HHH_glszm_LowGrayLevelZoneEmphasis	15,3706	1,38E-14	2,67E-14	3
wavelet-HLL_glcm_SumSquares	15,2537	1,73E-14	3,35E-14	1
wavelet-LHH_firstorder_Range	15,2268	1,84E-14	3,55E-14	1
wavelet-LHL_glcm_MaximumProbability	15,2062	1,96E-14	3,78E-14	3
log-sigma-5-0-mm-3D_glcm_MaximumProbability	15,0859	2,46E-14	4,73E-14	3
wavelet-HLL_glcm_Imc1	15,076	2,49E-14	4,78E-14	3
log-sigma-5-0-mm-3D_glcm_DifferenceVariance	14,9459	3,22E-14	6,17E-14	1
original_glcm_Imc1	14,8783	3,71E-14	7,1E-14	3
wavelet-LLH_gldm_DependenceEntropy	14,7403	4,9E-14	9,34E-14	1
wavelet-LLH_glrlm_ShortRunHighGrayLevelEmphasis	14,6679	5,65E-14	1,08E-13	1
wavelet-LLH_glcm_Imc2	14,5875	6,79E-14	1,29E-13	1
log-sigma-4-0-mm-3D_glszm_GrayLevelVariance	14,4472	8,96E-14	1,7E-13	1
wavelet-HLH_firstorder_Maximum	14,4368	9,06E-14	1,72E-13	1
wavelet-LHH_glcm_ClusterProminence	14,4197	9,39E-14	1,78E-13	1
wavelet-LLL_glcm_Imc2	14,2734	1,28E-13	2,41E-13	1
wavelet-HLH_glcm_JointAverage	14,2266	1,4E-13	2,63E-13	1
original_glcm_JointEnergy	14,1363	1,66E-13	3,13E-13	3
wavelet-HLL_firstorder_Maximum	14,0761	1,9E-13	3,57E-13	1
wavelet-HLL_glcm_Contrast	13,9574	2,39E-13	4,48E-13	1
wavelet-HLL_glcm_JointEnergy	13,8185	3,19E-13	5,97E-13	3
wavelet-LLH_gldm_HighGrayLevelEmphasis	13,8162	3,2E-13	5,98E-13	1
wavelet-HLH_firstorder_Minimum	13,7879	3,42E-13	6,38E-13	3
wavelet-LLH_glrlm_HighGrayLevelRunEmphasis	13,7542	3,63E-13	6,77E-13	1
wavelet-LLL_glcm_JointEnergy	13,7121	3,97E-13	7,39E-13	3
wavelet-LLH_glcm_Autocorrelation	13,6975	4,08E-13	7,58E-13	1
wavelet-LLH_firstorder_Maximum	13,4738	6,47E-13	1,2E-12	1
wavelet-HHH_glszm_GrayLevelVariance	13,4109	7,38E-13	1,37E-12	1
wavelet-HHH_glcm_Imc2	13,359	8,21E-13	1,52E-12	1
wavelet-HLL_glcm_DifferenceVariance	13,3113	9,04E-13	1,67E-12	1
wavelet-LLH_glszm_SizeZoneNonUniformity	13,2359	1,06E-12	1,95E-12	1

wavelet-HHL_gldm_SmallDependenceHighGrayLevelEmphasis	13,0518	1,57E-12	2,88E-12	1
wavelet-HLL_gldm_SmallDependenceHighGrayLevelEmphasis	12,8913	2,19E-12	4,02E-12	1
wavelet-HHL_glrlm_LongRunLowGrayLevelEmphasis	12,8877	2,26E-12	4,14E-12	3
wavelet-HHL_glszm_SmallAreaLowGrayLevelEmphasis	12,8783	2,32E-12	4,24E-12	3
wavelet-LLH_glszm_HighGrayLevelZoneEmphasis	12,7657	2,81E-12	5,14E-12	1
wavelet-HLH_glszm_SizeZoneNonUniformity	12,4165	5,85E-12	1,07E-11	1
wavelet-LLH_glszm_SmallAreaHighGrayLevelEmphasis	12,3844	6,25E-12	1,14E-11	1
log-sigma-5-0-mm-3D_gldm_DependenceNonUniformity	12,2734	7,91E-12	1,44E-11	1
log-sigma-4-0-mm-3D_firstorder_Maximum	12,2122	9,02E-12	1,64E-11	1
wavelet-HHL_glrlm_LowGrayLevelRunEmphasis	12,2146	9,14E-12	1,66E-11	3
wavelet-HHL_gldm_LowGrayLevelEmphasis	12,0903	1,19E-11	2,15E-11	3
log-sigma-3-0-mm-3D_glszm_LowGrayLevelZoneEmphasis	12,0183	1,45E-11	2,61E-11	3
log-sigma-2-0-mm-3D_glcm_Idn	11,9629	1,54E-11	2,77E-11	3
wavelet-HLL_glszm_SizeZoneNonUniformity	11,8909	1,77E-11	3,19E-11	1
log-sigma-2-0-mm-3D_glszm_LowGrayLevelZoneEmphasis	11,8183	2,12E-11	3,81E-11	3
wavelet-HHH_glrlm_GrayLevelNonUniformity	11,7892	2,2E-11	3,95E-11	3
log-sigma-3-0-mm-3D_glszm_GrayLevelNonUniformity	11,7685	2,3E-11	4,12E-11	1
log-sigma-2-0-mm-3D_glszm_SizeZoneNonUniformity	11,7533	2,37E-11	4,25E-11	1
log-sigma-3-0-mm-3D_glszm_SizeZoneNonUniformity	11,7043	2,63E-11	4,71E-11	1
log-sigma-5-0-mm-3D_glrlm_GrayLevelNonUniformity	11,6934	2,7E-11	4,81E-11	3
wavelet-LHH_gldm_SmallDependenceHighGrayLevelEmphasis	11,6799	2,77E-11	4,93E-11	1
wavelet-HHL_glrlm_ShortRunLowGrayLevelEmphasis	11,6261	3,17E-11	5,63E-11	3
wavelet-HHL_glszm_SmallAreaHighGrayLevelEmphasis	11,5201	3,92E-11	6,96E-11	1
log-sigma-4-0-mm-3D_glcm_Imc2	11,5122	3,95E-11	6,99E-11	1
original_glszm_SizeZoneNonUniformity	11,459	4,44E-11	7,86E-11	1
log-sigma-3-0-mm-3D_glcm_Idn	11,4317	4,77E-11	8,43E-11	3
wavelet-HLH_gldm_LargeDependenceLowGrayLevelEmphasis	11,3535	5,55E-11	9,79E-11	3
wavelet-HHL_glcm_Imc1	11,3428	5,69E-11	1E-10	3
wavelet-HHL_glrlm_GrayLevelNonUniformity	11,3092	6,12E-11	1,08E-10	3
wavelet-HHH_gldm_LargeDependenceLowGrayLevelEmphasis	11,2958	6,29E-11	1,1E-10	3
log-sigma-2-0-mm-3D_glrlm_LongRunLowGrayLevelEmphasis	11,2969	6,33E-11	1,11E-10	3
wavelet-HHL_glrlm_ShortRunHighGrayLevelEmphasis	11,2762	6,6E-11	1,16E-10	1
log-sigma-4-0-mm-3D_glrlm_GrayLevelNonUniformity	11,22	7,41E-11	1,29E-10	3
wavelet-LHH_glrlm_GrayLevelNonUniformity	11,1404	8,78E-11	1,53E-10	3
wavelet-HHL_glszm_SizeZoneNonUniformity	11,1199	9,18E-11	1,6E-10	1
log-sigma-4-0-mm-3D_glszm_GrayLevelNonUniformity	11,1078	9,42E-11	1,64E-10	1
wavelet-LHH_firstorder_Minimum	11,1072	9,43E-11	1,64E-10	3
wavelet-LHH_glszm_SizeZoneNonUniformity	11,1025	9,52E-11	1,65E-10	1
wavelet-HLH_glrlm_LongRunLowGrayLevelEmphasis	11,1021	9,59E-11	1,66E-10	3
original_firstorder_TotalEnergy	9,72095	9,84E-11	1,7E-10	1
original_firstorder_Energy	9,72095	9,84E-11	1,7E-10	1
wavelet-HHL_glszm_HighGrayLevelZoneEmphasis	11,0649	1,04E-10	1,79E-10	1
wavelet-HHH_glrlm_LongRunLowGrayLevelEmphasis	11,0202	1,14E-10	1,95E-10	3
wavelet-LLH_glcm_ClusterProminence	10,9276	1,14E-10	1,95E-10	1
log-sigma-4-0-mm-3D_gldm_DependenceNonUniformity	10,8897	1,51E-10	2,58E-10	1
wavelet-HHH_glrlm_RunLengthNonUniformity	10,8764	1,55E-10	2,65E-10	1
wavelet-HHL_glrlm_HighGrayLevelRunEmphasis	10,8046	1,82E-10	3,11E-10	1

wavelet-LHH_glcm_Idn	10,7836	1,9E-10	3,24E-10	3
wavelet-HHL_gldm_HighGrayLevelEmphasis	10,7834	1,9E-10	3,24E-10	1
wavelet-HLL_glszm_SmallAreaHighGrayLevelEmphasis	10,7811	1,92E-10	3,26E-10	1
wavelet-LLH_glszm_GrayLevelNonUniformity	10,7725	1,94E-10	3,29E-10	1
wavelet-HHH_gldm_GrayLevelNonUniformity	10,7698	1,95E-10	3,31E-10	3
wavelet-HHL_gldm_GrayLevelNonUniformity	10,7453	2,06E-10	3,48E-10	3
log-sigma-2-0-mm-3D_glrlm_LowGrayLevelRunEmphasis	10,7409	2,09E-10	3,53E-10	3
wavelet-LHL_glcm_JointEnergy	10,7384	2,12E-10	3,59E-10	3
wavelet-LLL_firstorder_TotalEnergy	9,34178	2,84E-10	4,79E-10	1
wavelet-LLL_firstorder_Energy	9,34178	2,84E-10	4,79E-10	1
wavelet-HLH_gldm_DependenceNonUniformity	10,5516	3,12E-10	5,25E-10	1
wavelet-LHL_glszm_SizeZoneNonUniformity	10,5503	3,13E-10	5,26E-10	1
wavelet-HLL_gldm_DependenceNonUniformity	10,5373	3,22E-10	5,4E-10	1
wavelet-HLH_glrlm_GrayLevelNonUniformity	10,5308	3,27E-10	5,47E-10	3
log-sigma-2-0-mm-3D_glrlm_ShortRunHighGrayLevelEmphasis	10,5328	3,29E-10	5,5E-10	1
log-sigma-2-0-mm-3D_glszm_SmallAreaHighGrayLevelEmphasis	10,4951	3,58E-10	5,97E-10	1
wavelet-HLL_glrlm_GrayLevelNonUniformity	10,4847	3,61E-10	6,02E-10	3
log-sigma-2-0-mm-3D_gldm_LowGrayLevelEmphasis	10,4703	3,75E-10	6,23E-10	3
wavelet-HLH_glrlm_LowGrayLevelRunEmphasis	10,3972	4,41E-10	7,32E-10	3
wavelet-HLL_glrlm_ShortRunHighGrayLevelEmphasis	10,3857	4,51E-10	7,48E-10	1
log-sigma-4-0-mm-3D_glrlm_LowGrayLevelRunEmphasis	10,3863	4,58E-10	7,59E-10	3
log-sigma-3-0-mm-3D_glrlm_GrayLevelNonUniformity	10,3341	5,01E-10	8,28E-10	3
wavelet-HLH_glszm_LowGrayLevelZoneEmphasis	10,3365	5,02E-10	8,29E-10	3
wavelet-HLL_gldm_GrayLevelNonUniformity	10,245	5,05E-10	8,33E-10	3
wavelet-HLH_gldm_LowGrayLevelEmphasis	10,281	5,67E-10	9,34E-10	3
log-sigma-4-0-mm-3D_glrlm_LongRunLowGrayLevelEmphasis	10,2864	5,69E-10	9,36E-10	3
log-sigma-3-0-mm-3D_gldm_DependenceNonUniformity	10,2649	5,82E-10	9,55E-10	1
log-sigma-3-0-mm-3D_glrlm_LongRunLowGrayLevelEmphasis	10,1916	6,97E-10	1,14E-09	3
log-sigma-2-0-mm-3D_glszm_GrayLevelNonUniformity	10,1732	7,11E-10	1,16E-09	1
wavelet-LHL_gldm_SmallDependenceHighGrayLevelEmphasis	10,1201	8E-10	1,31E-09	1
wavelet-HHL_gldm_DependenceNonUniformity	10,1147	8,07E-10	1,32E-09	1
wavelet-HHH_glcm_InverseVariance	10,1042	8,24E-10	1,34E-09	3
wavelet-HLH_glszm_SmallAreaLowGrayLevelEmphasis	10,0851	8,65E-10	1,41E-09	3
log-sigma-4-0-mm-3D_glrlm_ShortRunLowGrayLevelEmphasis	10,0851	8,8E-10	1,43E-09	3
log-sigma-2-0-mm-3D_glrlm_ShortRunLowGrayLevelEmphasis	10,0701	8,95E-10	1,45E-09	3
wavelet-HLL_gldm_HighGrayLevelEmphasis	9,99336	1,06E-09	1,72E-09	1
wavelet-HLL_glrlm_HighGrayLevelRunEmphasis	9,98888	1,07E-09	1,73E-09	1
wavelet-HLL_glszm_HighGrayLevelZoneEmphasis	9,92944	1,22E-09	1,97E-09	1
log-sigma-4-0-mm-3D_glszm_SizeZoneNonUniformity	9,90699	1,27E-09	2,05E-09	1
log-sigma-4-0-mm-3D_gldm_LowGrayLevelEmphasis	9,90956	1,29E-09	2,08E-09	3
log-sigma-2-0-mm-3D_glrlm_GrayLevelNonUniformity	9,89708	1,3E-09	2,09E-09	3
wavelet-LLL_firstorder_10Percentile	9,88772	1,32E-09	2,12E-09	2
wavelet-LLH_glrlm_LongRunLowGrayLevelEmphasis	9,87318	1,37E-09	2,2E-09	3
original_gldm_DependenceNonUniformity	9,86757	1,39E-09	2,22E-09	1
wavelet-LHH_glcm_JointAverage	9,86593	1,39E-09	2,22E-09	1
log-sigma-5-0-mm-3D_glrlm_ShortRunLowGrayLevelEmphasis	9,82341	1,55E-09	2,48E-09	3
wavelet-LLL_glszm_SizeZoneNonUniformity	9,78839	1,65E-09	2,63E-09	1

wavelet-LHH_gldm_DependenceNonUniformity	9,78097	1,67E-09	2,67E-09	1
wavelet-HHL_glszm_LargeAreaEmphasis	9,68542	1,76E-09	2,8E-09	3
log-sigma-5-0-mm-3D_gldm_GrayLevelNonUniformity	9,66335	1,84E-09	2,92E-09	3
log-sigma-3-0-mm-3D_gldm_SmallDependenceHighGrayLevelEmphasis	9,73672	1,86E-09	2,95E-09	1
log-sigma-5-0-mm-3D_glrlm_LowGrayLevelRunEmphasis	9,71904	1,96E-09	3,1E-09	3
log-sigma-5-0-mm-3D_glszm_GrayLevelNonUniformity	9,64288	2,27E-09	3,59E-09	1
wavelet-HHL_glszm_ZoneVariance	9,56897	2,28E-09	3,61E-09	3
wavelet-HHL_glcm_Autocorrelation	9,63038	2,34E-09	3,69E-09	1
wavelet-LLL_gldm_DependenceNonUniformity	9,61562	2,41E-09	3,79E-09	1
log-sigma-5-0-mm-3D_glszm_LowGrayLevelZoneEmphasis	9,62787	2,43E-09	3,82E-09	3
wavelet-HLL_glcm_Autocorrelation	9,50742	3,07E-09	4,82E-09	1
log-sigma-3-0-mm-3D_glrlm_LowGrayLevelRunEmphasis	9,50974	3,09E-09	4,85E-09	3
wavelet-HLH_glrlm_RunLengthNonUniformity	9,42819	3,64E-09	5,69E-09	1
log-sigma-4-0-mm-3D_gldm_GrayLevelNonUniformity	9,32688	3,89E-09	6,09E-09	3
log-sigma-2-0-mm-3D_gldm_DependenceNonUniformity	9,37466	4,09E-09	6,39E-09	1
wavelet-HHH_glszm_SizeZoneNonUniformity	9,36275	4,2E-09	6,54E-09	1
wavelet-HLH_glrlm_ShortRunLowGrayLevelEmphasis	9,36629	4,2E-09	6,54E-09	3
wavelet-HLL_glrlm_RunLengthNonUniformity	9,32862	4,53E-09	7,04E-09	1
original_glcm_Idn	9,32566	4,57E-09	7,1E-09	3
wavelet-HHH_glrlm_LowGrayLevelRunEmphasis	9,30913	4,74E-09	7,35E-09	3
log-sigma-2-0-mm-3D_glrlm_HighGrayLevelRunEmphasis	9,30395	4,82E-09	7,46E-09	1
log-sigma-2-0-mm-3D_gldm_HighGrayLevelEmphasis	9,30374	4,82E-09	7,46E-09	1
wavelet-HHL_glrlm_RunLengthNonUniformity	9,26916	5,16E-09	7,97E-09	1
wavelet-LLH_glszm_LowGrayLevelZoneEmphasis	9,27012	5,18E-09	7,98E-09	3
wavelet-LHL_glrlm_GrayLevelNonUniformity	9,26322	5,23E-09	8,06E-09	3
log-sigma-2-0-mm-3D_glszm_HighGrayLevelZoneEmphasis	9,26603	5,25E-09	8,07E-09	1
log-sigma-3-0-mm-3D_glrlm_ShortRunLowGrayLevelEmphasis	9,23229	5,7E-09	8,75E-09	3
log-sigma-3-0-mm-3D_gldm_LargeDependenceLowGrayLevelEmphasis	9,2251	5,75E-09	8,81E-09	3
log-sigma-2-0-mm-3D_glcm_Autocorrelation	9,19007	6,2E-09	9,49E-09	1
log-sigma-5-0-mm-3D_glrlm_RunLengthNonUniformity	9,10591	7,41E-09	1,13E-08	1
wavelet-LHL_firstorder_TotalEnergy	8,16753	7,44E-09	1,13E-08	1
wavelet-LHL_firstorder_Energy	8,16753	7,44E-09	1,13E-08	1
log-sigma-3-0-mm-3D_gldm_LowGrayLevelEmphasis	9,08434	7,89E-09	1,2E-08	3
log-sigma-3-0-mm-3D_glrlm_ShortRunHighGrayLevelEmphasis	9,07462	8,04E-09	1,22E-08	1
log-sigma-3-0-mm-3D_gldm_GrayLevelNonUniformity	8,97912	8,49E-09	1,29E-08	3
wavelet-HHH_gldm_LowGrayLevelEmphasis	8,99271	9,55E-09	1,45E-08	3
wavelet-LLL_glcm_Idn	8,9652	1,02E-08	1,54E-08	3
log-sigma-2-0-mm-3D_glcm_ClusterProminence	8,92967	1,09E-08	1,65E-08	1
wavelet-HHH_glszm_SmallAreaLowGrayLevelEmphasis	8,84411	1,14E-08	1,72E-08	3
wavelet-LLH_gldm_DependenceNonUniformity	8,90701	1,15E-08	1,74E-08	1
log-sigma-2-0-mm-3D_gldm_GrayLevelNonUniformity	8,84018	1,16E-08	1,75E-08	3
wavelet-LLH_glrlm_GrayLevelNonUniformity	8,89489	1,18E-08	1,78E-08	3
wavelet-LHH_gldm_LargeDependenceLowGrayLevelEmphasis	8,8927	1,19E-08	1,79E-08	3
wavelet-HLL_glrlm_LongRunLowGrayLevelEmphasis	8,89047	1,2E-08	1,8E-08	3
wavelet-HLH_gldm_GrayLevelNonUniformity	8,87997	1,22E-08	1,83E-08	3
wavelet-LLL_glszm_GrayLevelNonUniformity	8,87086	1,25E-08	1,87E-08	3

log-sigma-4-0-mm-3D_gldm_LargeDependenceLowGrayLevelEmphasis	8,85467	1,31E-08	1,96E-08	3
wavelet-LLL_gldm_GrayLevelNonUniformity	8,77423	1,35E-08	2,01E-08	3
wavelet-LHL_gldm_GrayLevelNonUniformity	8,77167	1,35E-08	2,02E-08	3
wavelet-LLL_glrlm_GrayLevelNonUniformity	8,83423	1,35E-08	2,02E-08	3
wavelet-HLL_glszm_LowGrayLevelZoneEmphasis	8,80352	1,46E-08	2,17E-08	3
original_gldm_GrayLevelNonUniformity	8,71706	1,53E-08	2,28E-08	3
original_glrlm_GrayLevelNonUniformity	8,77565	1,54E-08	2,29E-08	3
log-sigma-5-0-mm-3D_glszm_SizeZoneNonUniformity	8,77408	1,55E-08	2,29E-08	1
wavelet-LHL_gldm_DependenceNonUniformity	8,76091	1,59E-08	2,36E-08	1
log-sigma-5-0-mm-3D_firstorder_Mean	8,75838	1,61E-08	2,37E-08	3
wavelet-LHH_glrlm_LongRunLowGrayLevelEmphasis	8,75036	1,64E-08	2,41E-08	3
wavelet-LLH_glrlm_LowGrayLevelRunEmphasis	8,72306	1,74E-08	2,56E-08	3
wavelet-LLH_gldm_LargeDependenceLowGrayLevelEmphasis	8,67956	1,91E-08	2,81E-08	3
log-sigma-5-0-mm-3D_glszm_GrayLevelVariance	8,67433	1,94E-08	2,86E-08	1
log-sigma-5-0-mm-3D_gldm_LowGrayLevelEmphasis	8,63272	2,15E-08	3,16E-08	3
log-sigma-4-0-mm-3D_glszm_LowGrayLevelZoneEmphasis	8,63841	2,17E-08	3,17E-08	3
wavelet-LLH_gldm_LowGrayLevelEmphasis	8,61487	2,21E-08	3,24E-08	3
log-sigma-5-0-mm-3D_glrlm_LongRunLowGrayLevelEmphasis	8,59278	2,38E-08	3,48E-08	3
log-sigma-2-0-mm-3D_glszm_LargeAreaEmphasis	8,46081	2,75E-08	4,01E-08	3
wavelet-LHH_gldm_GrayLevelNonUniformity	8,51077	2,78E-08	4,06E-08	3
log-sigma-5-0-mm-3D_firstorder_Median	8,50269	2,84E-08	4,14E-08	3
log-sigma-4-0-mm-3D_glrlm_RunLengthNonUniformity	8,45881	3,13E-08	4,54E-08	1
log-sigma-2-0-mm-3D_glszm_SmallAreaLowGrayLevelEmphasis	8,44528	3,27E-08	4,75E-08	3
log-sigma-3-0-mm-3D_glcm_Autocorrelation	8,38361	3,74E-08	5,42E-08	1
log-sigma-2-0-mm-3D_glszm_ZoneVariance	8,27217	4,21E-08	6,1E-08	3
original_glszm_LowGrayLevelZoneEmphasis	8,29761	4,54E-08	6,56E-08	3
wavelet-HHL_glrlm_LongRunHighGrayLevelEmphasis	8,28856	4,59E-08	6,63E-08	1
log-sigma-3-0-mm-3D_gldm_HighGrayLevelEmphasis	8,27831	4,73E-08	6,82E-08	1
wavelet-HHH_glrlm_ShortRunLowGrayLevelEmphasis	8,27094	4,77E-08	6,88E-08	3
original_glrlm_RunLengthNonUniformity	8,25427	4,94E-08	7,11E-08	1
log-sigma-3-0-mm-3D_glrlm_HighGrayLevelRunEmphasis	8,24972	5,04E-08	7,24E-08	1
original_firstorder_10Percentile	8,22273	5,29E-08	7,6E-08	2
wavelet-LHH_glrlm_RunLengthNonUniformity	8,19811	5,6E-08	8,03E-08	1
wavelet-LLH_glrlm_ShortRunLowGrayLevelEmphasis	8,13312	5,78E-08	8,27E-08	3
log-sigma-3-0-mm-3D_glrlm_RunLengthNonUniformity	8,16775	6E-08	8,58E-08	1
wavelet-HLH_glrlm_ShortRunHighGrayLevelEmphasis	8,13044	6,53E-08	9,32E-08	1
log-sigma-5-0-mm-3D_firstorder_RootMeanSquared	8,11343	6,79E-08	9,69E-08	3
log-sigma-3-0-mm-3D_glszm_LargeAreaEmphasis	8,05302	6,93E-08	9,87E-08	3
wavelet-HHL_glcm_Idn	8,0964	7,07E-08	1E-07	3
wavelet-HLL_glrlm_LongRunHighGrayLevelEmphasis	8,06908	7,52E-08	1,07E-07	1
log-sigma-4-0-mm-3D_firstorder_90Percentile	8,03363	8,12E-08	1,15E-07	1
log-sigma-3-0-mm-3D_glszm_SmallAreaLowGrayLevelEmphasis	7,95285	9,86E-08	1,4E-07	3
wavelet-HLL_glrlm_LowGrayLevelRunEmphasis	7,94011	1E-07	1,42E-07	3
wavelet-LHH_gldm_LowGrayLevelEmphasis	7,93666	1,01E-07	1,43E-07	3
wavelet-LHH_glrlm_LowGrayLevelRunEmphasis	7,91385	1,06E-07	1,5E-07	3
log-sigma-2-0-mm-3D_glcm_Idmn	7,90651	1,08E-07	1,53E-07	3
log-sigma-3-0-mm-3D_glszm_SmallAreaHighGrayLevelEmphasis	7,89854	1,11E-07	1,56E-07	1

log-sigma-2-0-mm-3D_glrlm_RunLengthNonUniformity	7,89129	1,12E-07	1,57E-07	1
wavelet-LHL_glszm_SmallAreaHighGrayLevelEmphasis	7,87699	1,15E-07	1,62E-07	1
wavelet-HHL_firstorder_RootMeanSquared	7,8646	1,19E-07	1,66E-07	1
log-sigma-4-0-mm-3D_glszm_LargeAreaEmphasis	7,8022	1,23E-07	1,72E-07	3
wavelet-LHH_glszm_LargeAreaEmphasis	7,80077	1,23E-07	1,72E-07	3
log-sigma-3-0-mm-3D_glszm_ZoneVariance	7,79773	1,24E-07	1,73E-07	3
log-sigma-2-0-mm-3D_glcm_Imc1	7,84096	1,25E-07	1,75E-07	3
wavelet-HLL_gldm_LowGrayLevelEmphasis	7,8308	1,28E-07	1,79E-07	3
wavelet-HHH_firstorder_Range	7,81557	1,33E-07	1,85E-07	1
wavelet-LLL_glrlm_RunLengthNonUniformity	7,80294	1,36E-07	1,89E-07	1
wavelet-HHL_gldm_LargeDependenceLowGrayLevelEmphasis	7,8001	1,38E-07	1,92E-07	3
wavelet-LHH_glszm_ZoneVariance	7,74362	1,4E-07	1,94E-07	3
wavelet-HLH_glszm_HighGrayLevelZoneEmphasis	7,7233	1,63E-07	2,26E-07	1
log-sigma-5-0-mm-3D_glcm_Imc2	7,72072	1,64E-07	2,27E-07	1
log-sigma-3-0-mm-3D_glszm_HighGrayLevelZoneEmphasis	7,70034	1,73E-07	2,4E-07	1
wavelet-HHH_glcm_JointAverage	7,66671	1,85E-07	2,56E-07	1
wavelet-HLL_glrlm_ShortRunLowGrayLevelEmphasis	7,66235	1,88E-07	2,59E-07	3
log-sigma-2-0-mm-3D_gldm_LargeDependenceLowGrayLevelEmphasis	7,62095	2,05E-07	2,83E-07	3
wavelet-LHL_glrlm_RunLengthNonUniformity	7,5157	2,61E-07	3,58E-07	1
wavelet-HHL_firstorder_TotalEnergy	6,84221	2,83E-07	3,88E-07	1
wavelet-HHL_firstorder_Energy	6,84222	2,83E-07	3,88E-07	1
wavelet-HLH_glszm_ZoneVariance	7,38536	3,17E-07	4,35E-07	3
wavelet-HLL_glszm_LargeAreaEmphasis	7,42724	3,19E-07	4,36E-07	3
log-sigma-3-0-mm-3D_glcm_Idmn	7,42685	3,2E-07	4,38E-07	3
log-sigma-4-0-mm-3D_glszm_ZoneVariance	7,34657	3,48E-07	4,75E-07	3
wavelet-LHL_glrlm_ShortRunHighGrayLevelEmphasis	7,37709	3,57E-07	4,87E-07	1
wavelet-HLL_glszm_ZoneVariance	7,36967	3,63E-07	4,95E-07	3
wavelet-HHH_firstorder_Minimum	7,36653	3,66E-07	4,97E-07	3
wavelet-LHH_glszm_LowGrayLevelZoneEmphasis	7,36103	3,71E-07	5,04E-07	3
wavelet-LLH_glszm_SmallAreaLowGrayLevelEmphasis	7,35331	3,75E-07	5,09E-07	3
wavelet-HLH_glcm_Idn	7,33527	3,95E-07	5,36E-07	3
wavelet-HLH_glszm_LargeAreaEmphasis	7,27229	4,11E-07	5,57E-07	3
wavelet-HLH_gldm_SmallDependenceHighGrayLevelEmphasis	7,26319	4,21E-07	5,69E-07	1
wavelet-LLH_gldm_GrayLevelNonUniformity	7,25048	4,32E-07	5,83E-07	3
wavelet-HLH_glszm_SmallAreaHighGrayLevelEmphasis	7,24236	4,85E-07	6,54E-07	1
wavelet-HHH_glszm_ZoneVariance	7,14777	5,48E-07	7,38E-07	3
wavelet-LHL_glszm_HighGrayLevelZoneEmphasis	7,17018	5,71E-07	7,68E-07	1
log-sigma-5-0-mm-3D_glszm_LargeAreaEmphasis	7,08213	6,39E-07	8,58E-07	3
wavelet-HLH_glrlm_HighGrayLevelRunEmphasis	7,11304	6,5E-07	8,73E-07	1
wavelet-LHL_glrlm_HighGrayLevelRunEmphasis	7,11016	6,54E-07	8,77E-07	1
wavelet-LHL_gldm_HighGrayLevelEmphasis	7,10633	6,6E-07	8,84E-07	1
wavelet-LLH_glrlm_LongRunHighGrayLevelEmphasis	7,07668	7,05E-07	9,43E-07	1
wavelet-HLH_gldm_HighGrayLevelEmphasis	7,07288	7,12E-07	9,52E-07	1
wavelet-LHL_firstorder_RootMeanSquared	7,05092	7,48E-07	9,99E-07	1
wavelet-LHH_glrlm_ShortRunHighGrayLevelEmphasis	7,04136	7,64E-07	1,02E-06	1
wavelet-LLH_glrlm_RunLengthNonUniformity	6,92055	1,01E-06	1,34E-06	1
wavelet-HHL_glszm_GrayLevelNonUniformity	6,85751	1,16E-06	1,54E-06	1

wavelet-LHH_glrlm_ShortRunLowGrayLevelEmphasis	6,8109	1,3E-06	1,72E-06	3
wavelet-LHL_glszm_LargeAreaEmphasis	6,71202	1,5E-06	1,99E-06	3
wavelet-LHL_glcm_Autocorrelation	6,73864	1,52E-06	2,02E-06	1
wavelet-HLL_glcm_Correlation	6,72272	1,58E-06	2,09E-06	2
wavelet-LHL_glcm_Idn	6,69877	1,67E-06	2,21E-06	3
wavelet-HLH_glcm_Autocorrelation	6,69083	1,7E-06	2,25E-06	1
log-sigma-4-0-mm-3D_firstorder_Mean	6,65072	1,86E-06	2,46E-06	3
wavelet-LHL_glszm_ZoneVariance	6,59537	1,97E-06	2,59E-06	3
log-sigma-4-0-mm-3D_glrlm_ShortRunHighGrayLevelEmphasis	6,60414	2,08E-06	2,74E-06	1
wavelet-LHL_glszm_LowGrayLevelZoneEmphasis	6,58599	2,17E-06	2,85E-06	3
log-sigma-4-0-mm-3D_glcm_Autocorrelation	6,5496	2,36E-06	3,09E-06	1
log-sigma-5-0-mm-3D_gldm_LargeDependenceLowGrayLevelEmphasis	6,50119	2,67E-06	3,5E-06	3
log-sigma-5-0-mm-3D_glszm_ZoneVariance	6,45746	2,69E-06	3,53E-06	3
wavelet-HHL_glcm_Idmn	6,488	2,71E-06	3,54E-06	3
wavelet-LLL_glcm_Idmn	6,45796	2,9E-06	3,78E-06	3
wavelet-HLL_firstorder_TotalEnergy	5,97686	2,93E-06	3,82E-06	1
wavelet-HLL_firstorder_Energy	5,97688	2,93E-06	3,82E-06	1
log-sigma-4-0-mm-3D_gldm_HighGrayLevelEmphasis	6,38304	3,45E-06	4,49E-06	1
original_glcm_Idmn	6,36594	3,57E-06	4,65E-06	3
log-sigma-4-0-mm-3D_glrlm_HighGrayLevelRunEmphasis	6,33015	3,9E-06	5,06E-06	1
log-sigma-4-0-mm-3D_firstorder_Median	6,24358	4,73E-06	6,14E-06	3
log-sigma-4-0-mm-3D_glcm_Idn	6,20668	5,15E-06	6,67E-06	3
log-sigma-4-0-mm-3D_glszm_LargeAreaLowGrayLevelEmphasis	6,20397	5,17E-06	6,69E-06	3
wavelet-LHH_glszm_SmallAreaHighGrayLevelEmphasis	6,19293	5,3E-06	6,86E-06	1
wavelet-HLL_gldm_LargeDependenceLowGrayLevelEmphasis	6,18656	5,4E-06	6,97E-06	3
original_glszm_SmallAreaLowGrayLevelEmphasis	6,1729	5,58E-06	7,19E-06	3
wavelet-LHL_glcm_ClusterProminence	6,12352	6,22E-06	8,01E-06	1
wavelet-HHH_firstorder_Maximum	6,11676	6,32E-06	8,14E-06	1
log-sigma-4-0-mm-3D_firstorder_RootMeanSquared	6,11193	6,4E-06	8,22E-06	3
wavelet-HHL_glszm_LargeAreaLowGrayLevelEmphasis	6,10211	6,52E-06	8,37E-06	3
wavelet-HLL_glcm_Idn	6,09005	6,73E-06	8,63E-06	3
log-sigma-2-0-mm-3D_firstorder_TotalEnergy	5,65411	6,96E-06	8,9E-06	1
log-sigma-2-0-mm-3D_firstorder_Energy	5,65411	6,96E-06	8,9E-06	1
original_glszm_LargeAreaEmphasis	6,02326	7,85E-06	1E-05	3
wavelet-LLH_glszm_LargeAreaEmphasis	5,98878	7,95E-06	1,01E-05	3
original_glszm_ZoneVariance	5,97538	8,76E-06	1,12E-05	3
wavelet-HLL_glszm_SmallAreaLowGrayLevelEmphasis	5,97402	8,79E-06	1,12E-05	3
wavelet-LLL_glszm_LowGrayLevelZoneEmphasis	5,97309	8,8E-06	1,12E-05	3
wavelet-LLH_glszm_LargeAreaLowGrayLevelEmphasis	5,95619	9,22E-06	1,17E-05	3
wavelet-LHL_glrlm_LongRunHighGrayLevelEmphasis	5,91865	9,96E-06	1,26E-05	1
wavelet-HLL_firstorder_RootMeanSquared	5,91079	1,01E-05	1,29E-05	1
wavelet-LLH_glszm_ZoneVariance	5,85603	1,08E-05	1,37E-05	3
log-sigma-3-0-mm-3D_glszm_LargeAreaLowGrayLevelEmphasis	5,84357	1,18E-05	1,49E-05	3
wavelet-HLH_glszm_GrayLevelNonUniformity	5,83655	1,2E-05	1,52E-05	1
wavelet-LHH_glcm_Correlation	5,79524	1,32E-05	1,67E-05	2
wavelet-LHH_glszm_HighGrayLevelZoneEmphasis	5,79293	1,33E-05	1,67E-05	1
log-sigma-5-0-mm-3D_glszm_LargeAreaLowGrayLevelEmphasis	5,77085	1,4E-05	1,76E-05	3

wavelet-HLH_glcm_Correlation	5,77045	1,4E-05	1,77E-05	2
original_glrlm_LongRunLowGrayLevelEmphasis	5,6657	1,79E-05	2,25E-05	3
wavelet-LLL_glszm_SmallAreaLowGrayLevelEmphasis	5,55796	2,28E-05	2,86E-05	3
log-sigma-2-0-mm-3D_glszm_LargeAreaLowGrayLevelEmphasis	5,53021	2,43E-05	3,04E-05	3
wavelet-LHL_glszm_SmallAreaLowGrayLevelEmphasis	5,50395	2,59E-05	3,24E-05	3
wavelet-HHL_glcm_ClusterProminence	5,47455	2,61E-05	3,26E-05	1
wavelet-LHL_firstorder_Kurtosis	5,42108	2,97E-05	3,71E-05	3
wavelet-LHH_glszm_SmallAreaEmphasis	5,43883	3E-05	3,75E-05	1
wavelet-LHH_glszm_SmallAreaLowGrayLevelEmphasis	5,40955	3,21E-05	4E-05	3
wavelet-LLH_firstorder_TotalEnergy	5,07505	3,23E-05	4,01E-05	1
wavelet-LLH_firstorder_Energy	5,07505	3,23E-05	4,01E-05	1
log-sigma-3-0-mm-3D_glcm_ClusterProminence	5,33477	3,81E-05	4,73E-05	1
wavelet-HHH_glszm_GrayLevelNonUniformity	5,30936	3,84E-05	4,76E-05	1
wavelet-HLH_glcm_Imc1	5,27162	4,41E-05	5,46E-05	3
log-sigma-4-0-mm-3D_glszm_HighGrayLevelZoneEmphasis	5,24658	4,69E-05	5,8E-05	1
log-sigma-4-0-mm-3D_gldm_SmallDependenceHighGrayLevelEmphasis	5,24347	4,7E-05	5,82E-05	1
wavelet-LHH_glrlm_HighGrayLevelRunEmphasis	5,12767	6,13E-05	7,58E-05	1
log-sigma-5-0-mm-3D_firstorder_Maximum	5,12528	6,18E-05	7,62E-05	1
wavelet-LHH_gldm_HighGrayLevelEmphasis	5,10517	6,46E-05	7,96E-05	1
original_gldm_LargeDependenceLowGrayLevelEmphasis	5,04525	7,43E-05	9,14E-05	3
wavelet-LHL_glrlm_LongRunLowGrayLevelEmphasis	5,04412	7,45E-05	9,17E-05	3
log-sigma-5-0-mm-3D_glcm_Autocorrelation	4,99365	8,37E-05	0,000103	1
log-sigma-3-0-mm-3D_firstorder_Mean	4,84908	0,000117	0,000143	3
log-sigma-5-0-mm-3D_gldm_HighGrayLevelEmphasis	4,84231	0,000119	0,000145	1
wavelet-LLH_glcm_Imc1	4,82993	0,000122	0,000149	3
original_glszm_GrayLevelNonUniformity	4,82035	0,000124	0,000152	1
log-sigma-4-0-mm-3D_glszm_SmallAreaHighGrayLevelEmphasis	4,80487	0,000129	0,000158	1
original_glrlm_LowGrayLevelRunEmphasis	4,79229	0,000133	0,000163	3
log-sigma-5-0-mm-3D_glrlm_HighGrayLevelRunEmphasis	4,75362	0,000145	0,000177	1
wavelet-LHH_firstorder_Kurtosis	4,7204	0,000157	0,000191	3
log-sigma-5-0-mm-3D_glrlm_ShortRunHighGrayLevelEmphasis	4,69965	0,000165	0,0002	1
log-sigma-2-0-mm-3D_glrlm_LongRunHighGrayLevelEmphasis	4,6826	0,000171	0,000208	1
log-sigma-3-0-mm-3D_firstorder_TotalEnergy	4,395	0,00019	0,00023	1
log-sigma-3-0-mm-3D_firstorder_Energy	4,395	0,00019	0,00023	1
wavelet-LHH_glcm_Autocorrelation	4,56963	0,000221	0,000268	1
original_gldm_LowGrayLevelEmphasis	4,56874	0,000223	0,000269	3
wavelet-LLL_firstorder_Skewness	4,52928	0,000243	0,000294	3
wavelet-HHH_glszm_LargeAreaEmphasis	4,52032	0,000248	0,0003	3
wavelet-HHL_firstorder_Kurtosis	4,49732	0,000253	0,000305	3
original_glrlm_ShortRunLowGrayLevelEmphasis	4,50846	0,000256	0,000308	3
log-sigma-5-0-mm-3D_glcm_Idn	4,50627	0,000256	0,000308	3
log-sigma-3-0-mm-3D_firstorder_RootMeanSquared	4,50005	0,00026	0,000313	3
wavelet-LHL_gldm_LargeDependenceLowGrayLevelEmphasis	4,48423	0,000271	0,000326	3
wavelet-HLH_firstorder_Kurtosis	4,39806	0,00033	0,000396	3
log-sigma-3-0-mm-3D_firstorder_Median	4,38316	0,00034	0,000408	3
wavelet-LHL_glrlm_LowGrayLevelRunEmphasis	4,38264	0,000341	0,000408	3
wavelet-LHH_glszm_SizeZoneNonUniformityNormalized	4,32487	0,000389	0,000465	1

wavelet-LHL_gldm_LowGrayLevelEmphasis	4,26009	0,000451	0,000539	3
log-sigma-5-0-mm-3D_firstorder_90Percentile	4,25352	0,000458	0,000546	1
log-sigma-2-0-mm-3D_firstorder_RootMeanSquared	4,21221	0,000503	0,0006	3
wavelet-LHL_glrlm_ShortRunLowGrayLevelEmphasis	4,19672	0,000522	0,000621	3
log-sigma-2-0-mm-3D_firstorder_Kurtosis	4,15416	0,000559	0,000665	3
wavelet-LHH_glcm_Imc1	4,14883	0,000582	0,000691	3
wavelet-LHL_glcm_Idmn	4,13139	0,000606	0,000719	3
wavelet-LLL_gldm_LargeDependenceHighGrayLevelEmphasis	4,11915	0,000623	0,000738	2
wavelet-HLL_firstorder_Kurtosis	4,08983	0,000648	0,000767	3
log-sigma-4-0-mm-3D_glszm_SmallAreaLowGrayLevelEmphasis	4,10109	0,000651	0,00077	3
wavelet-HLH_glcm_ClusterProminence	4,08283	0,000658	0,000778	1
wavelet-LHH_glcm_Idmn	4,0688	0,000699	0,000825	3
log-sigma-3-0-mm-3D_glrlm_LongRunHighGrayLevelEmphasis	4,03159	0,000761	0,000897	1
wavelet-LHH_glszm_LargeAreaLowGrayLevelEmphasis	3,95372	0,000908	0,00107	3
original_glcm_Correlation	3,94252	0,000931	0,001096	2
wavelet-HLH_firstorder_RootMeanSquared	3,88486	0,001063	0,001249	2
wavelet-HLL_glcm_ClusterShade	3,86521	0,001111	0,001304	3
wavelet-LLH_glcm_Idn	3,83262	0,001197	0,001405	3
original_glszm_LargeAreaHighGrayLevelEmphasis	3,79923	0,001263	0,001479	3
wavelet-HLL_glcm_ClusterProminence	3,80875	0,001263	0,001479	1
original_firstorder_Skewness	3,79152	0,001314	0,001536	3
log-sigma-5-0-mm-3D_glrlm_LongRunHighGrayLevelEmphasis	3,78055	0,00135	0,001577	1
log-sigma-2-0-mm-3D_firstorder_Median	3,76712	0,00139	0,001622	3
wavelet-HHH_gldm_SmallDependenceHighGrayLevelEmphasis	3,74972	0,001413	0,001648	1
wavelet-HHH_glcm_Imc1	3,74446	0,001463	0,001704	3
wavelet-HHH_glszm_SizeZoneNonUniformityNormalized	3,68616	0,001663	0,001935	3
log-sigma-2-0-mm-3D_firstorder_Mean	3,681	0,001691	0,001965	3
wavelet-LHL_glcm_Correlation	3,67692	0,001707	0,001982	2
log-sigma-4-0-mm-3D_glrlm_LongRunHighGrayLevelEmphasis	3,65183	0,001808	0,002096	1
wavelet-LHL_glszm_LargeAreaLowGrayLevelEmphasis	3,48963	0,002606	0,003019	3
wavelet-HHH_glcm_ClusterProminence	3,45386	0,002774	0,003211	1
original_firstorder_Kurtosis	3,43728	0,002896	0,003349	3
wavelet-LHH_glszm_GrayLevelNonUniformity	3,43797	0,002931	0,003385	1
wavelet-LLL_gldm_LargeDependenceLowGrayLevelEmphasis	3,4367	0,002943	0,003396	3
wavelet-LHL_glcm_ClusterShade	3,43031	0,002984	0,003439	3
log-sigma-5-0-mm-3D_glszm_HighGrayLevelZoneEmphasis	3,42673	0,003008	0,003463	1
log-sigma-4-0-mm-3D_firstorder_TotalEnergy	3,29643	0,003032	0,003484	1
log-sigma-4-0-mm-3D_firstorder_Energy	3,29643	0,003032	0,003484	1
wavelet-LLL_glszm_LargeAreaHighGrayLevelEmphasis	3,20531	0,004869	0,005589	3
wavelet-LLL_glszm_LargeAreaEmphasis	3,18637	0,005159	0,005916	3
wavelet-HLL_firstorder_Mean	3,17847	0,005251	0,006015	3
wavelet-HLL_glszm_GrayLevelNonUniformity	3,16803	0,005375	0,006151	1
log-sigma-3-0-mm-3D_firstorder_Kurtosis	3,10428	0,006114	0,00699	3
log-sigma-3-0-mm-3D_glcm_Imc1	3,10163	0,006235	0,00712	3
wavelet-LLL_firstorder_Kurtosis	3,0785	0,0065	0,007416	3
wavelet-HLL_glcm_Idmn	3,01554	0,007555	0,00861	3
wavelet-HHH_gldm_DependenceNonUniformity	2,95117	0,008712	0,009918	3

wavelet-LLL_glrlm_LowGrayLevelRunEmphasis	2,9492	0,008756	0,009959	3
wavelet-LLL_glrlm_LongRunLowGrayLevelEmphasis	2,9446	0,008847	0,010052	3
log-sigma-5-0-mm-3D_gldm_SmallDependenceHighGrayLevelEmphasis	2,92787	0,009172	0,01041	1
wavelet-LLL_glrlm_ShortRunLowGrayLevelEmphasis	2,92688	0,009199	0,010431	3
log-sigma-5-0-mm-3D_glszm_SmallAreaHighGrayLevelEmphasis	2,90576	0,009636	0,010915	1
wavelet-HHL_gldm_SmallDependenceLowGrayLevelEmphasis	2,8869	0,010048	0,01137	3
wavelet-LLH_glcm_Correlation	2,85057	0,01088	0,012299	2
wavelet-LLL_gldm_SmallDependenceLowGrayLevelEmphasis	2,80991	0,011903	0,013441	3
log-sigma-5-0-mm-3D_firstorder_Kurtosis	2,79118	0,012265	0,013837	3
wavelet-LLL_gldm_LowGrayLevelEmphasis	2,78507	0,012576	0,014174	3
wavelet-HLL_gldm_SmallDependenceLowGrayLevelEmphasis	2,75445	0,013444	0,015137	3
log-sigma-3-0-mm-3D_gldm_SmallDependenceLowGrayLevelEmphasis	2,74991	0,013582	0,015276	2
wavelet-LLH_firstorder_Skewness	2,73127	0,014141	0,015889	2
wavelet-LLL_glszm_ZoneVariance	2,70894	0,014852	0,016671	3
wavelet-HHH_glrlm_ShortRunHighGrayLevelEmphasis	2,69644	0,015102	0,016935	1
original_glszm_LargeAreaLowGrayLevelEmphasis	2,69509	0,015301	0,01714	3
wavelet-HLH_glrlm_LongRunHighGrayLevelEmphasis	2,63645	0,017401	0,019474	1
wavelet-HLH_glcm_Idmn	2,6132	0,018317	0,020479	3
log-sigma-5-0-mm-3D_glszm_LargeAreaHighGrayLevelEmphasis	2,54098	0,018428	0,020582	3
log-sigma-4-0-mm-3D_glcm_Idmn	2,58089	0,019633	0,021905	3
wavelet-HLL_glszm_LargeAreaLowGrayLevelEmphasis	2,56895	0,020145	0,022455	3
wavelet-LLL_glszm_LargeAreaLowGrayLevelEmphasis	2,55885	0,020588	0,022926	3
wavelet-HLH_glszm_LargeAreaLowGrayLevelEmphasis	2,55426	0,020796	0,023134	3
log-sigma-5-0-mm-3D_firstorder_TotalEnergy	2,47077	0,021665	0,024054	1
log-sigma-5-0-mm-3D_firstorder_Energy	2,47077	0,021666	0,024054	1
log-sigma-2-0-mm-3D_glszm_LargeAreaHighGrayLevelEmphasis	2,42346	0,024146	0,026781	3
wavelet-HHH_glszm_HighGrayLevelZoneEmphasis	2,44185	0,026277	0,029115	1
wavelet-LHL_glszm_GrayLevelNonUniformity	2,41096	0,02831	0,031337	1
wavelet-HHH_glcm_Correlation	2,39664	0,029195	0,032285	2
log-sigma-4-0-mm-3D_glcm_ClusterShade	2,38065	0,03021	0,033374	3
log-sigma-2-0-mm-3D_glcm_Correlation	2,36422	0,031278	0,034486	2
wavelet-LHL_gldm_LargeDependenceHighGrayLevelEmphasis	2,36106	0,031254	0,034486	3
log-sigma-4-0-mm-3D_glcm_ClusterProminence	2,36175	0,031445	0,034636	1
log-sigma-5-0-mm-3D_gldm_LargeDependenceHighGrayLevelEmphasis	2,34716	0,032202	0,035435	3
log-sigma-4-0-mm-3D_firstorder_Kurtosis	2,30542	0,035213	0,03871	3
wavelet-HHH_glrlm_HighGrayLevelRunEmphasis	2,30474	0,035248	0,03871	1
wavelet-HLH_glszm_SmallAreaEmphasis	2,29532	0,036254	0,039777	1
wavelet-HHH_gldm_HighGrayLevelEmphasis	2,28859	0,03648	0,039985	1
wavelet-LLH_firstorder_Kurtosis	2,2904	0,036595	0,040072	3
wavelet-HLH_firstorder_Median	2,27391	0,037892	0,041451	2
wavelet-LLH_glszm_LargeAreaHighGrayLevelEmphasis	2,18418	0,041397	0,045241	3
log-sigma-5-0-mm-3D_firstorder_Skewness	2,21968	0,042242	0,046101	2
wavelet-HHL_glszm_LargeAreaHighGrayLevelEmphasis	2,17482	0,042266	0,046101	3
wavelet-HHH_firstorder_Kurtosis	2,20483	0,043564	0,047471	3
wavelet-HLH_firstorder_Mean	2,18544	0,045649	0,049694	2

wavelet-HLH_gldm_LargeDependenceHighGrayLevelEmphasis	2,17556	0,046621	0,050702	n.s.
log-sigma-4-0-mm-3D_glszm_LargeAreaHighGrayLevelEmphasis	2,12377	0,04731	0,051402	n.s.
wavelet-HHH_glcm_Autocorrelation	2,16425	0,047436	0,051489	n.s.
log-sigma-3-0-mm-3D_glszm_LargeAreaHighGrayLevelEmphasis	2,10836	0,04894	0,05307	n.s.
log-sigma-5-0-mm-3D_glcm_ClusterShade	2,13	0,051264	0,055536	n.s.
log-sigma-2-0-mm-3D_gldm_LargeDependenceHighGrayLevelEmphasis	2,09888	0,054396	0,058872	n.s.
wavelet-HHH_glszm_SmallAreaHighGrayLevelEmphasis	2,08015	0,056544	0,061138	n.s.
wavelet-HHH_firstorder_RootMeanSquared	2,04185	0,061525	0,066459	n.s.
wavelet-HLL_firstorder_Median	2,03627	0,06193	0,066832	n.s.
wavelet-HLL_glszm_LargeAreaHighGrayLevelEmphasis	1,9846	0,064062	0,069066	n.s.
wavelet-HLH_glszm_LargeAreaHighGrayLevelEmphasis	1,97412	0,065525	0,070575	n.s.
wavelet-LLL_firstorder_Minimum	1,99864	0,06725	0,072364	n.s.
original_gldm_LargeDependenceHighGrayLevelEmphasis	1,99056	0,068388	0,073517	n.s.
log-sigma-4-0-mm-3D_glcm_Correlation	1,93837	0,076055	0,081681	n.s.
wavelet-HHH_glszm_LargeAreaHighGrayLevelEmphasis	1,90142	0,076542	0,08209	n.s.
wavelet-LHL_firstorder_Median	1,93476	0,076616	0,08209	n.s.
wavelet-LHH_glcm_ClusterShade	1,93274	0,076657	0,08209	n.s.
wavelet-HLL_firstorder_Skewness	1,93191	0,077049	0,082431	n.s.
wavelet-HHL_firstorder_Skewness	1,93107	0,077197	0,082511	n.s.
wavelet-LHH_glszm_LargeAreaHighGrayLevelEmphasis	1,88728	0,078874	0,084223	n.s.
wavelet-HHL_firstorder_Mean	1,91887	0,079119	0,084404	n.s.
wavelet-HHH_firstorder_Median	1,90342	0,081651	0,087022	n.s.
wavelet-LHH_gldm_LargeDependenceHighGrayLevelEmphasis	1,89775	0,082581	0,087929	n.s.
original_glcm_ClusterShade	1,89184	0,08357	0,088897	n.s.
log-sigma-2-0-mm-3D_glcm_ClusterShade	1,88494	0,084393	0,089687	n.s.
log-sigma-3-0-mm-3D_glcm_ClusterShade	1,8758	0,086331	0,09166	n.s.
log-sigma-4-0-mm-3D_firstorder_Skewness	1,83247	0,093837	0,099535	n.s.
log-sigma-5-0-mm-3D_glcm_Correlation	1,82636	0,095326	0,101018	n.s.
wavelet-LLH_firstorder_Median	1,81842	0,096484	0,102149	n.s.
wavelet-LLH_firstorder_Mean	1,80806	0,098879	0,104487	n.s.
wavelet-LLL_glcm_Correlation	1,80811	0,098863	0,104487	n.s.
log-sigma-5-0-mm-3D_glcm_ClusterProminence	1,76007	0,108754	0,114813	n.s.
wavelet-LHH_firstorder_Skewness	1,73353	0,114599	0,12087	n.s.
wavelet-LHL_glszm_LargeAreaHighGrayLevelEmphasis	1,69954	0,116589	0,122852	n.s.
wavelet-LLL_glcm_ClusterShade	1,7164	0,118514	0,124763	n.s.
log-sigma-4-0-mm-3D_gldm_LargeDependenceHighGrayLevelEmphasis	1,69135	0,124109	0,130531	n.s.
log-sigma-3-0-mm-3D_gldm_LargeDependenceHighGrayLevelEmphasis	1,64915	0,134752	0,141591	n.s.
log-sigma-5-0-mm-3D_gldm_SmallDependenceLowGrayLevelEmphasis	1,6191	0,143184	0,15031	n.s.
log-sigma-4-0-mm-3D_gldm_SmallDependenceLowGrayLevelEmphasis	1,54867	0,163799	0,171789	n.s.
wavelet-HHH_glcm_Idn	1,5264	0,170846	0,179012	n.s.
original_gldm_SmallDependenceLowGrayLevelEmphasis	1,52365	0,171732	0,179772	n.s.
wavelet-HHH_glszm_LargeAreaLowGrayLevelEmphasis	1,5184	0,173467	0,181418	n.s.
wavelet-LLH_glcm_Idmn	1,51423	0,174795	0,182636	n.s.

log-sigma-2-0-mm-3D_gldm_SmallDependenceLowGrayLevelEmphasis	1,48485	0,184688	0,192793	n.s.
wavelet-LHL_firstorder_Skewness	1,46939	0,190057	0,198061	n.s.
wavelet-HLL_gldm_LargeDependenceHighGrayLevelEmphasis	1,46827	0,190089	0,198061	n.s.
wavelet-LLH_firstorder_RootMeanSquared	1,44542	0,198705	0,206845	n.s.
log-sigma-3-0-mm-3D_glcm_Correlation	1,39257	0,218915	0,227671	n.s.
original_glcm_ClusterProminence	1,36096	0,226208	0,235038	n.s.
wavelet-HHH_firstorder_TotalEnergy	1,34039	0,23502	0,243741	n.s.
wavelet-HHH_firstorder_Energy	1,34038	0,235021	0,243741	n.s.
wavelet-LHH_firstorder_TotalEnergy	1,32529	0,241659	0,250161	n.s.
wavelet-LHH_firstorder_Energy	1,32529	0,241658	0,250161	n.s.
wavelet-LHL_firstorder_Mean	1,31419	0,252052	0,26068	n.s.
wavelet-HLH_gldm_SmallDependenceLowGrayLevelEmphasis	1,31019	0,25386	0,262306	n.s.
wavelet-LHH_firstorder_Median	1,30728	0,254833	0,263068	n.s.
wavelet-HHL_gldm_LargeDependenceHighGrayLevelEmphasis	1,30588	0,255804	0,263827	n.s.
wavelet-HHL_glcm_Correlation	1,29508	0,260713	0,268643	n.s.
wavelet-LLL_glcm_ClusterProminence	1,24368	0,280219	0,288476	n.s.
wavelet-LLH_gldm_SmallDependenceLowGrayLevelEmphasis	1,24003	0,287079	0,295266	n.s.
wavelet-HLH_firstorder_TotalEnergy	1,18644	0,310015	0,31827	n.s.
wavelet-HLH_firstorder_Energy	1,18644	0,310013	0,31827	n.s.
wavelet-LHL_gldm_SmallDependenceLowGrayLevelEmphasis	1,18263	0,316746	0,324883	n.s.
wavelet-HLH_glszm_SizeZoneNonUniformityNormalized	1,18108	0,317617	0,325478	n.s.
log-sigma-5-0-mm-3D_glszm_SmallAreaLowGrayLevelEmphasis	1,13544	0,342792	0,350954	n.s.
wavelet-LLH_glcm_ClusterShade	1,1274	0,347447	0,355394	n.s.
log-sigma-5-0-mm-3D_glcm_Idmn	1,1089	0,358203	0,366061	n.s.
wavelet-HHH_glcm_ClusterShade	1,06077	0,38679	0,394914	n.s.
log-sigma-5-0-mm-3D_glcm_Imc1	1,01437	0,416883	0,425251	n.s.
wavelet-HLH_firstorder_Skewness	0,97381	0,443925	0,452423	n.s.
original_firstorder_Minimum	0,9661	0,449193	0,457375	n.s.
wavelet-HHH_gldm_SmallDependenceLowGrayLevelEmphasis	0,96383	0,450744	0,458537	n.s.
wavelet-HHH_glrlm_LongRunHighGrayLevelEmphasis	0,95974	0,453373	0,460792	n.s.
wavelet-HHH_firstorder_Mean	0,95804	0,454725	0,461746	n.s.
wavelet-HHH_glszm_SmallAreaEmphasis	0,95143	0,459148	0,465814	n.s.
wavelet-HHH_glcm_Idmn	0,94396	0,464511	0,470828	n.s.
wavelet-LLH_gldm_LargeDependenceHighGrayLevelEmphasis	0,90516	0,492065	0,498305	n.s.
log-sigma-3-0-mm-3D_firstorder_Skewness	0,88488	0,506843	0,512806	n.s.
log-sigma-2-0-mm-3D_firstorder_Skewness	0,84949	0,533107	0,538891	n.s.
wavelet-HHL_glcm_ClusterShade	0,7537	0,607061	0,613093	n.s.
wavelet-HHH_firstorder_Skewness	0,7389	0,618823	0,624408	n.s.
log-sigma-4-0-mm-3D_glcm_Imc1	0,73398	0,622764	0,627818	n.s.
wavelet-HHH_gldm_LargeDependenceHighGrayLevelEmphasis	0,698	0,651491	0,656186	n.s.
wavelet-HHH_gldm_DependenceNonUniformityNormalized	0,64126	0,697132	0,701525	n.s.
wavelet-LHH_firstorder_Mean	0,6255	0,709778	0,713608	n.s.
wavelet-HLH_glcm_ClusterShade	0,60843	0,723459	0,726709	n.s.
wavelet-LHH_gldm_SmallDependenceLowGrayLevelEmphasis	0,489	0,816172	0,819103	n.s.
wavelet-HHL_firstorder_Median	0,43442	0,855515	0,857817	n.s.
wavelet-LHH_firstorder_RootMeanSquared	0,41979	0,865376	0,866927	n.s.
wavelet-LHH_glrlm_LongRunHighGrayLevelEmphasis	0,32199	0,925015	0,925843	n.s.

wavelet-HHH_gldm_DependenceVariance	0,26616	0,952187	0,952187	n.s.
