# Supplementary figures and images for: Contrast Agent Dynamics Determine Radiomics Profiles in Oncologic Imaging
Source: Cancers (Basel). 2024 Apr 16;16(8):1519. doi: 10.3390/cancers16081519 (PMC11049400; doi:10.3390/cancers16081519)

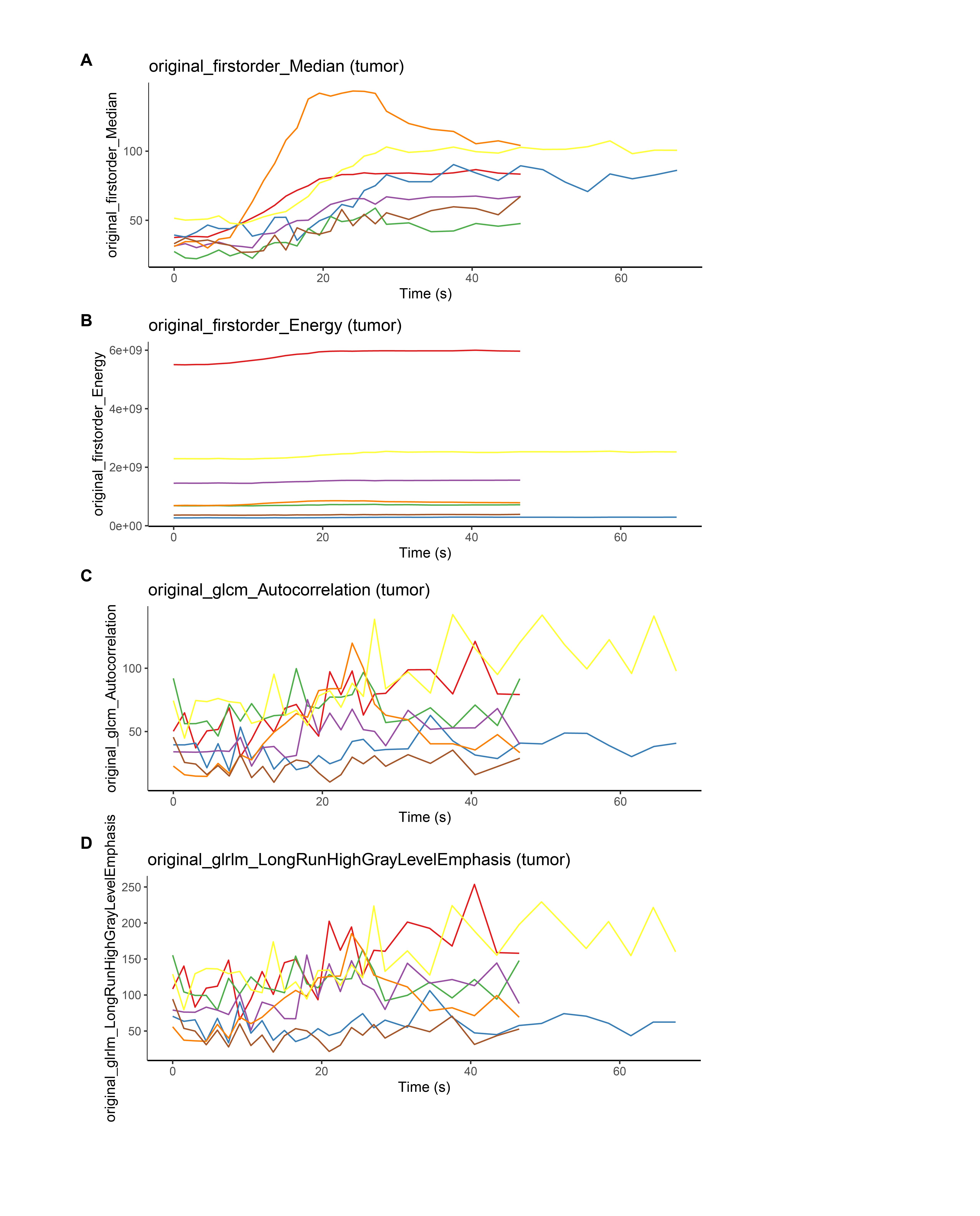

Supplement: Supplementary file 1 [file cancers-16-01519-s001.zip › Supp_Figure_S1-01.jpg]

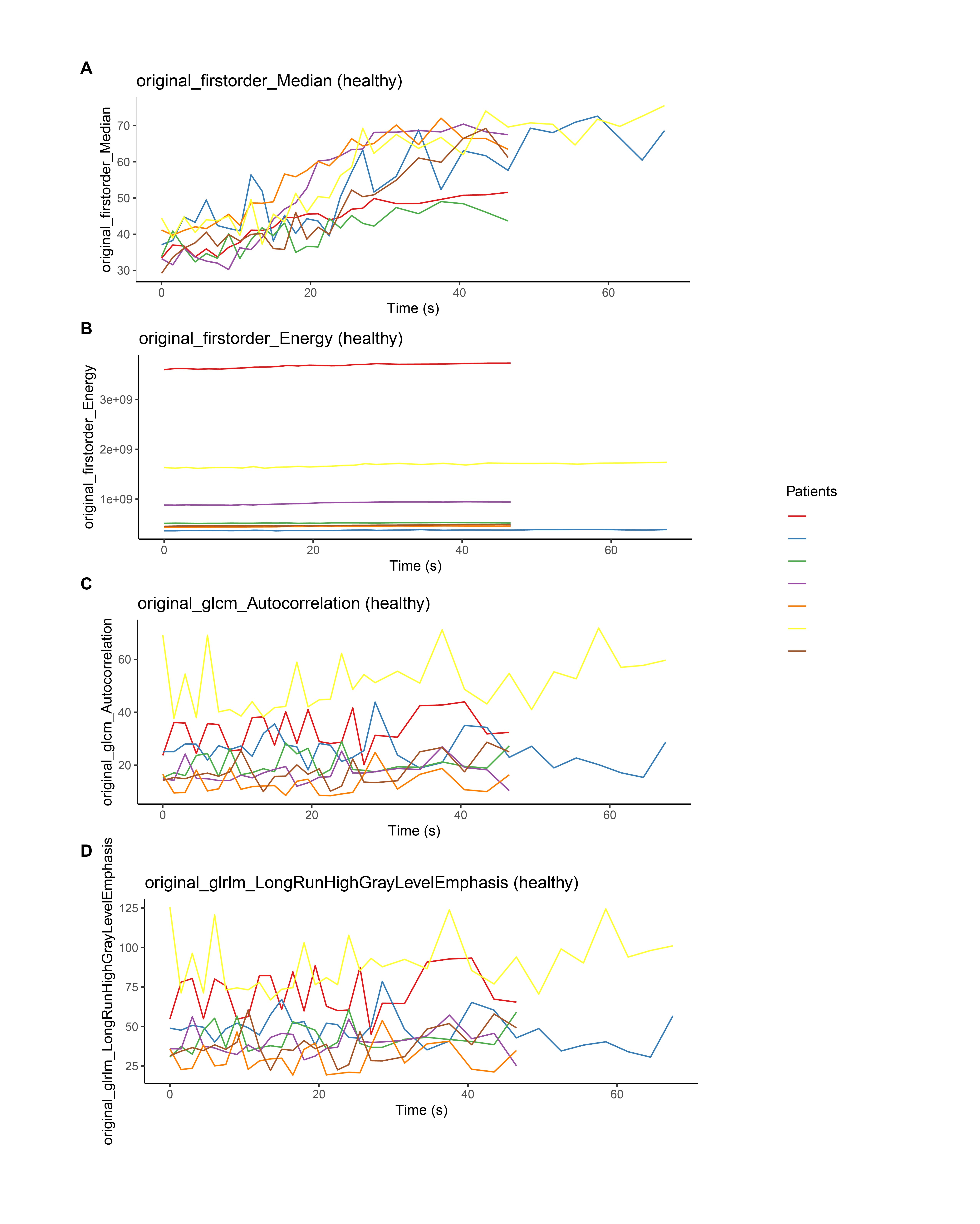

Supplement: Supplementary file 1 [file cancers-16-01519-s001.zip › Supp_Figure_S2-01.jpg]
